# Research on persimmon fruit diameter accurate detection method based on improved RCNN instance segmentation algorithm

**DOI:** 10.3389/fpls.2025.1636727

**Published:** 2025-08-29

**Authors:** Yuan Fang, Yangyang Liu, Ya Feng, Yougen Chen, Haikun Jiang

**Affiliations:** ^1^ School of Mechanical Engineering, Anhui University of Technology, Ma’anshan, China; ^2^ School of Horticulture College, Anhui Agricultural University, Hefei, China; ^3^ College of Information Engineering, Shaoxing Vocational & Technical College, Shaoxing, China; ^4^ Institute of Vegetables, Anhui Academy of Agricultural Sciences, Hefei, China

**Keywords:** persimmon recognition, fruit diameter detection, Mask RCNN, instance segmentation algorithm, binarization

## Abstract

Aiming at the problem of inaccurate fruit recognition and fruit diameter detection in the persimmon inspection process, this research proposes a novel persimmon accurate recognition and fruit diameter detection algorithm based on the Region-based Convolutional Neural Network (RCNN) Mask and instance segmentation algorithm. The algorithm strategically targets the object of interest by integrating cropping, morphological processing, and concave point segmentation modules into the fully connected layer following the Region of Interest (RoI) feature. Initially, the algorithm separates the front and back background of the cropped target object using morphological processing to obtain a binarized image. Subsequently, concave point segmentation is applied to address sticking issues arising from overlapping or occlusion between fruits, while a template matching algorithm helps in image recognition. The improved instance segmentation algorithm enhances the segmentation accuracy of the target fruit and reduces the relative error in the fruit diameter measurement caused by sticking problems during occlusion and overlap. Notably, compared with the original algorithm, the improved Mask RCNN instance segmentation algorithm achieves a mean Average Precision (mAP) of 94.25%, representing an improvement of 8.05%, with the Mean Intersection-over-Union (MIoU) value increasing by 18.5%. The maximum relative error in fruit diameter measurement is reduced to 1.3%, while the maximum relative error in fruit thickness measurement is 1.98%, meeting the stringent requirements of orchard inspection. Overall, the proposed method enhances the precision and accuracy of fruit diameter detection, offering valuable theoretical and technical insights for intelligent inspection, yield estimation, fruit detection, and mechanized picking in the agricultural domain.

## 1 Introduction

An orchard is a compound ecosystem, playing a vital role in the development of the rural economy in China. The digitalization, informatization, and intelligent management of orchards are important foundation for the development of such modern ecosystems (Faria et al., 2025; Wang, 2025; Zhenyu et al., 2025). Moreover, China is one of the main persimmon-producing countries in the world, and the efficient identification, precise positioning, and accurate detection of the fruit diameter of persimmons is essential to realize the intelligent orchard (Zhang et al., 2022; Mao et al., 2025). In a complex natural environment, persimmons face serious problems, such as mutual shading and sticking, restricting the intelligent development of the orchard.

With the innovative development of Deep Learning (DL) recognition and detection technology in the agriculture domain, effective technical support is provided for the intelligent development of orchards (Bai et al., 2023; Fu et al., 2024; Ling et al., 2024). Therefore, local and international researchers have made some progress in research on fruit recognition and classification in complex orchard environments, utilizing neural network models, such as Faster Region-based Convolutional Neural Network (RCNN) and You Only Look Once (YOLO) algorithms, for fruit target detection in highly complex scenes (Tian et al., 2023; Hou et al., 2024; Ullah et al., 2024).

Xu et al (Xu et al., 2022). and Zhou et al (Zhou et al., 2021). proposed an improved masked RCNN algorithm to identify cherry tomatoes by modifying the input layer of the network. Therefore, they performed bimodal data fusion of RGB and depth images, yielding good cherry tomato recognition results in the case where the fruit is adhered to the stem. However, this technique does not investigate the fruit diameter and is not able to meet the recognition requirements of the current study. Moreover, Yang et al (Yang et al., 2022). proposed a fast recognition algorithm for multi-apple targets in dense scenes, applying an improved Center Net model with the absence of an anchor frame to achieve accurate detection of apples in dense scenes; however, this method did not identify and detect fruits in the case of branch and leaf occlusion. Furthermore, Song et al (Song et al., 2022). introduced a fast and accurate localization algorithm for oil tea fruits in a natural and complex scene. Although this fruit is small, densely distributed, and colorful, the accuracy of the YOLOv5 Convolutional Neural Network (CNN) algorithm was high, but the algorithm’s performance was limited in uneven light environments.

Regarding the Mask RCNN neural network model (Blok et al., 2021), it is mostly applied in detecting strawberries, apples, and pears. For instance, Chen et al (Chen et al., 2022). proposed a novel citrus fruit ripeness method combining visual saliency and CNNs. Initially, this method recognizes citrus fruits in an image using the YOLOv5 algorithm. Then, the visual saliency detection algorithm is improved, and a saliency map of the fruit is generated. Consequently, a four-channel ResNet34 network is utilized to combine the RGB image information with the saliency map in order to determine the fruit ripeness level. As a result, the detection accuracy is better; however, this method does not recognize and detect the fruits in the case of branch and leaf occlusion, presenting an important limitation. In addition, Pan et al (Pan and Ahamed, 2022). applied the Mask RCNN model combined with a three-dimensional (3D) stereoscopic camera to detect balsam pears in complex orchard environments. Consequently, the accuracy of the target detection in the validation set and the test set was relatively high; yet, in the case of picking scenarios, considering obstructed, overlapped, and unevenly illuminated fruits, the detection accuracy decreased sharply, making it difficult to meet the requirements of automated operation.

Therefore, to address the issue regarding fruits being obscured, overlapped, and unevenly illuminated, as well as the adherence to complex environments, this study used persimmons as the detection target. Then, we proposed a target detection method based on the improved Mask RCNN model. This technique consists of integrating cropping, morphological processing, and concave-point segmentation modules in the fully connected layer of the Mask RCNN network structure to reach the target detection in scenarios of fruits being, or not, obstructed by branches and leaves, fruit overlapping, and other scenarios of persimmon accurate detection. As a result, the proposed method targets provide theoretical and technical support for the development of intelligent picking robot fruit target detection technology.

## 2 Design of the recognition algorithm

### 2.1 Color space model

**Figure 1:**
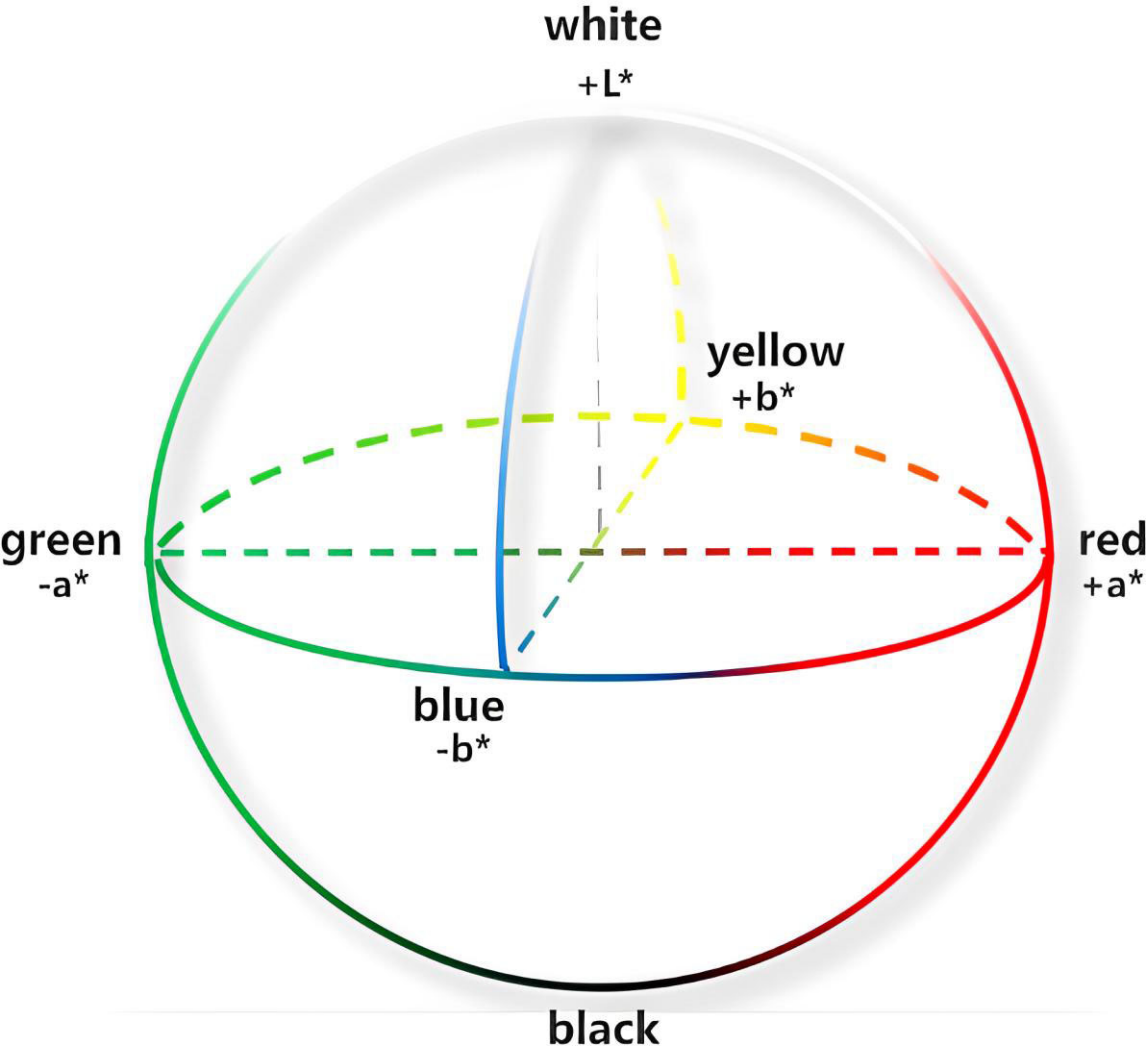
L*a*b* color space model.

**Figure 2:**
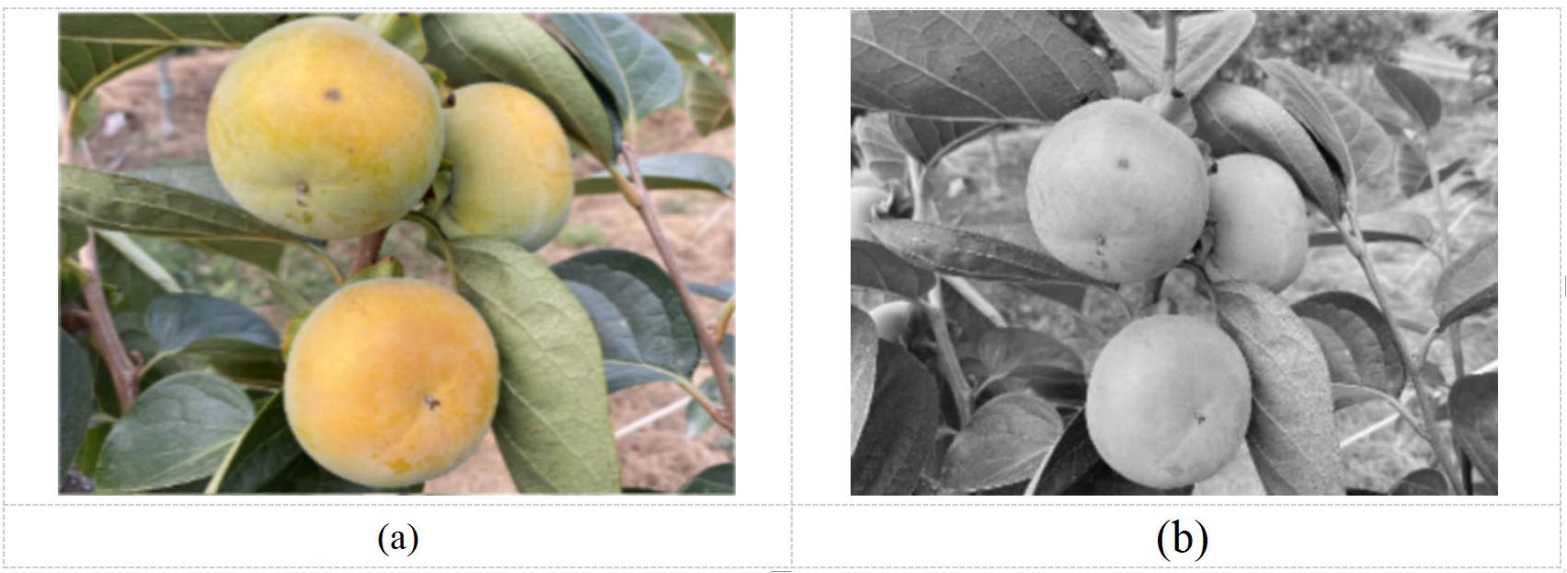
Median filter processing diagram. **(a)** Original, **(b)** median filter plot.

**Figure 3:**
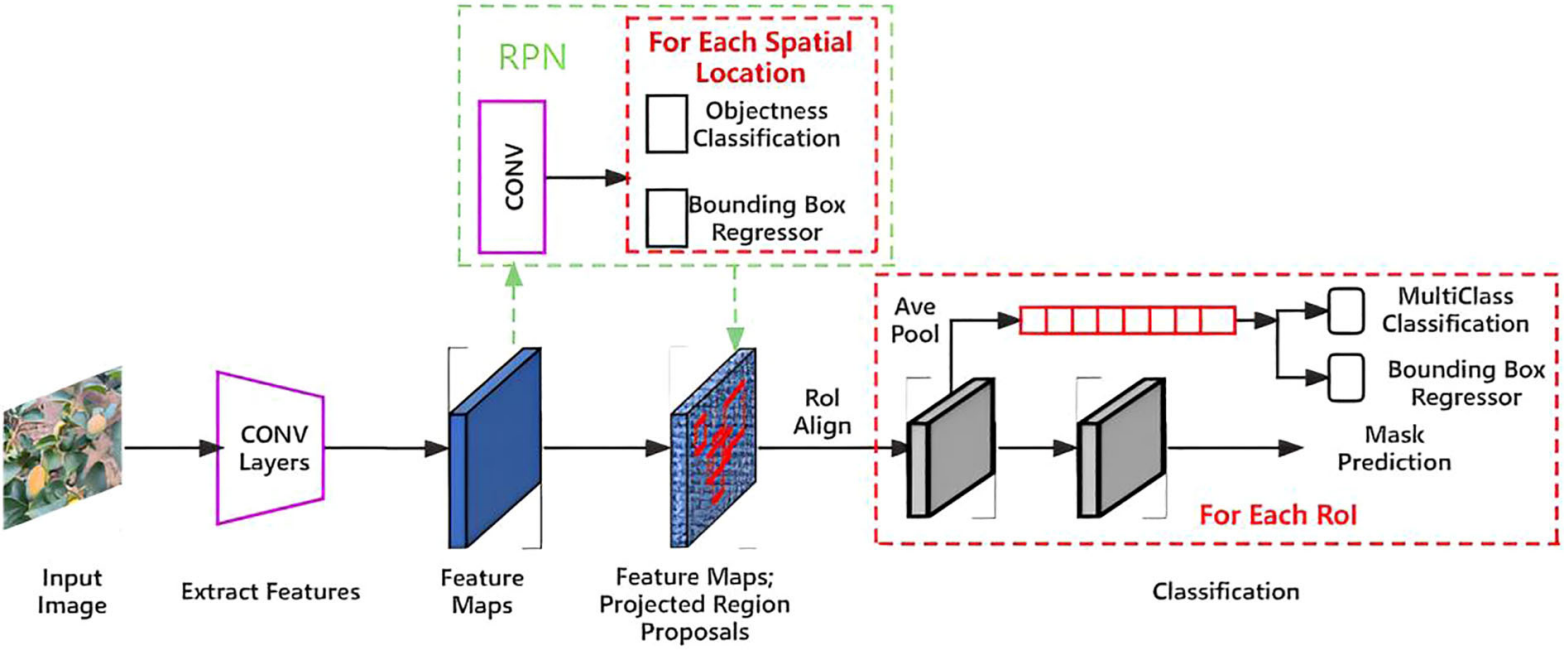
Mask RCNN network process.

**Figure 4:**
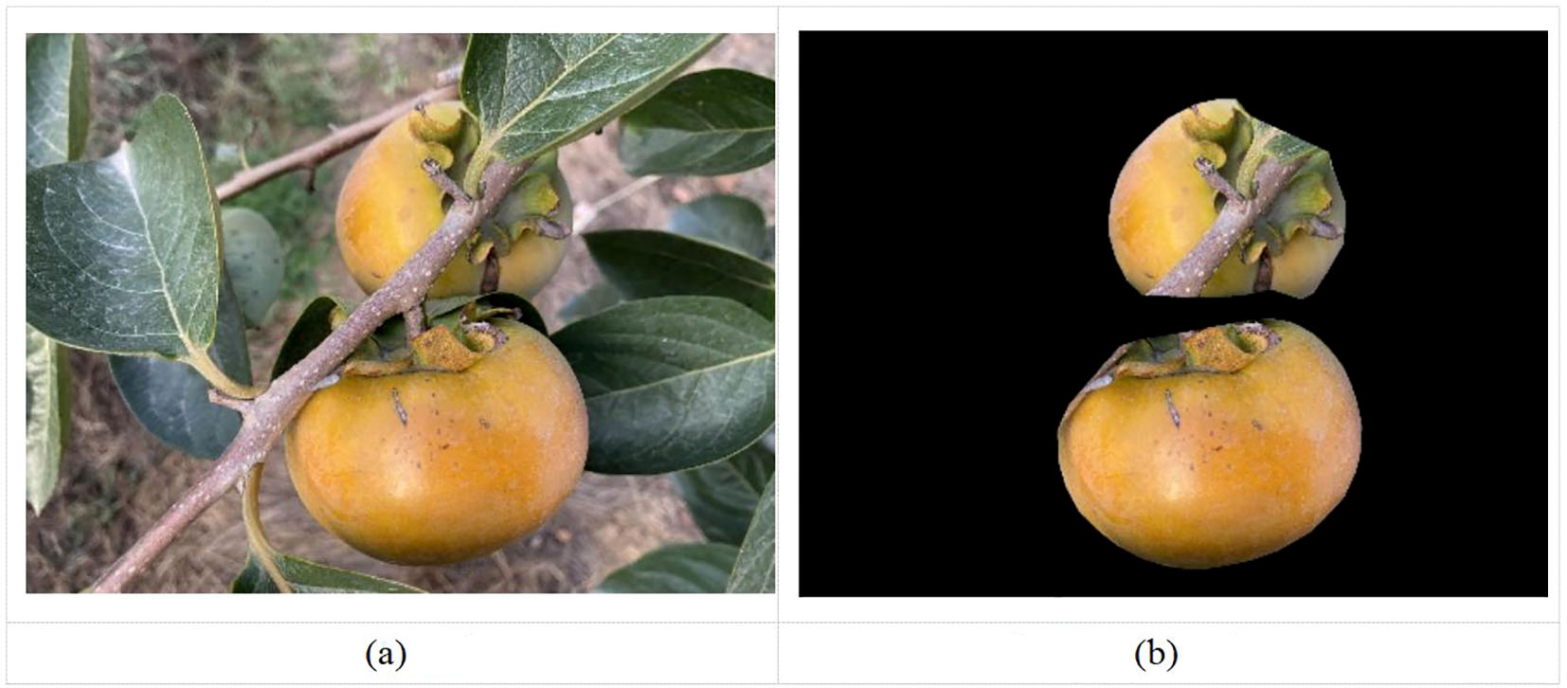
Visualization effect of target detection and mask segmentation. **(a)** Original, **(b)** Visualization of mask segmentation.

The color feature is one of the most intuitive global features describing the target object (Liu et al., 2023b). It is frequently applied in orchard robots, where the color space model is a representation of different color component scales through coordinate axis parameters. Since the color change of the full life cycle of persimmon ranges from green to red, whereas the color change of leaves is from green to yellow, this study adopted the (L*a*b*color) space model based on comparisons and analysis of a variety of color space models. This representation is the most expressive recognition of the persimmon’s full life cycle color features. The L*a*b* color space model provides a comprehensive, reliable, 3D color space with significant scalability to meet a variety of different color needs without being affected by any external environment and surpassing the traditional visual recognition methods. Therefore, this model achieves more accurate color recognition (Huang et al., 2023), as shown in **Figure 1**. Furthermore, the model stereogram was displayed in **Figures 2**–**4**, where the L* channel represents different degrees of light intensity, the value range is (0, 100), and the boundary of color ranges between black and white; the a* color changes between red and green; the b* color changes between yellow and blue, where the a* and b* value ranges are between −128 and 127.

Regarding the study of fruit recognition and classification, the purpose of the color space transformation is to determine the appropriate color component and construct an effective color operator (Yu et al., 2023). The three-component gray scale example map of persimmon samples in L*a*b*space was shown in **Table 1**. The spatial transformation relationship between L*a*b*color and RGB color is nonlinear, whereas the XYZ color space is deployed as a bridge to achieve the spatial transformation between both. The conversion relationship between the three spaces is defined as follows:

**Table 1:** Three-primary color component plot of persimmon samples in L*a*b* space.

L*a*b*mold	L*color component	a*color component	b*color component
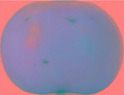	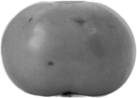	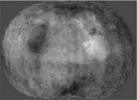	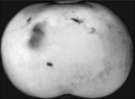

Changing the RGB color space to an XYZ color space yields in the following (**Equation 1**):


(1) $$\left[\begin{array}{c} X\\ Y\\ Z \end{array}]=\frac{1}{0.17697}[\begin{array}{ccc} 0.49 & 0.31 & 0.20\\ 0.17697 & 0.8124 & 0.01063\\ 0.00 & 0.01 & 0.99 \end{array}][\begin{array}{c} R\\ G\\ B \end{array}\right]$$

The XYZ color space is further varied into the L*a*b*color space where it is expressed as follows (**Equation 2**):


(2) $$\left\{ \begin{array}{l} L=116f(Y)-16\\ a=500[f(\frac{X}{0.982})-f(Y)]\\ b=200[f(Y)-f(\frac{Z}{1.192})] \end{array}\right.$$

The formula shown in **Equation 3**:


(3) $$f(t)=\{\begin{array}{ll} t^{\frac{\;}{13}} & t>0.008856\\ 7.787t+0.138 & t\leq 0.008856 \end{array}$$

### 2.2 Design of persimmon identification method

Shooting in different weather and angles may cause shadows and noise in the image, therefore impacting the image recognition of the fruit. In this study, the median filter processing was applied to complete the denoising process, and the median filter processed image was depicted in **Figure 2b**.

After denoising the image through morphology, concave point segmentation, and filtering, the persimmon fruits are recognized by template matching algorithm. This algorithm is processed by spatially aligning the sensor to the acquired image under different conditions. Therefore, the template is a known small image and template matching consists of searching for a target image among the known small images, where both target and template have the same size and orientation, and the image is processed using a specific algorithm to find its target and determine its coordinate position (Jiang et al., 2023).

Moreover, the algorithm is designed in the following way: the search template *T* is superimposed on the searched map *S* (W*H pixels) to translate, and the area where the template covers the searched map is called the sub-map *S_ij_*, where *i* and *j* represent the coordinates of the top left corner of the sub-map on the searched map S. The search range was shown in Equations 4, 5 as follows:


(4) 1≤i≤W−m

(5) 1≤j≤H-n

The process of template matching is achieved by comparing the similarity of *T* and *S_ij_*. To measure the degree of matching between both entities, the following two measures can be applied, shown in **Equations 6**, **7**:


(6) $$D(i,j)={\sum_{m=1}^{M}\sum_{n=1}^{N}\left[S_{ij}(m,n)-T(m,n)\right]}^{2}$$

(7) $$D(i,j)=\sum_{m=1}^{M}\sum_{n=1}^{N}\left|S_{ij}(m,n)-T(m,n)\right|$$

## 3 Improvement of instance segmentation algorithm for mask RCNN

### 3.1 Mask RCNN algorithm improvement method

Mask RCNN modifies the network structure of Faster RCNN by adjusting two aspects of Region of Interest (RoI) Pooling and adding target mask branches to realize target pixel-level segmentation (Lv et al., 2023). Initially, the quantization operation of RoI Pooling is replaced with RoI Align of the linear interpolation algorithm to achieve accurate point-to-point alignment in the feature mapping process while maintaining the precise spatial location. Consequently, the proposed architecture integrates the predicted target mask branch in parallel to the original basis. Through this approach, binary mask images of all persimmon targets in the image are generated using Fully Convolutional Networks (FCNs) with inverse convolution (Zhang et al., 2023). Finally, segmentation masks and segmented persimmon images of varying ripeness levels are predicted and obtained in a pixel-to-pixel manner. This simultaneous execution of target detection and segmentation occurs across parallel branches, with separate branches handling classification and target detection frame regression concurrently.

The Mask RCNN algorithm empowers the final model to perform not only target detection and classification but also instance segmentation. This capability arises from its ability to accurately preserve the spatial location of pixels and achieve pixel-by-pixel mask prediction. The total loss function in Mask RCNN comprises three main components: the classification loss of candidate frames, location regression loss, and target mask loss, defined as shown in Equations 8–**12**


(8) $$Loss=L_{cls}+L_{bos}+L_{mask}$$

where $L_{cls}$ represents the classification loss function, $L_{box}$ denotes the location regression loss function, and $L_{mask}$ represents the target mask loss function.


(9) $$L_{cls}=\frac{1}{N_{cls}}\sum_{i}-\log[p_{i}^{*}p_{i}+(1-p_{i}^{*})(1-p_{i})]$$

where $N_{cls}$ represents the normalization parameters, $p_{i}$ indicates the probability that the RoI with serial number is predicted to be a positive sample. Moreover, if $p_{i}^{*}=1$ , the suggested regions are positive samples, whereas, when $p_{i}^{*}=0$ , the suggested regions are negative samples.


(10) $$L_{box}=\frac{1}{N_{box}}\sum ip_{i}^{*}R(t_{i},t_{i}^{v})$$

(11) $$Smooth_{L}=\{\begin{array}{ll} 0.5X^{2} & if|X|<1\\ |X|-0.5 & otherwise \end{array}\}$$

where $N_{box}$ denotes the normalized parameters, $t_{i}$ represents the offset of the prediction box, $t_{i}^{v}\;$ highlights the parameters for the actual offset, and R— indicates the loss value.


(12) $$L_{mask}=-\frac{1}{m^{2}}[\sum y_{v}\log y_{v}^{k}+(1-y_{v})\log(1-y_{v}^{k})]$$

where $y_{v}$ represents the target true label value, $y_{v}^{k}$ indicates the predicted values, and show the number of target categories for the instance segmentation task.

The network flow of Mask RCNN was illustrated in **Figure 3**, and its algorithm process steps are the following: first, the image pre-processed picture is inputted to the Resnet50 feature extraction network pre-trained by mitigating learning to obtain the feature mapping map. Then, the predetermined RoIs are set in the feature map to generate multiple ROIs. Consequently, the first before and after scene classification are carried out in the Region Proposal Network (RPN) to detect if there is a target background in the prediction frames, and perform some correction on the prediction frames, such as filtering out part of the useless prediction frames. Finally, the resulting candidate frames are subjected to RoI Align operation, suggesting an output layer of prediction class labels, bounding boxes, and target masks for the region.

### 3.2 Example segmentation algorithm improvement method

Instance segmentation serves as a combination of two methods, semantic segmentation and target detection (Yi and Wang, 2023; Wang et al., 2024). The former consists of dividing the image or video, according to category similarities and differences, into multiple blocks, which are then transformed into machine language, realizing the classification of the image at the pixel level. As for the latter, it consists of distinguishing all target objects of interest in an image and determining their categories and locations, as various types of objects have different appearances, shapes, and postures, as well as distinct interference factors such as illumination and occlusion during imaging. This results in target detection difficulties and yields several problems in classification, localization, detection, and segmentation.

The requirement of instance segmentation consists of performing target detection based on semantic segmentation requirements, that is, to distinguish each instance target based on predicting the target contour for each pixel category (Ye et al., 2023). Therefore, this study serves to recognize fruit diameter detection of persimmons in a complex orchard environment, requiring high recognition and segmentation accuracy. Therefore, this study utilizes the expandability of the Mask RCNN algorithm to accurately achieve persimmon fruit diameter detection in complex orchard environments by instance segmentation based on target detection. Finally, the Mask RCNN algorithm’s target detection of persimmons and mask segmentation visualization results were illustrated in **Figure 4**.

The visualization effect of mask segmentation can be found in the picture. While the mask RCNN instance segmentation algorithm for persimmon fruit generally outlines the location accurately, it exhibits less precision at the edges of the fruit segmentation. Additionally, some occlusion objects within the fruit edge segmentation obscure accurate reflection and location of the persimmon fruit’s edge information. Further optimization of the segmentation process is necessary to enhance the accuracy of edge detection and localization.

### 3.3 Design of fruit instance segmentation network architecture based on improved Mask RCNN

The mask in the Mask RCNN algorithm relies on the persimmon recognition detection module, where the mask branch is acquired through a cropping process based on the prediction frame and specific thresholds. However, the masks obtained for fruit instances using this method exhibit limited differentiation ability, particularly at the boundaries of objects of the same species. This becomes more pronounced when fruits occlude each other, leading to competition among edge pixels.

Furthermore, the fruit target detection frame generated by the RoI feature in the fully connected layer may include images of objects other than fruits. Thus, another cropping process is applied to extract individual fruit target images. However, when fruits are partially occluded by branches or leaves, or when they overlap with each other, thresholding the pixels at the boundary of the fruits during cropping can result in issues such as sticking to occluded objects or mixing with background features, thereby affecting the final segmentation results.

**Figure 5:**
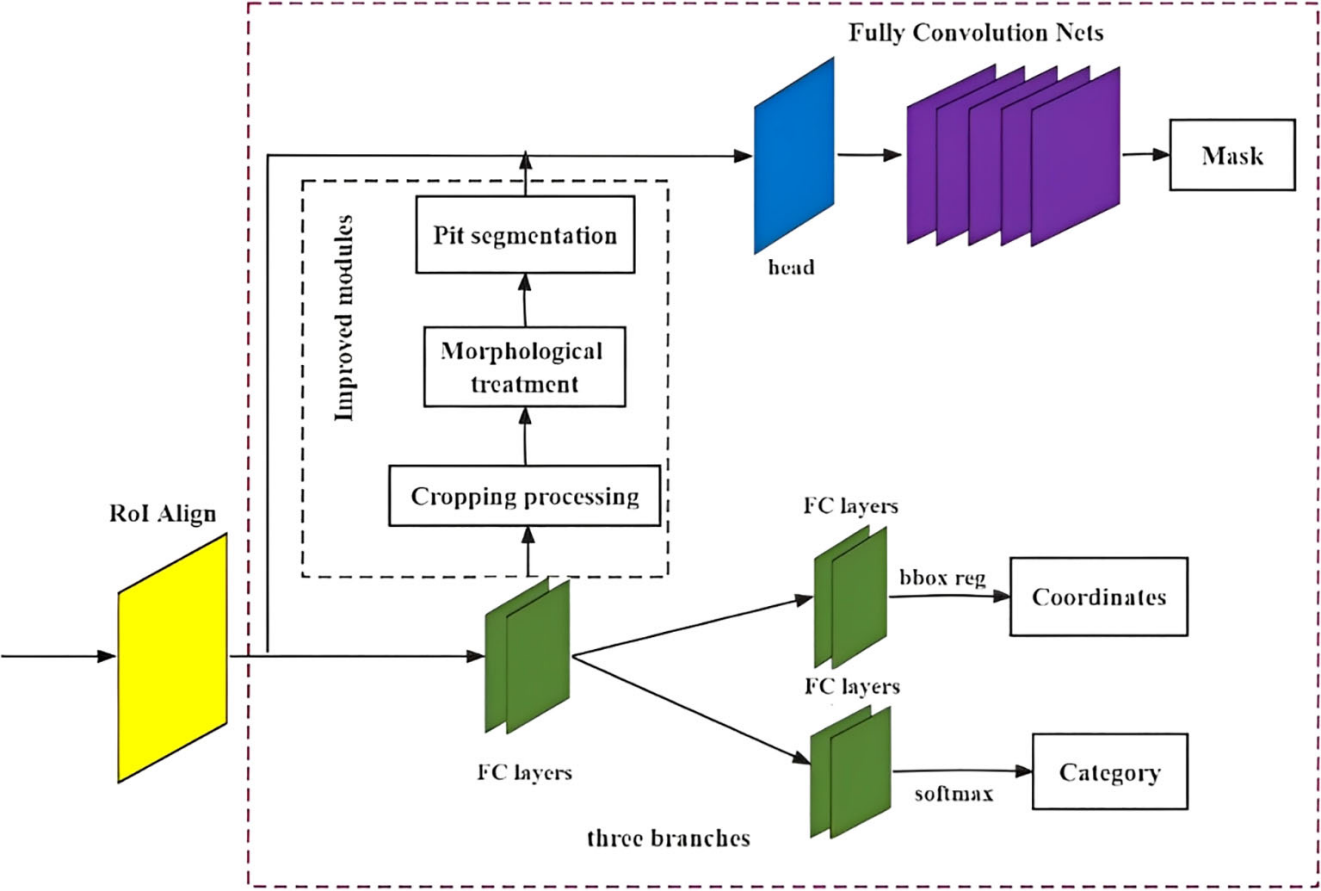
Improved Mask RCNN instance segmentation network structure.

To mitigate the effects of occlusion, overlap, and adhesion, morphological processing and concave point segmentation operations are employed to obtain the accurate contour of the target fruit. The improved Mask RCNN instance segmentation network structure was illustrated in **Figure 5**.

Morphological processing is a fundamental technique used for manipulating the shape features of an image to achieve specific objectives. It primarily involves operations such as expansion, erosion, and adhesion analysis. In more detail, the expansion operation consists of reading each pixel in the image one by one using a defined rectangular template and modify the value of the pixel to the maximum value to connect the salient points on the periphery of the image and extend them outwards. As for the expansion operation, it involves the process of finding the local maximum value by assigning the maximum value pixel point as a reference to the surrounding pixels to achieve the purpose of expanding the highlighted features in the image. The expanded image has a larger target area compared to the original image. Finally, the erosion operation has an opposite effect to expansion operation, that is, the process of obtaining the local minimum value. The assignment of the minimum value to the surrounding pixel points yielding in making the highlighted part of the image shrink; as a result, the target area shrinkage is reflected in the visual effect, and the location and area size of the fruit adhesion are generated by extracting the adhesion component. This method consists of using a rectangle as a template, reading all the pixels covered by the template one by one, modifying the value of pixel X to the smallest value among all the pixels, and corroding the highlights in the periphery of the image.

After performing the fruit example cropping, there are still some adhesions between the fruit surrounding and branches, leaves, and other fruits. Consequently, the cropped image is converted into a binarized image to determine the location and area of the adhesion image (Zhou et al., 2022); then, small ranges of adhesion areas are directly deleted.

**Figure 6:**
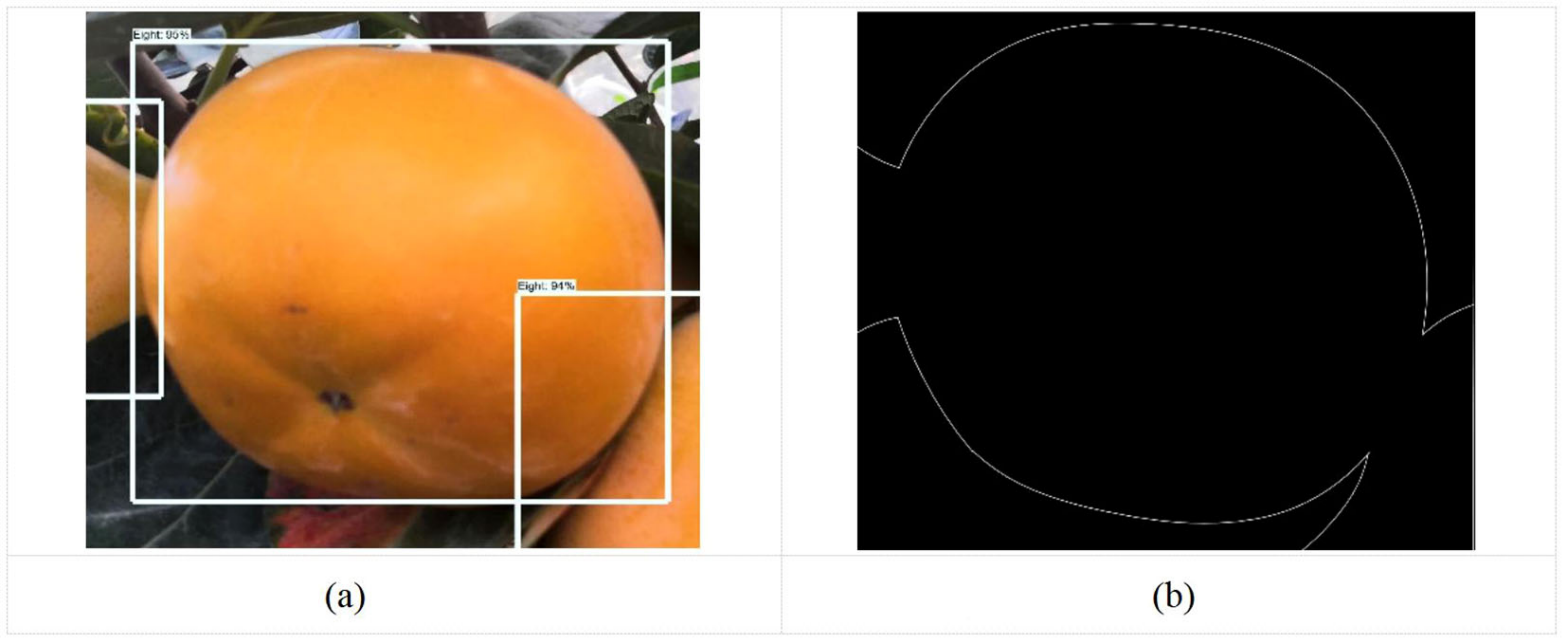
Processed pictures. **(a)** The target cropped picture, **(b)** Morphological treatment.

The target and background separation was basically solved by morphological processing; however, there were many problems of mutual occlusion and adhesion of persimmon fruits in natural environment, as shown in **Figure 6a**. The image after the morphological processing was illustrated in **Figure 6b**. Therefore, in the segmentation process of fruit instances, the problem of adhesion caused by mutual occlusion between fruits at their edge still resides. To tackle it, a solution consists of using concave point-based segmentation to find the concave points at the location of adhesion of fruits. Once found, these concave points will be connected so as to complete a more detailed segmentation of the target fruits.

**Figure 7:**
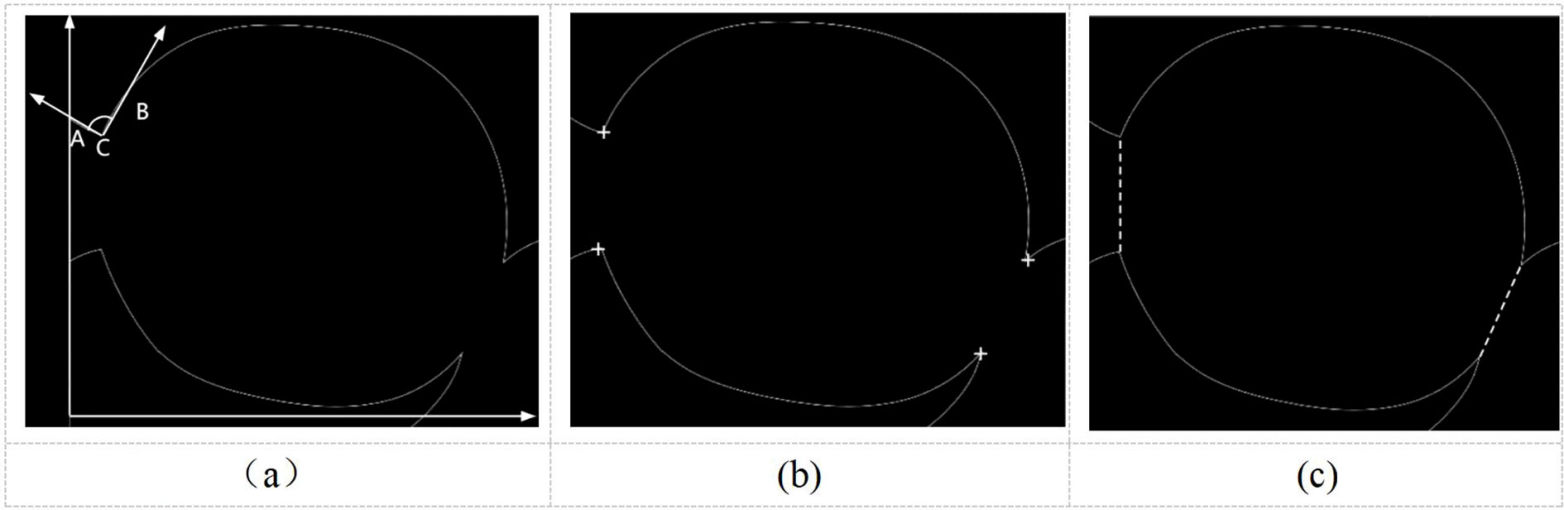
Diagram of the concave point segmentation process. **(a)** Vectorial pinch angle, **(b)** Concave point extraction, **(c)** Dividing line.

As the first step of concave point segmentation consists of finding the exact location of the concave point, this study adopts the vector pinch method for point extraction. This method considers each pixel point on the edge of the target object as a corner point, connected to front loci and back loci. These loci are also on the edge of the target object and at the same distance from the corner point; moreover, they are set pre- and post-determination of the corner point, as shown in **Figure 7**. The angle between the two straight lines formed by the front locus and the corner point and the back locus and the corner point, denoted as, and expressed in **Equations 13**–16 for concave point extraction.

An excessively high threshold may misidentify minor contour fluctuations as concave points, while an excessively low threshold may fail to detect shallow adhesion regions. In local coordinate systems, concave points exhibit negative curvature, typically corresponding to negative angular ranges (−50° to 0°), whereas convex points correspond to positive angles (0° to 50°). Therefore, this paper selecting [−50°, 50°] effectively covers typical adhesion-induced concavities, and it was shown in **Figure 7a**.


(13) $$|AC|=\sqrt{{\left(a_{1}-c_{1}\right)}^{2}+{\left(a_{2}-c_{2}\right)}^{2}}$$

(14) $$|CB|=\sqrt{{\left(b_{1}-c_{1}\right)}^{2}+{\left(b_{2}-c_{2}\right)}^{2}}$$

(15) $$|AB|=\sqrt{{\left(a_{1}-b_{1}\right)}^{2}+{\left(a_{2}-b_{2}\right)}^{2}}$$

(16) $$\theta =\frac{a\cos(|AC{|}^{2}+|CB{|}^{2}+|AB{|}^{2})}{2|AC||CB|}\times 180$$

The concave points are determined by the concave point segmentation formula as shown in **Figure 7b**. Consequently, the concave points are matched and connected to form the segmentation line. During the segmentation process, the pairs of concave points that conform to the directional characteristics of the circular growth of the fruit and have the smallest Euclidean distance between two pairs of concave points are matched and connected.

This study employs the Douglas-Peucker algorithm for polygonal contour approximation while preserving principal geometric features, implementing a comprehensive processing pipeline that includes (1) noise reduction through local curvature filtering, where the curvature (angle change per arc length) at each point is calculated to eliminate outliers with abrupt variations; (2) false segmentation prevention via dual geometric constraints on concave points, enforcing inter-point spacing (Rmin ≤ d < 2Rmax) and recession depth (h > 0.2R) to exclude shallow depressions caused by leaf occlusion; and (3) topological consistency validation requiring segmented sub-contours to satisfy both area thresholds (A ∈ [0.7Aavg, 1.34Aavg], where Aavg represents the average single-fruit area) and circularity criteria (4πA/P² > 0.7, with P denoting perimeter, effectively filtering non-fruit fragments). Therefore, the segmentation line is finally determined, as displayed in **Figure 7c**.

## 4 Fruit diameter detection method design

### 4.1 Fruit edge information extraction

After getting a finer image segmentation after performing morphological processing, the fruit edge coordinate information is extracted. Moreover, the mask pixel point coordinates, obtained after segmentation, are first saved in the extraction process, followed by the edge coordinates. In the Mask RCNN algorithm, the pixel coordinates in the output image of the mask are “True” when the mask image yields the target object and “False” when the mask image is the background image. Considering the upper left corner of the image as the origin to establish the (*X*, *Y*) axis coordinate system, take the *X* axis as the base. By moving the straight line *y* = *i* parallel to the *X* axis, this line is traversed from left to right. After achieving the retrieval, the line is panned from top to bottom, as highlighted in **Figure 7a**. According to the above method, all pixel coordinates in the retrieved image are gradually traversed, and, finally, all pixel coordinates of the target area in the mask are set as “True.”

**Figure 8:**
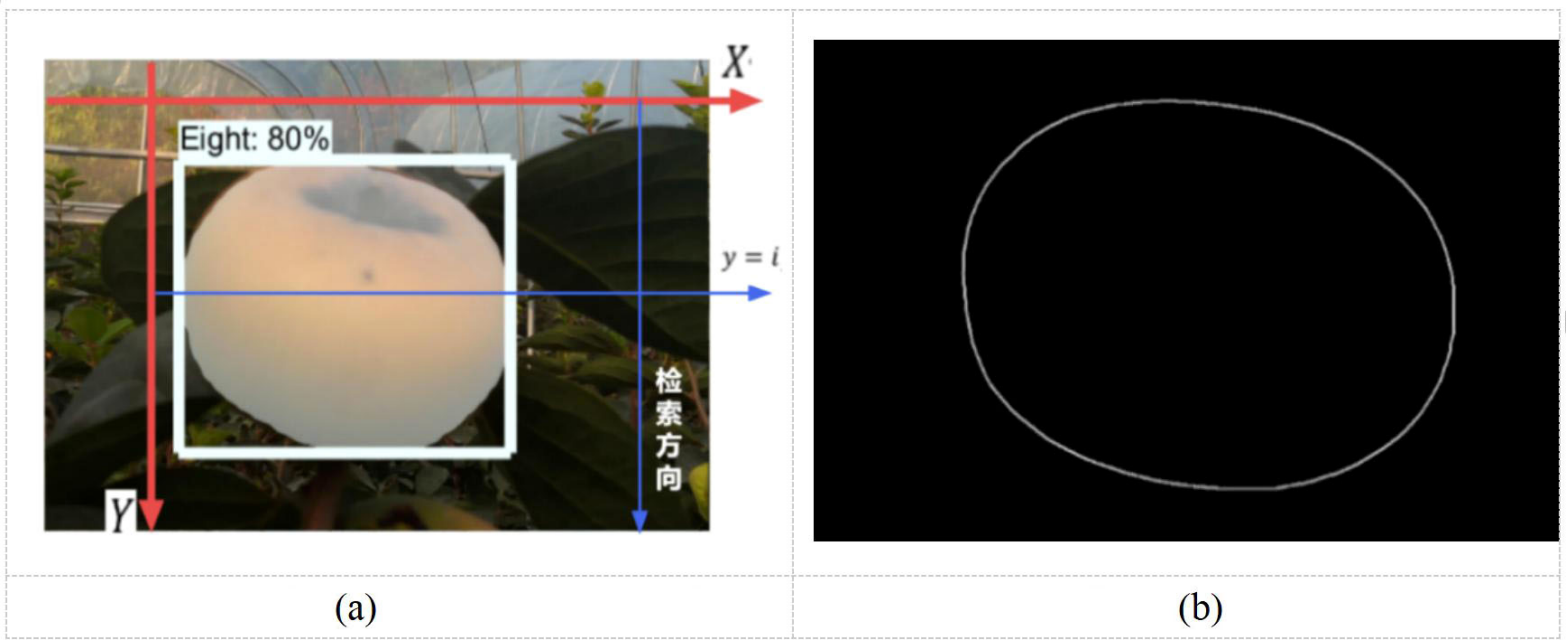
Edge information processing diagram. **(a)** How mask coordinates are retrieved, **(b)** Fruit edge extraction.

Following the implementation of this method, only the fruit mask edge coordinate information is preserved in order to detect the size of the fruit. While saving the coordinates of all pixels, the information in the image is also stored from left to right by traversing the line parallel to the *X* axis. Consequently, for each line parallel to the *X* axis, the first and the last pixel coordinates “True” correspond to both edges of the fruit, and the cycle of this method acquires all pixel coordinates of the fruit edges. The visualization results were shown in **Figures 8a, b**.

### 4.2 Fruit diameter detection method

Before measuring the fruit diameter, the coordinates of the center of the persimmon fruit image are generated, where the maximum connection length through the center of the shape represents the width of the persimmon fruit and the shortest connection length through the center of the shape indicates the thickness of the fruit. Based on this definition, the calculation begins by traversing the pixel points along the edge of the fruit. Then, the distance between each pair of consecutive coordinates is computed using the following expression, shown in **Equation 17**:


(17) $$d=\sqrt{{\left(y_{2}-y_{1}\right)}^{2}+{\left(x_{2}-x_{1}\right)}^{2}}$$

The process continues by iteratively calculating the distance equation between each pair of consecutive coordinates. During the iteration, the algorithm prioritizes the coordinates with the longest distance between them. At the end of the cycle, the fruit’s edge is determined by the line connecting the two points of the longest connection with the shortest connection serving as the fruit diameter width and thickness. It is important to mention that the four Pixel coordinate points $P_{l1}(x_{l1},y_{l1}),\;P_{l2}(x_{l2},y_{l2}),\;P_{s1}(x_{s2},y_{s2})$ and $P_{s2}(x_{s2},y_{s2})$ were saved for further analysis.

The persimmon fruit diameter size measurements are obtained by applying this calculation. In this study, the camera was calibrated using the Zhang Zhengyou calibration method, converting the acquired pixel point coordinates into real 3D spatial points. The conversion relation for obtaining the spatial point $P(X_{w},Y_{w},Z_{w})$ with pixel coordinate point $P_{l1}(x_{l1},y_{l1})$ was represented in Equation 18.


(18) $$Z_{c}[\begin{array}{l} x\\ y\\ 1 \end{array}]=[\begin{array}{cccc} \alpha & 0 & \mu_{0} & 0\\ 0 & \beta & \nu_{0} & 0\\ 0 & 0 & 1 & 0 \end{array}][\begin{array}{cc} R & T\\ 0^{T} & 1 \end{array}][\begin{array}{l} X_{w}\\ Y_{w}\\ Z_{w} \end{array}]$$

Where $Z_{c}$ represents the vertical axis of the camera’s spatial coordinate system, demote the external parameters of the camera, and $a,\;\;\beta ,\;\;\mu_{0},\;\;\upsilon_{0}$ indicate the internal parameters of the camera.

According to the Zhang Zhengyou calibration method, to achieve the camera left and right sides of the respective single target calibration, the conversion relationship is represented as follows (**Equation 19**):


(19) $$[\begin{array}{l} x_{c_{0}}\\ y_{c_{0}}\\ z_{c_{0}} \end{array}]=R_{z}R_{y}^{-1}[\begin{array}{l} x_{c_{1}}\\ y_{c_{1}}\\ z_{c} \end{array}]+T_{z}-R_{z}R_{y}^{-1}T_{y}$$

Where $\left(x_{c_{0}},y_{c_{0}},z_{c_{0}}\right)$ represents the points on the imaging plane of the left camera, $\left(x_{c_{1}},y_{c_{1}},z_{c_{1}}\right)$ denote the point on the imaging plane of the right camera, $R_{z}$ and $T_{z}$ indicate the external parameters of the left camera, $R_{y}\;\;$ and $T_{y}$ highlight the right camera external parameters.

## 5 Test results and analysis

### 5.1 Purpose and methodology of the test

To test the effectiveness of the improved instance segmentation algorithm, this study initially analyzed and compared the effectiveness of the modules added to the Mask RCNN instance segmentation using the ablation test, as well as quantitatively analyzed the role of each module. Consequently, the comparison test of the fruit diameter measurement was performed to verify the efficiency of the improved segmentation and realize the automatic calculation and measurement of the program.

This study builds upon previous research (Liu et al., 2023a), utilizing a training set of 9,300 persimmon images at different growth stages. Additionally, 900 new persimmon images were collected from the National Persimmon Germplasm Repository in Hefei, Anhui Province, covering various growth phases. Through data augmentation techniques—including random cropping, random flipping, random contrast enhancement, and color variation—the newly acquired 900-image dataset was expanded to 3,000 images to serve as the test set.

Ablation test method: by means of module superposition, that is, the control variable method, the improved modules are combined in different arrangements, and the test was carried out sequentially. The test platform is set up as shown in Section 4.3, using MIoU, mean Average Precision (mAP) value, and Frames Per Second (FPS) as evaluation indexes, and applied to analyze and compare the instance segmentation impact of the improved algorithm.

The samples of the 200 were randomly divided into five groups, where the images of Group 1 were detected using the basic Mask RCNN algorithm, then Groups 2, 3, and 4 using three algorithms (e.g., the cropping processing module, the morphological processing module, and the concave point segmentation module, respectively), and Group 5 using the improved Mask RCNN algorithm. Each group of tests was repeated three times, and the Average Precision (AP) and mAP were recorded. Moreover, the average value of the three times was recorded as the valid value. The results of these three algorithms were compared to make sure that the proposed Mask RCNN algorithm can accurately recognize the target.

Fruit diameter measurement test method: 200 images were randomly selected in a natural environment without being covered by branches and leaves or other objects of the fruit as a test sample. Moreover, vernier calipers were used to manually detect the same this, to obtain the fruit diameter measurement width ${\text{ K}}_{\text{z}1}$ and thickness ${\text{ K}}_{\text{z}2}$ . According to the size, fruits were divided into 10 groups. In addition, a test through the split detection was performed to obtain the fruit diameter size width ${\text{K}}_{\text{c}1}$ and thickness ${\text{K}}_{\text{c}2}$ , and compared with the manual measurement value. Each group consisted of an average of 20 fruits recorded. Through analysis, the size of the error value was computed to test the accuracy of the improved algorithm in this study.

### 5.2 Data acquisition and test bench construction

**Figure 9:**
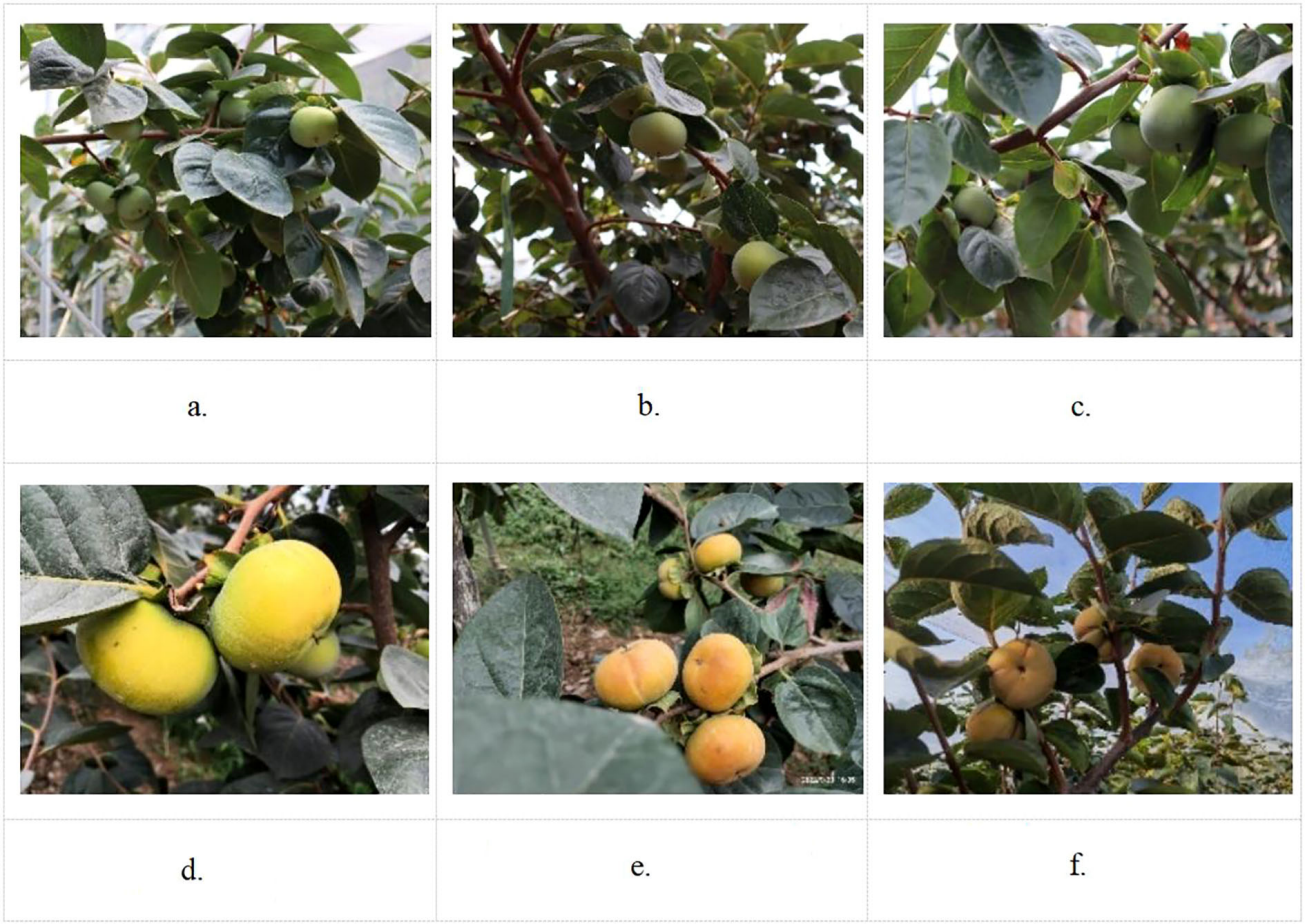
Example of a partial image sample of a persimmon fruit in nature. **(a)** Overlapping fruits shaded by foliage, **(b)** Shade, **(c)** Fruits are independent of each other, **(d)** Exposure, **(e)** Cloudy day and smooth light, **(f)** Backlighting on a sunny day.

To meet the diversity of persimmon literacy growth in complex environments, the collected images fully considered the variability of the sample data; that is, 3,300 persimmons in different growth periods were collected in variable environments such as using different light conditions, different angles, different numbers of fruits, branch leaf shade, and overlapping of multiple clusters of fruits, as shown in **Figure 9**.

The specific operating environment parameters of this study were displayed in **Table 2**. Moreover, two sets of self-constructed persimmon datasets were randomly assigned to the training and test sets using a 9:1 ratio.

**Table 2:** Runtime environment parameters.

Hardware	Configuration	Environment	Version
CPU	Intel Core i7-12700	Python	3.7.13
GPU	RTX 3060	PyTorch	1.7.1+cu110
RAM	64 G	CUDA	11.0
Hard-disk	520G	CUDNN	8.0.5

To avoid problems such as overfitting or underfitting caused by improper tuning of hyper-parameters for model training, the tuning of hyper-parameters was achieved by the network searching method to obtain optimal numerical points by giving a larger range of data and smaller searching step size. In this study, the number of iteration rounds (epoch) was set to 300, the learning rate (Ir) was set to 0.01, the optimizer adopts Stochastic Gradient Descent (SGD), and the momentum factor (momentum) parameter was set to 0.9. During the training period, the tensor-board was employed to record the loss and learning rate of the training set generated by each iteration. Data, including the training set loss and learning rate changes generated, and weights were kept and saved.

### 5.3 Evaluation indicators

**(1) *Precision (P***): for the model prediction results, it represents the number of positive cases in the prediction process, that is, the ratio of the number of correct objects detected to the total number of correct objects in the sample. Moreover, the response category prediction correctness is employed as a measure of the accuracy for the model detection, shown in **Equation 20**.


(20) $$\text{P}=\frac{TP}{TP+FP}\times 100\%$$

where TP denotes the genuine example and  FP represents the False Positive example.

**(2)*Recall (R)***: for all the samples in the dataset, it indicates the number many positive cases that are correctly predicted, that is, the ratio of the number of correct objects detected to the number of objects in the sample. It also measures the number of positive samples obtained by the model through the prediction process, shown in **Equation 21**.


(21) $$\text{R}=\frac{TP}{TP+FN}\times 100\%$$

Where FN represents the False Negative example.

**(3)*P-R curve***: a graph constituted by connecting the corresponding points of the horizontal and vertical coordinates indicated through the recall and the precision rates. Furthermore, a corresponding P-R curve is built for each category in the prediction process.

**(4)*Average-Precision (AP***): it represents the area of the *PR* curve plotted by *P* and *R*. The area under the curve denotes the average of all the accuracies across recall values. Moreover, it measures the accuracy of the model in given categories, shown in **Equation 22**.


(22) $$\text{AP}=\sum_{j=0}^{i-1}(R_{k}+R_{k+1})P_{k}$$

Where i in number of thresholds and j represents the category.

**(5)*Mean Average Precision (mAP)***: it represents the average value of AP in each category, measuring the accuracy value of the trained model in all categories. Moreover, it denotes the most important evaluation index in target detection algorithms, shown in **Equation 23**.


(23) $$mAP=\frac{1}{n}\sum_{j=1}^{n}AP_{i}$$

where *n* represents the total number of categories.

**(6)*Mean Intersection-over-Union (MIoU)***: it represents the most representative evaluation index for segmentation networks. It describes the overlapping degree between the candidate frames and the manually labeled frames generated by all categories during the model training process. Moreover, it calculates the average value of the ratio of the resulting intersection and concatenation to quantify the fitting degree between both frames and then to judge the quality of the model detection, shown in **Equation 24**.


(24) $$MIoU=\frac{1}{n+1}\sum_{l}^{i}\frac{TP}{FN+FP+TP}$$

Where 1 represents the true value.

**(7)*FPS***: it represents the number of images that can be processed per second by the network model, being an evaluation index to reflect the real-time performance of the model.

**(8)*Relative error***, shown in **Equations 25**, **26**:


(25) $$E_{r1}=\frac{\left|K_{c1}-K_{z1}\right|}{K_{z1}}\times 100\%$$

(26) $$E_{r2}=\frac{\left|K_{c2}-K_{z2}\right|}{K_{z2}}\times 100\%$$

where $K_{C1}\;$ represents the width of the fruit diameter, $K_{C2}\;$ denotes the thickness of the fruit diameter, $K_{Z1}\;$ indicates the actual fruit diameter width, and, finally, $K_{Z2}\;$ yields the Actual fruit thickness.

### 5.4 Ablation test results and analysis

**Figure 10:**
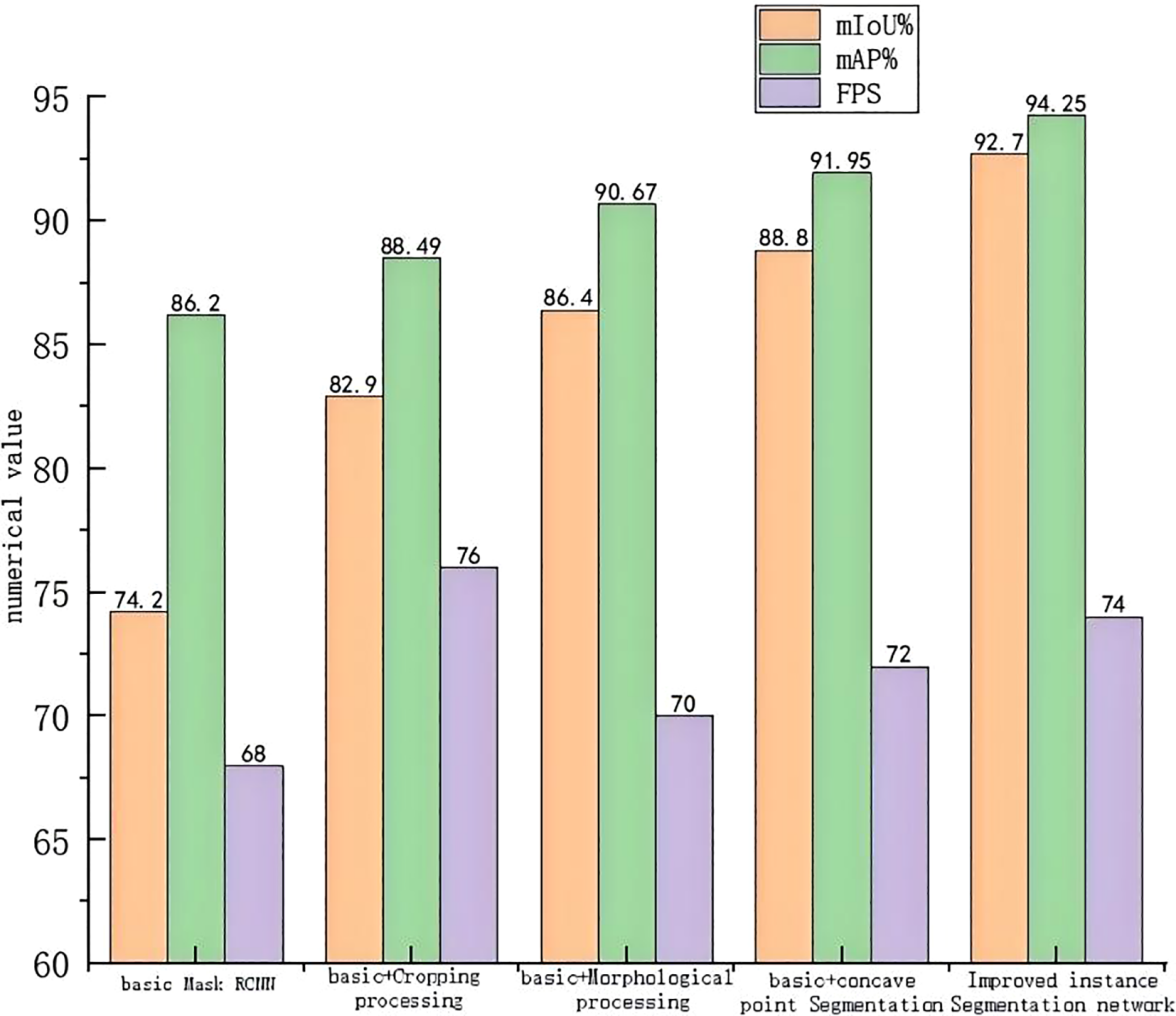
Ablation test data chart.

Referring to **Figure 10**, the MIoU values of the three network algorithms after the integration of the cropping processing module, morphological processing module, and concave point segmentation module to the base network Mask RCNN were set to 82.9%, 86.4%, and 88.8%, respectively; as for the mAP values, they were set to be 88.49%, 90.67%, and 91.95%, respectively. The three indexes of adding the cropping processing module compared to the basic network are improved by 11.73%, 2.66%, and 11.76%, respectively, proving that the addition of the cropping processing module can significantly enhance the performance of the network in terms of the mean intersection, parallel ratio, and processing speed. However, the enhancement of the average accuracy remains relatively small.

In addition, the three metrics of adding the morphological processing module compared to the base network are improved by 16.44%, 5.19%, and 2.94%, respectively, showing that adding the morphological processing module can significantly improve the performance of the network regarding the equalization and concatenation ratio. Moreover, the improvement of the average accuracy is relatively large, but the enhancement of the performance of the processing speed is relatively small. All three indexes of adding the concave point segmentation module compared to the base network are improved by 19.68%, 6.67%, and 5.88%, respectively, proving that the addition of the concave point segmentation module can significantly enhance the network’s equalization and concurrency ratio performance. Meanwhile, the effect of the average accuracy improvement and processing speed performance is more significant. It is verified that the enhanced method proposed in this study for the base network Mask RCNN is efficient and effective.

Referring to **Figure 10**, the improved algorithm in this study by simultaneously adding the three modules of cropping, morphological processing, and concave point segmentation has an MIoU value of 92.7%, an mAP value of 94.25%, and an FPS value of 74. Compared to the base network, the MIoU value has been increased by 24.93%, the mAP value has been improved by 9.34%, and the FPS value has been adjusted by 8.82%. This proves that the improvement method by adding the three modules to the base network Mask RCNN at the same time is effective. Compared to the base network, the performance of all aspects is improved, and the required accuracy of the fruit diameter measurement is achieved.

### 5.5 Fruit diameter measurement test results and analysis

(1) Comparative analysis of fruit diameter width

(2) Comparison of fruit diameter thickness analysis

**Figure 11:**
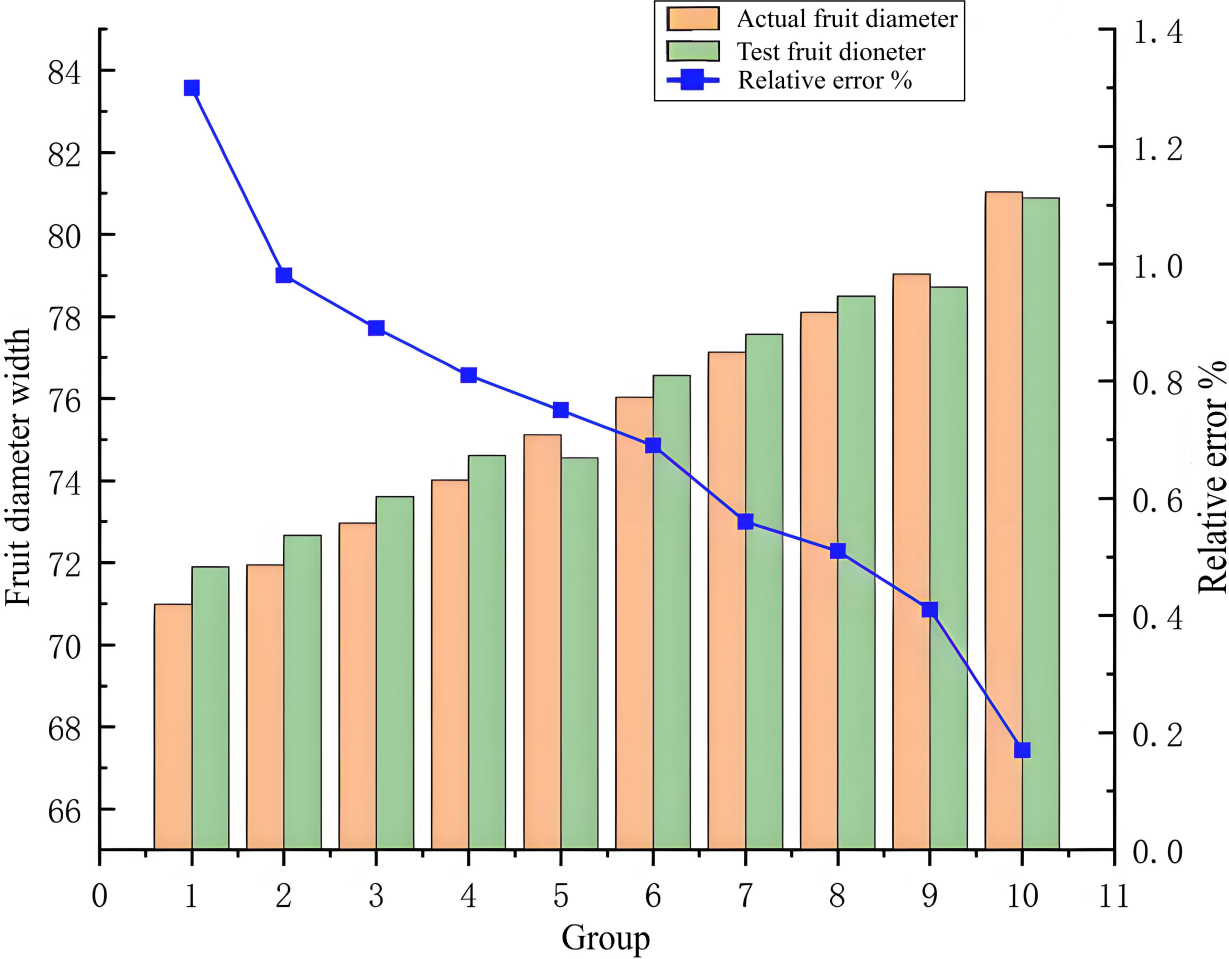
Comparison of data from some data and fruit width.

**Figure 12:**
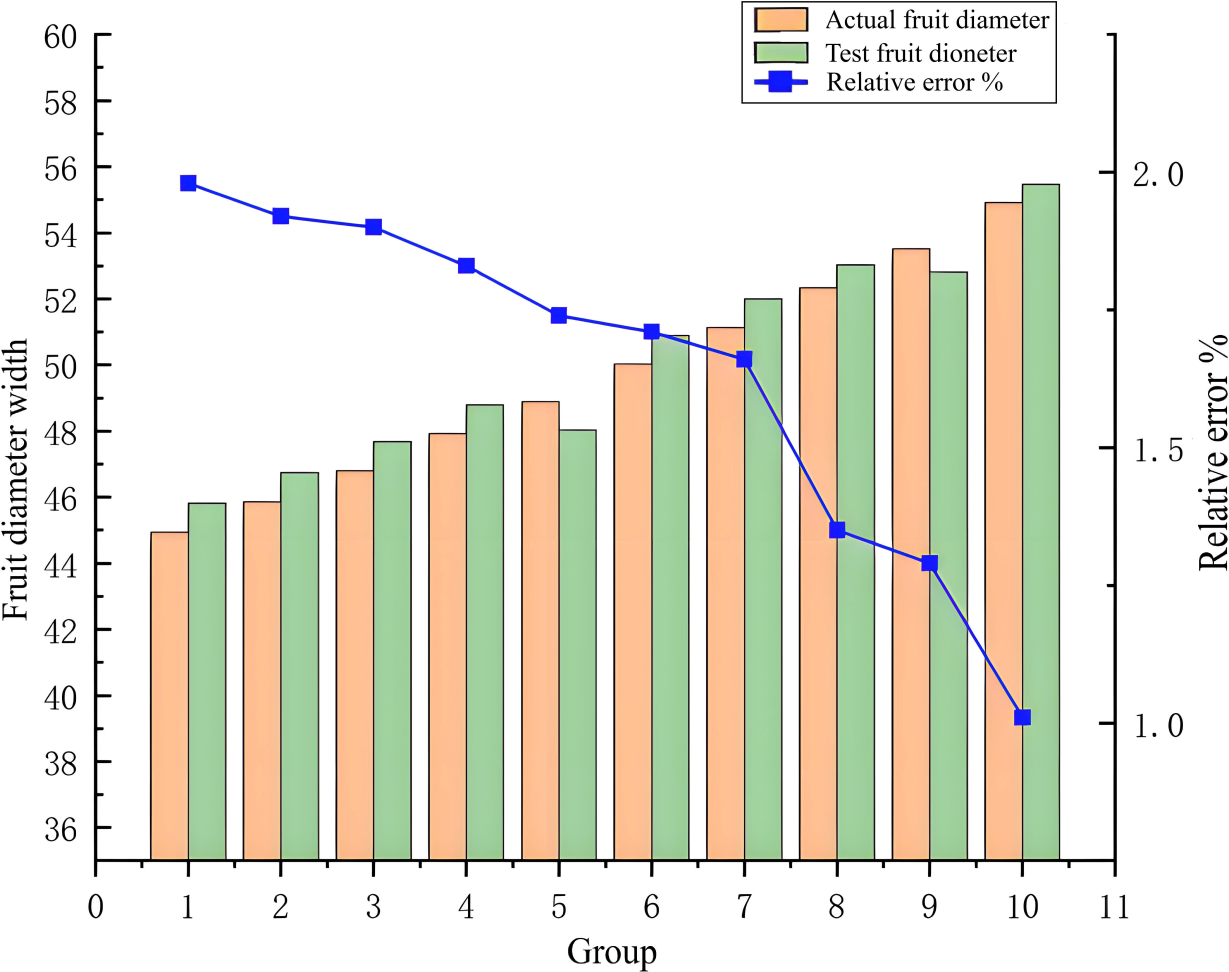
Comparison of some data and fruit thickness test data.

The analysis of the test error results, illustrated in **Figures 11**, **12**, shows that due to the autogenous growth characteristics of persimmon fruit, its edge is smooth and not easily deformed, yielding measurement difficulties. However, in the measurement of fruit width, the relative error varies between 0.17% and 1.3%, representing a small interval and proving that this study has a high accuracy in the measurement of fruit width. Concerning the thickness of persimmon fruit, it is difficult to measure it for fruit diameter due to the easy deformation of the fruit caused by the connection of the fruit stalk. However, while measuring it, the relative error varies between 1.01% and 1.98%, also representing a small interval, and proving, once again, this study had a high accuracy in the measurement of fruit thickness. While excluding the effect of deformed fruit and vernier caliper reading error, the relative error range in the overall measurement of fruit thickness and fruit width is within a reasonable acceptable range.

Based on the error curve, it is clear that, with the increase of persimmon fruit width or fruit thickness, the relative error is smaller. Due to the error index of this study that represents the relative error, the error decreases with the increase of fruit diameter. Therefore, it can be concluded that the measurement accuracy of fruit diameter is stable and unchanged, and the surface of the improved algorithm has a significant improvement in detection accuracy and precision performance.

### 5.6 Discussion

Target detection has been the most challenging problem in the field of computer vision due to the different appearances, shapes, and poses of outdoor persimmons. Moreover, several environmental conditions, including the interference of light and occlusion, were considered as disturbances during imaging. Therefore, the addition of the cropping processing module, the morphological processing module, and the concave point segmentation module, respectively, yielded three new detection algorithms and resulted in improved Mask RCNN instance segmentation algorithm detection.

Referring to **Figure 10**, the results of the ablation experiments indicate that, compared to the basic Mask RCNN instance segmentation algorithm, the improved Mask RCNN instance segmentation algorithm resulted in an enhancement of the MIoU value by 24.93%, the mAP value by 9.34%, and the FPS value by 8.82%. Therefore, the MIoU and mAP values are most significantly improved with the addition of clipping, morphological processing, and concave point segmentation modules, respectively; yet, the FPS metric improvement effect is missing and lower than the improvement rate when only the clipping module is added.

As the FPS index indicates the number of images processed per second by the network model, representing an evaluation index to reflect the real-time performance of the model is a must. In the fully connected layer of the basic Mask RCNN network structure, adding the cropping module will improve extracted image features and increase the model processing speed. However, when the clipping, morphological processing, and concave point segmentation modules are simultaneously integrated, the model becomes more replicated, therefore increasing the program running time, which in turn leads to a lower overall processing speed than when the clipping module is added alone.

Moreover, in this study, the vector clip angle method is applied for concave point extraction. This method consists of considering each pixel point on the edge of the target object as a corner point. Consequently, set two anterior and posterior loci, representing also the edge of the target object and at an equal distance from the corner point, before and after the determined corner point, and the anterior and posterior loci from two straight lines with the corner point, respectively, and the two straight lines form an angle, and then set the appropriate clip angle for the extraction of concave points applying Equation 16. When the clipping module and the concave point segmentation module are added simultaneously, the clipping module will crop out some pixel points, reducing the pixel points at the edges of the target object and slowing down the concave point extraction speed. Therefore, the addition of the three modules at the same time will reduce the execution speed compared to the use of the clipping module alone.

According to **Figures 11**, **12**, the relative error decreases with the increase of fruit diameter, and this decreasing trend of the relative error curve is approximately linear, showing that the improved Mask RCNN algorithm has stable measurement accuracy. Moreover, this study further segments the occluded objects, such as fruit stalks, by adding a morphological processing module and a concave point extraction processing method to improve the measurement accuracy. The enhanced Mask RCNN algorithm reduces the parameters for training, rendering the filter independent of the signal position; it also improves the characteristics of the detected signal and reinforces the generalization ability of the trained model. Finally, the proposed algorithm proves the correctness and effectiveness of the improved Mask RCNN instance segmentation method.

## 6 Conclusion

In this study, focusing on the persimmon inspection recognition and fruit diameter detection in natural environments, challenges, such as fruit occlusion and adhesion, were addressed. Through enhancements made to the Mask RCNN instance segmentation algorithm, the aim was to achieve high-precision, non-destructive detection of fruit diameter.

This study leverages the versatility of the Mask RCNN algorithm to achieve accurate fruit recognition in intricate environments through instance segmentation following target detection. Additionally, an enhanced instance segmentation algorithm for Mask RCNN is proposed to achieve accurate fruit detection. Building upon the RCNN network structure, the RoI Pooling quantization operation is substituted by RoI Align employing a linear interpolation algorithm, and a parallel predictive target mask branch is introduced to enable accurate fruit recognition. Moreover, in this study, a fruit diameter detection method in complex environments was added to tackle the problem of edge pixel competition when fruits mask each other. Finally, integrating cropping, morphological processing, and concave point segmentation modules to the fully connected layer of the Mask RCNN network structure results in a precise contour detection of the target fruits and the identification of the fruit diameter measurement.

The results of the ablation test highlight that the improved algorithm has significantly improved the mAP, MIoU, and FPS values compared to the traditional Mask RCNN instance segmentation algorithm, verifying the validity of this study to significantly improve the measurement accuracy of the improved Mask RCNN instance segmentation algorithm by adding these different modules. Moreover, the results of the fruit diameter measurement test verify that the proposed method can effectively tackle the challenges of adhesion in the fruit measurement by adding the cropping, morphology processing, and concave point segmentation module to the Mask RCNN instance segmentation improved algorithm. As a result, high segmentation accuracy would solve the fruit measurement challenges in sticky problems and can accurately detect the size of the fruit diameter.

Finally, it is worth noting that this study provides theoretical support and technical reference for orchard inspection, yield estimation, fruit detection, and mechanized picking. This study enables prediction of batch harvesting schedules and yield estimation for persimmons based on market demand, thereby increasing growers’ income.

## Data Availability

The original contributions presented in the study are included in the article/**Supplementary Material**. Further inquiries can be directed to the corresponding authors.
