# Supplementary figures and images for: Research on persimmon fruit diameter accurate detection method based on improved RCNN instance segmentation algorithm (part 2 of 2)
Source: Front Plant Sci. 2025 Aug 29;16:1636727. doi: 10.3389/fpls.2025.1636727 (PMC12426853; doi:10.3389/fpls.2025.1636727)

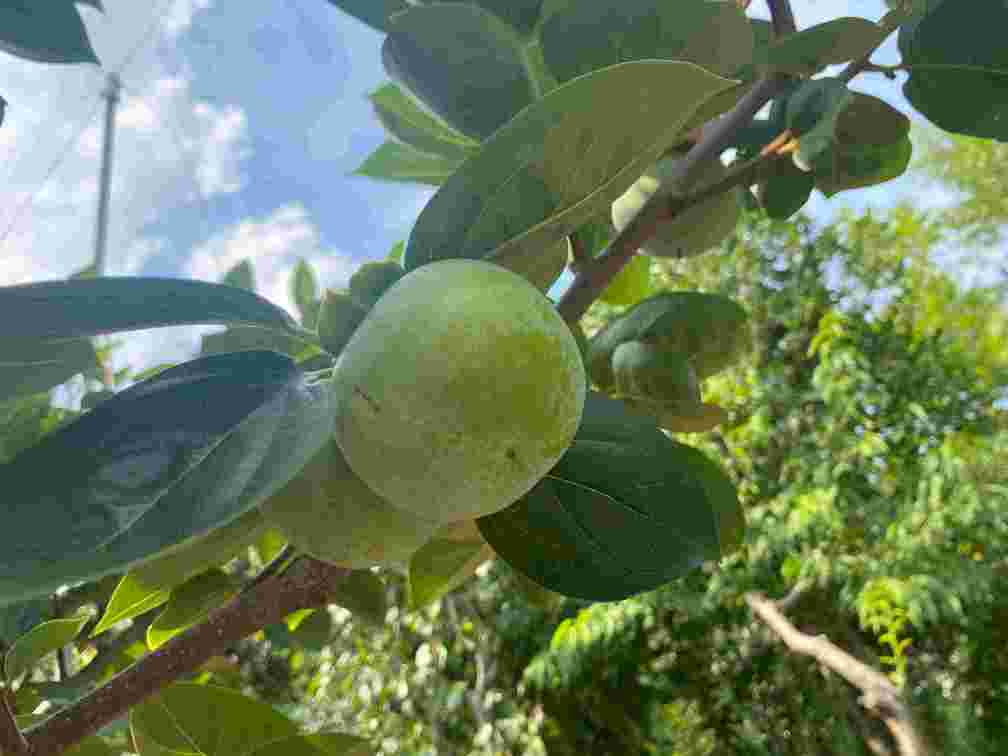

Supplement: Supplementary file 1 [file DataSheet1.zip › 2022-07-22 183454_20220722_183635.jpg]

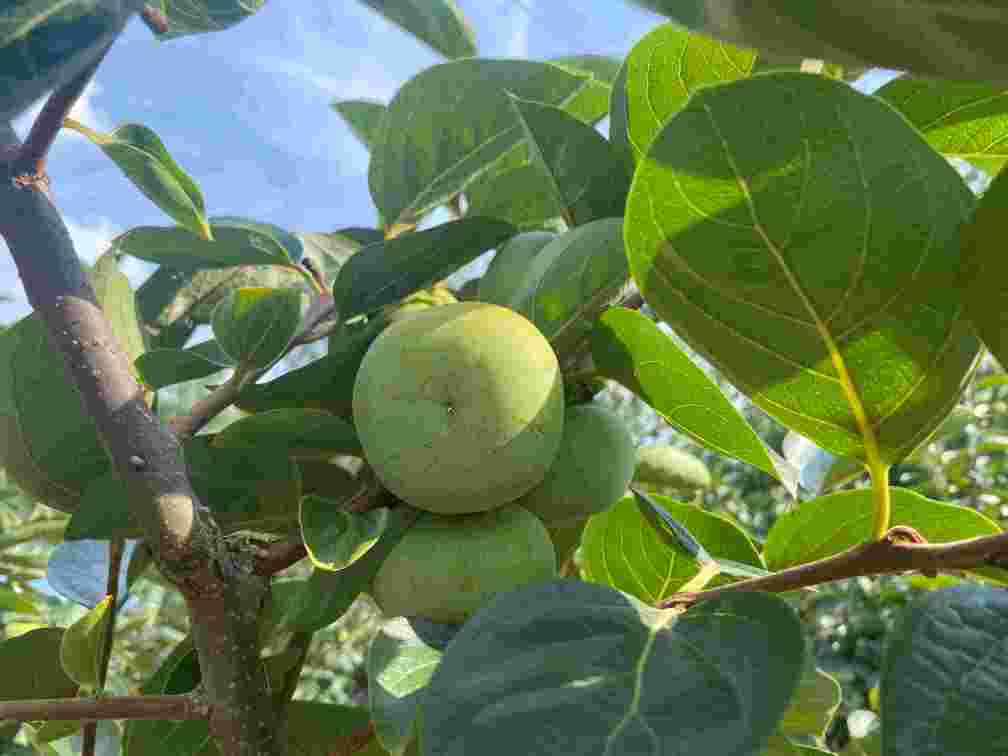

Supplement: Supplementary file 1 [file DataSheet1.zip › 2022-07-22 183454_20220722_183636.jpg]

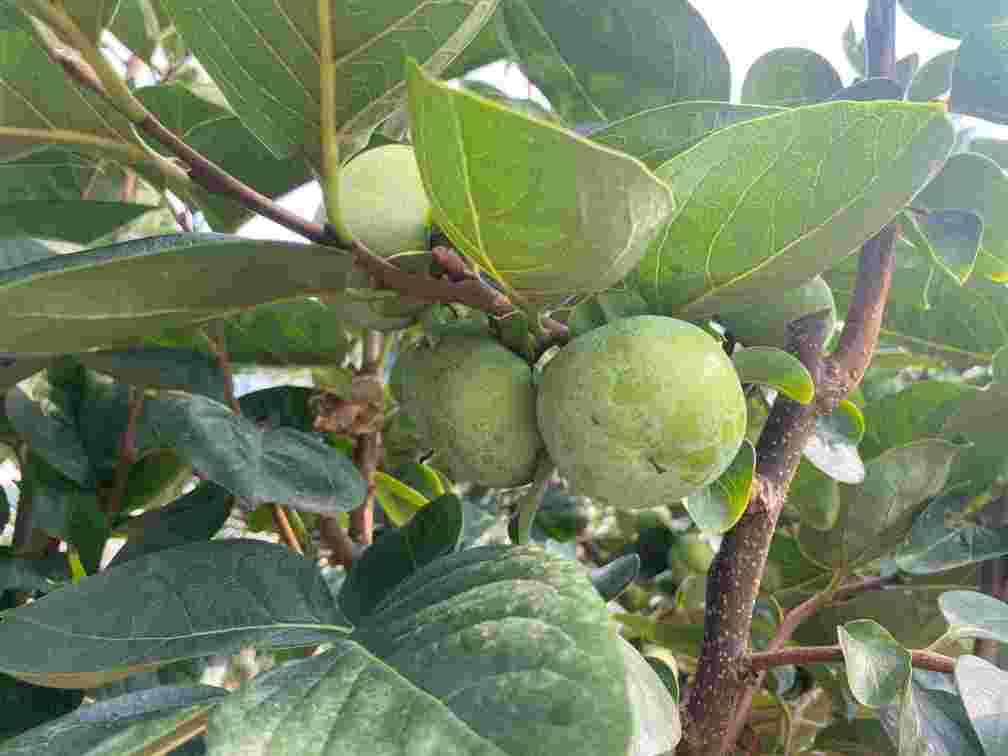

Supplement: Supplementary file 1 [file DataSheet1.zip › 2022-07-22 183454_20220722_183637.jpg]

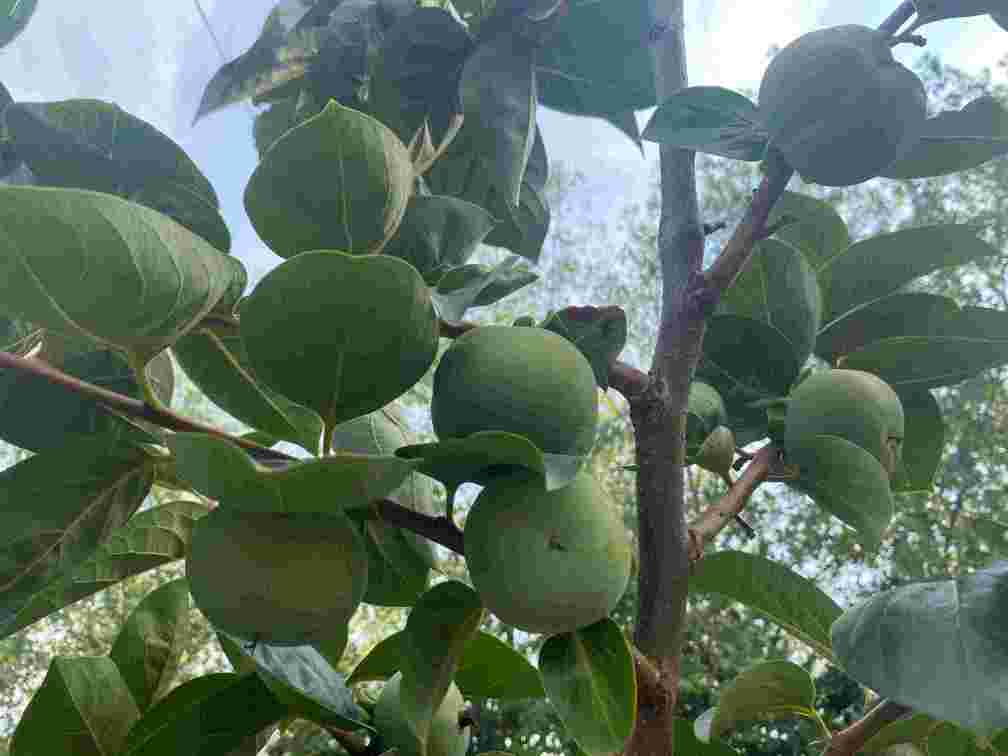

Supplement: Supplementary file 1 [file DataSheet1.zip › 2022-07-22 183454_20220722_183638.jpg]

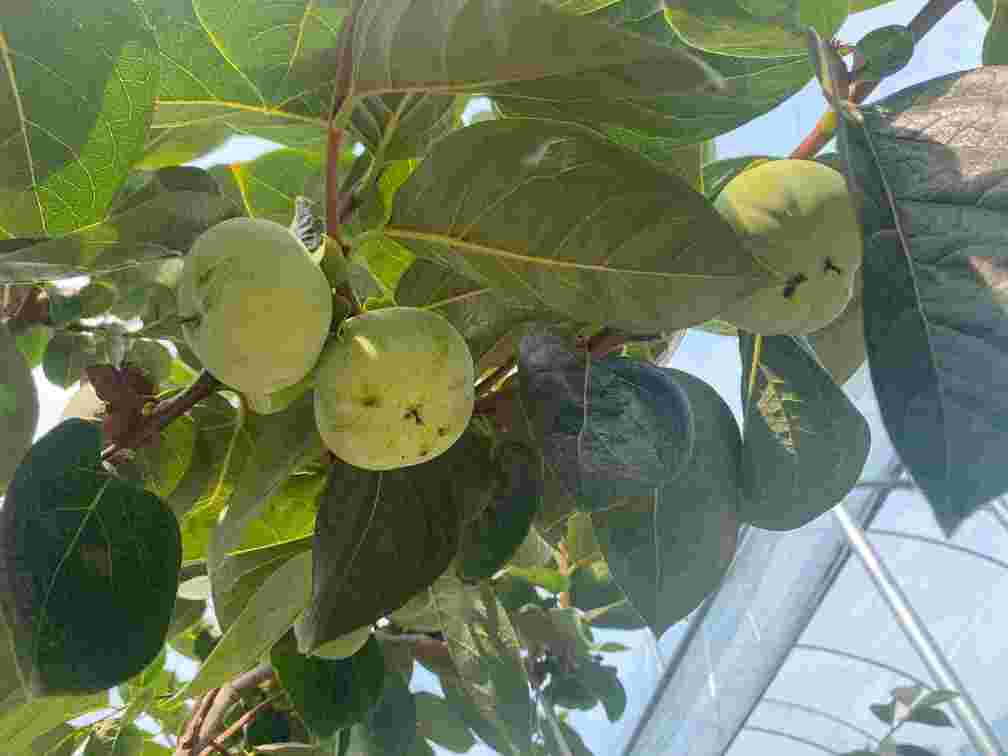

Supplement: Supplementary file 1 [file DataSheet1.zip › 2022-07-22 183454_20220722_183639.jpg]

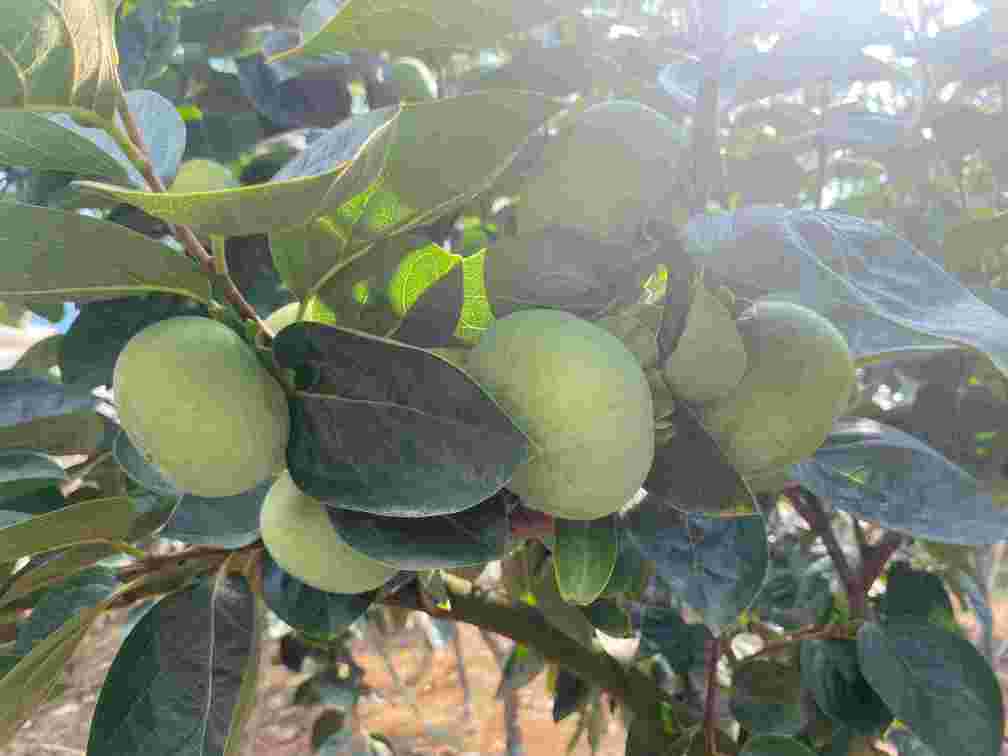

Supplement: Supplementary file 1 [file DataSheet1.zip › 2022-07-22 183454_20220722_183640.jpg]

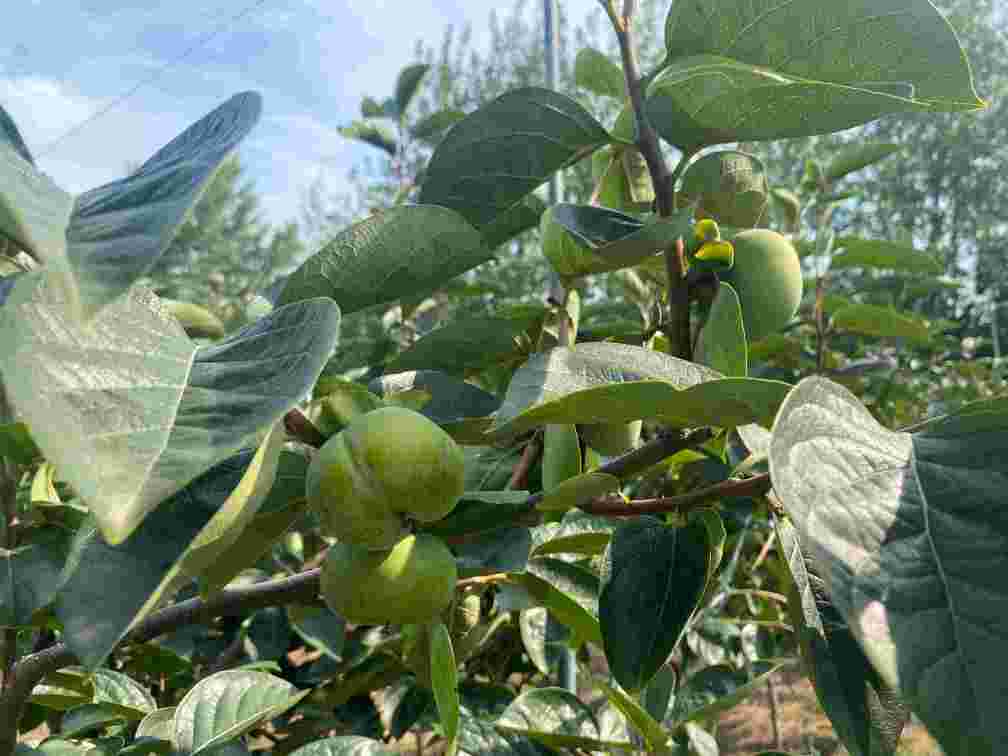

Supplement: Supplementary file 1 [file DataSheet1.zip › 2022-07-22 183454_20220722_183641.jpg]

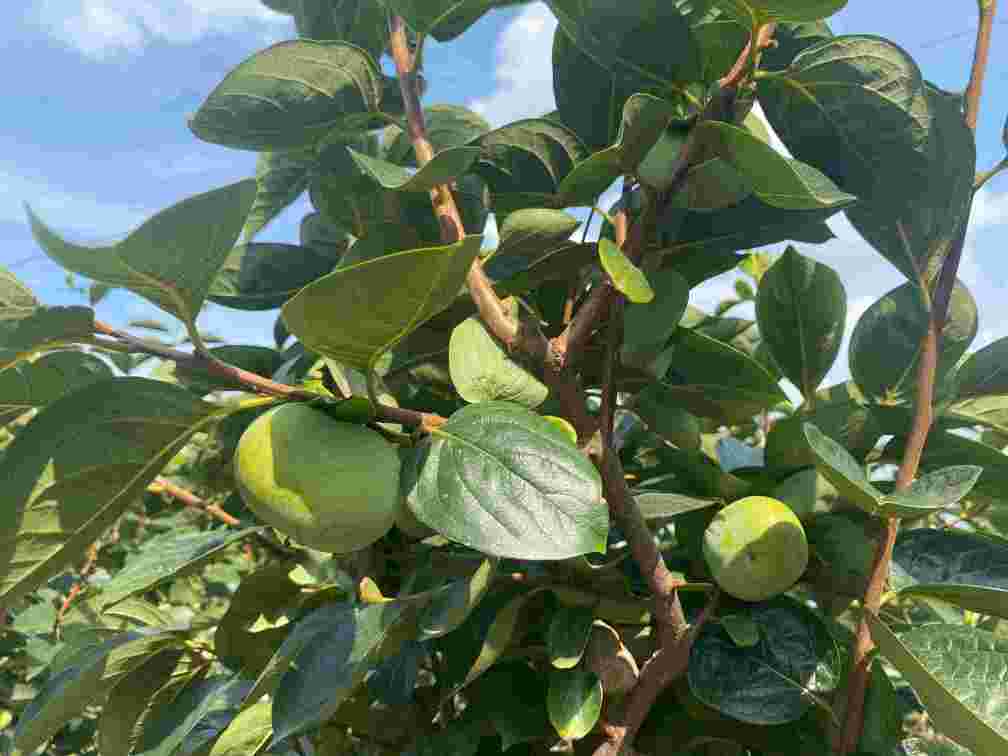

Supplement: Supplementary file 1 [file DataSheet1.zip › 2022-07-22 183454_20220722_183642.jpg]

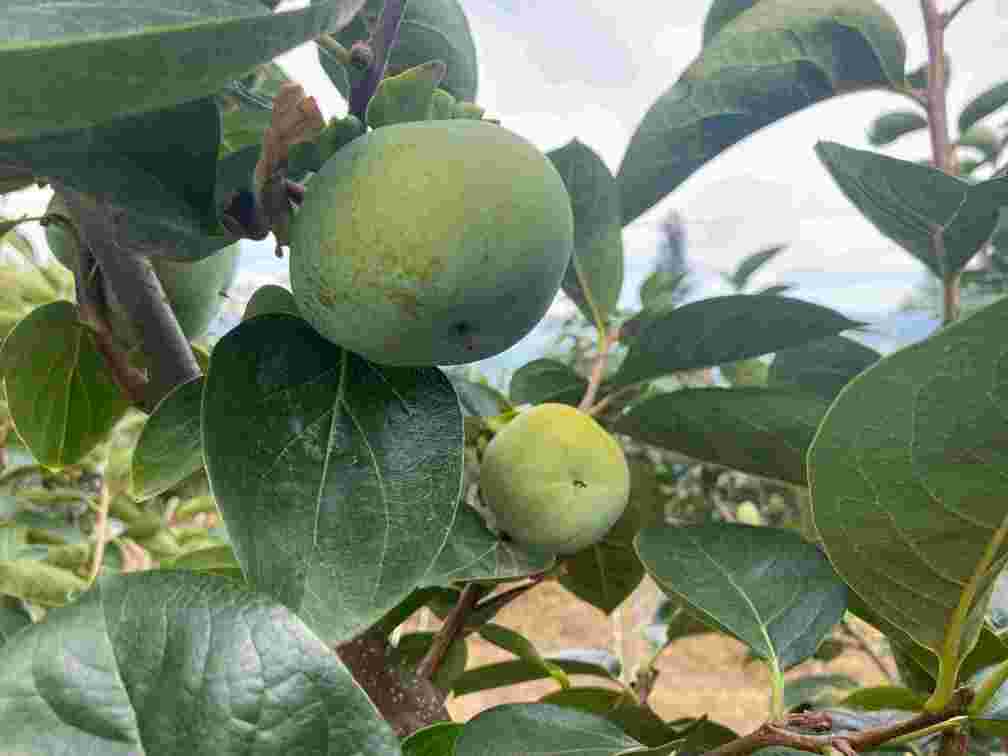

Supplement: Supplementary file 1 [file DataSheet1.zip › 2022-07-22 183454_20220722_183643.jpg]

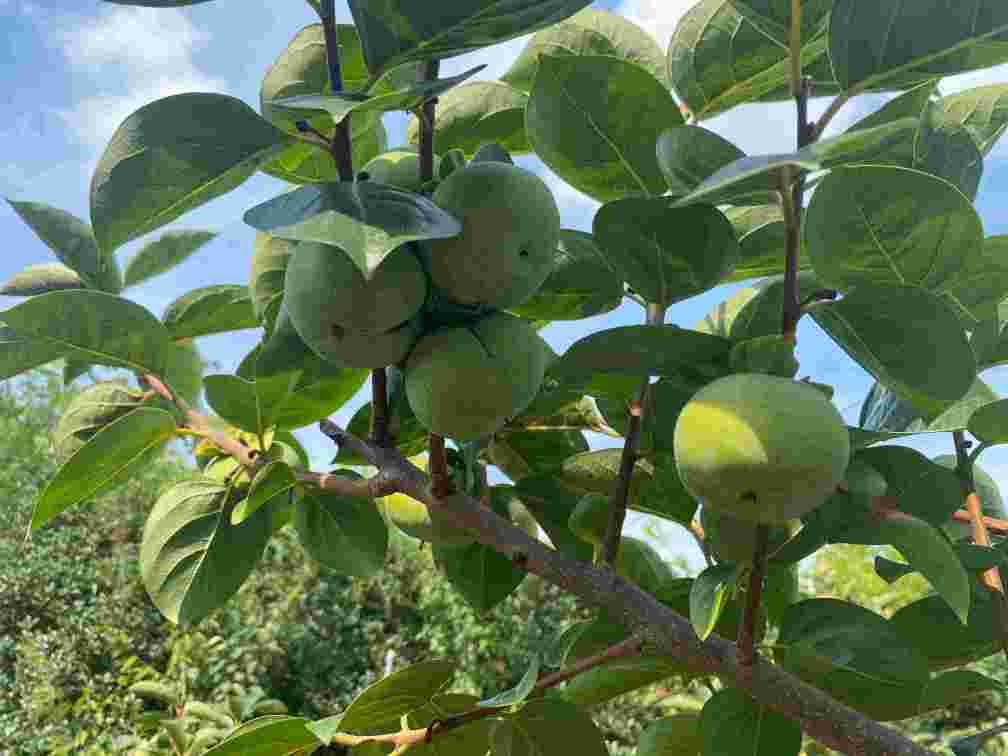

Supplement: Supplementary file 1 [file DataSheet1.zip › 2022-07-22 183454_20220722_183644.jpg]

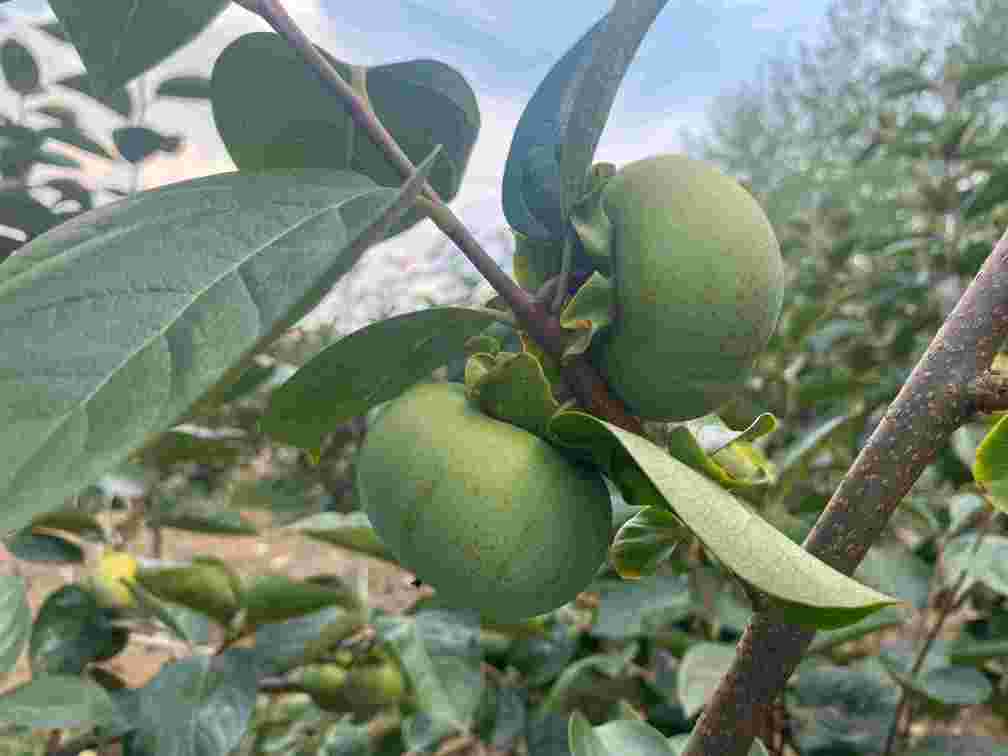

Supplement: Supplementary file 1 [file DataSheet1.zip › 2022-07-22 183454_20220722_183645.jpg]

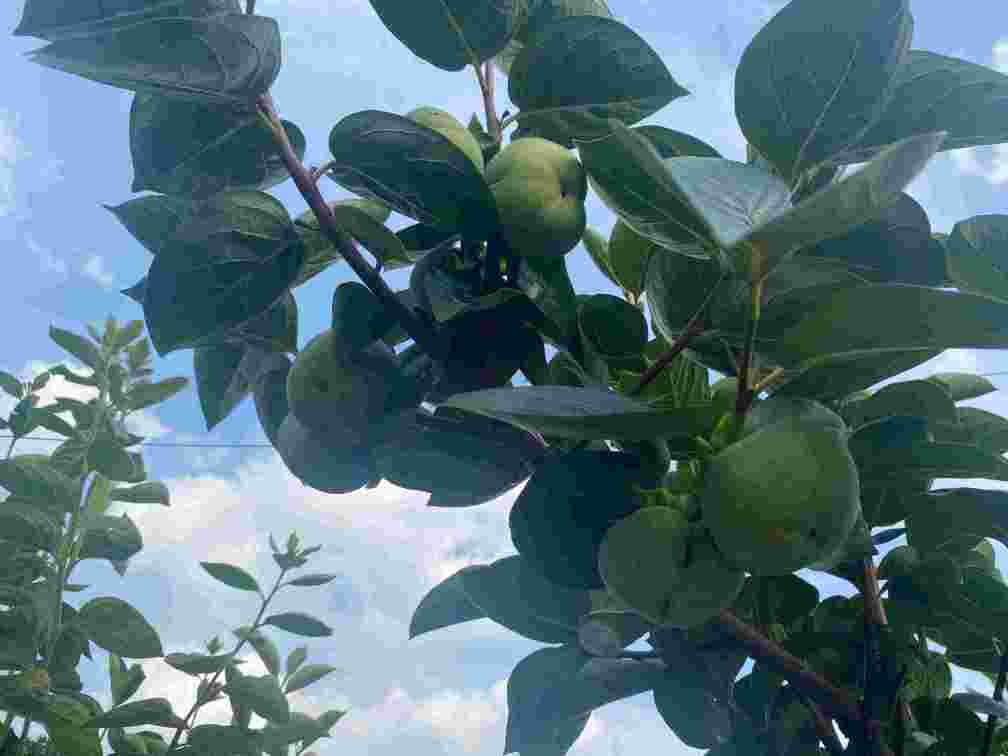

Supplement: Supplementary file 1 [file DataSheet1.zip › 2022-07-22 183454_20220722_183646.jpg]

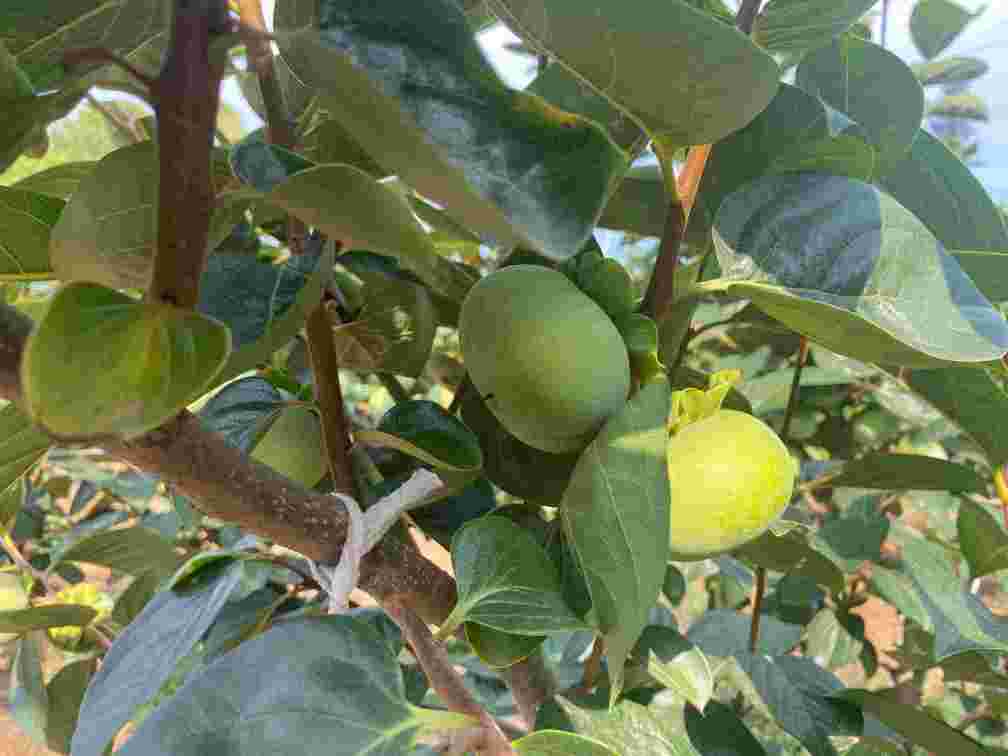

Supplement: Supplementary file 1 [file DataSheet1.zip › 2022-07-22 183454_20220722_183647.jpg]

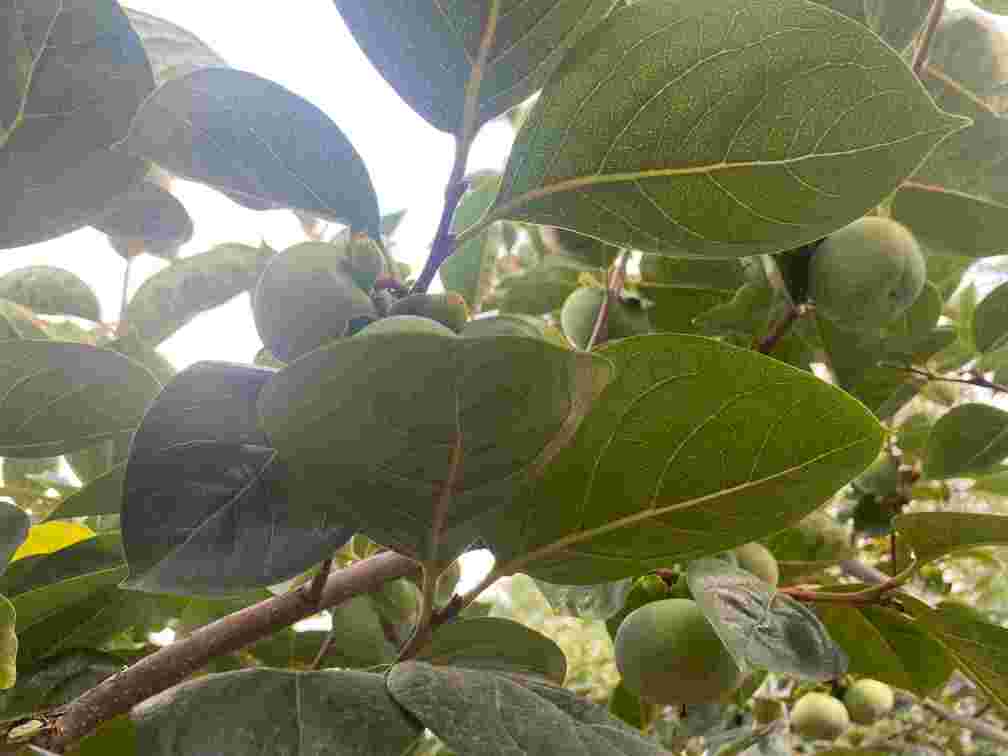

Supplement: Supplementary file 1 [file DataSheet1.zip › 2022-07-22 183454_20220722_183648.jpg]

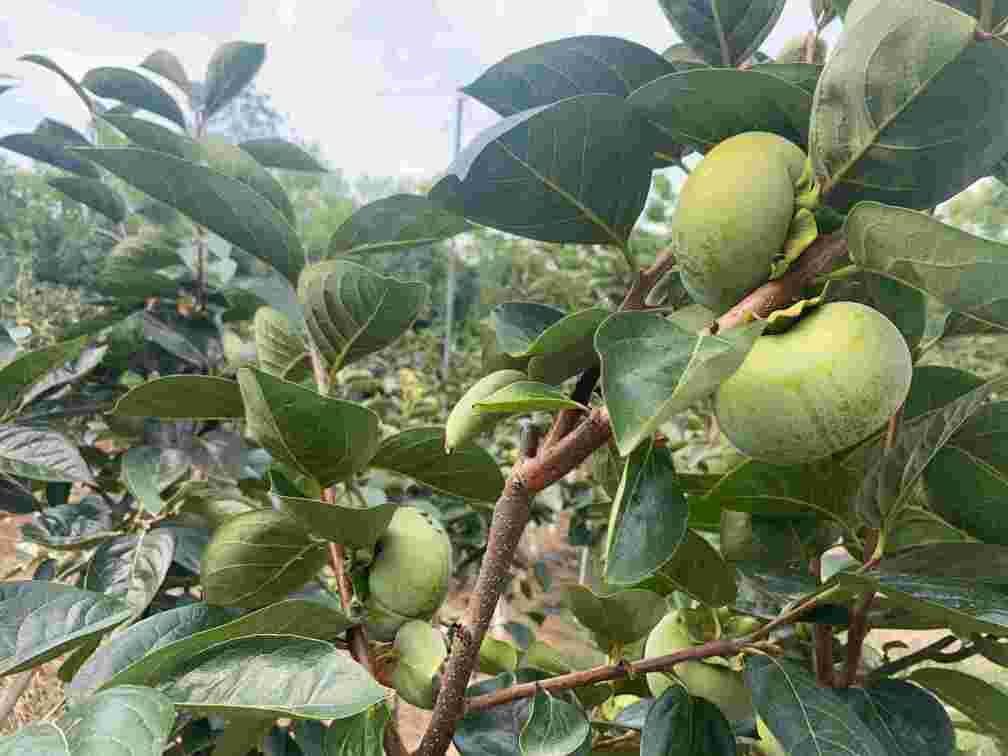

Supplement: Supplementary file 1 [file DataSheet1.zip › 2022-07-22 183454_20220722_183650.jpg]

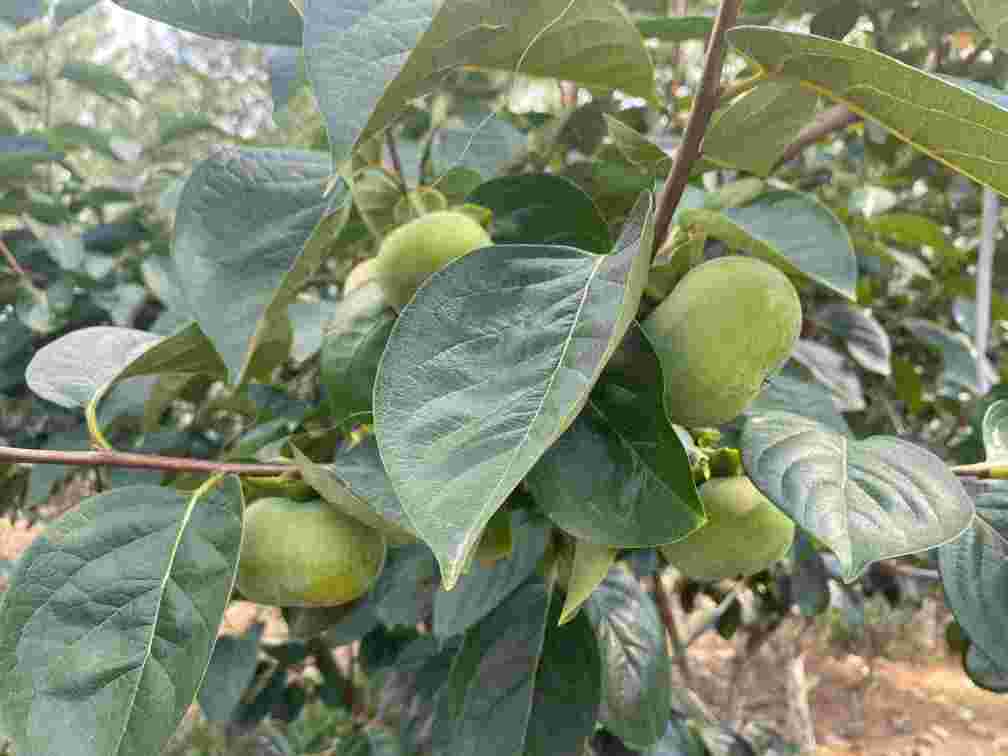

Supplement: Supplementary file 1 [file DataSheet1.zip › 2022-07-22 183454_20220722_183651.jpg]

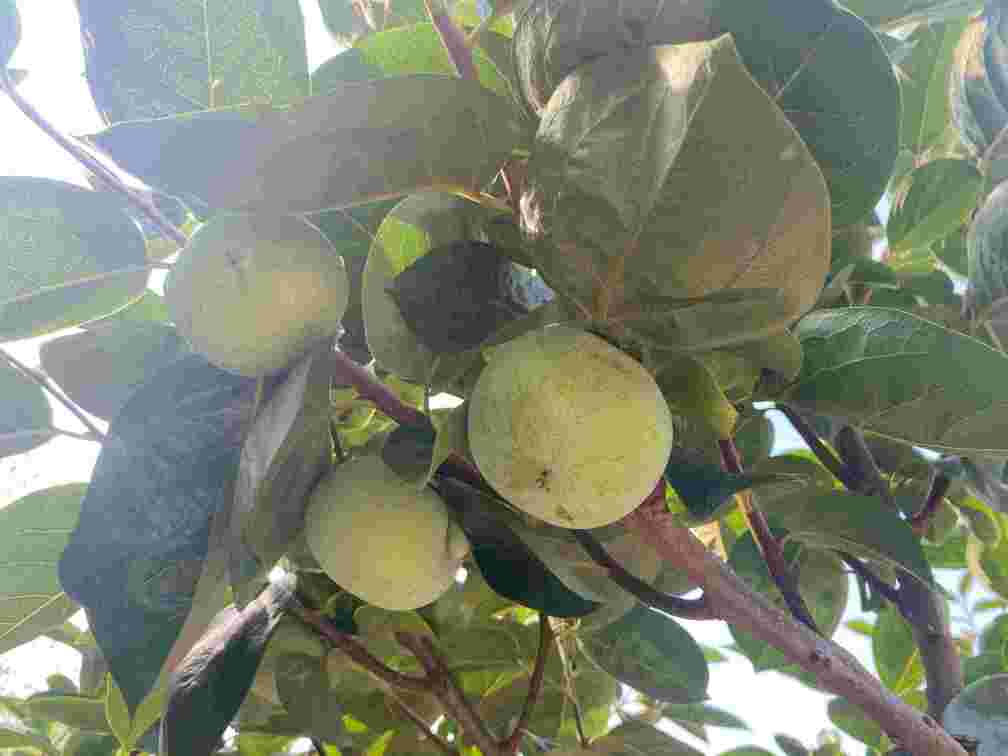

Supplement: Supplementary file 1 [file DataSheet1.zip › 2022-07-22 183454_20220722_183652.jpg]

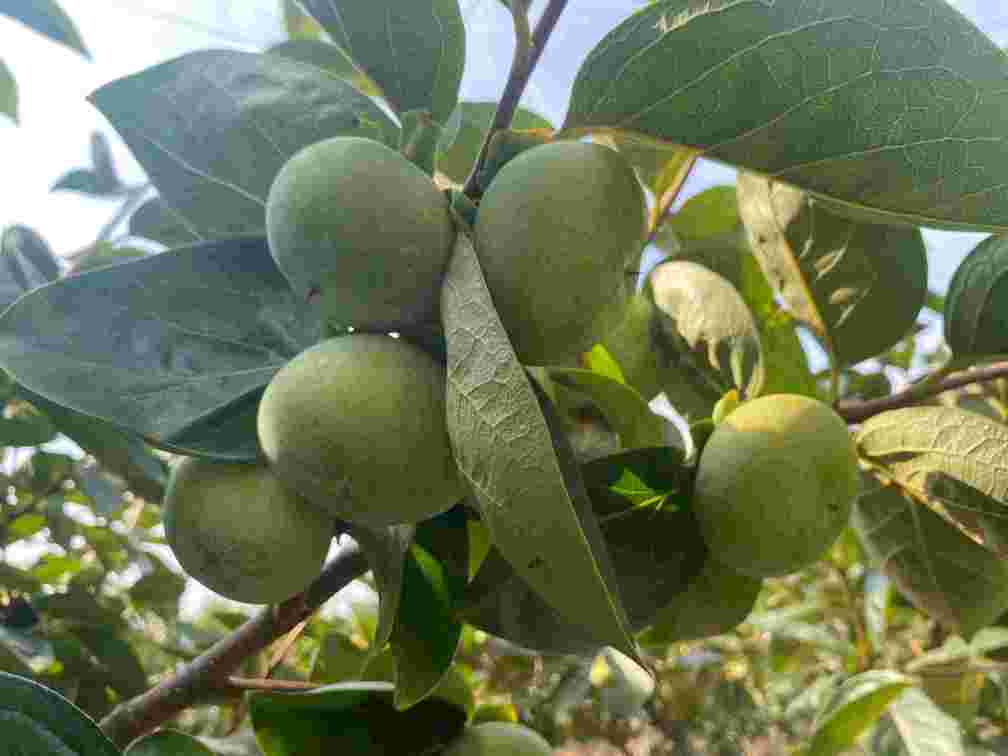

Supplement: Supplementary file 1 [file DataSheet1.zip › 2022-07-22 183454_20220722_183653.jpg]

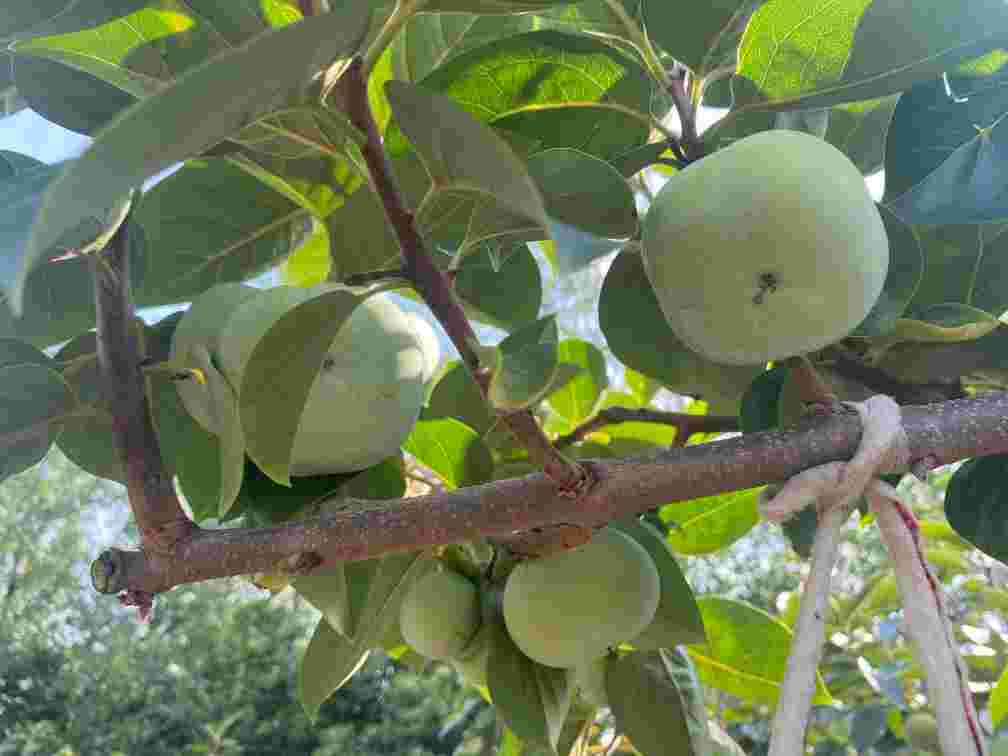

Supplement: Supplementary file 1 [file DataSheet1.zip › 2022-07-22 183454_20220722_183654.jpg]

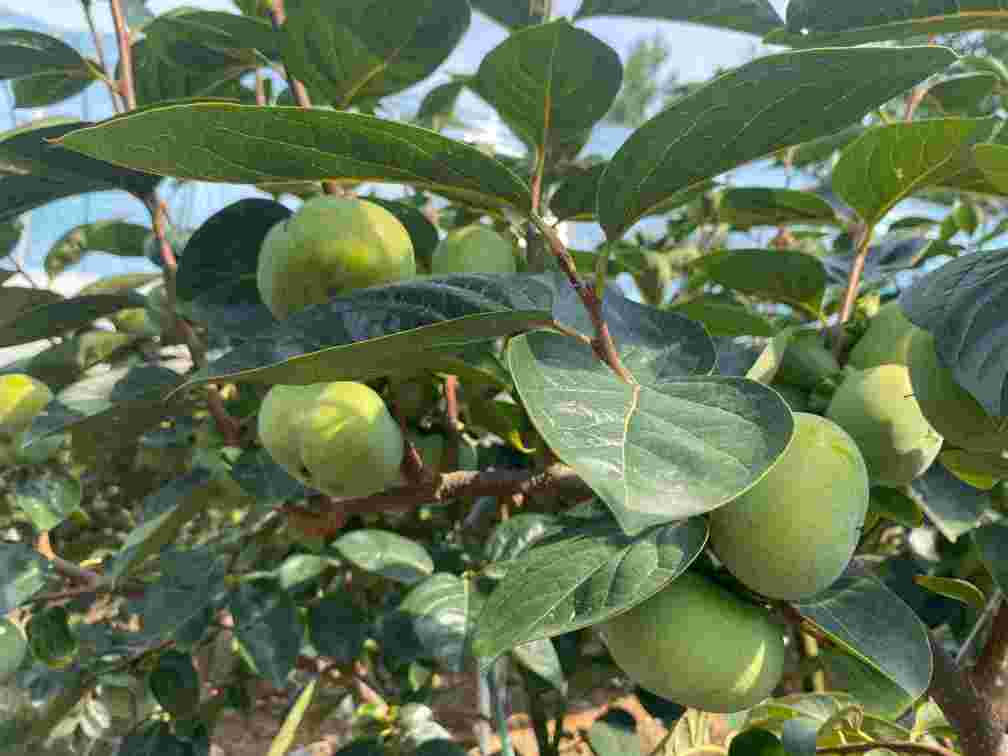

Supplement: Supplementary file 1 [file DataSheet1.zip › 2022-07-22 183454_20220722_183655.jpg]

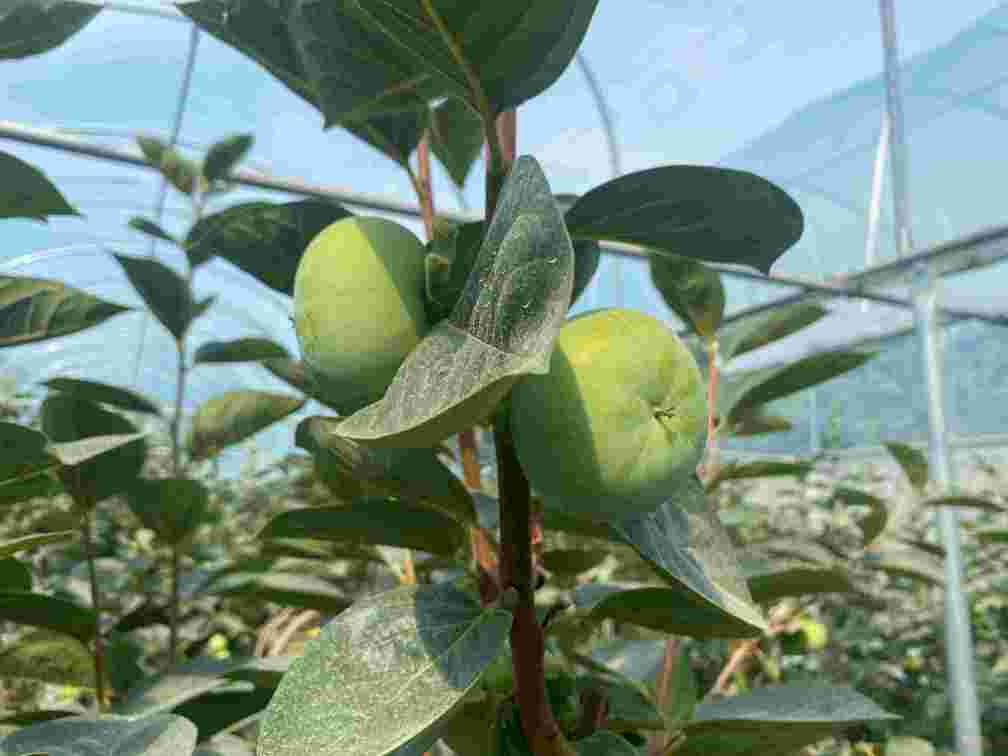

Supplement: Supplementary file 1 [file DataSheet1.zip › 2022-07-22 183454_20220722_183657.jpg]

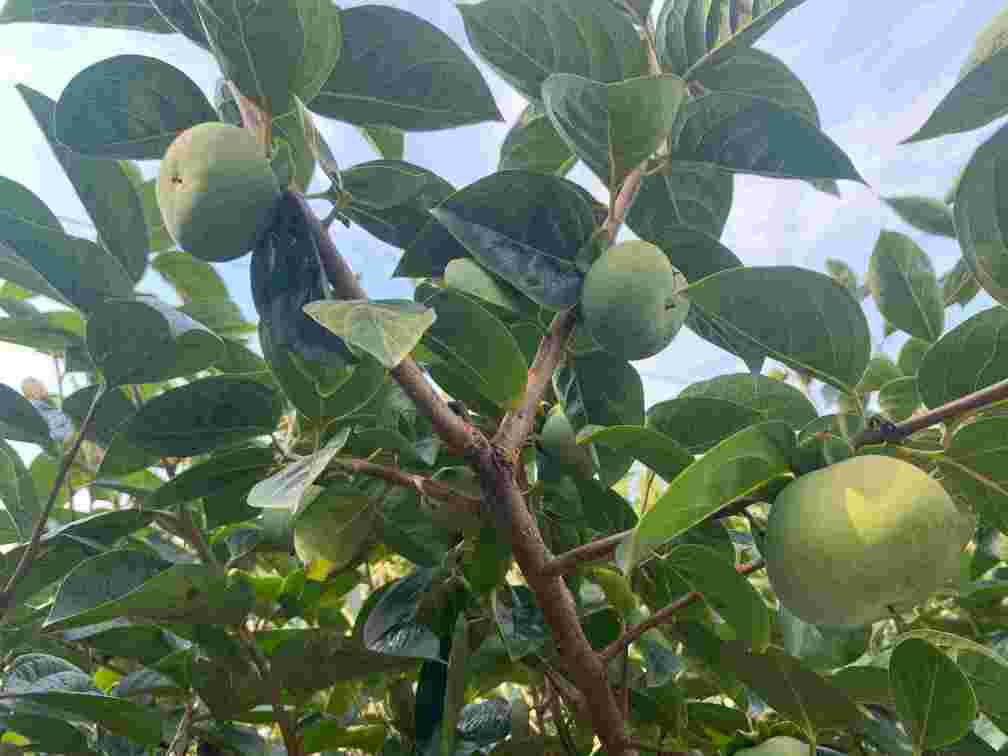

Supplement: Supplementary file 1 [file DataSheet1.zip › 2022-07-22 183454_20220722_183658.jpg]

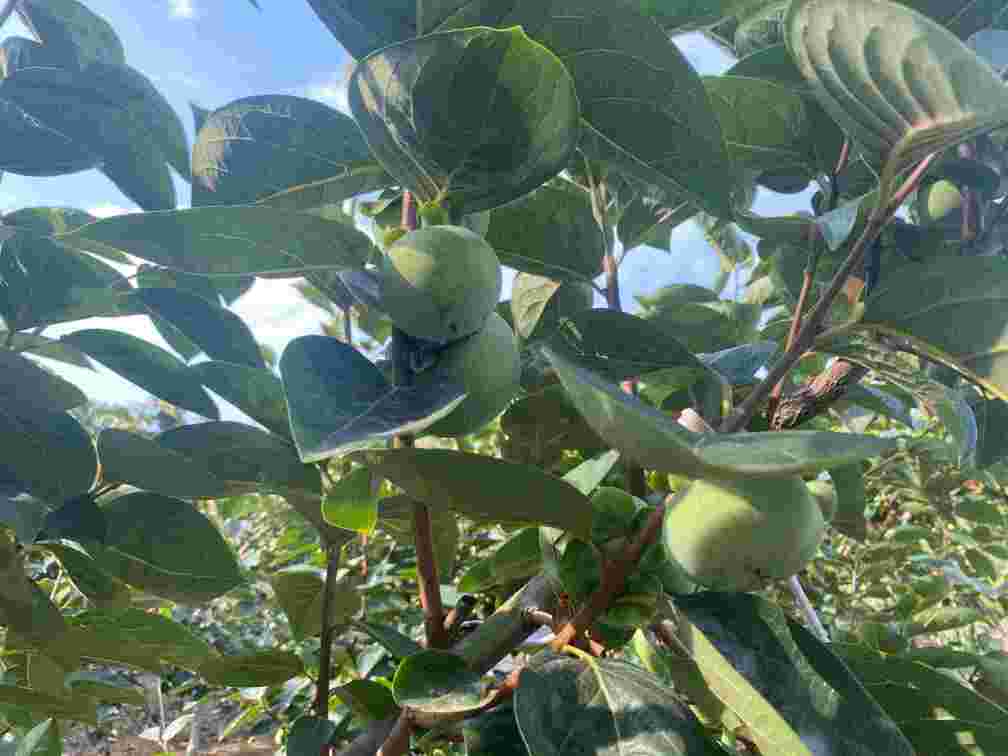

Supplement: Supplementary file 1 [file DataSheet1.zip › 2022-07-22 183454_20220722_183659.jpg]

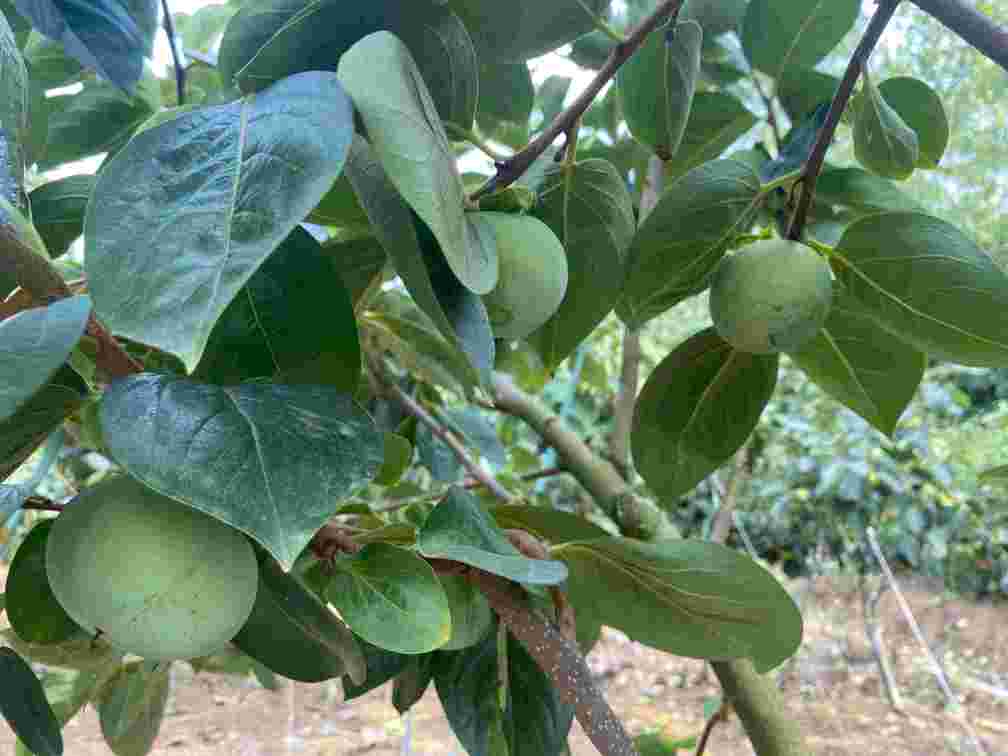

Supplement: Supplementary file 1 [file DataSheet1.zip › 2022-07-22 183454_20220722_183700.jpg]

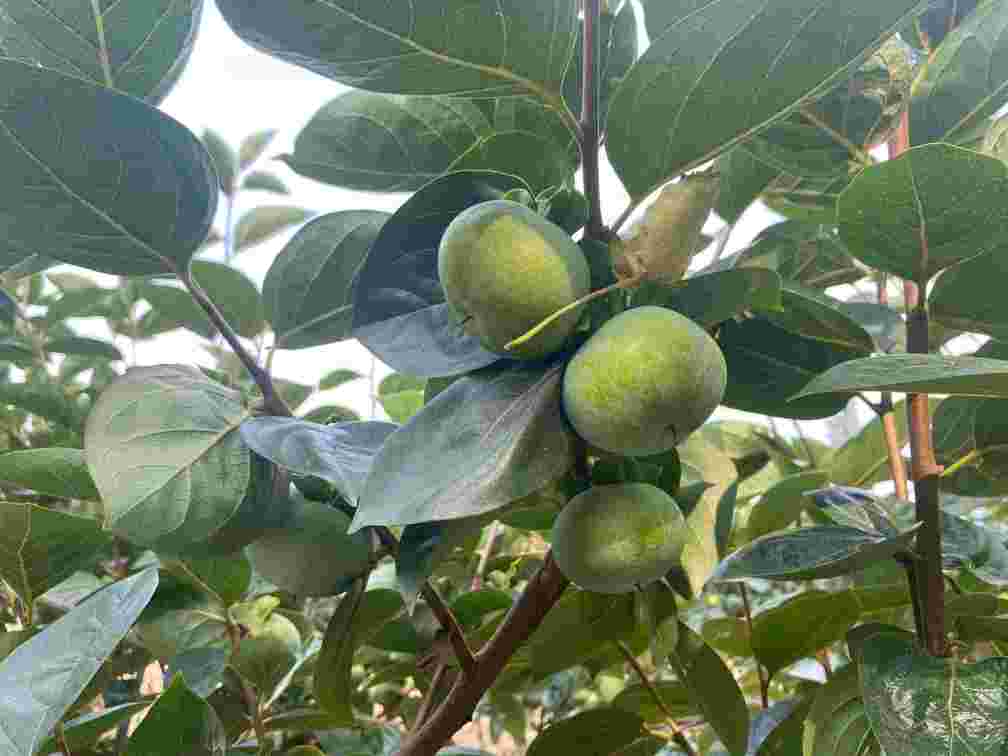

Supplement: Supplementary file 1 [file DataSheet1.zip › 2022-07-22 183454_20220722_183701.jpg]

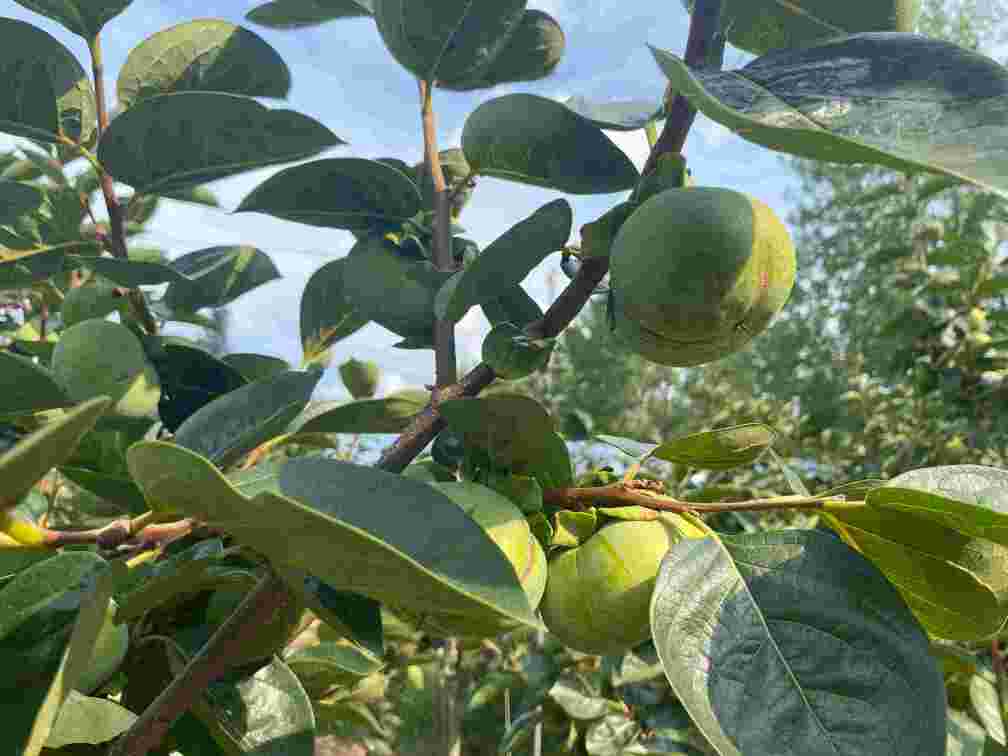

Supplement: Supplementary file 1 [file DataSheet1.zip › 2022-07-22 183454_20220722_183702.jpg]

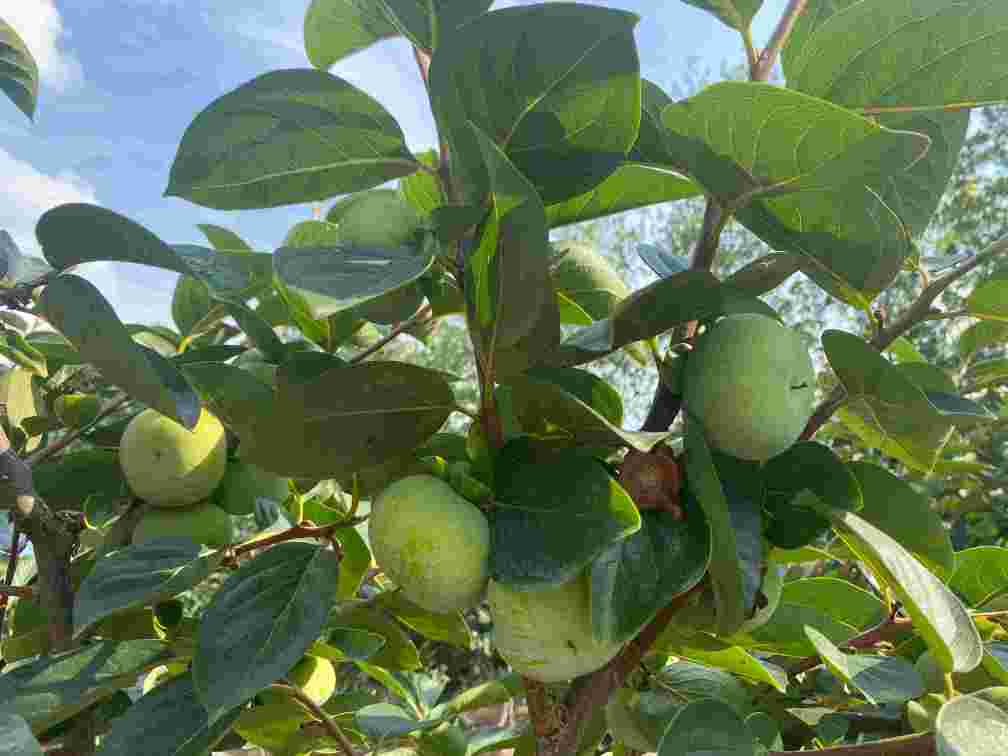

Supplement: Supplementary file 1 [file DataSheet1.zip › 2022-07-22 183454_20220722_183703.jpg]

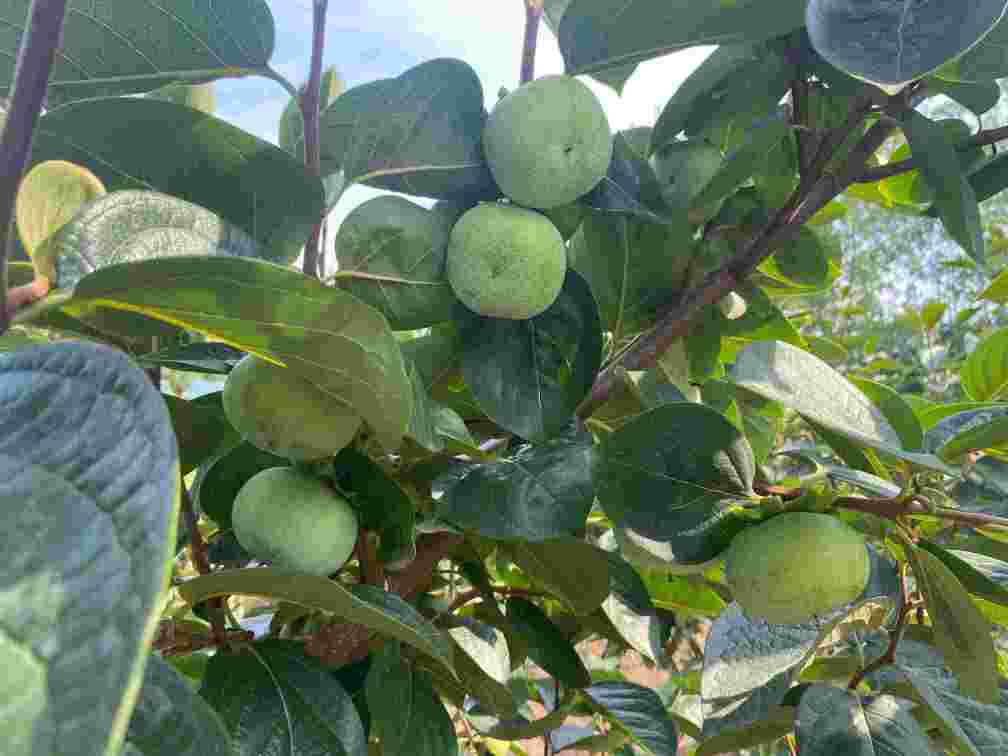

Supplement: Supplementary file 1 [file DataSheet1.zip › 2022-07-22 183454_20220722_183704.jpg]

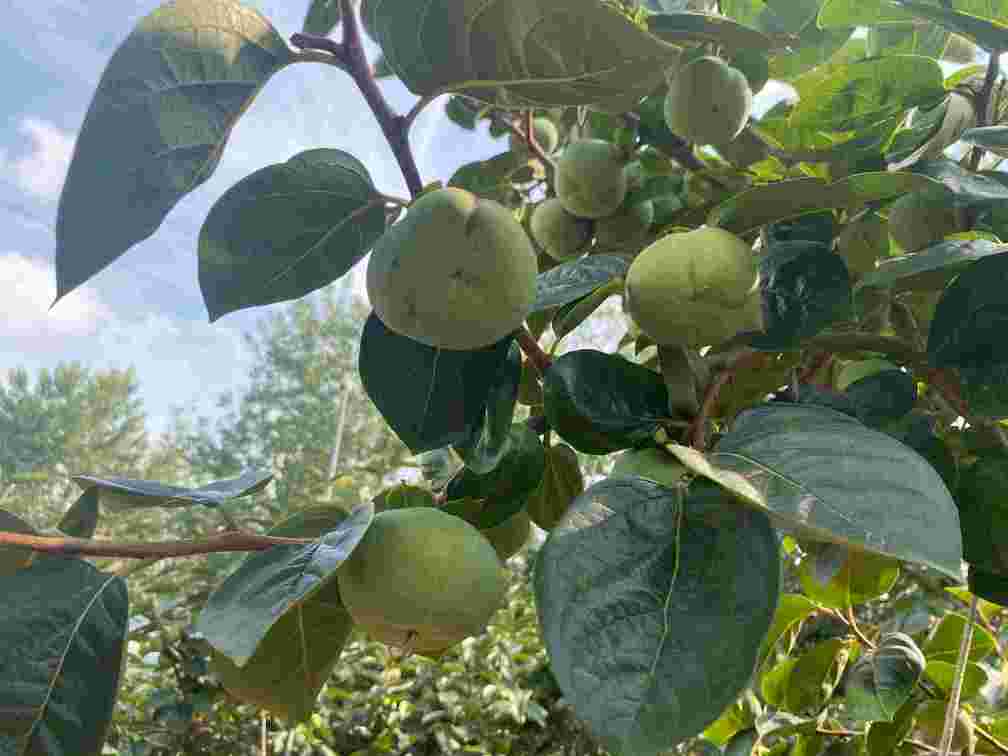

Supplement: Supplementary file 1 [file DataSheet1.zip › 2022-07-22 183454_20220722_183705.jpg]

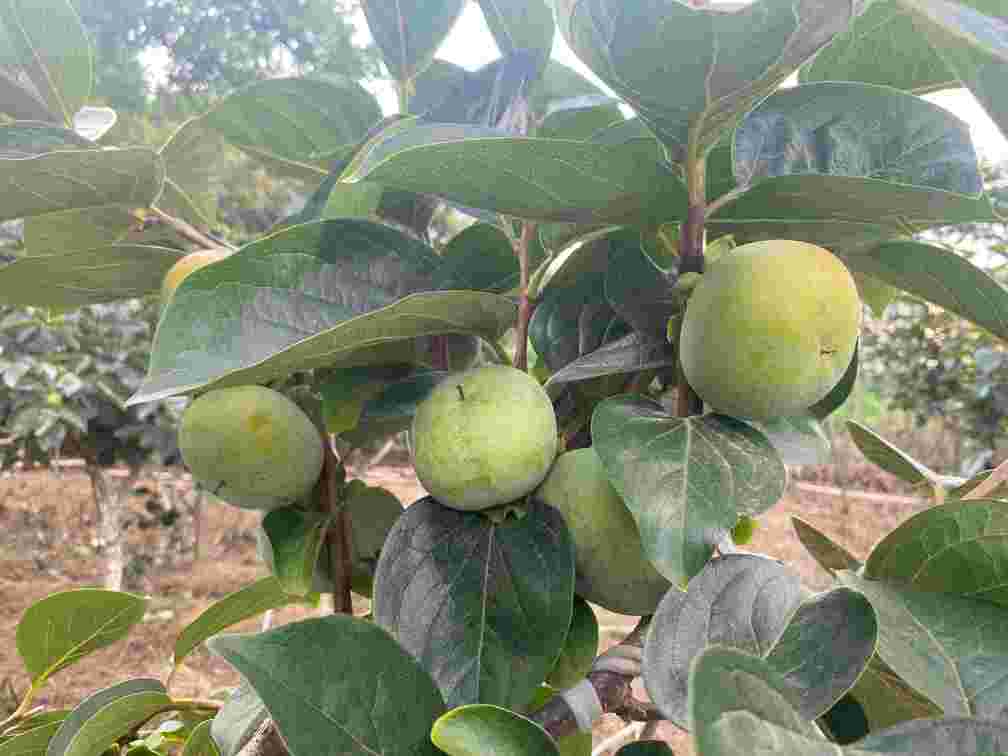

Supplement: Supplementary file 1 [file DataSheet1.zip › 2022-07-22 183454_20220722_183706.jpg]

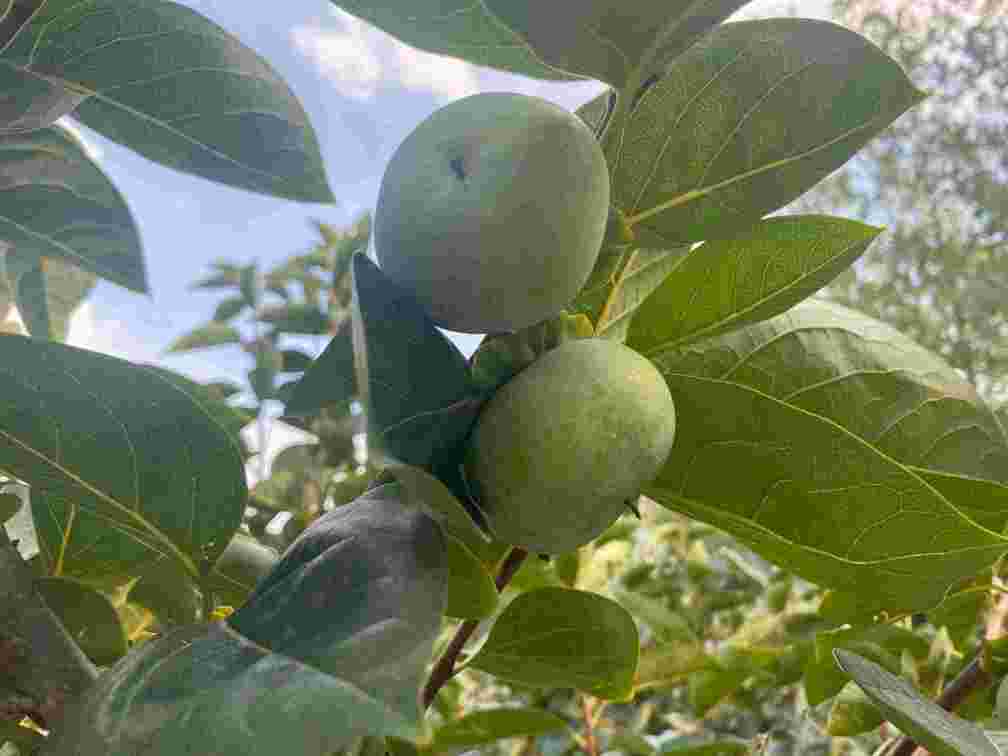

Supplement: Supplementary file 1 [file DataSheet1.zip › 2022-07-22 183454_20220722_183707.jpg]

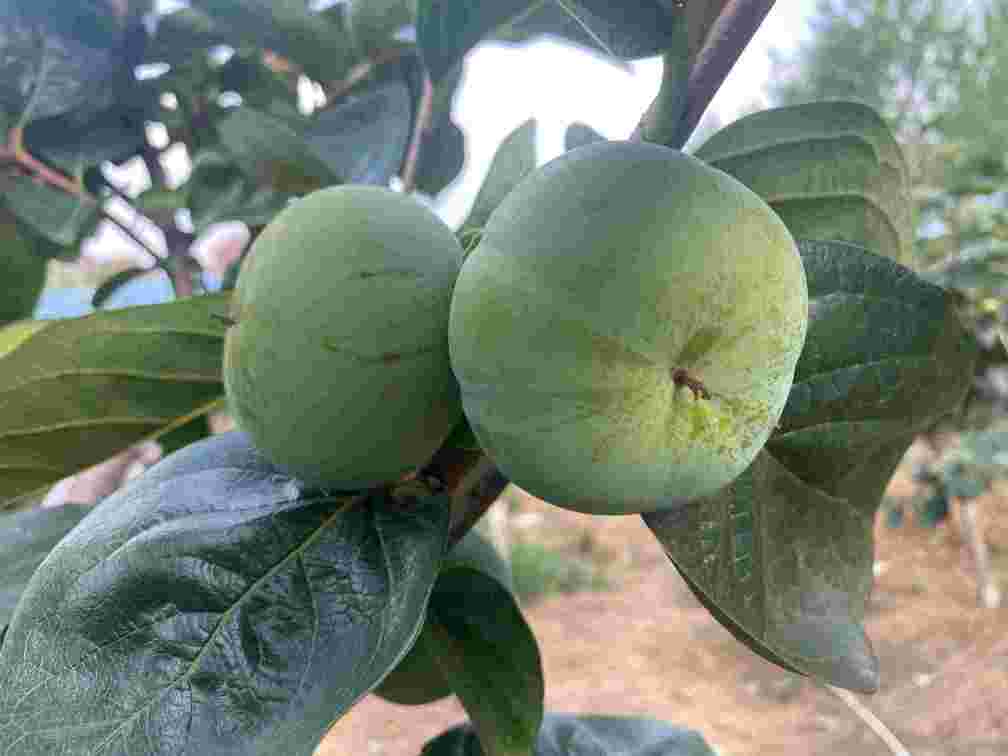

Supplement: Supplementary file 1 [file DataSheet1.zip › 2022-07-22 183454_20220722_183709.jpg]

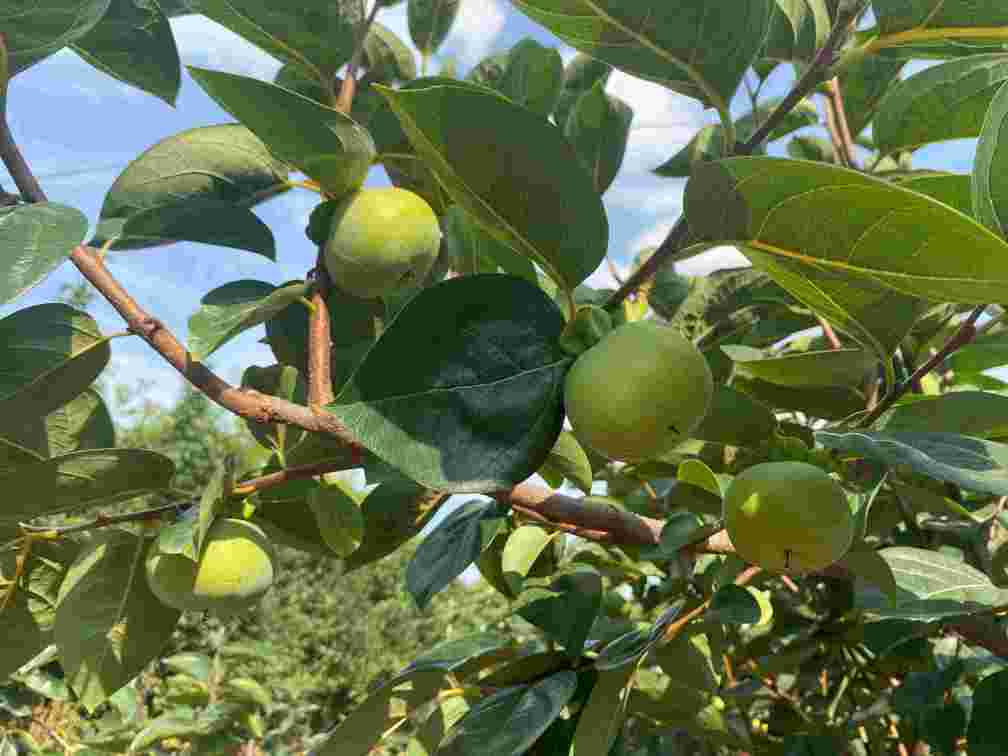

Supplement: Supplementary file 1 [file DataSheet1.zip › 2022-07-22 183454_20220722_183710.jpg]

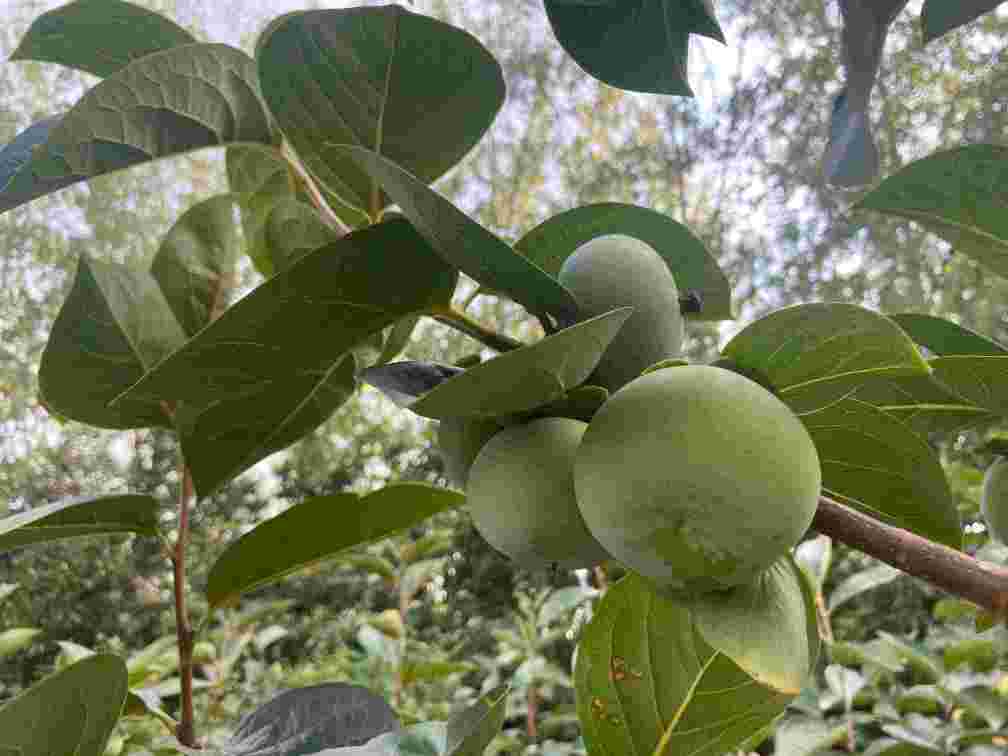

Supplement: Supplementary file 1 [file DataSheet1.zip › 2022-07-22 183454_20220722_183711.jpg]

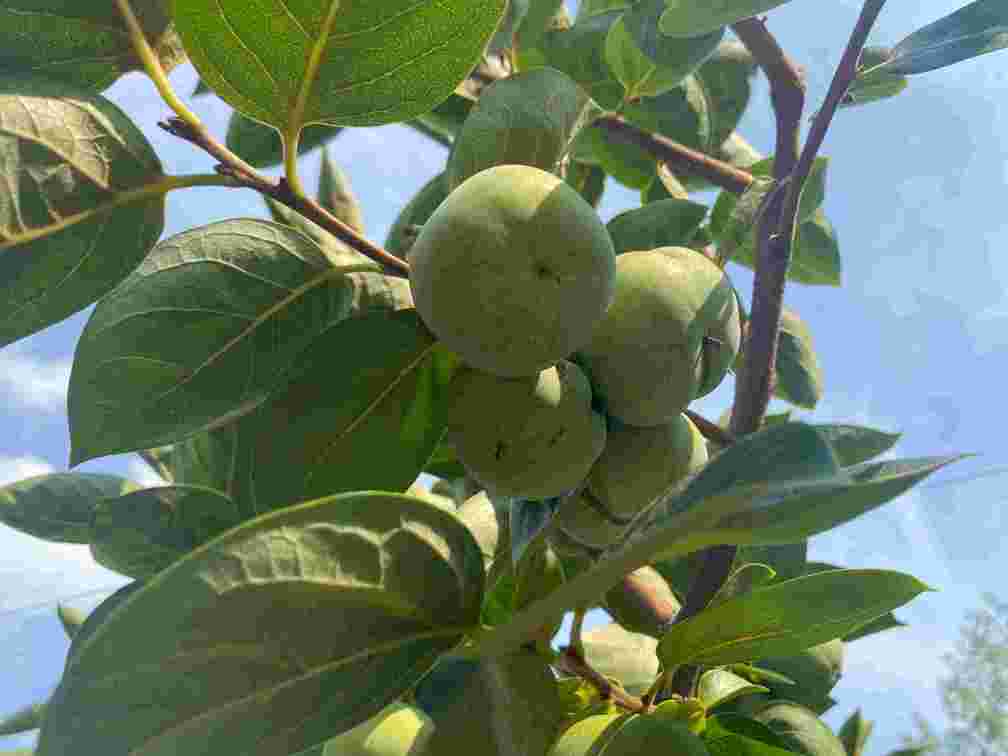

Supplement: Supplementary file 1 [file DataSheet1.zip › 2022-07-22 183454_20220722_183712.jpg]

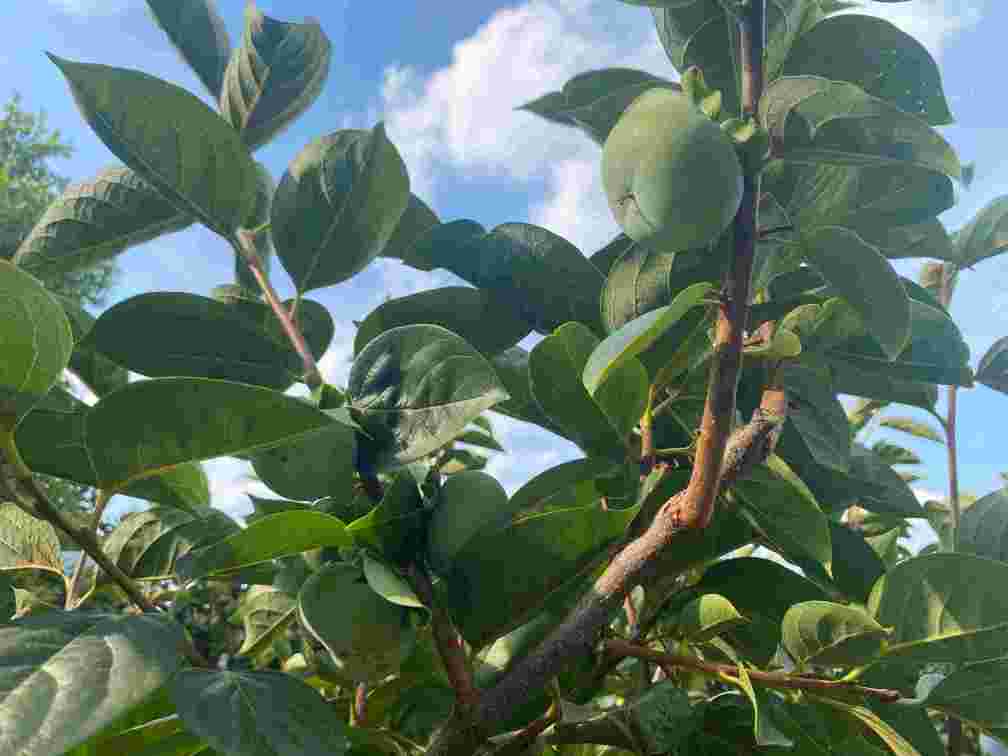

Supplement: Supplementary file 1 [file DataSheet1.zip › 2022-07-22 183454_20220722_183713.jpg]

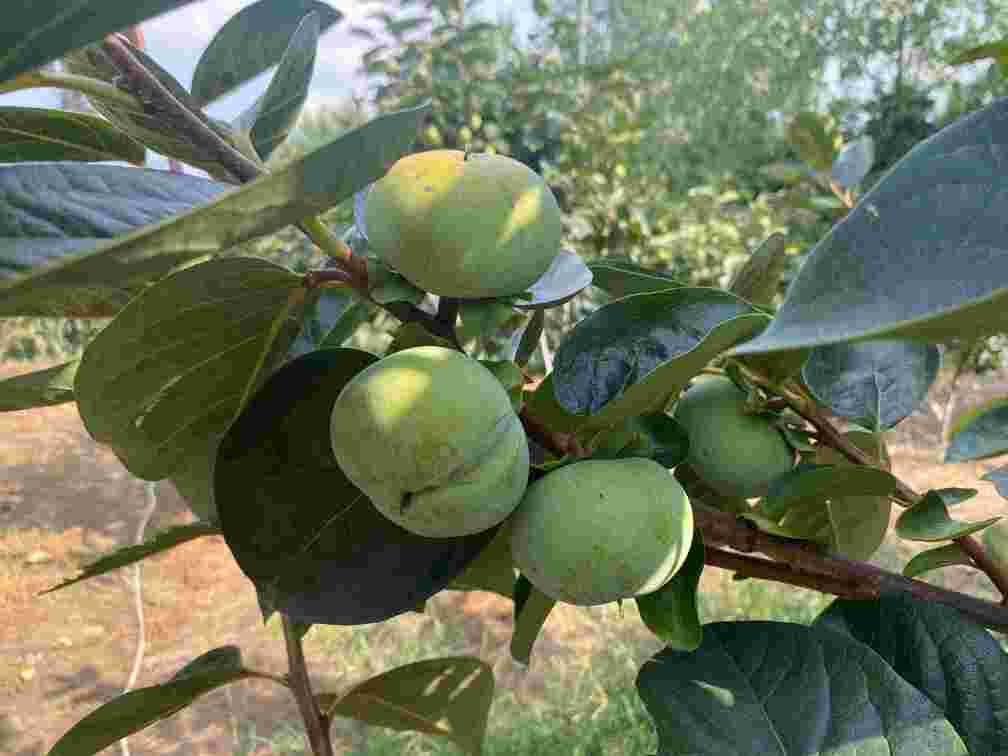

Supplement: Supplementary file 1 [file DataSheet1.zip › 2022-07-22 183454_20220722_183714.jpg]

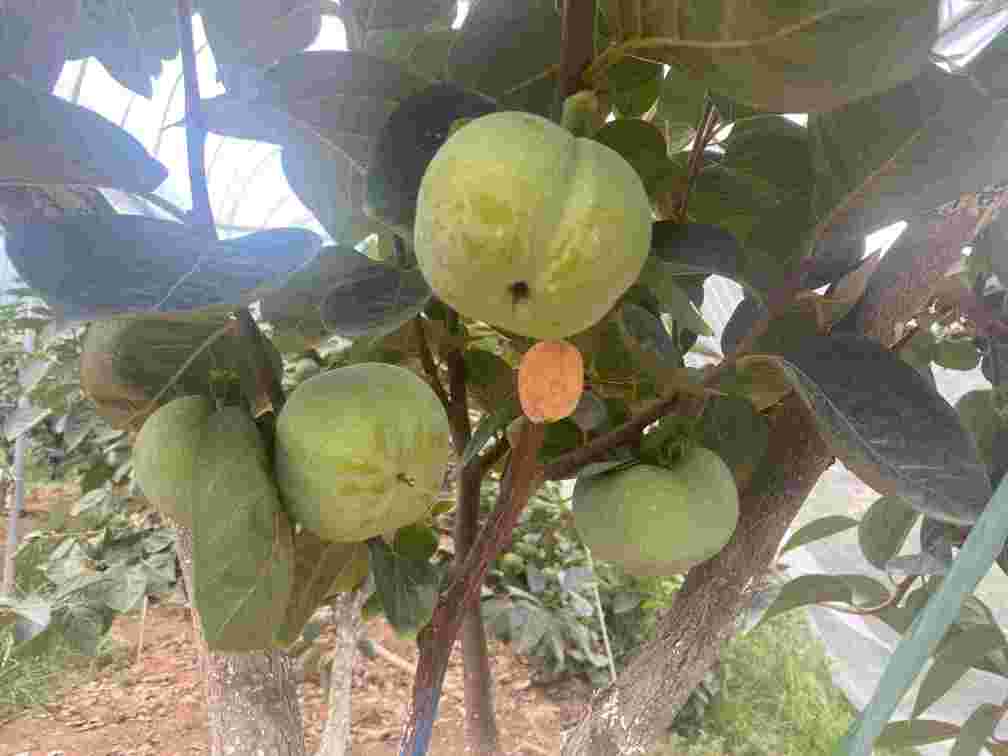

Supplement: Supplementary file 1 [file DataSheet1.zip › 2022-07-22 183454_20220722_183715.jpg]

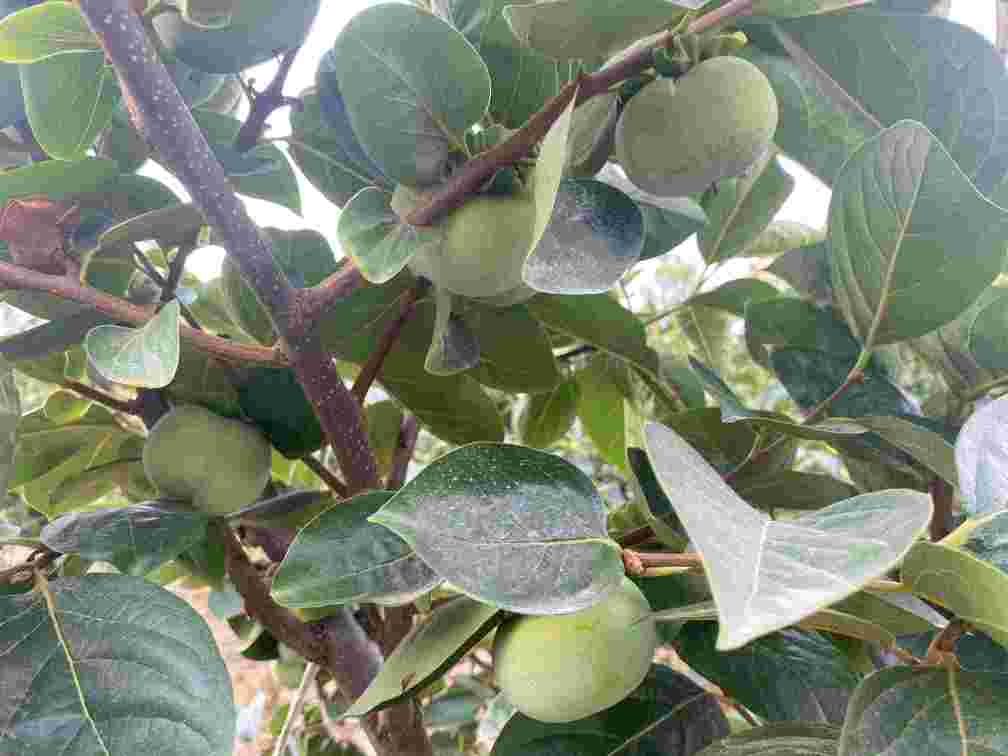

Supplement: Supplementary file 1 [file DataSheet1.zip › 2022-07-22 183454_20220722_183716.jpg]

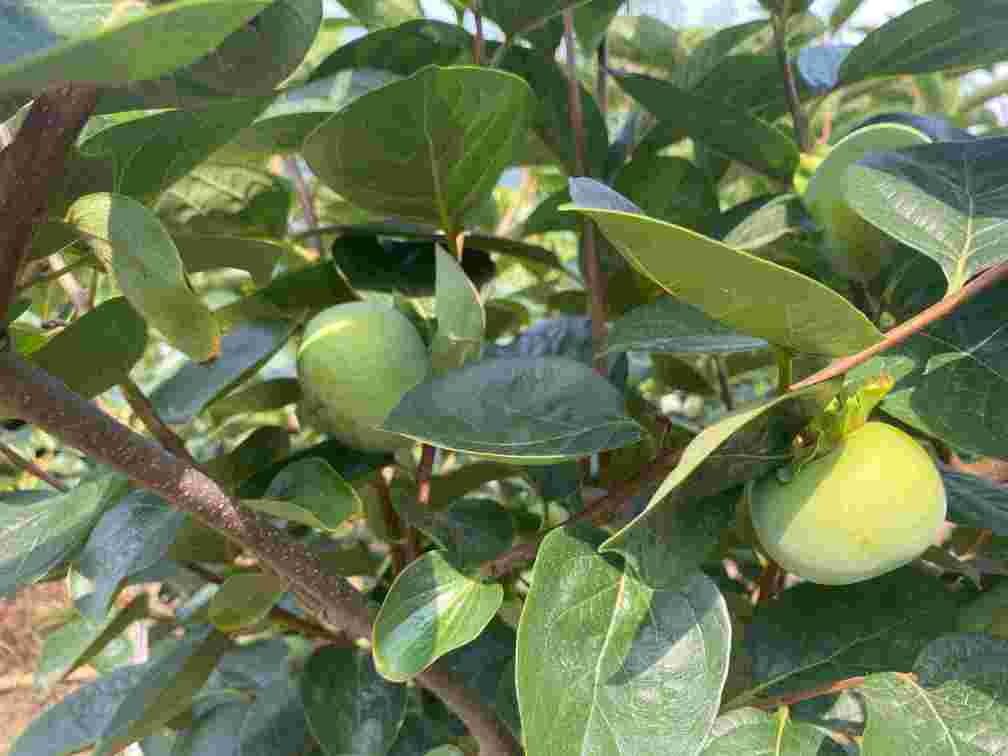

Supplement: Supplementary file 1 [file DataSheet1.zip › 2022-07-22 183454_20220722_183717.jpg]

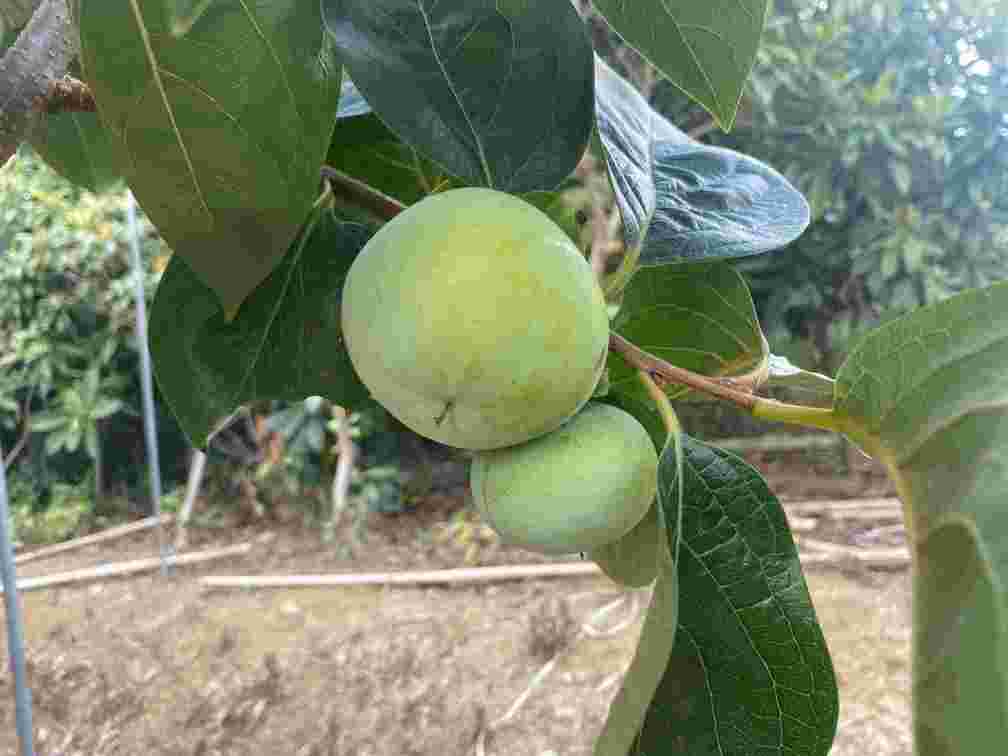

Supplement: Supplementary file 1 [file DataSheet1.zip › 2022-07-22 183454_20220722_183718.jpg]

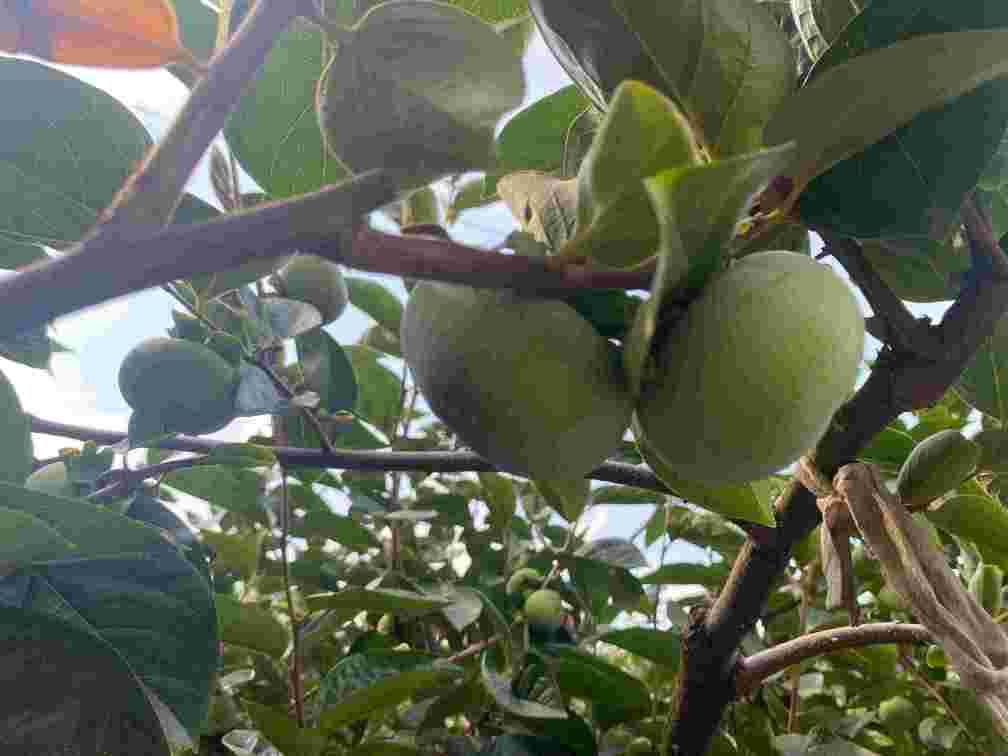

Supplement: Supplementary file 1 [file DataSheet1.zip › 2022-07-22 183454_20220722_183719.jpg]

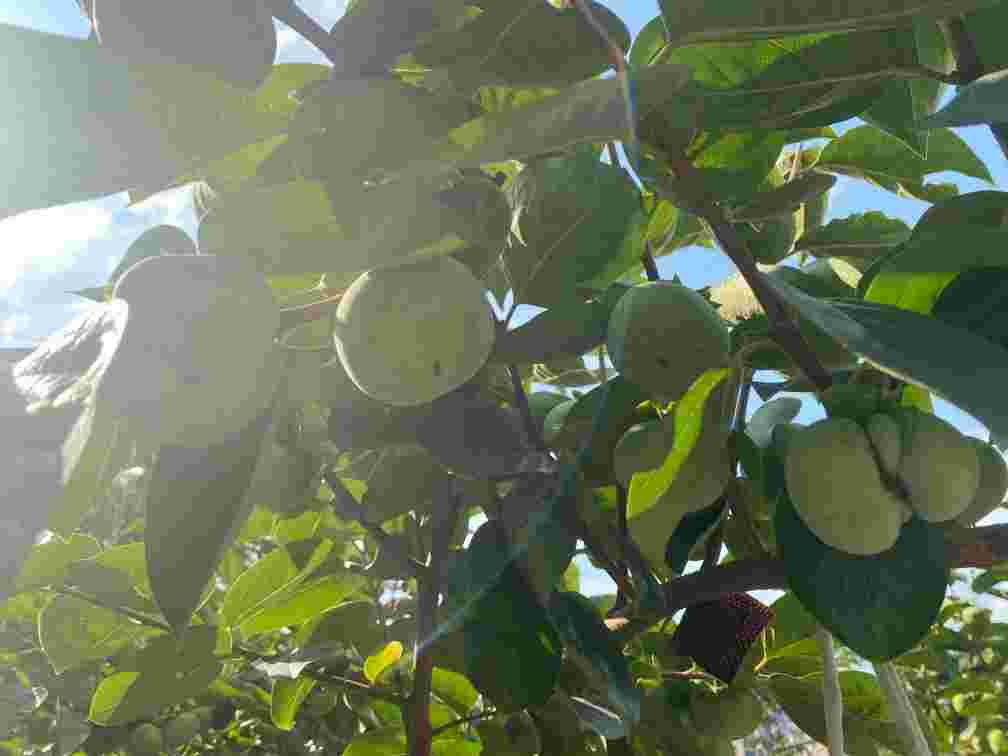

Supplement: Supplementary file 1 [file DataSheet1.zip › 2022-07-22 183454_20220722_183720.jpg]

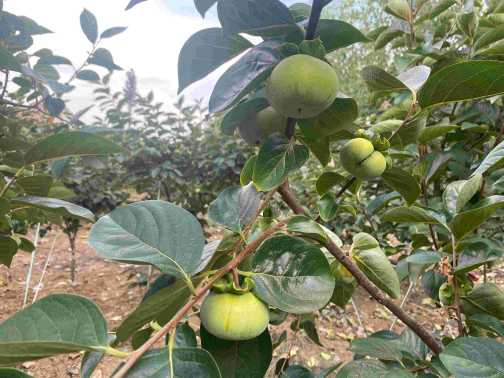

Supplement: Supplementary file 1 [file DataSheet1.zip › 2022-07-22 183454_20220722_183721.jpg]

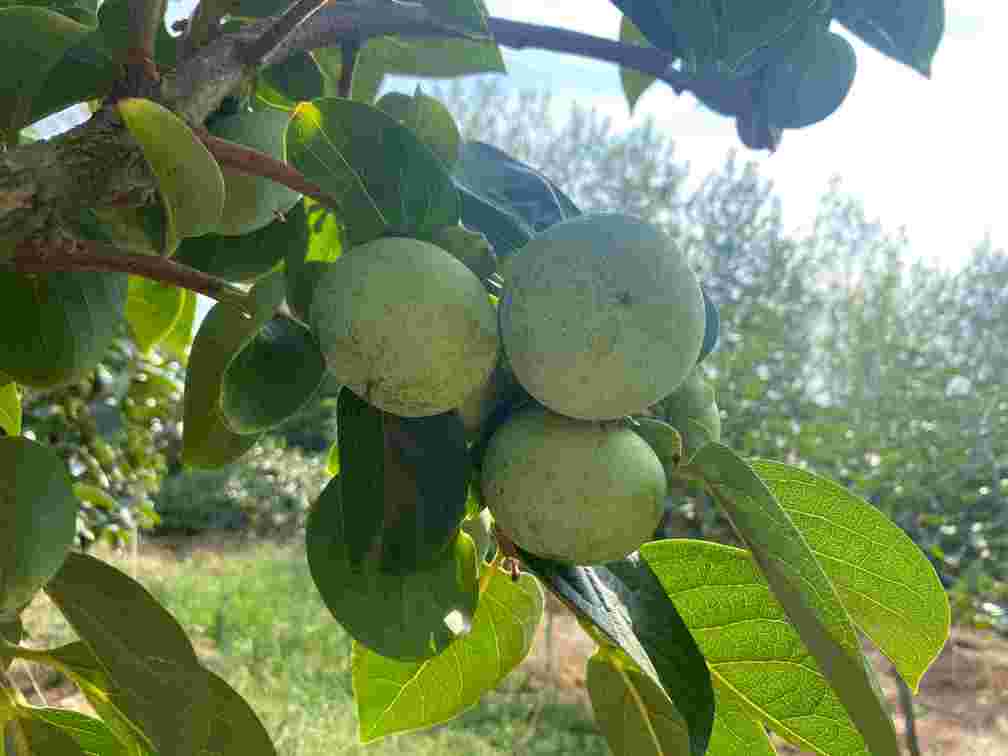

Supplement: Supplementary file 1 [file DataSheet1.zip › 2022-07-22 183454_20220722_183722.jpg]

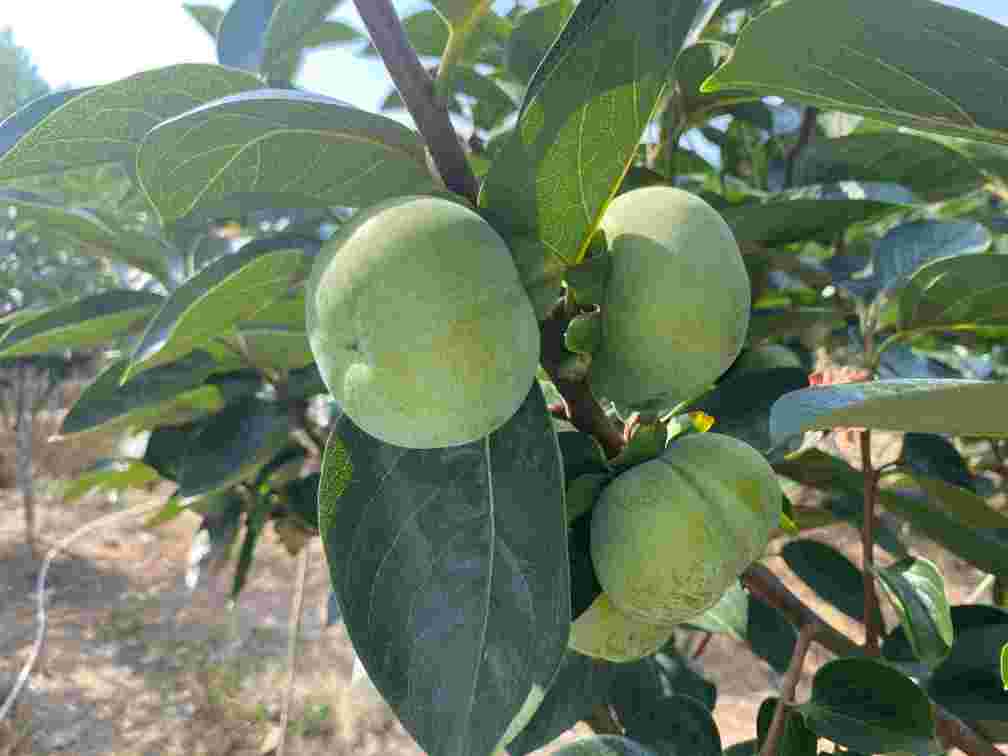

Supplement: Supplementary file 1 [file DataSheet1.zip › 2022-07-22 183454_20220722_183723.jpg]

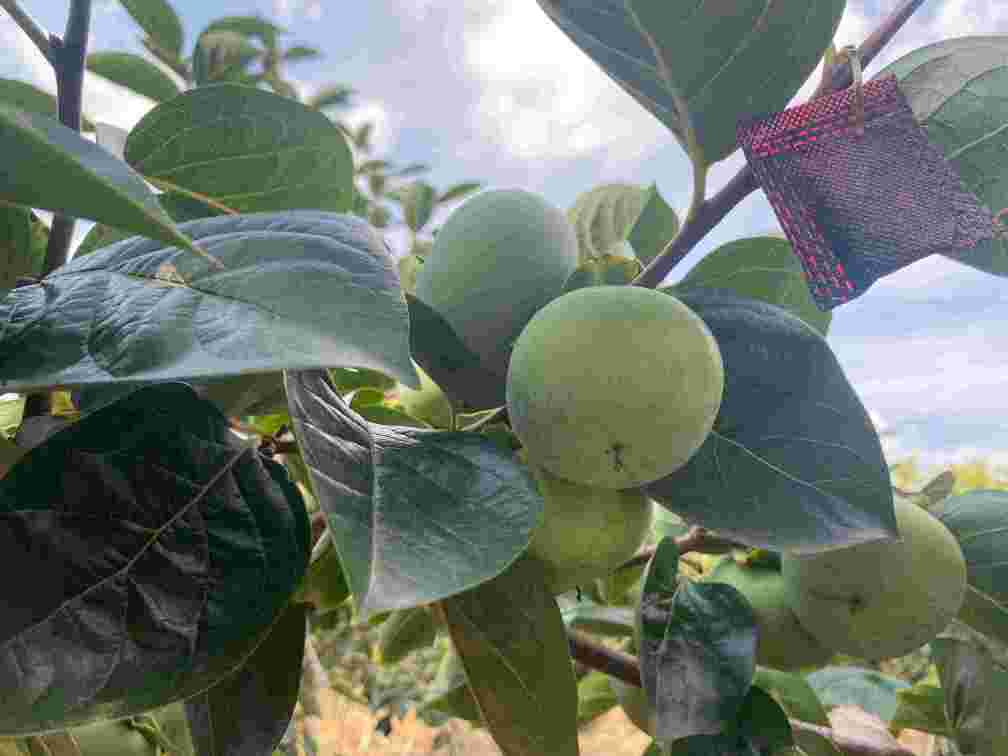

Supplement: Supplementary file 1 [file DataSheet1.zip › 2022-07-22 183454_20220722_183724.jpg]

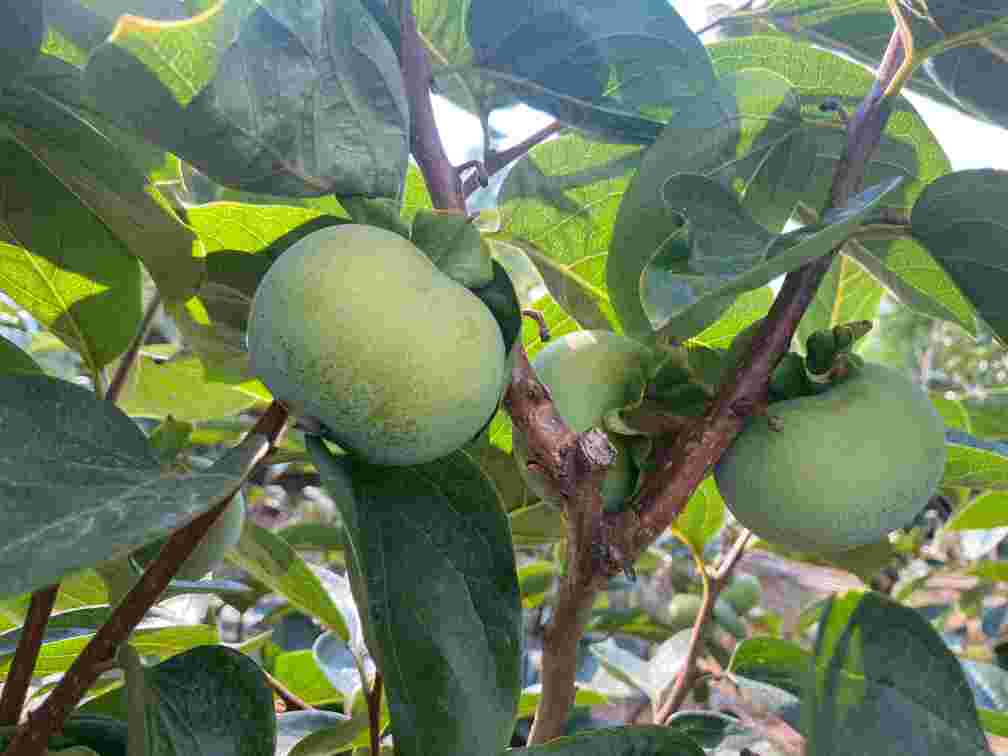

Supplement: Supplementary file 1 [file DataSheet1.zip › 2022-07-22 183454_20220722_183725.jpg]

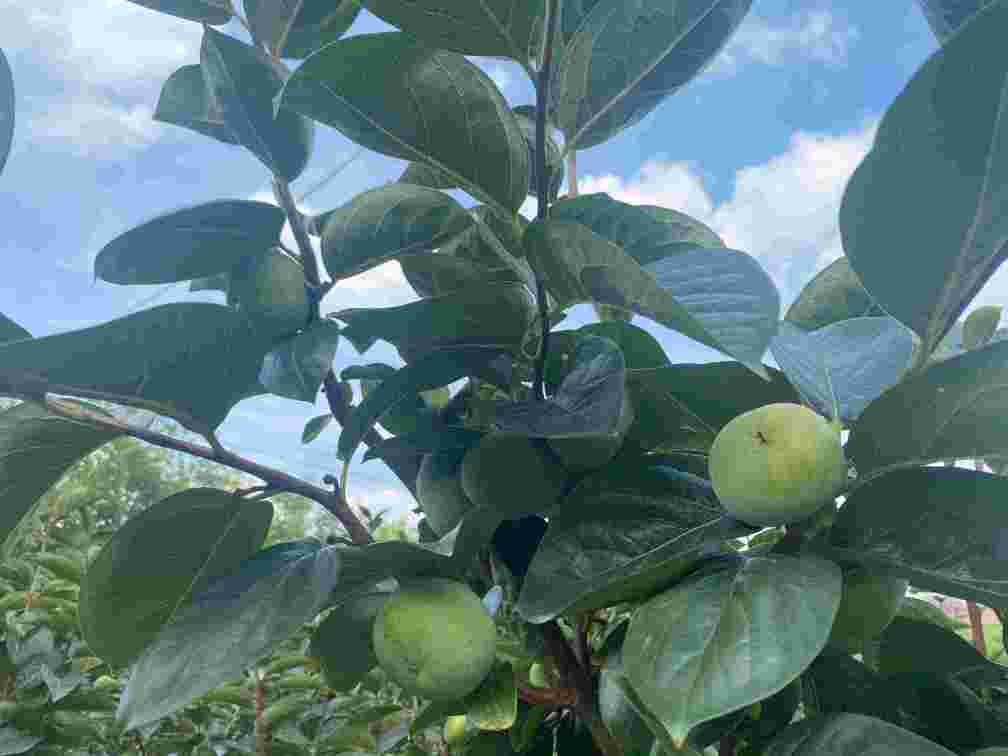

Supplement: Supplementary file 1 [file DataSheet1.zip › 2022-07-22 183454_20220722_183726.jpg]

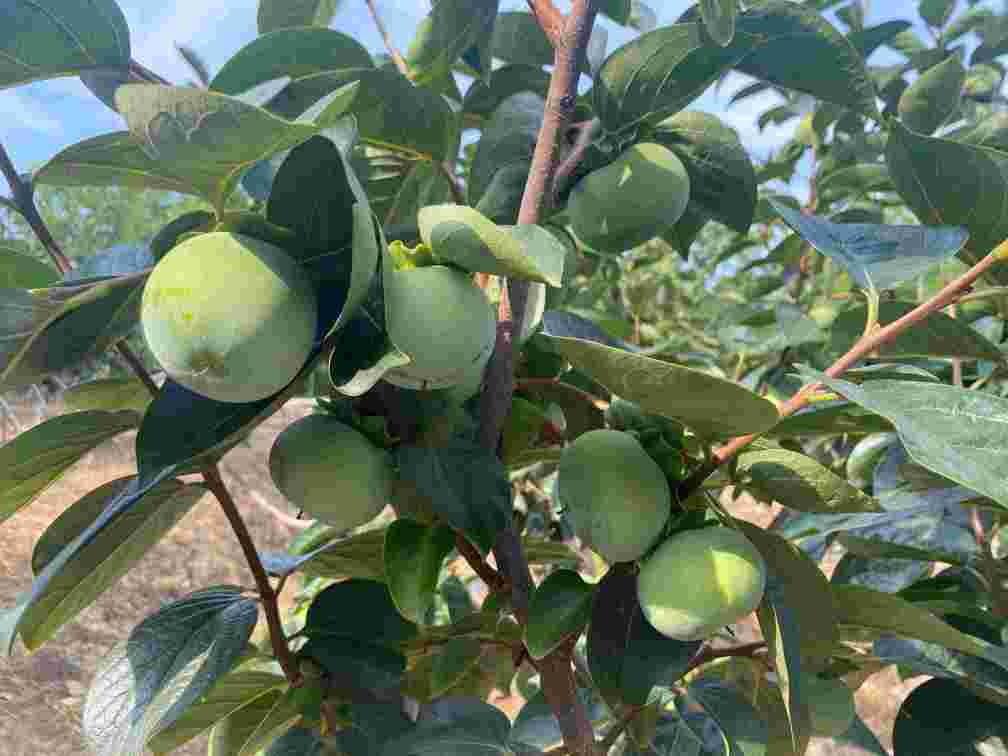

Supplement: Supplementary file 1 [file DataSheet1.zip › 2022-07-22 183454_20220722_183727.jpg]

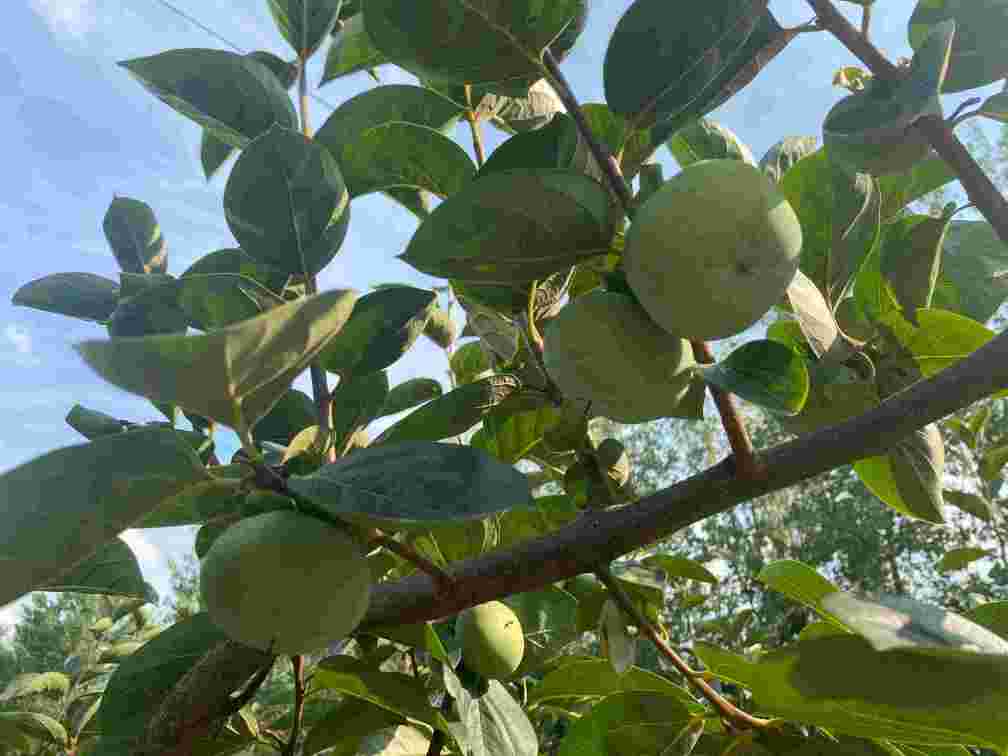

Supplement: Supplementary file 1 [file DataSheet1.zip › 2022-07-22 183454_20220722_183728.jpg]

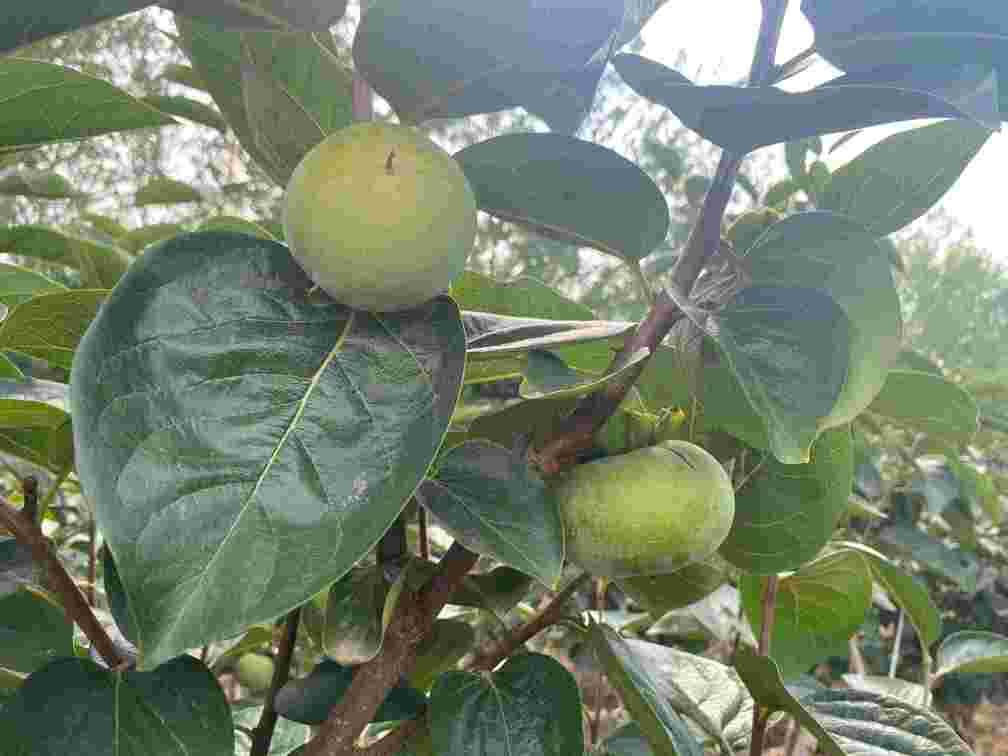

Supplement: Supplementary file 1 [file DataSheet1.zip › 2022-07-22 183454_20220722_183729.jpg]

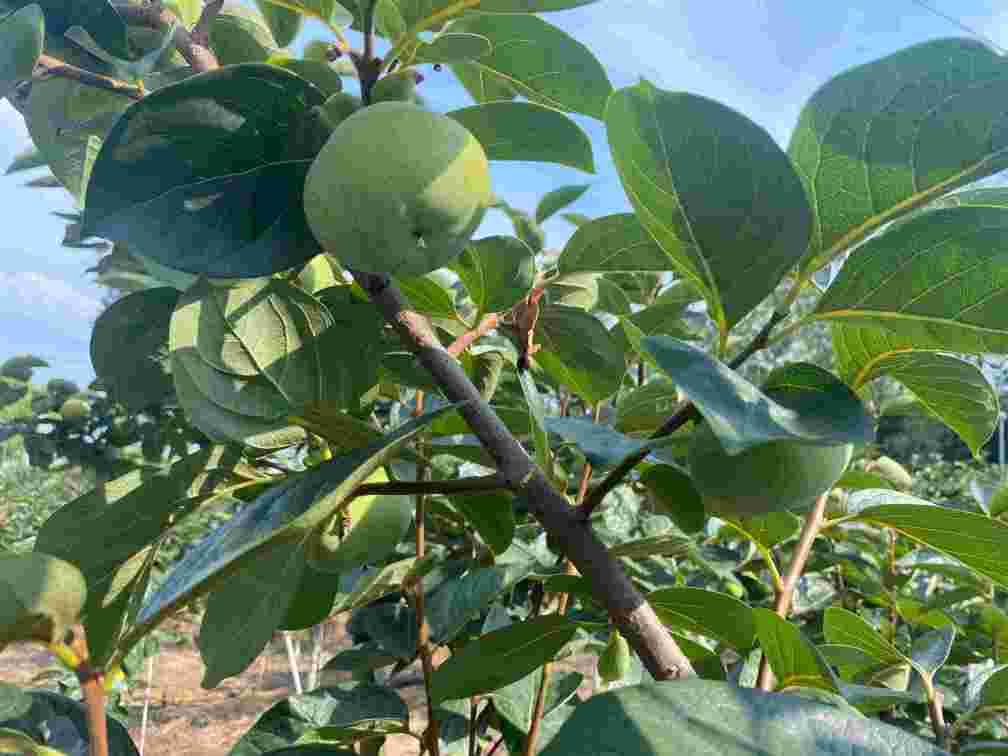

Supplement: Supplementary file 1 [file DataSheet1.zip › 2022-07-22 183454_20220722_183730.jpg]

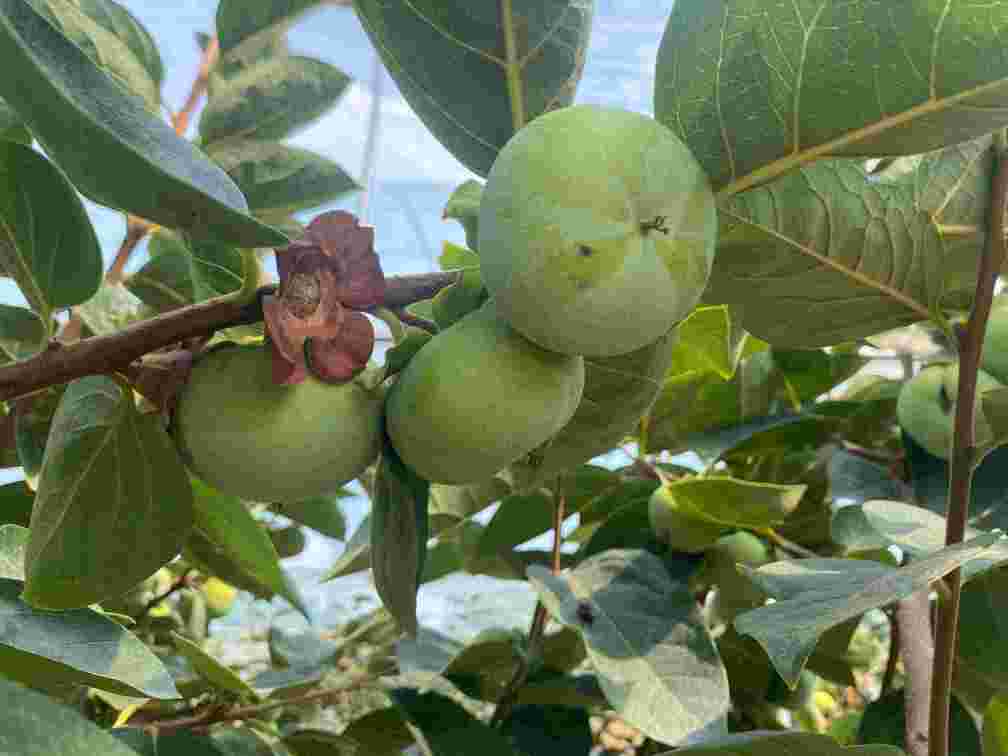

Supplement: Supplementary file 1 [file DataSheet1.zip › 2022-07-22 183454_20220722_183731.jpg]

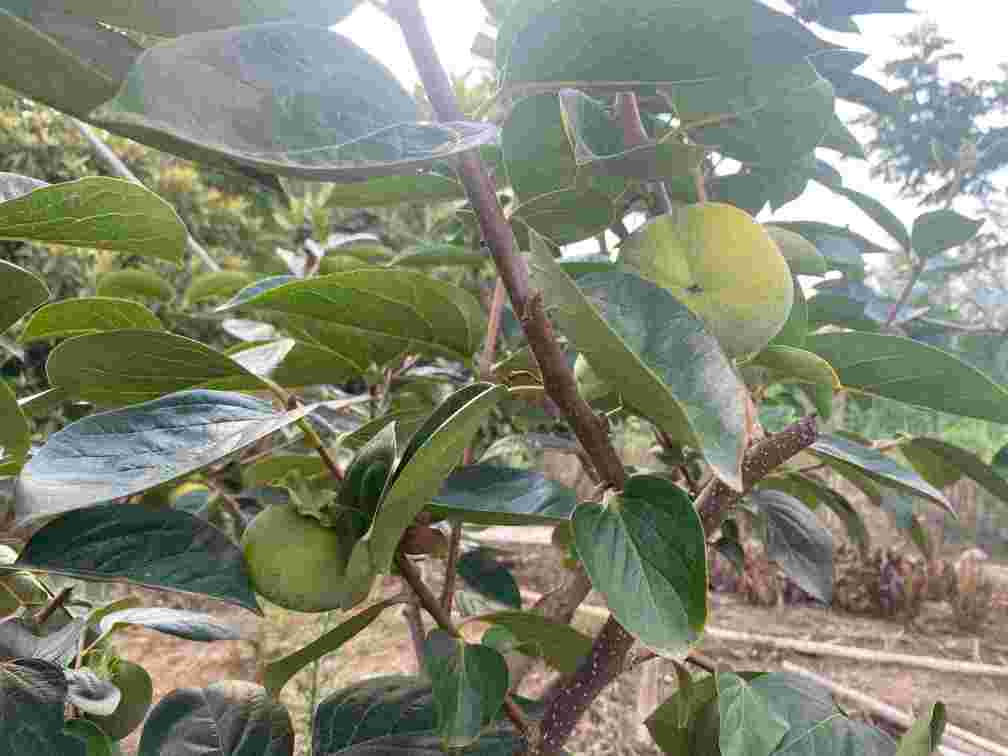

Supplement: Supplementary file 1 [file DataSheet1.zip › 2022-07-22 183454_20220722_183732.jpg]

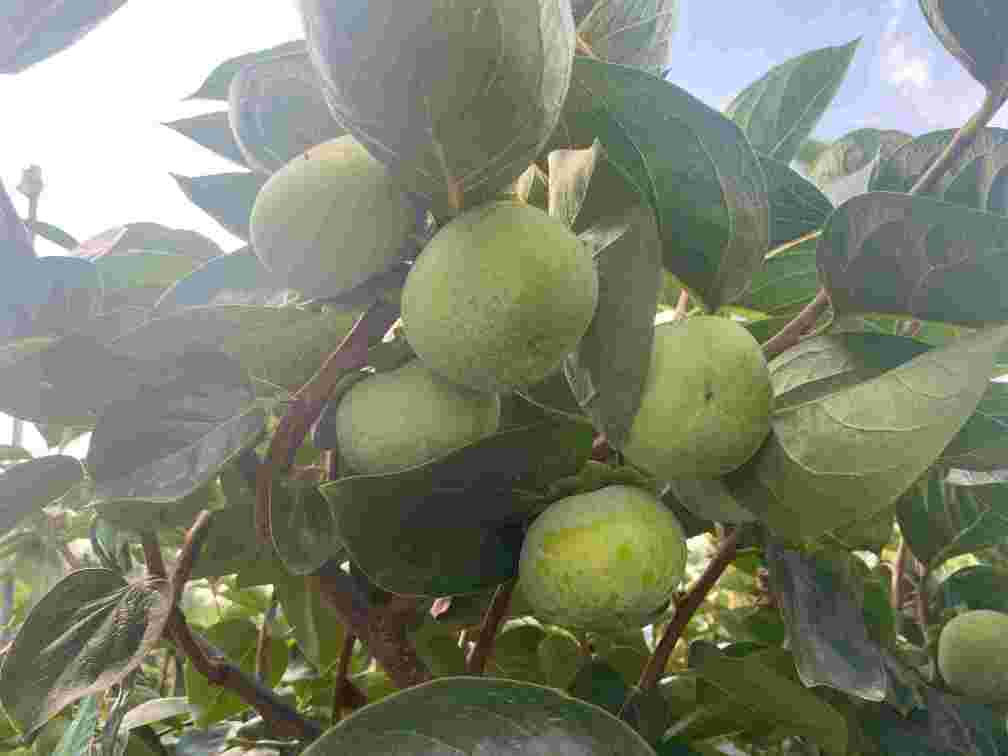

Supplement: Supplementary file 1 [file DataSheet1.zip › 2022-07-22 183454_20220722_183733.jpg]

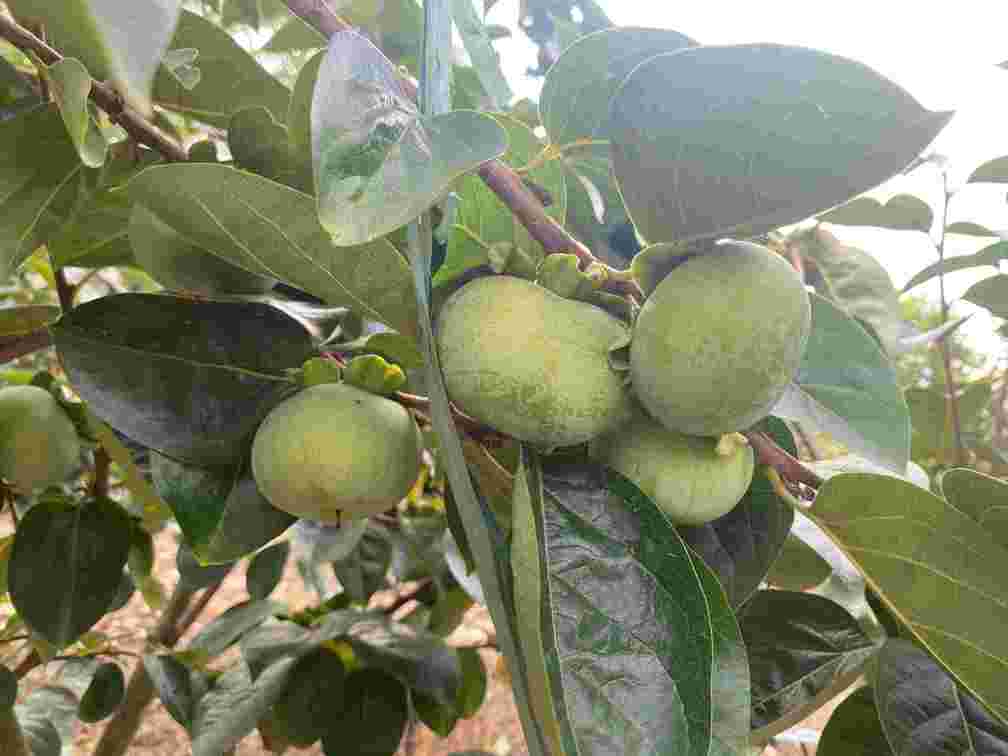

Supplement: Supplementary file 1 [file DataSheet1.zip › 2022-07-22 183454_20220722_183734.jpg]

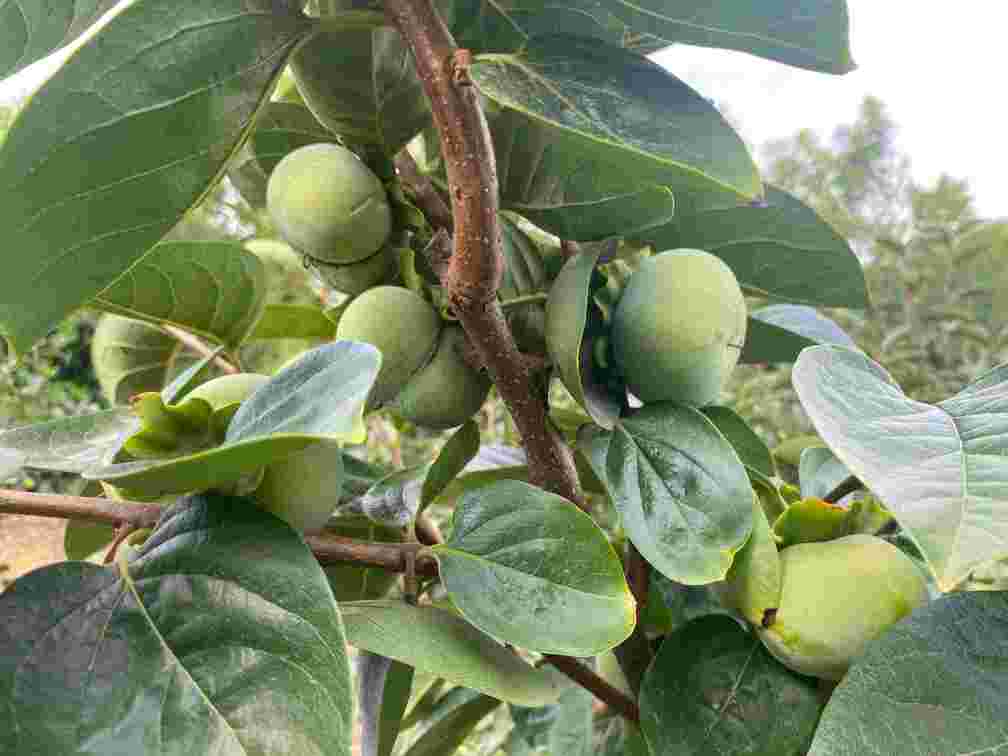

Supplement: Supplementary file 1 [file DataSheet1.zip › 2022-07-22 183454_20220722_183735.jpg]

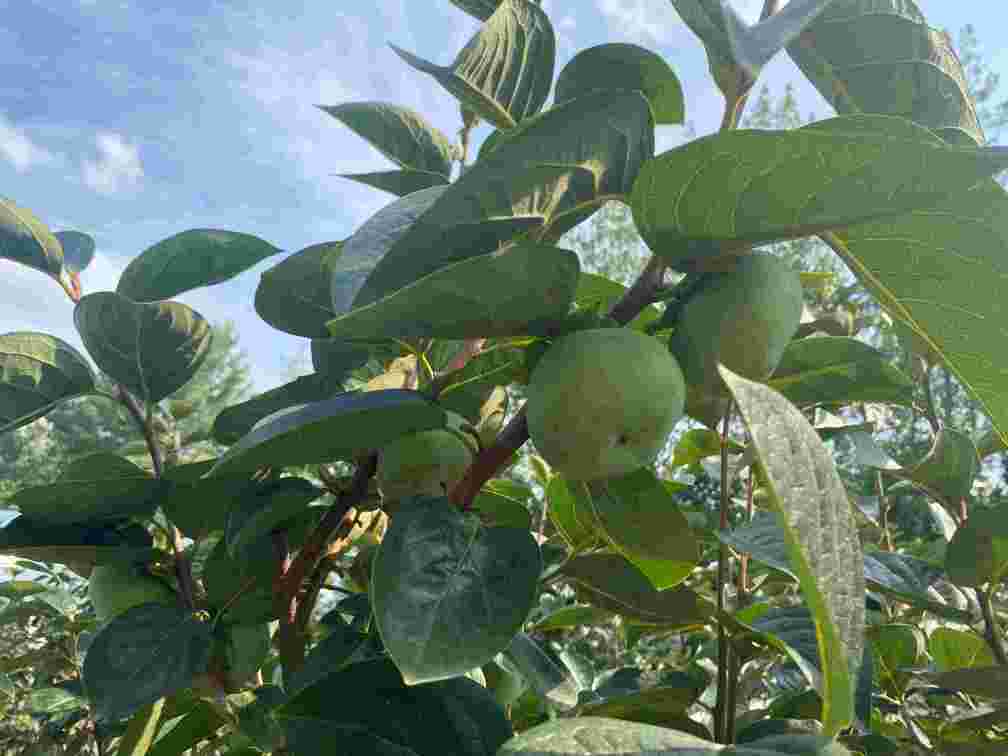

Supplement: Supplementary file 1 [file DataSheet1.zip › 2022-07-22 183454_20220722_183736.jpg]

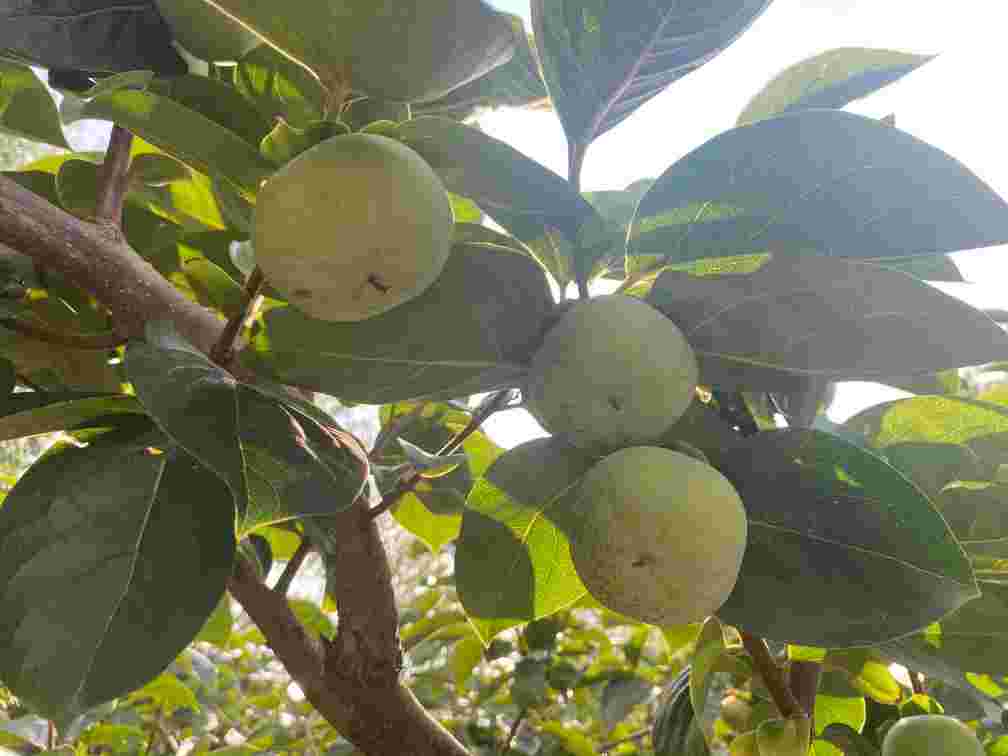

Supplement: Supplementary file 1 [file DataSheet1.zip › 2022-07-22 183454_20220722_183737.jpg]

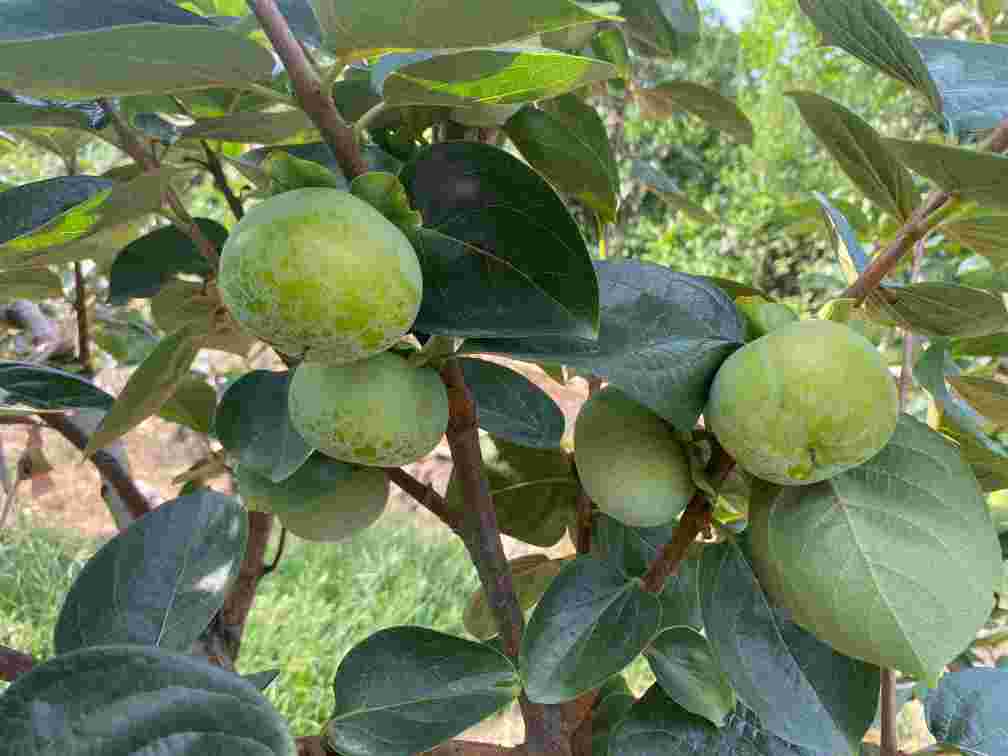

Supplement: Supplementary file 1 [file DataSheet1.zip › 2022-07-22 183454_20220722_183738.jpg]

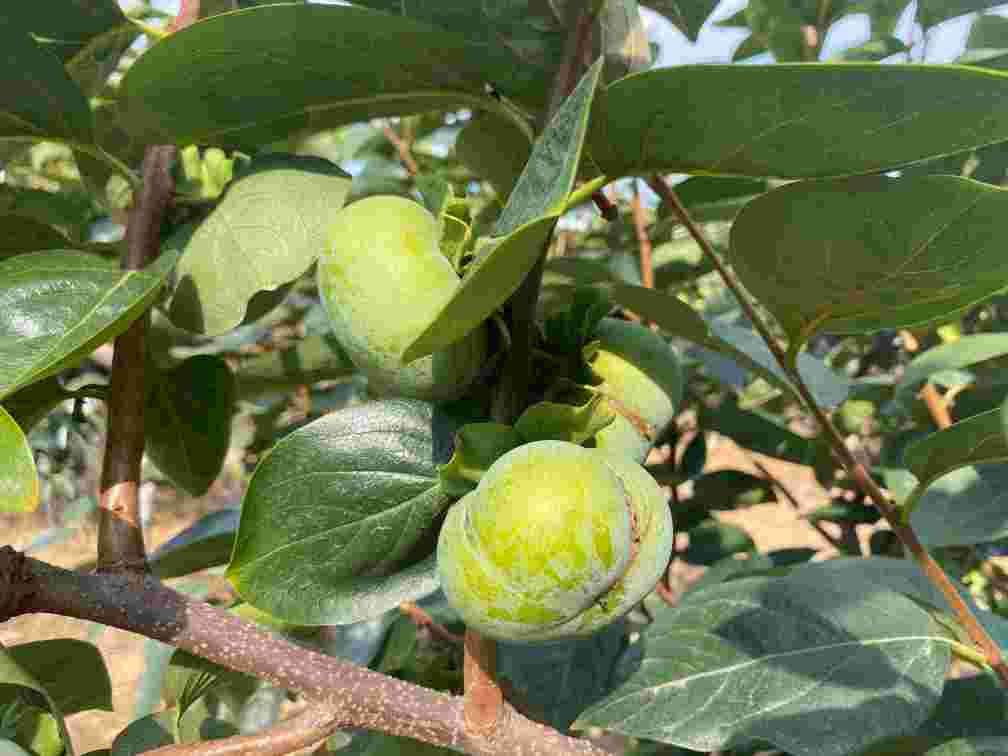

Supplement: Supplementary file 1 [file DataSheet1.zip › 2022-07-22 183454_20220722_183739.jpg]

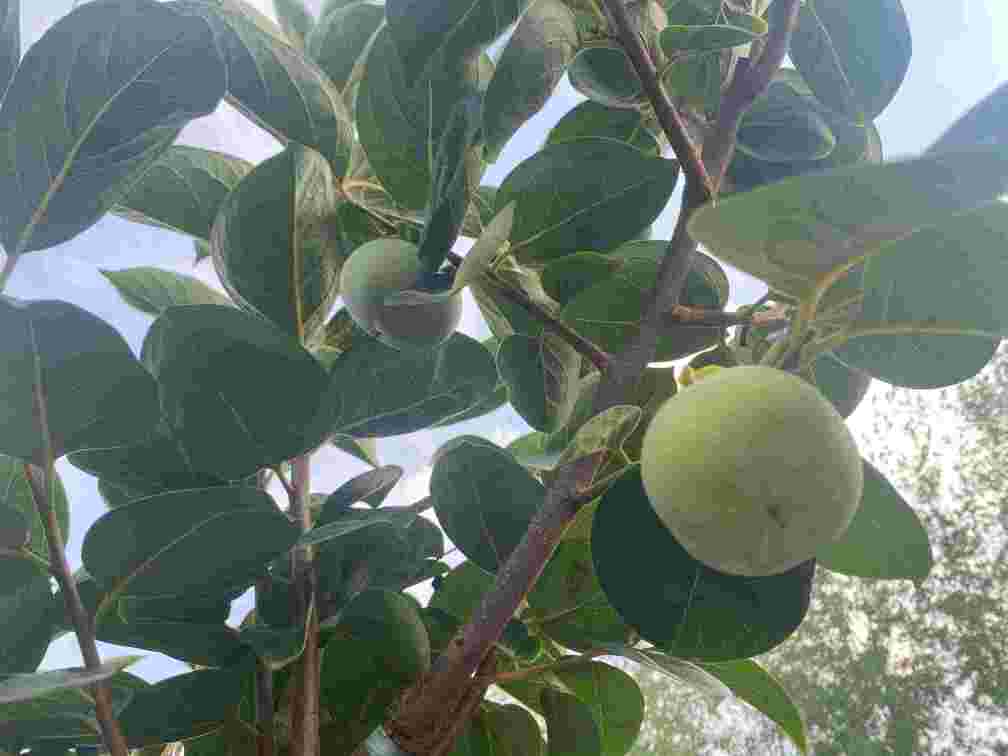

Supplement: Supplementary file 1 [file DataSheet1.zip › 2022-07-22 183454_20220722_183740.jpg]

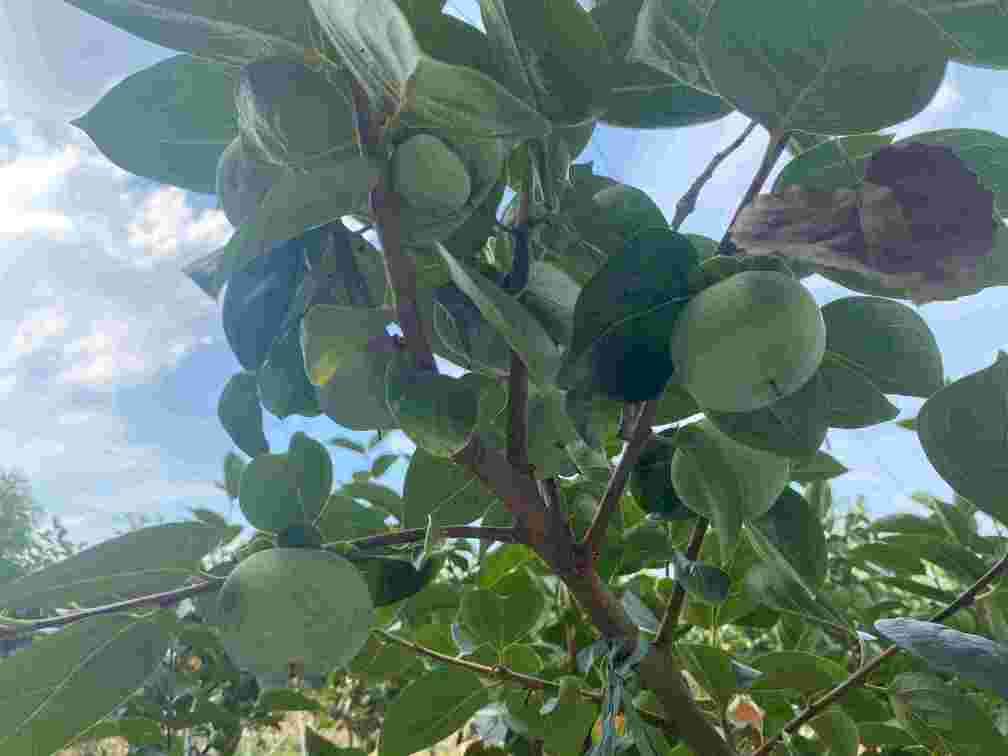

Supplement: Supplementary file 1 [file DataSheet1.zip › 2022-07-22 183454_20220722_183741.jpg]

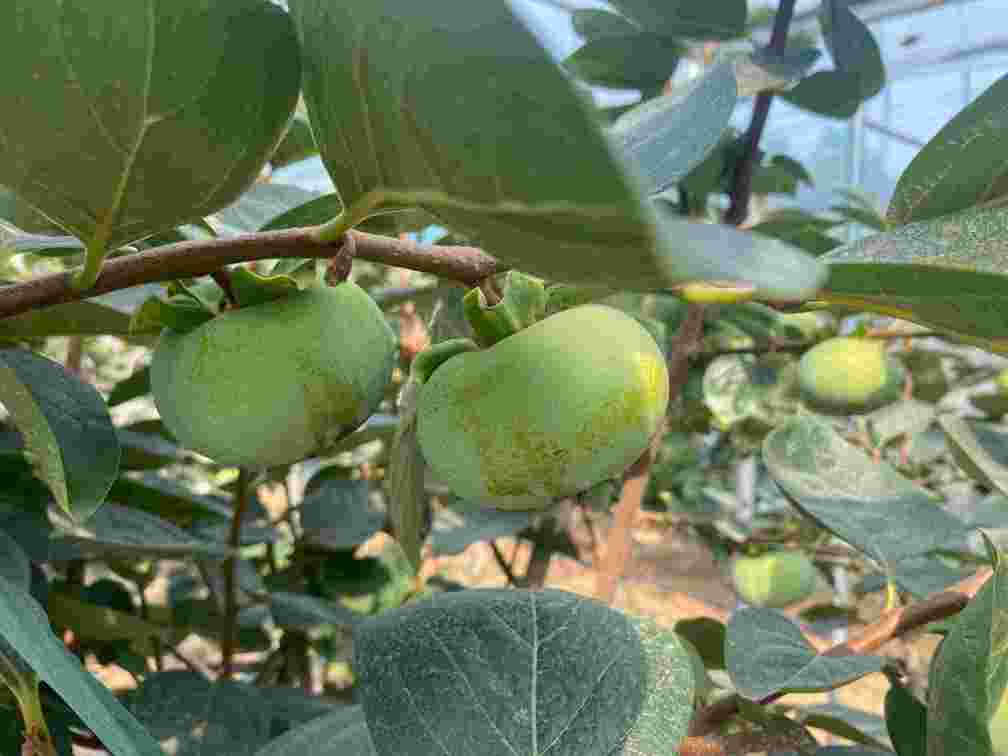

Supplement: Supplementary file 1 [file DataSheet1.zip › 2022-07-22 183454_20220722_183742.jpg]

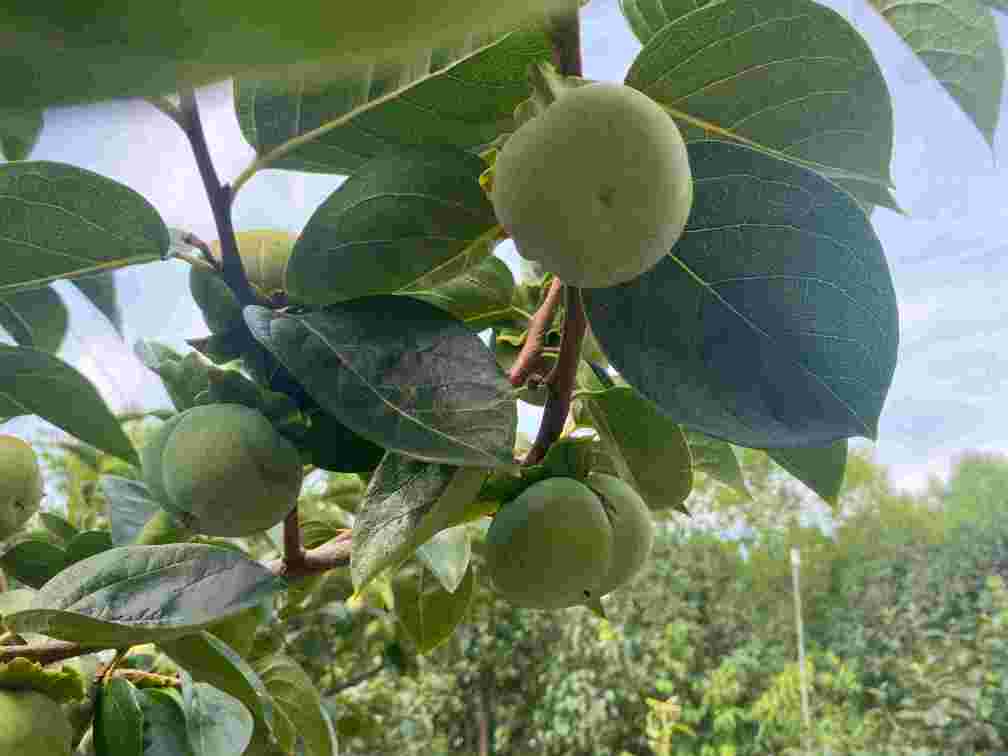

Supplement: Supplementary file 1 [file DataSheet1.zip › 2022-07-22 183454_20220722_183743.jpg]

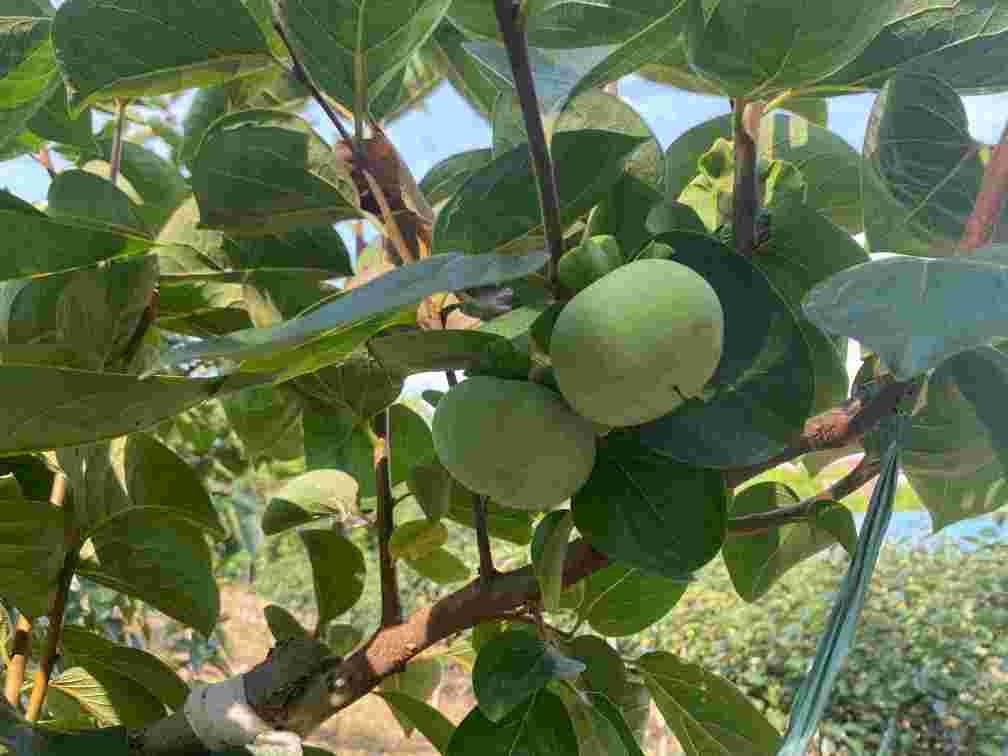

Supplement: Supplementary file 1 [file DataSheet1.zip › 2022-07-22 183454_20220722_183744.jpg]

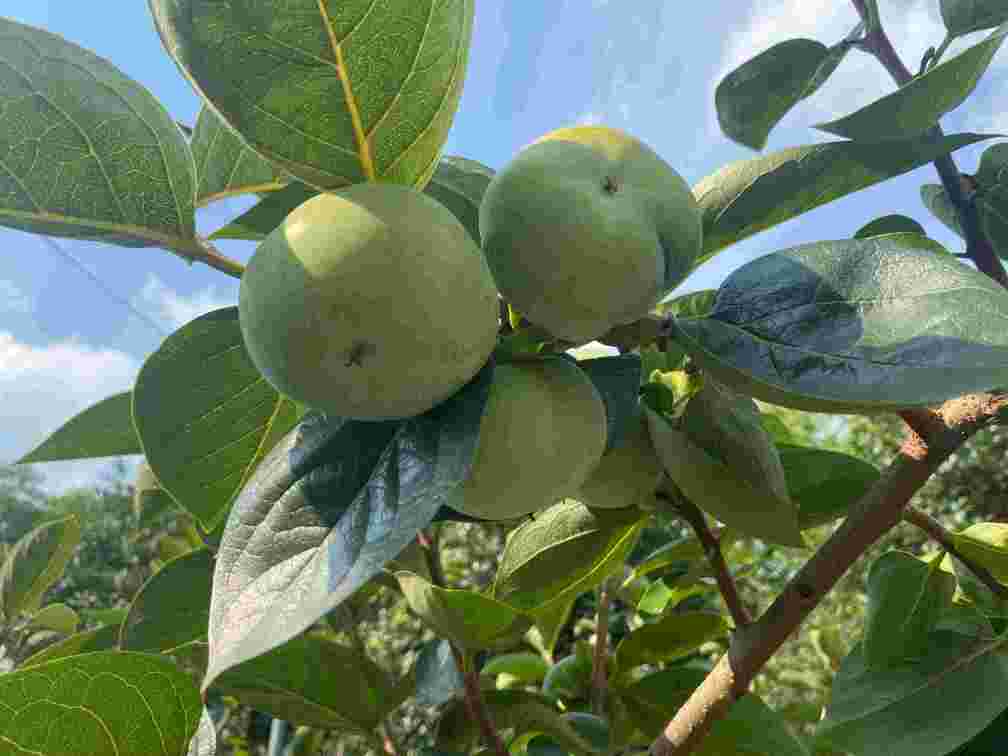

Supplement: Supplementary file 1 [file DataSheet1.zip › 2022-07-22 183454_20220722_183745.jpg]

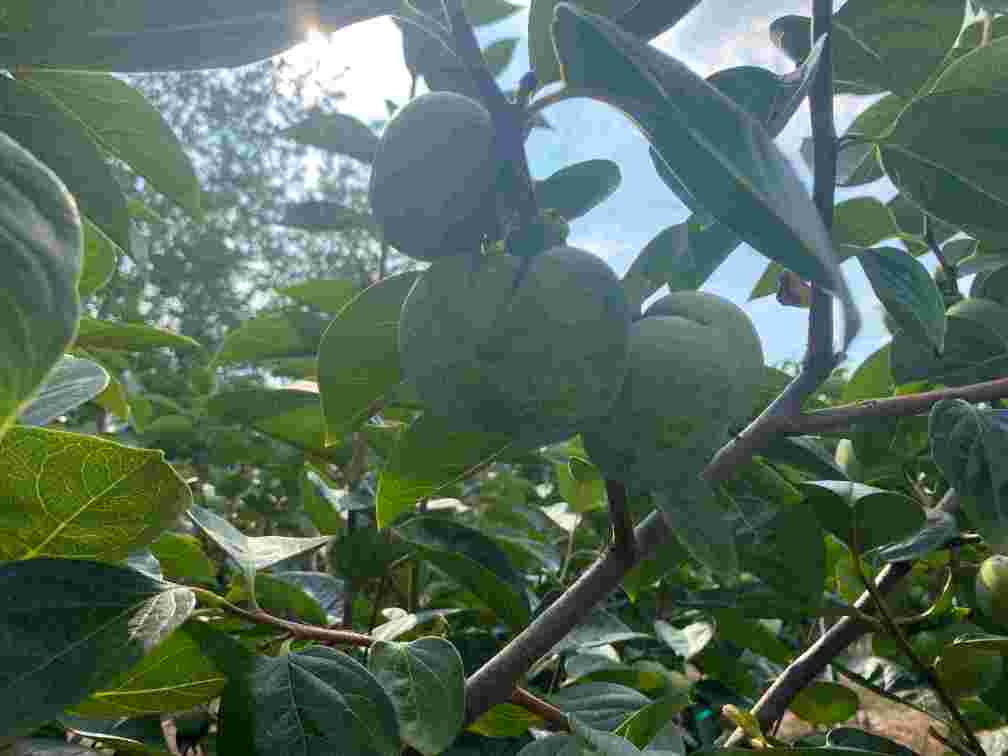

Supplement: Supplementary file 1 [file DataSheet1.zip › 2022-07-22 183454_20220722_183747.jpg]

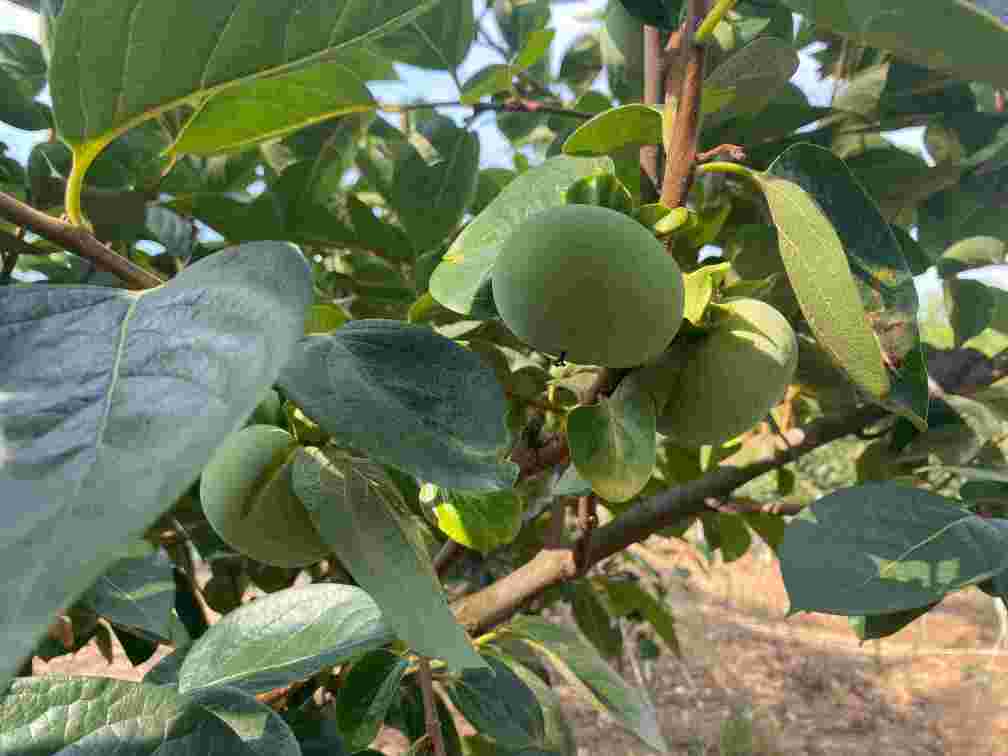

Supplement: Supplementary file 1 [file DataSheet1.zip › 2022-07-22 183454_20220722_183748.jpg]

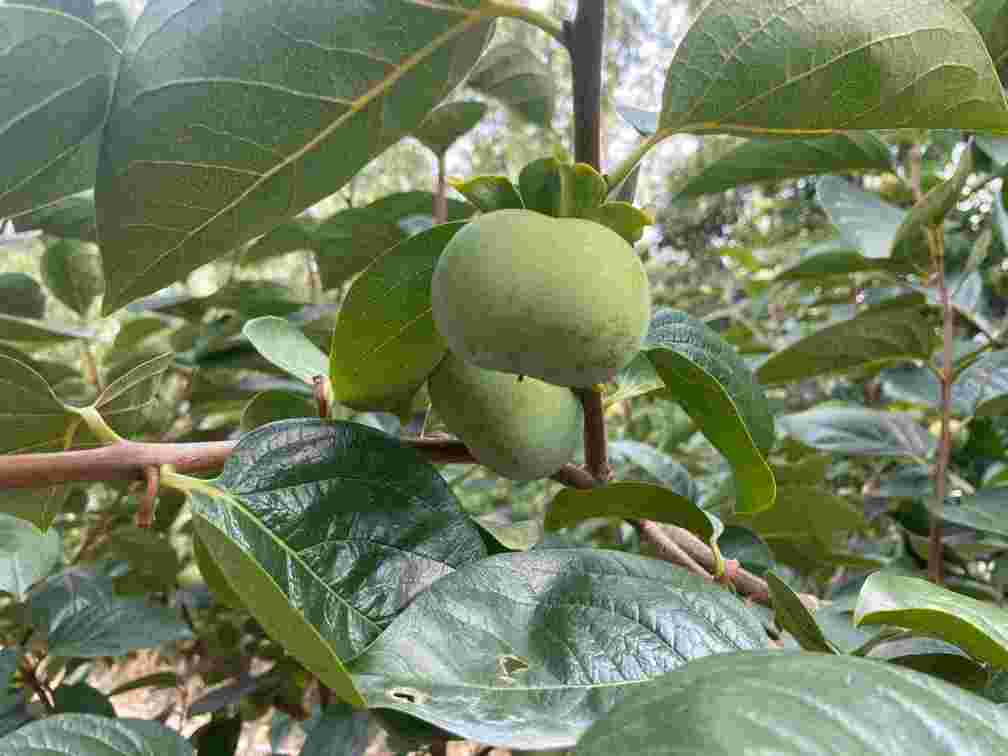

Supplement: Supplementary file 1 [file DataSheet1.zip › 2022-07-22 183454_20220722_183749.jpg]

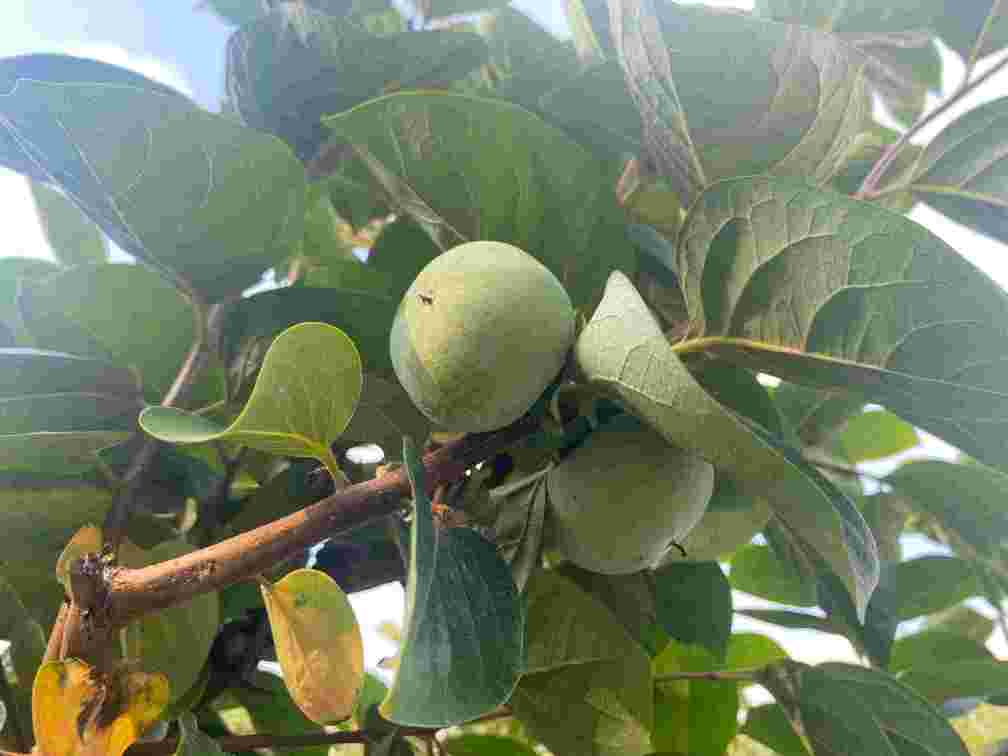

Supplement: Supplementary file 1 [file DataSheet1.zip › 2022-07-22 183454_20220722_183750.jpg]

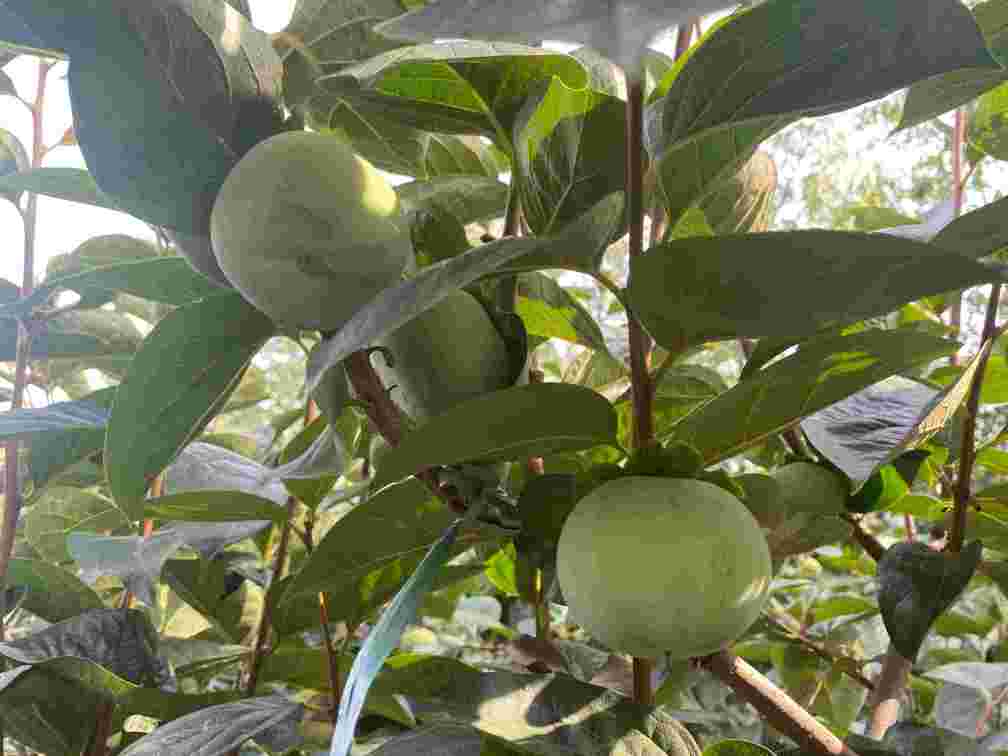

Supplement: Supplementary file 1 [file DataSheet1.zip › 2022-07-22 183454_20220722_183751.jpg]

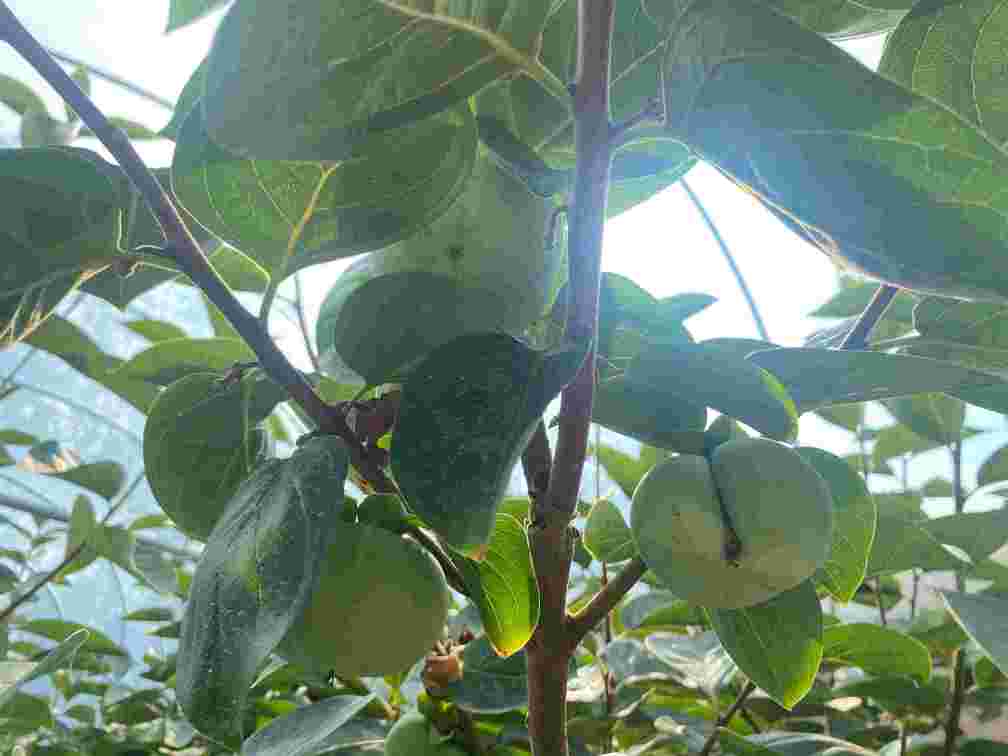

Supplement: Supplementary file 1 [file DataSheet1.zip › 2022-07-22 183454_20220722_183752.jpg]

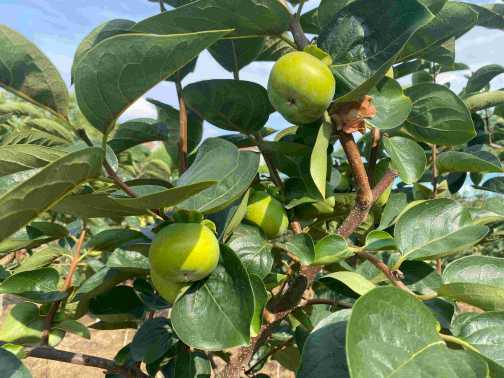

Supplement: Supplementary file 1 [file DataSheet1.zip › 2022-07-22 183454_20220722_183753.jpg]

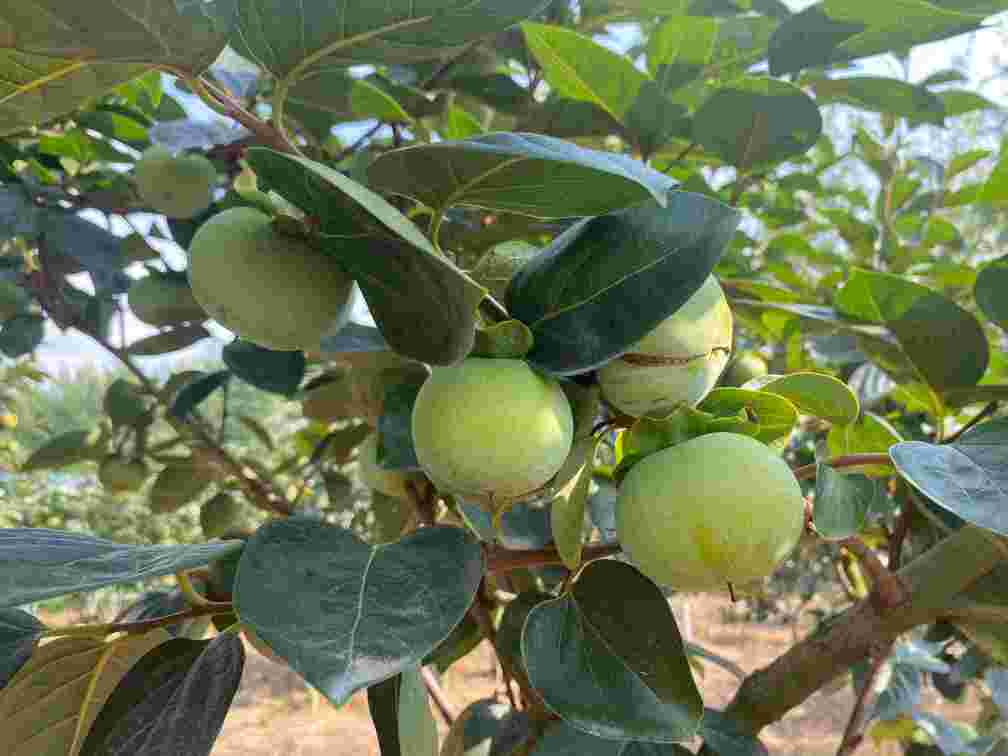

Supplement: Supplementary file 1 [file DataSheet1.zip › 2022-07-22 183454_20220722_183755.jpg]

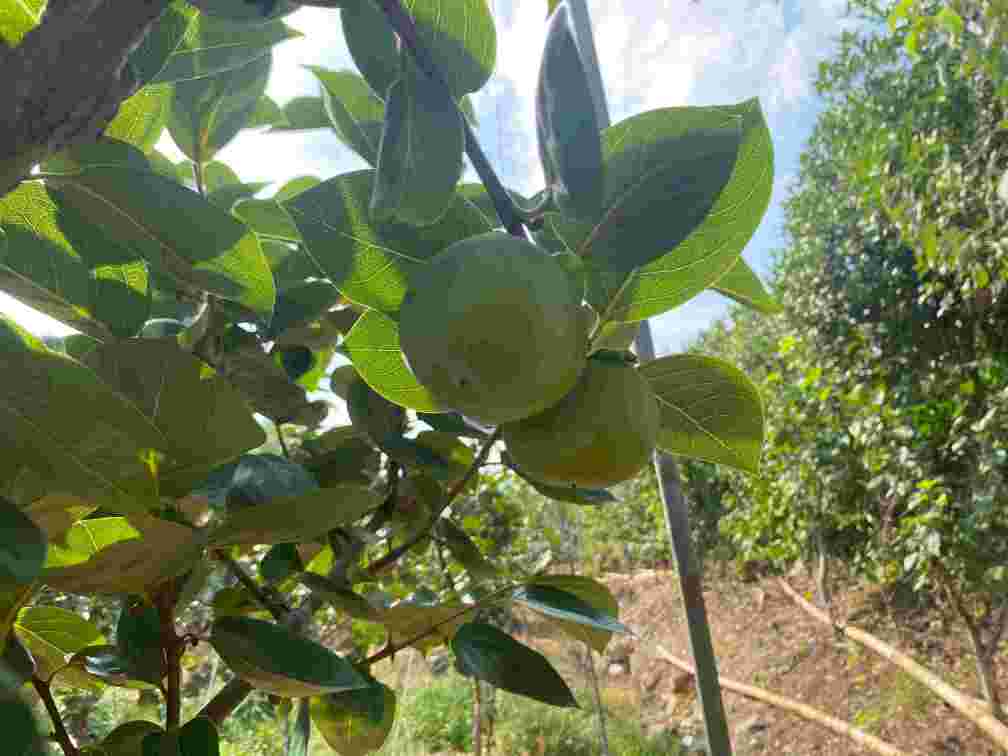

Supplement: Supplementary file 1 [file DataSheet1.zip › 2022-07-22 183454_20220722_183756.jpg]

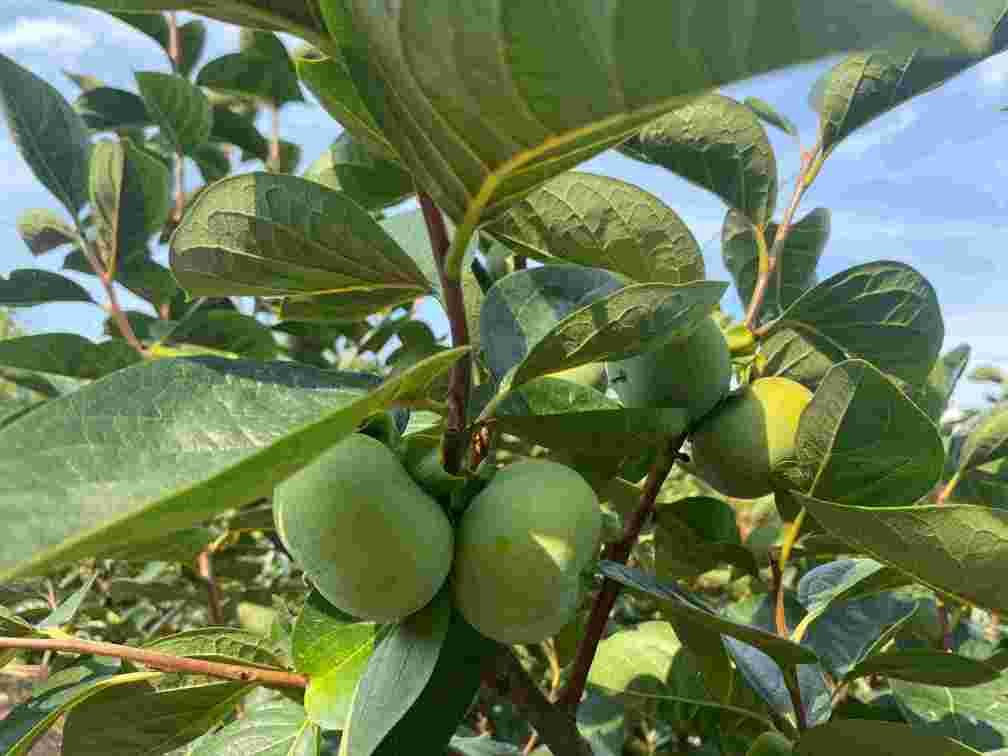

Supplement: Supplementary file 1 [file DataSheet1.zip › 2022-07-22 183454_20220722_183757.jpg]

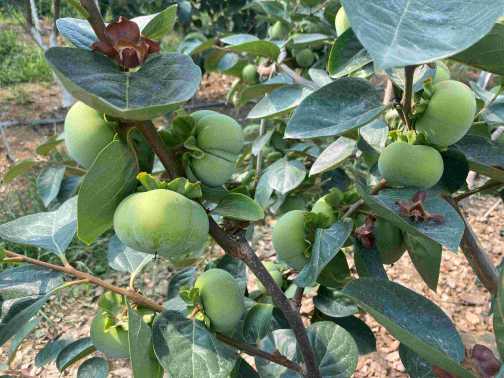

Supplement: Supplementary file 1 [file DataSheet1.zip › 2022-07-22 183454_20220722_183758.jpg]

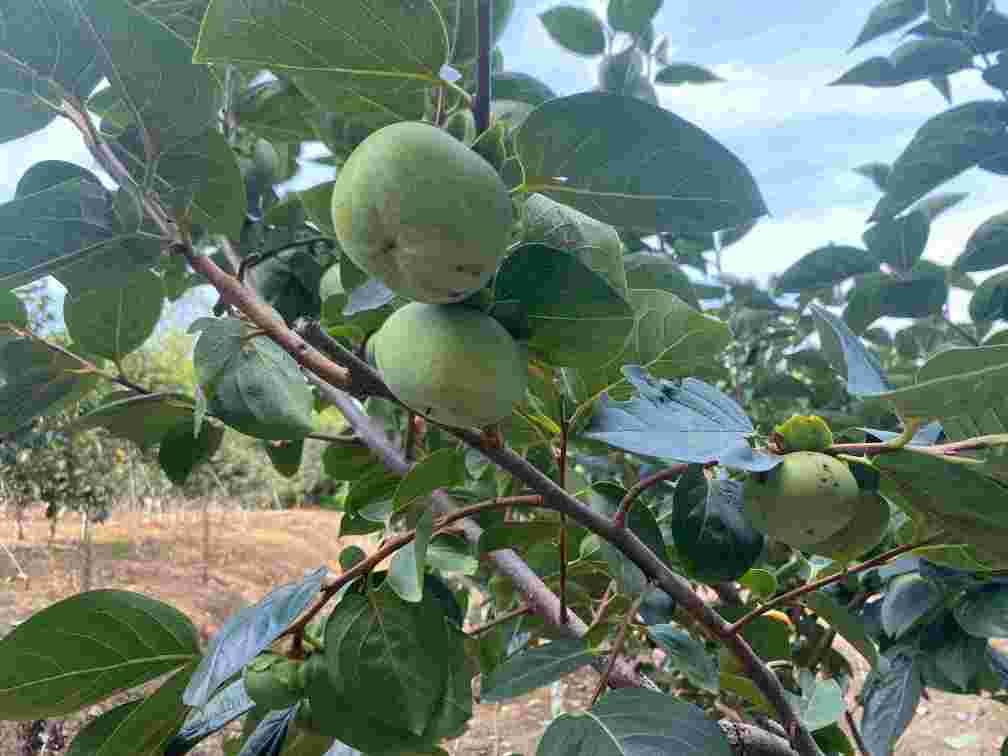

Supplement: Supplementary file 1 [file DataSheet1.zip › 2022-07-22 183454_20220722_183759.jpg]

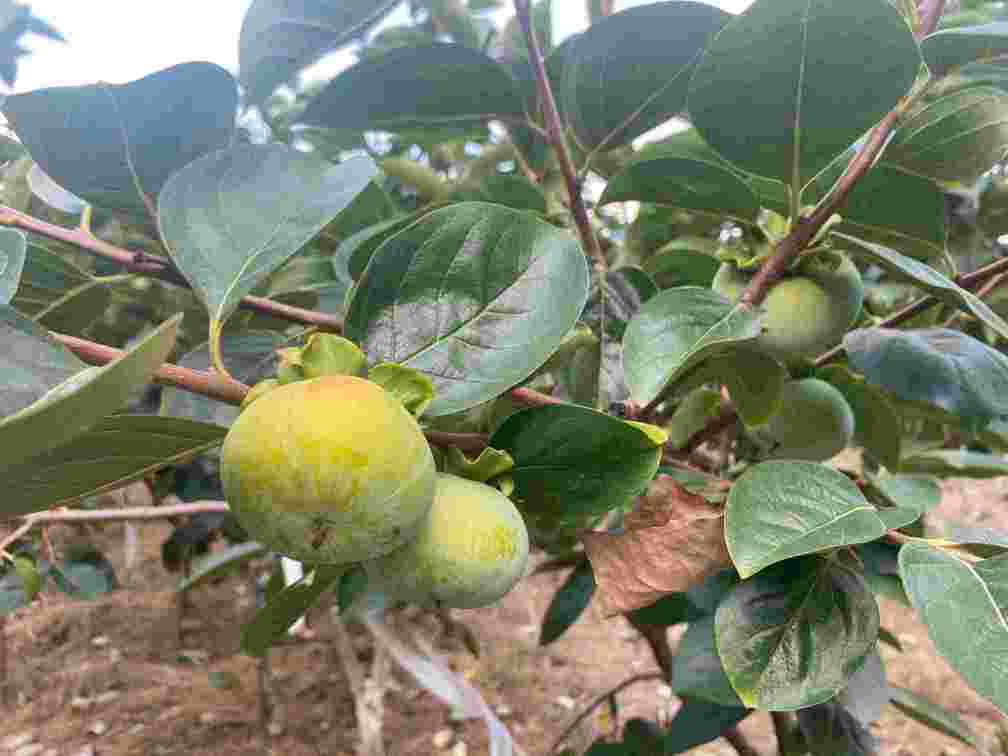

Supplement: Supplementary file 1 [file DataSheet1.zip › 2022-07-22 183454_20220722_183800.jpg]

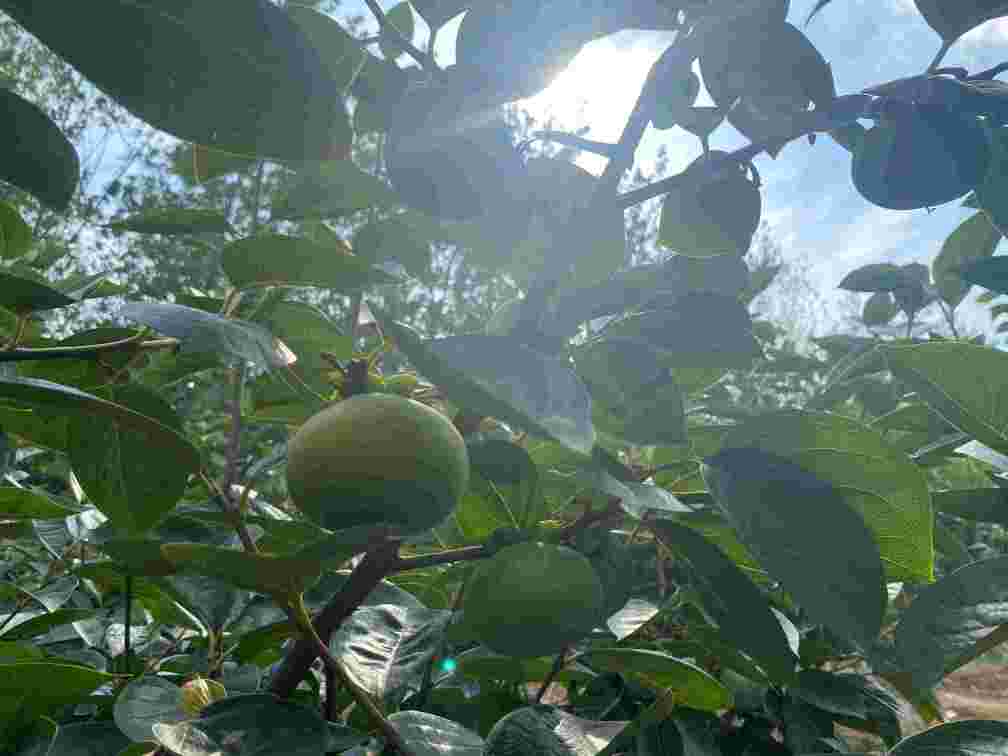

Supplement: Supplementary file 1 [file DataSheet1.zip › 2022-07-22 183454_20220722_183801.jpg]

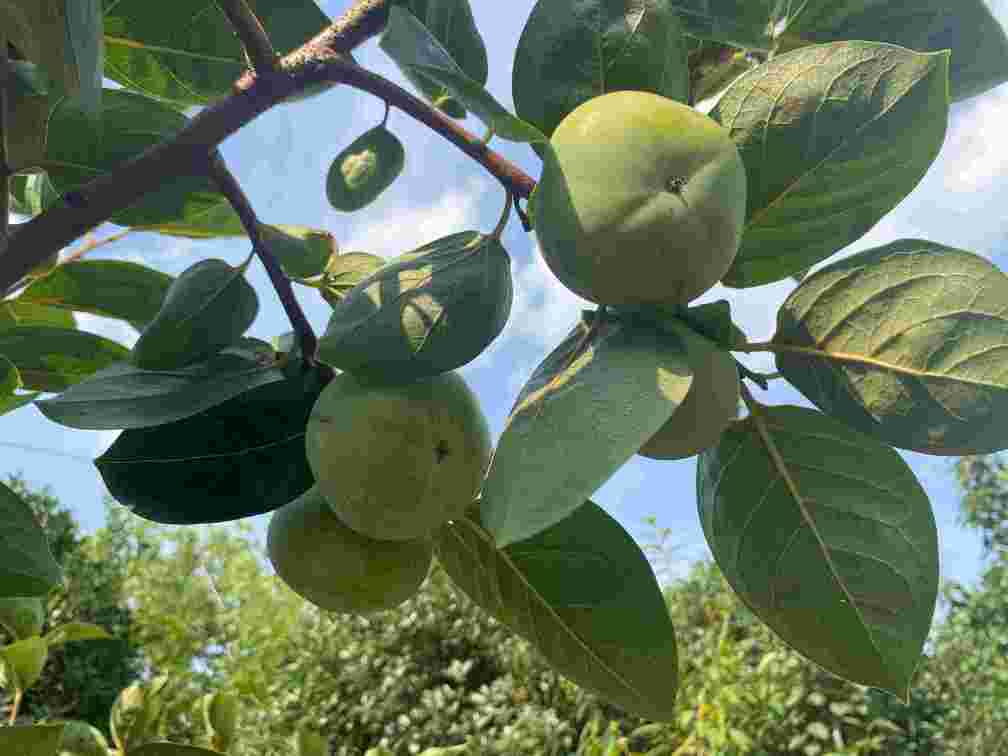

Supplement: Supplementary file 1 [file DataSheet1.zip › 2022-07-22 183454_20220722_183802.jpg]

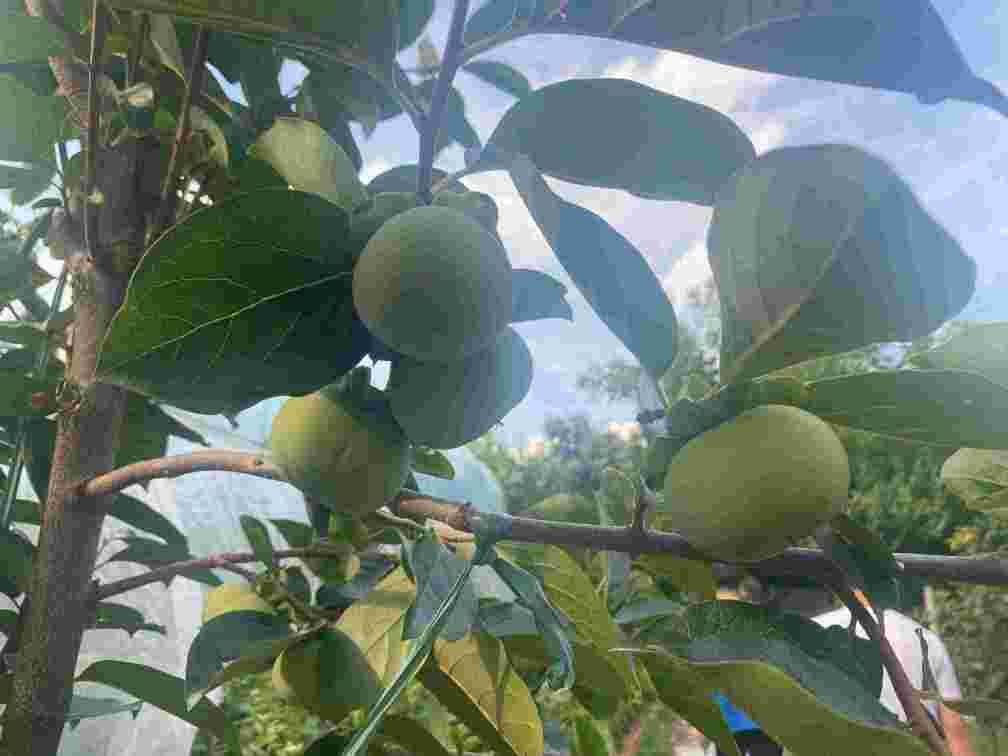

Supplement: Supplementary file 1 [file DataSheet1.zip › 2022-07-22 183454_20220722_183803.jpg]

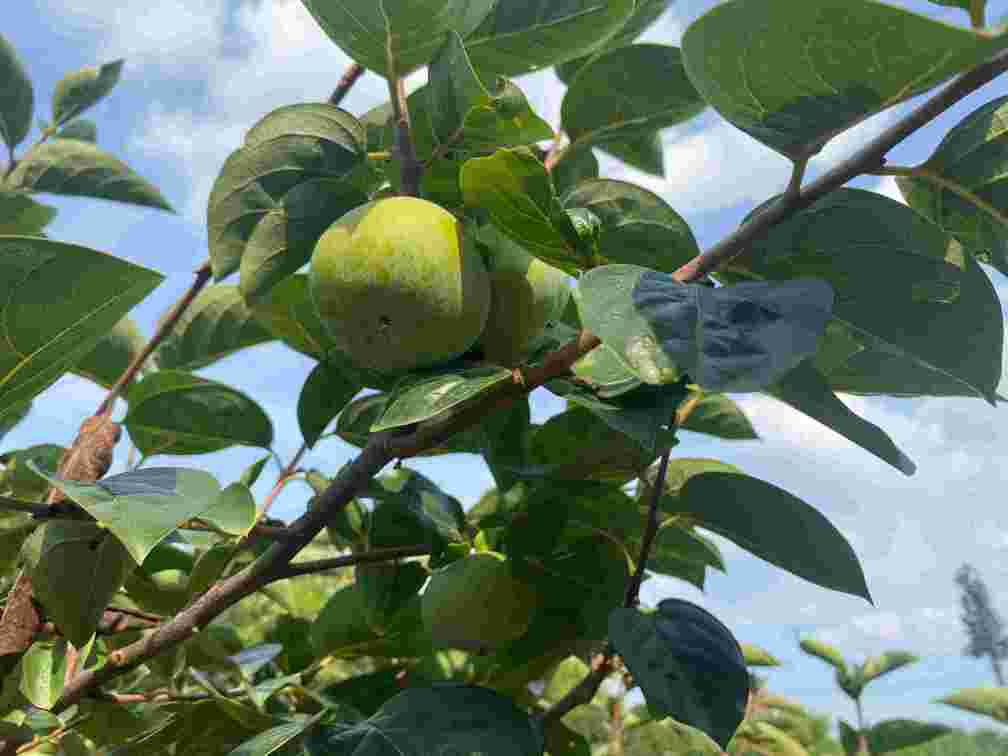

Supplement: Supplementary file 1 [file DataSheet1.zip › 2022-07-22 183454_20220722_183804.jpg]

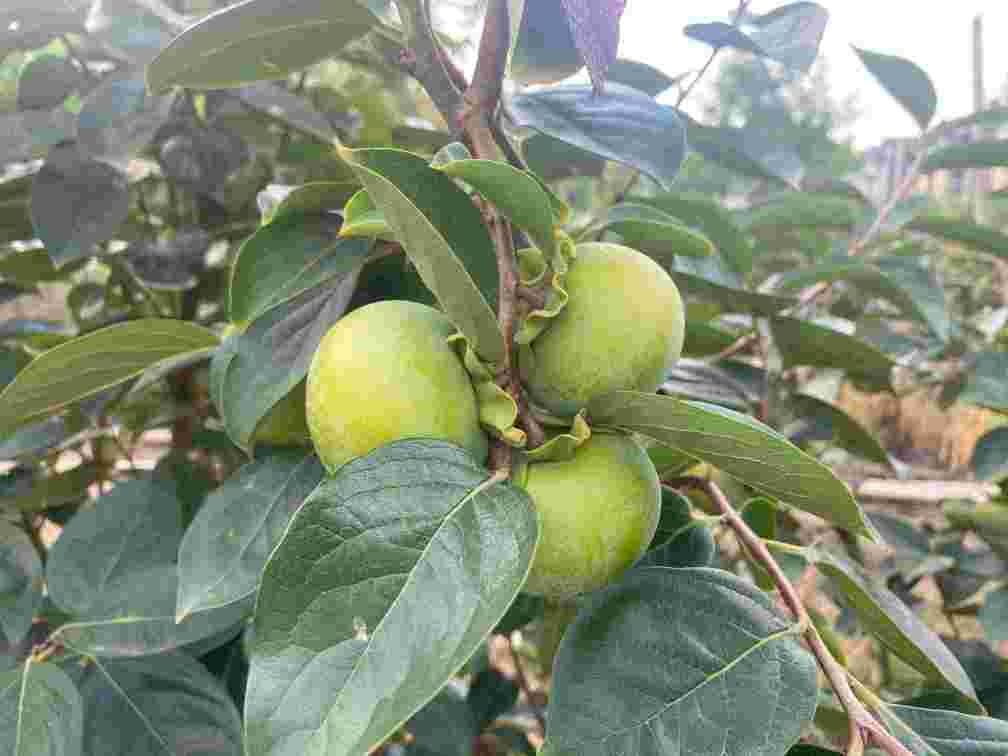

Supplement: Supplementary file 1 [file DataSheet1.zip › 2022-07-22 183454_20220722_183805.jpg]

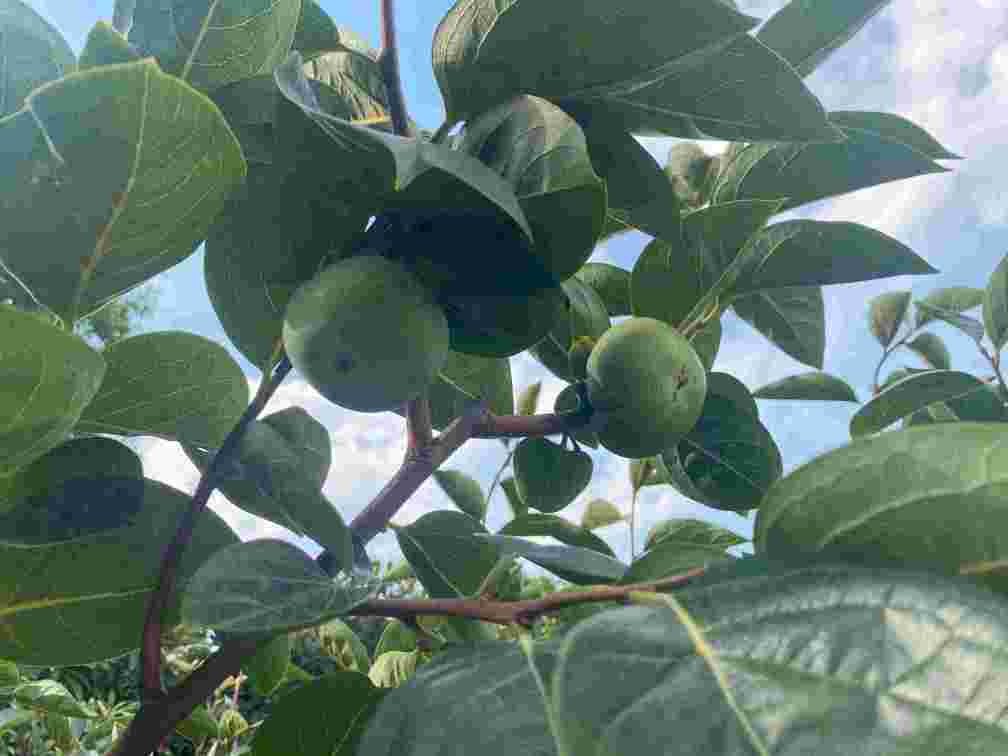

Supplement: Supplementary file 1 [file DataSheet1.zip › 2022-07-22 183454_20220722_183806.jpg]

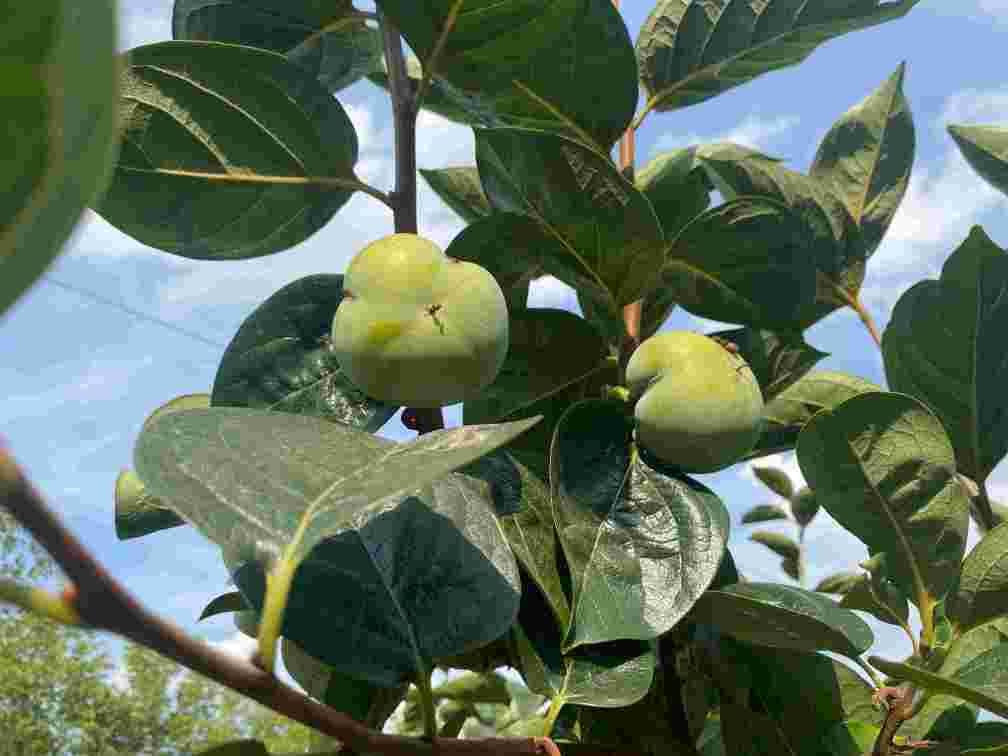

Supplement: Supplementary file 1 [file DataSheet1.zip › 2022-07-22 183454_20220722_183807.jpg]

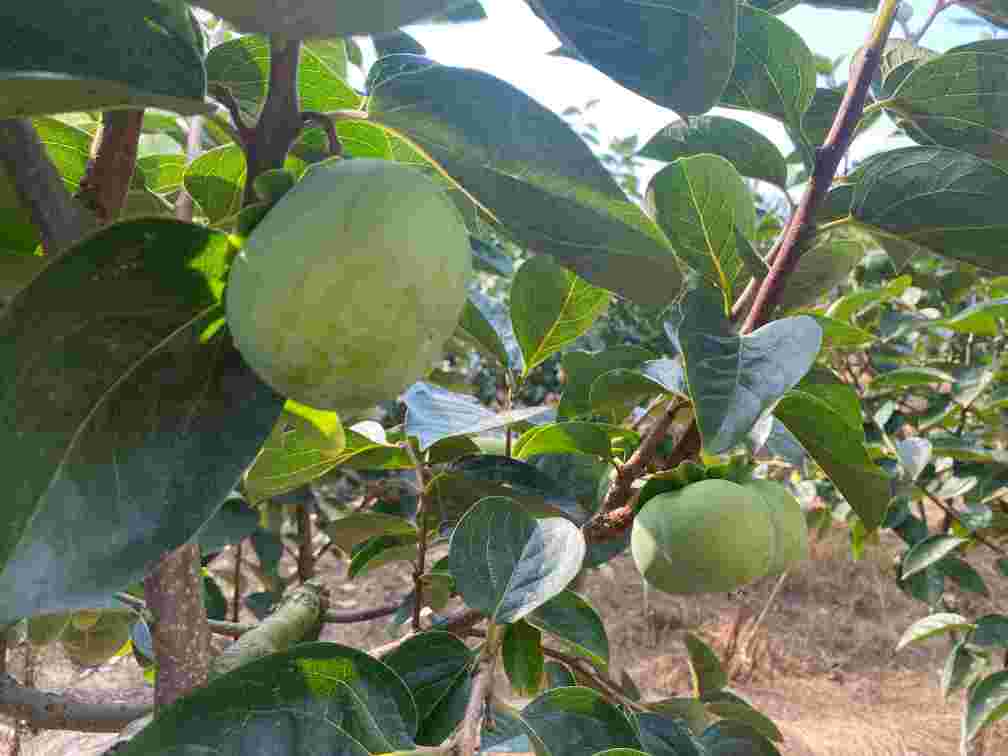

Supplement: Supplementary file 1 [file DataSheet1.zip › 2022-07-22 183454_20220722_183808.jpg]

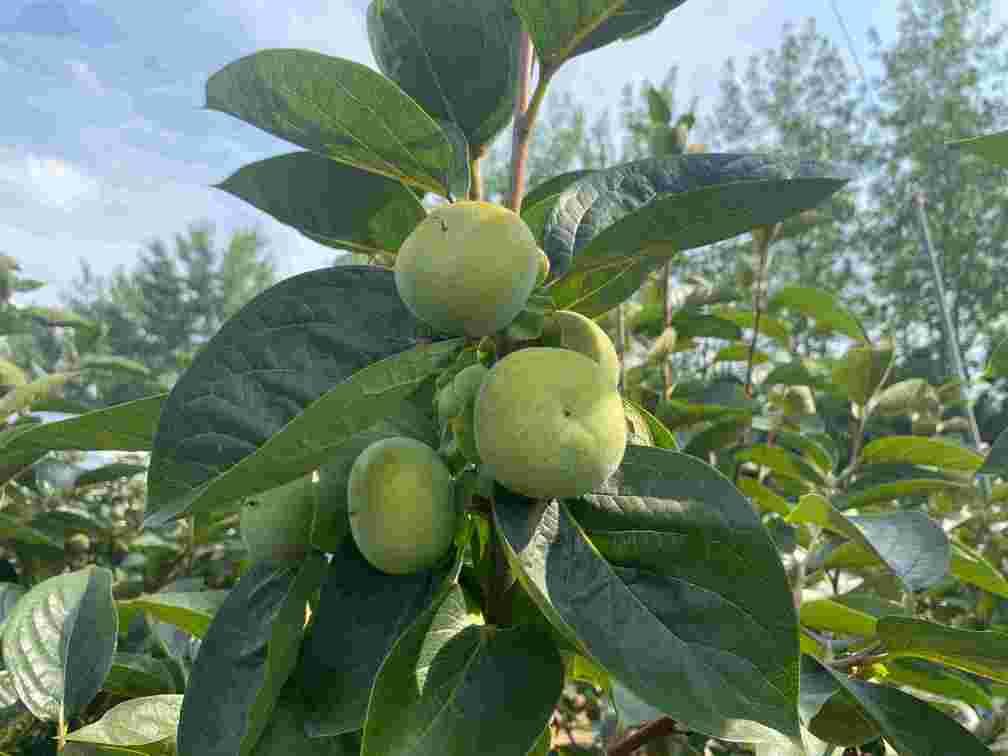

Supplement: Supplementary file 1 [file DataSheet1.zip › 2022-07-22 183454_20220722_183809.jpg]

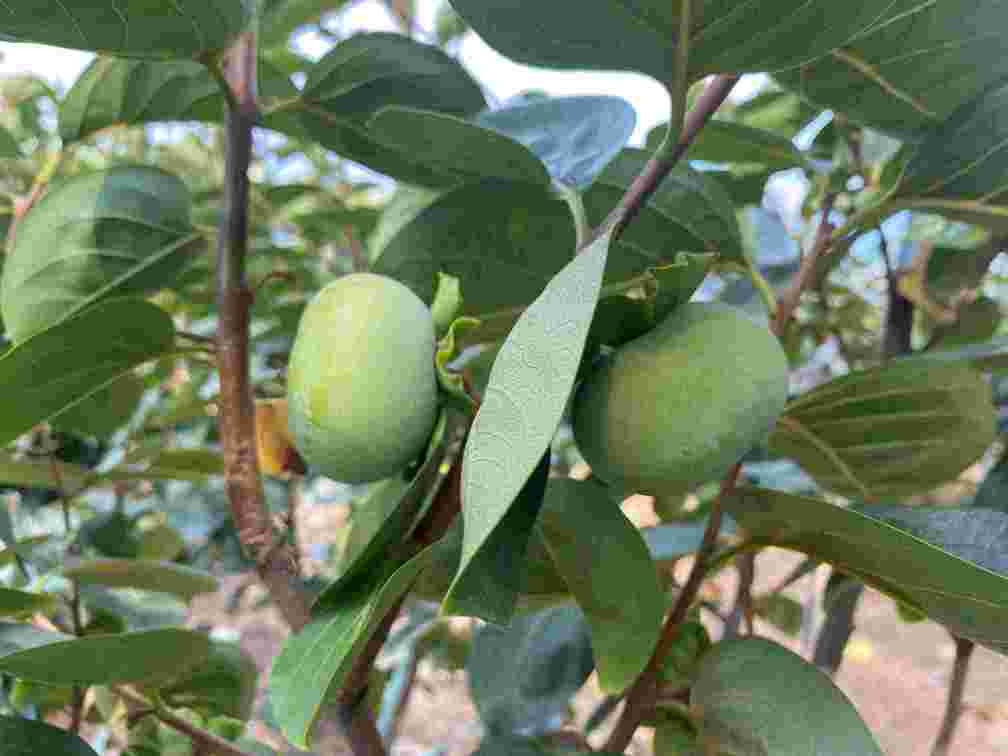

Supplement: Supplementary file 1 [file DataSheet1.zip › 2022-07-22 183454_20220722_183810.jpg]

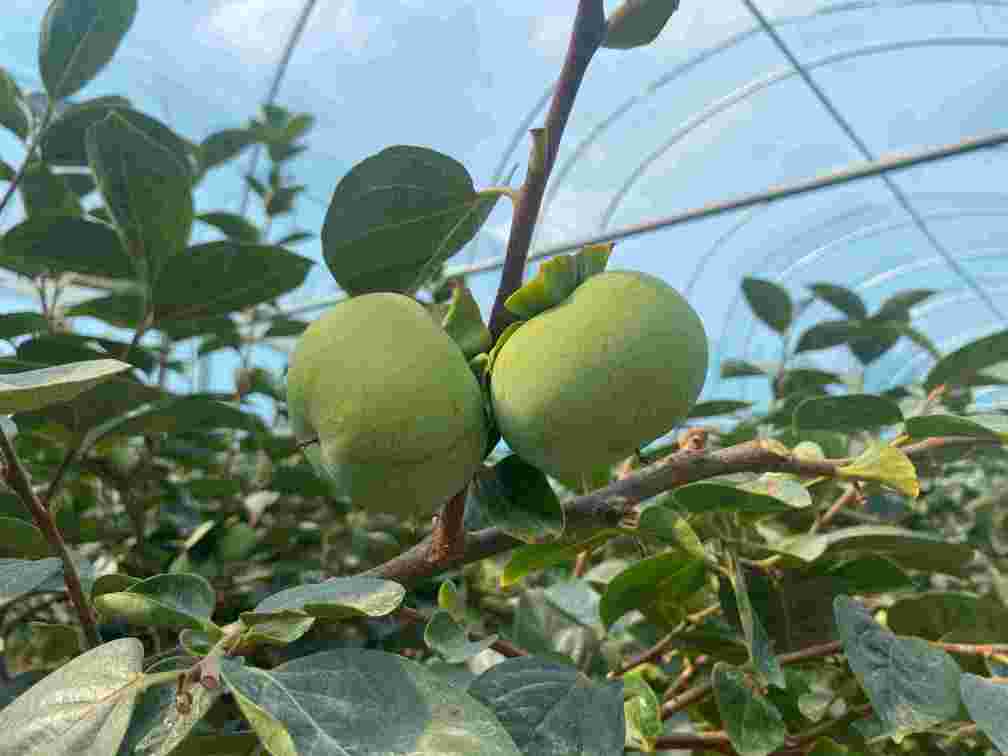

Supplement: Supplementary file 1 [file DataSheet1.zip › 2022-07-22 183454_20220722_183811.jpg]

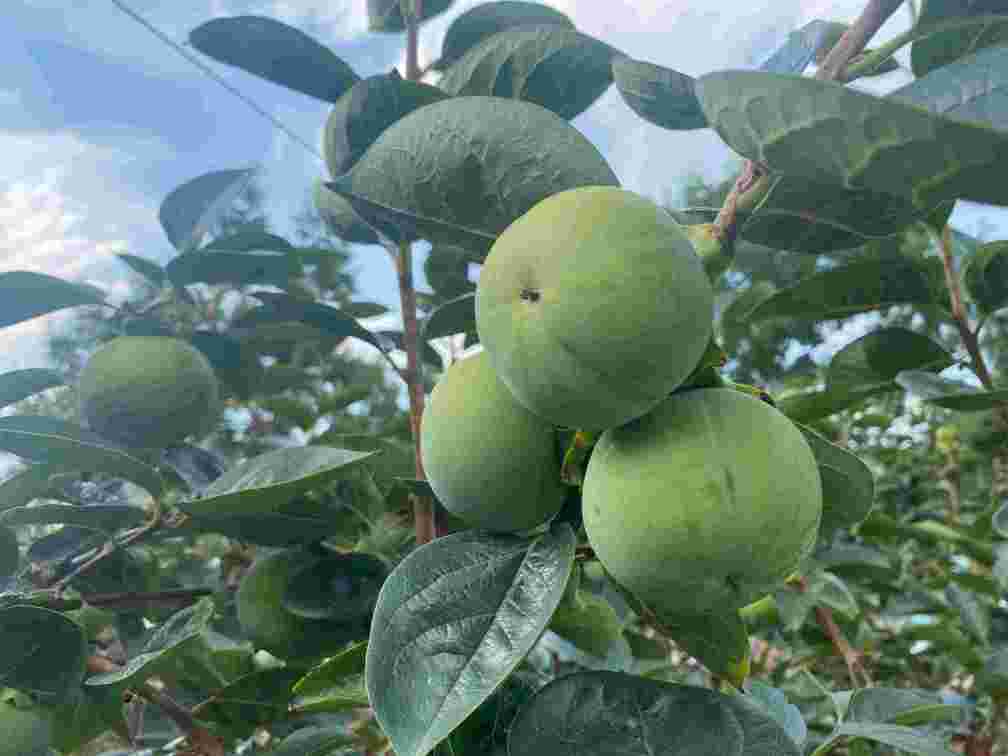

Supplement: Supplementary file 1 [file DataSheet1.zip › 2022-07-22 183454_20220722_183812.jpg]

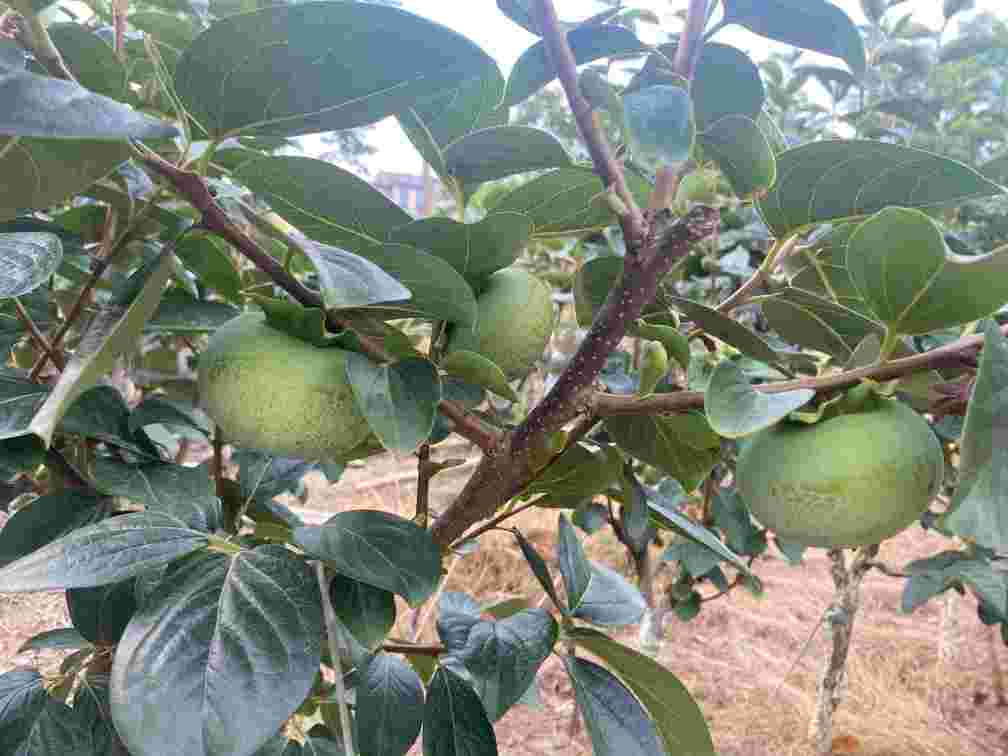

Supplement: Supplementary file 1 [file DataSheet1.zip › 2022-07-22 183454_20220722_183813.jpg]

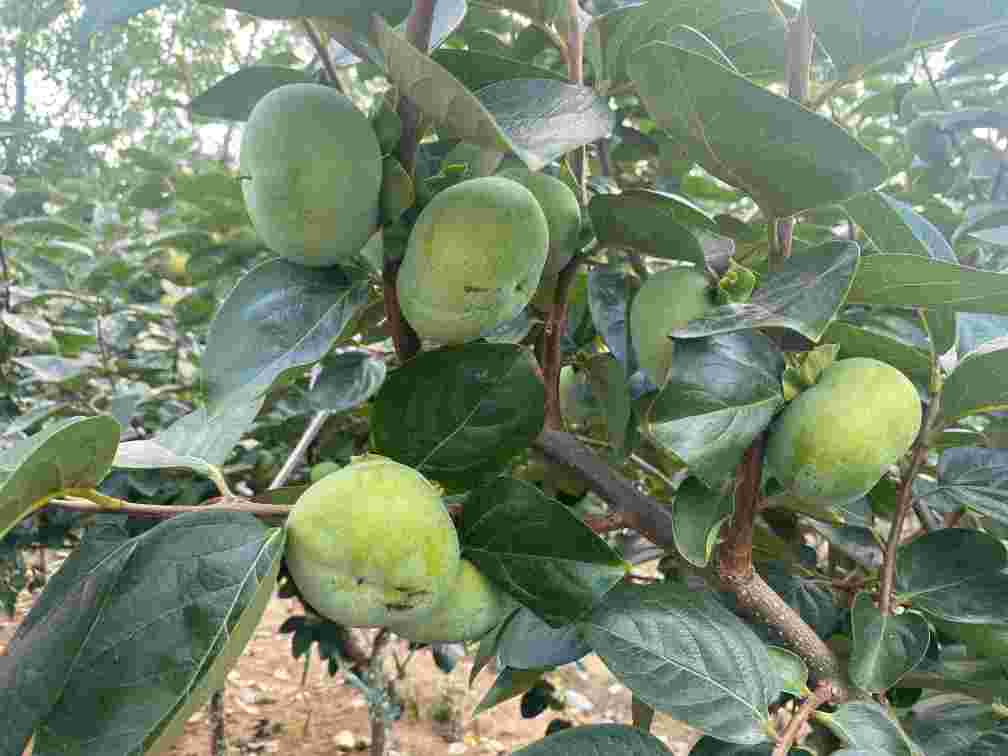

Supplement: Supplementary file 1 [file DataSheet1.zip › 2022-07-22 183454_20220722_183814.jpg]

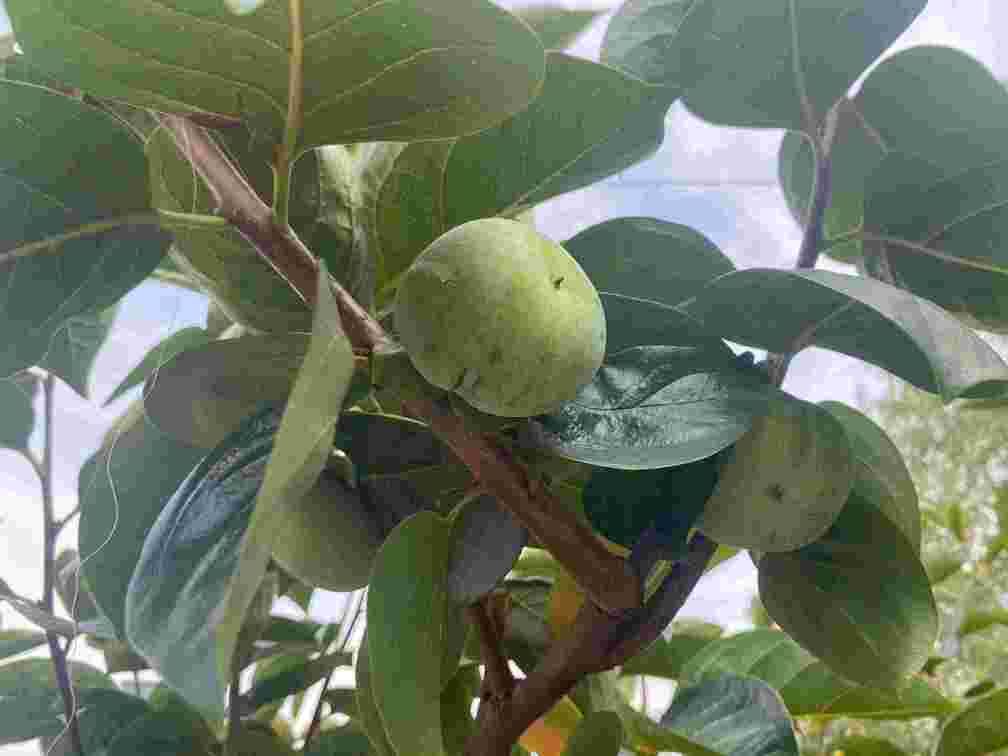

Supplement: Supplementary file 1 [file DataSheet1.zip › 2022-07-22 183454_20220722_183815.jpg]

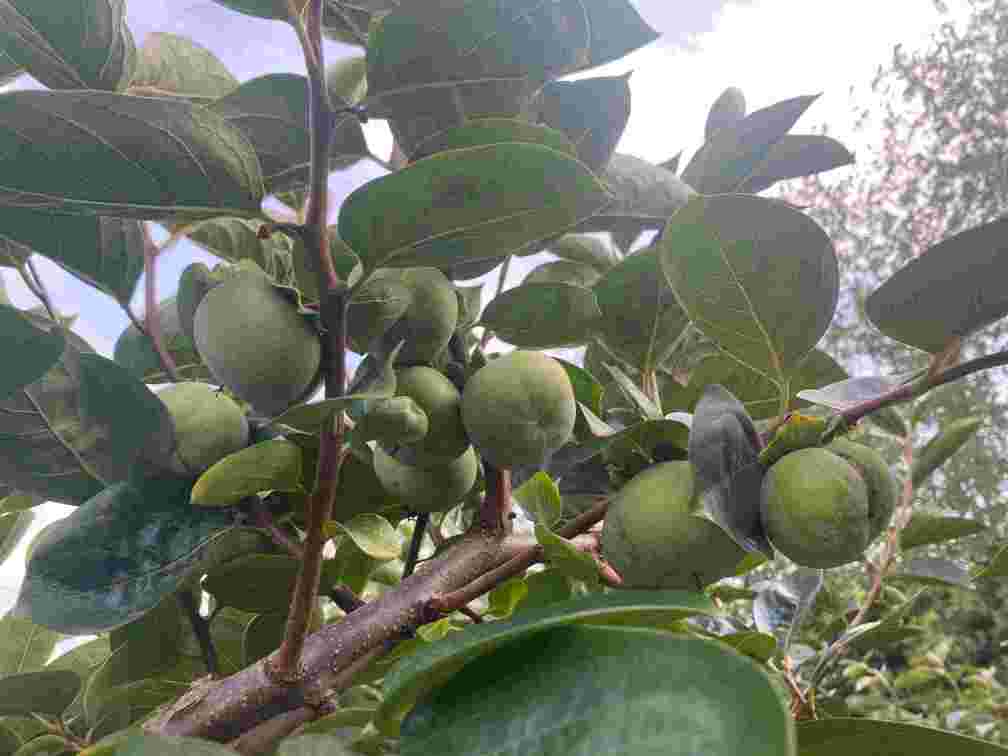

Supplement: Supplementary file 1 [file DataSheet1.zip › 2022-07-22 183454_20220722_183816.jpg]

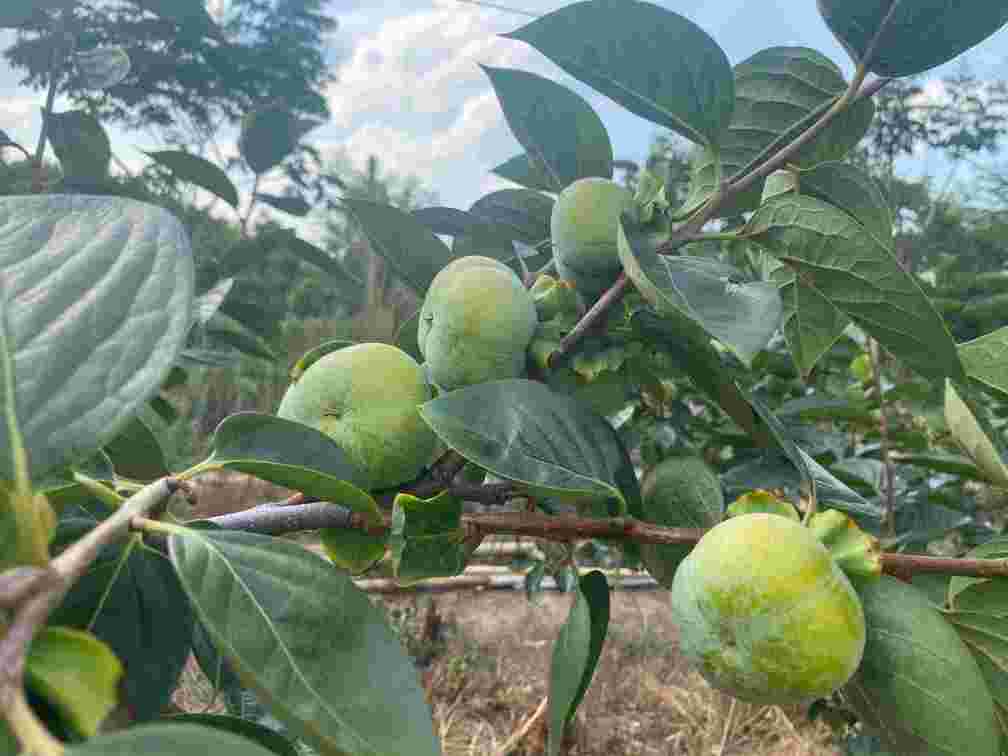

Supplement: Supplementary file 1 [file DataSheet1.zip › 2022-07-22 183454_20220722_183817.jpg]

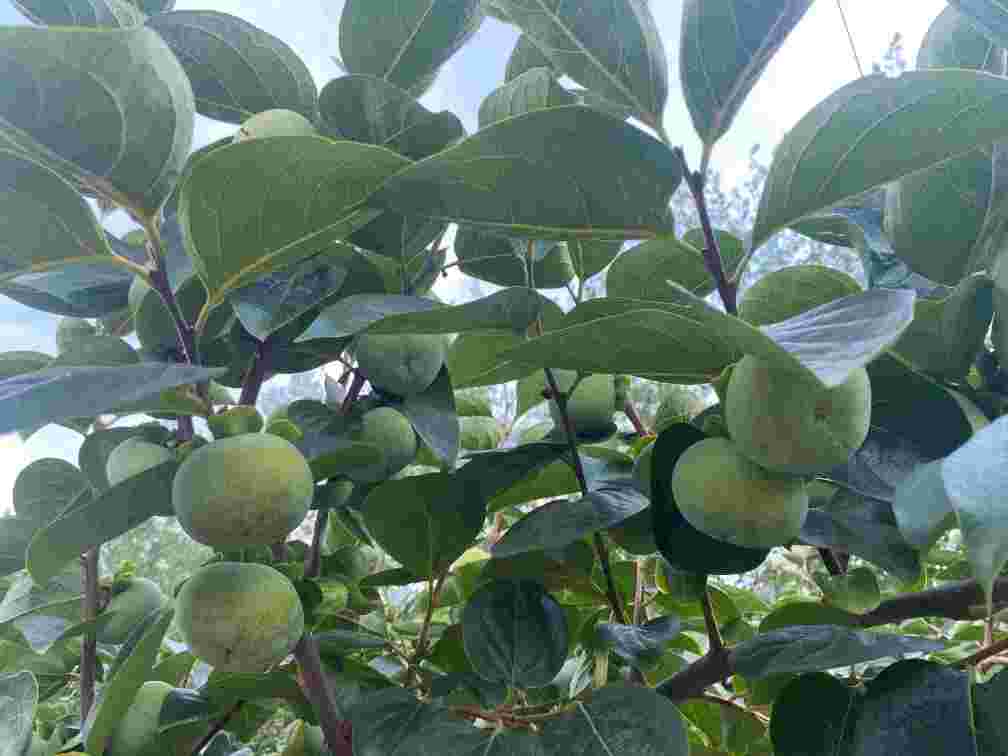

Supplement: Supplementary file 1 [file DataSheet1.zip › 2022-07-22 183454_20220722_183818.jpg]

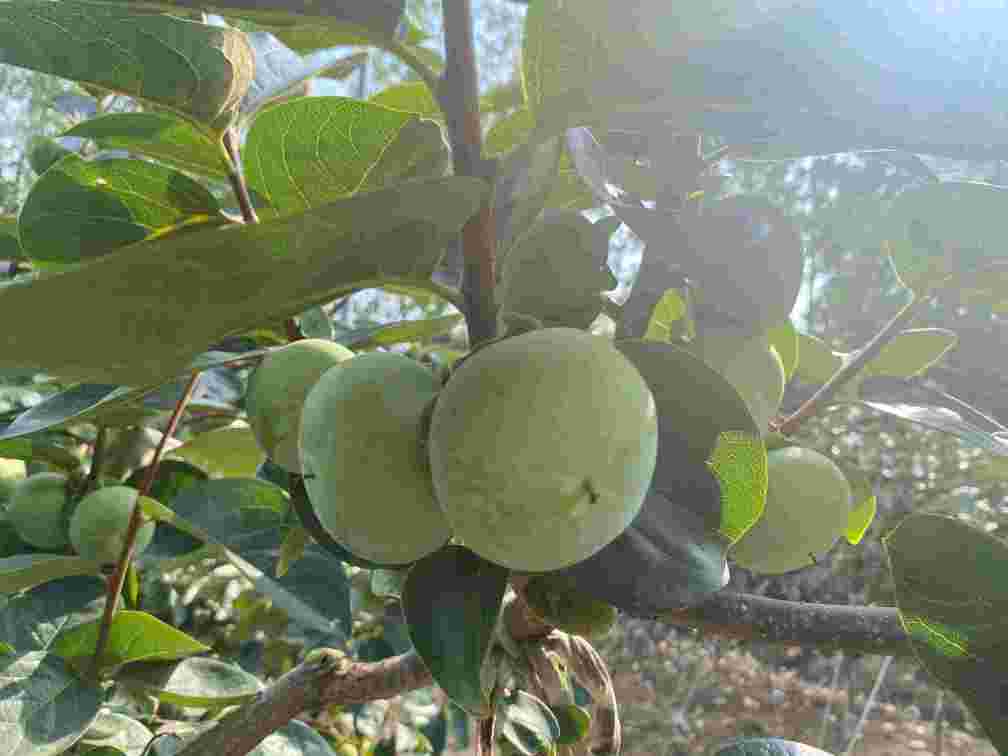

Supplement: Supplementary file 1 [file DataSheet1.zip › 2022-07-22 183454_20220722_183819.jpg]
