# Supplementary figures and images for: Research on persimmon fruit diameter accurate detection method based on improved RCNN instance segmentation algorithm (part 1 of 2)
Source: Front Plant Sci. 2025 Aug 29;16:1636727. doi: 10.3389/fpls.2025.1636727 (PMC12426853; doi:10.3389/fpls.2025.1636727)

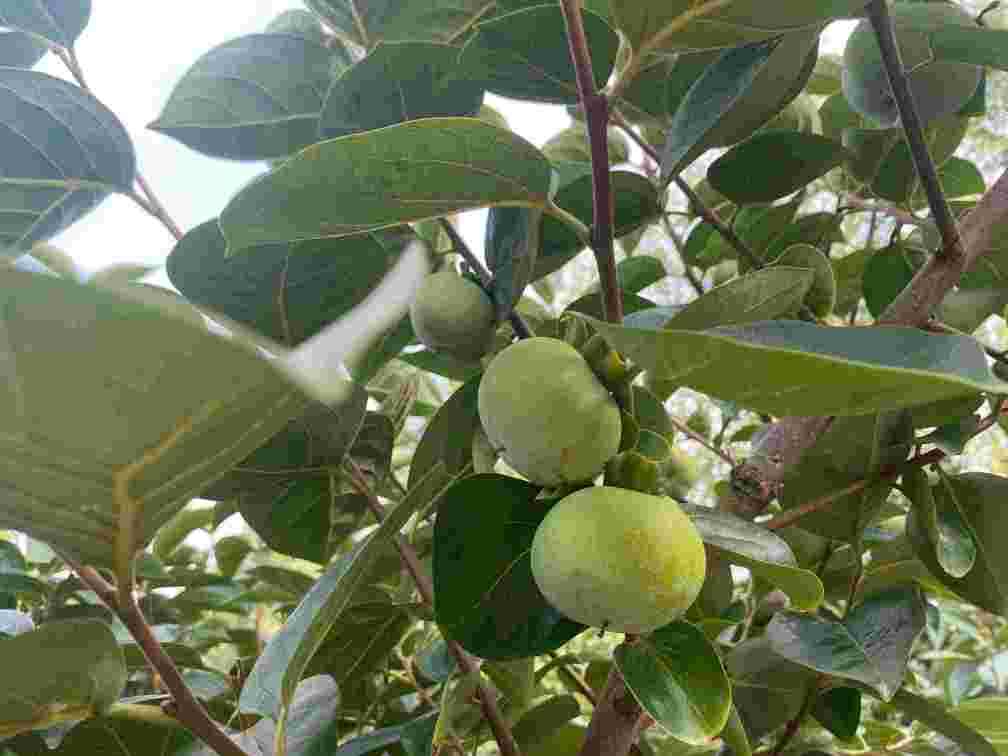

Supplement: Supplementary file 1 [file DataSheet1.zip › 2022-07-22 183454(12).jpg]

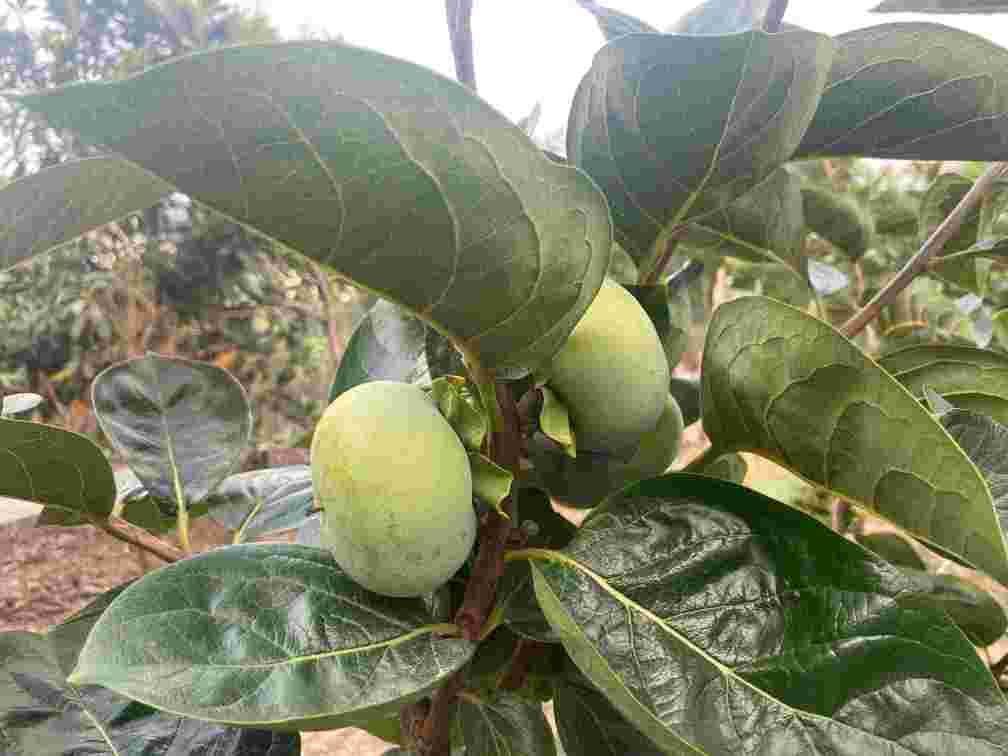

Supplement: Supplementary file 1 [file DataSheet1.zip › 2022-07-22 183454(14).jpg]

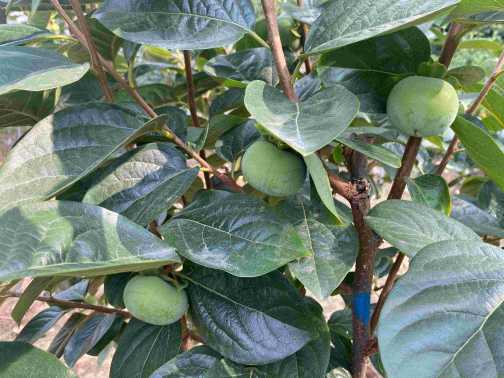

Supplement: Supplementary file 1 [file DataSheet1.zip › 2022-07-22 183454(15).jpg]

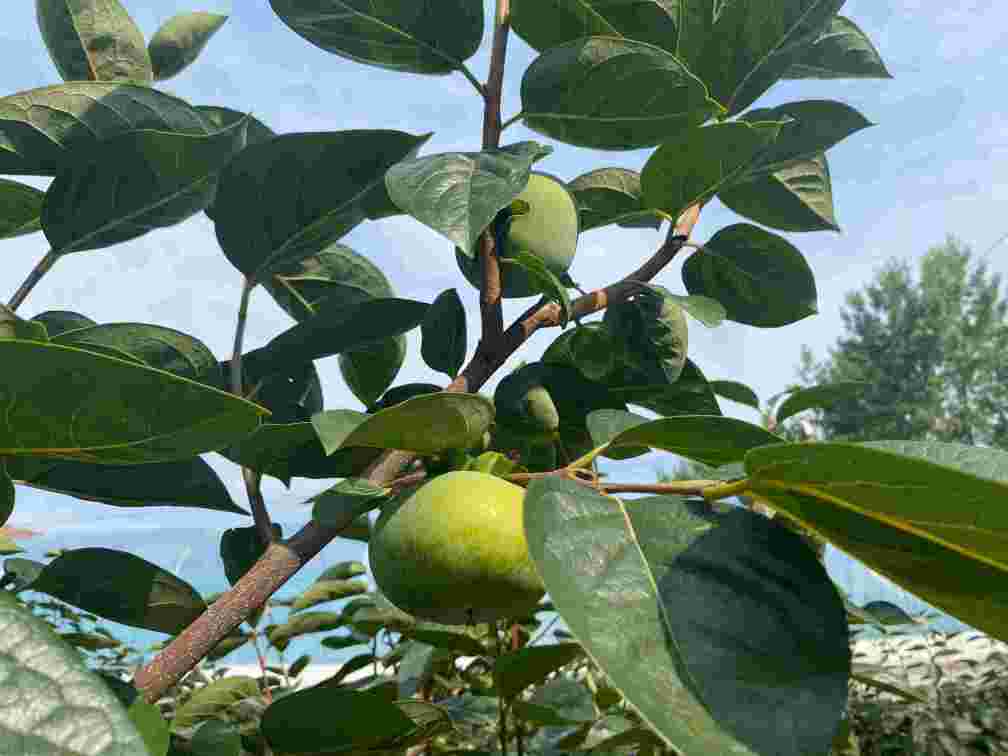

Supplement: Supplementary file 1 [file DataSheet1.zip › 2022-07-22 183454(18).jpg]

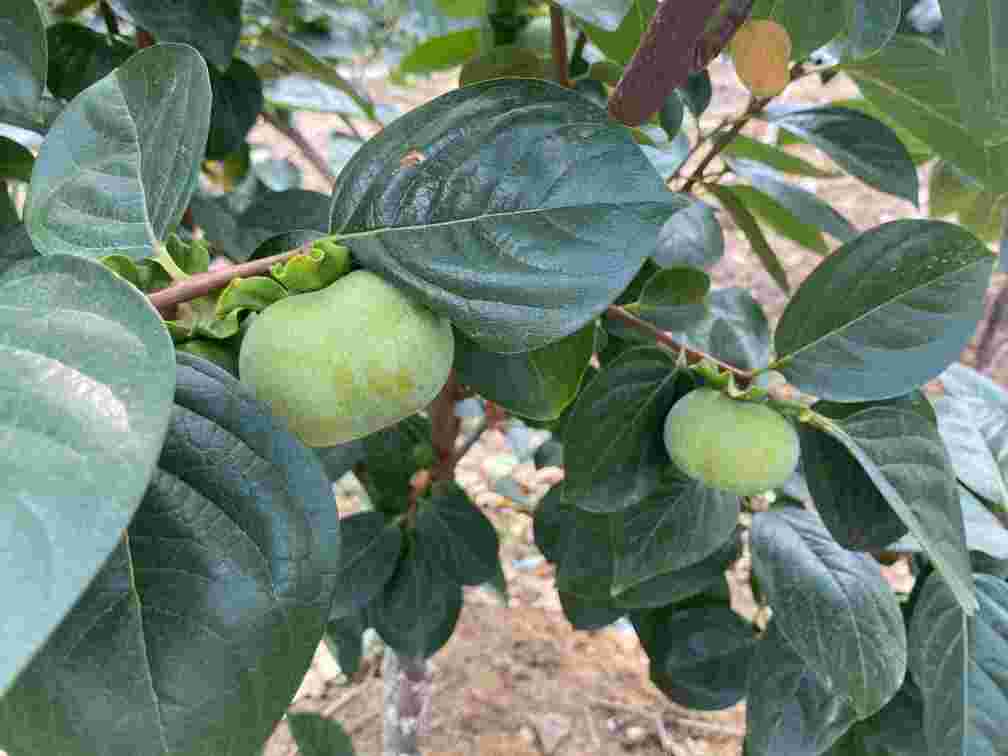

Supplement: Supplementary file 1 [file DataSheet1.zip › 2022-07-22 183454(21).jpg]

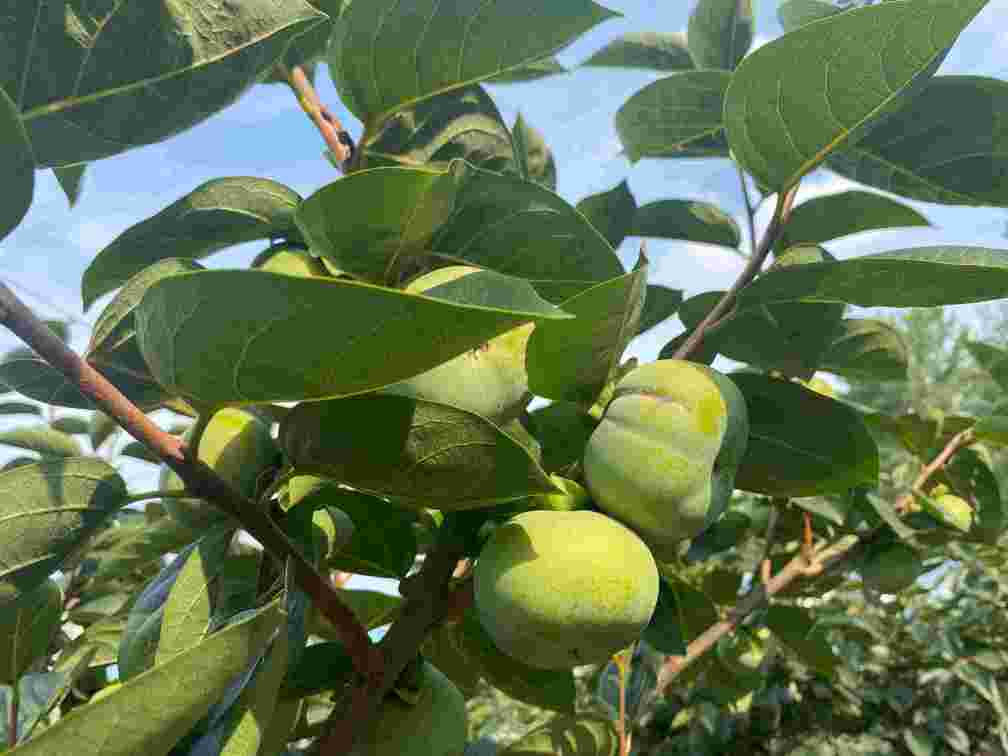

Supplement: Supplementary file 1 [file DataSheet1.zip › 2022-07-22 183454(22).jpg]

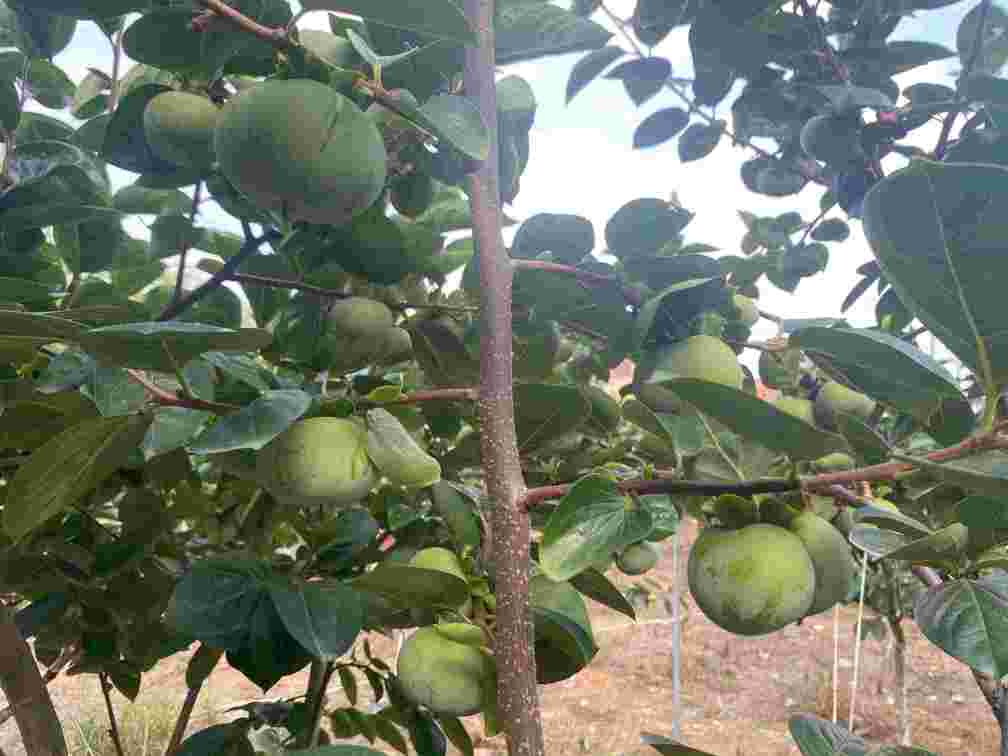

Supplement: Supplementary file 1 [file DataSheet1.zip › 2022-07-22 183454(23).jpg]

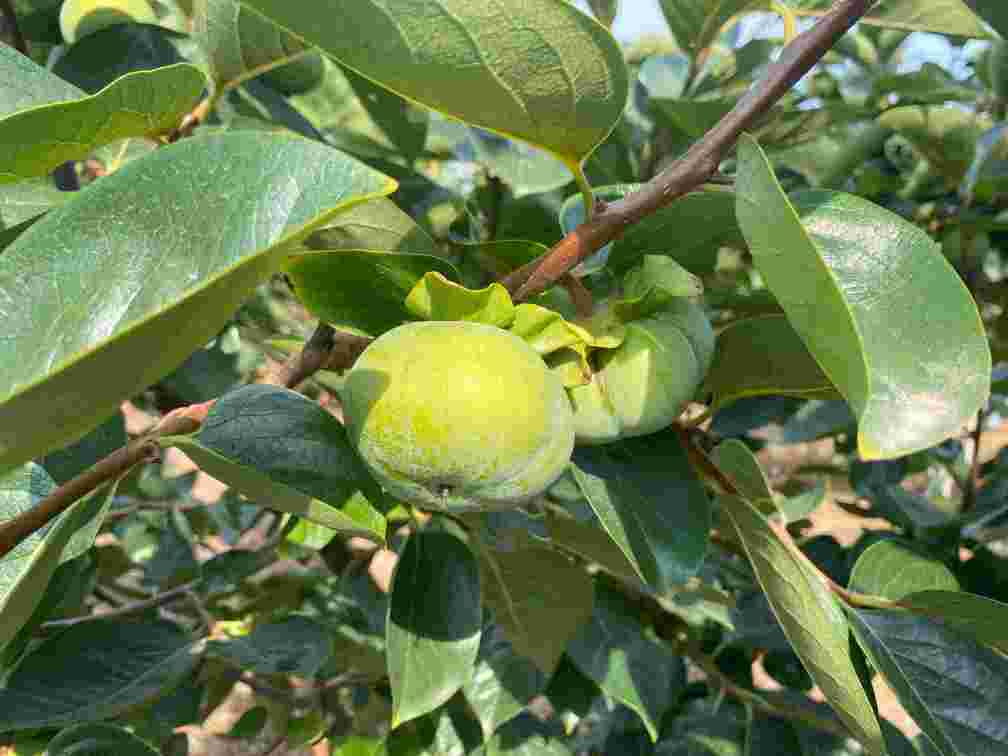

Supplement: Supplementary file 1 [file DataSheet1.zip › 2022-07-22 183454(24).jpg]

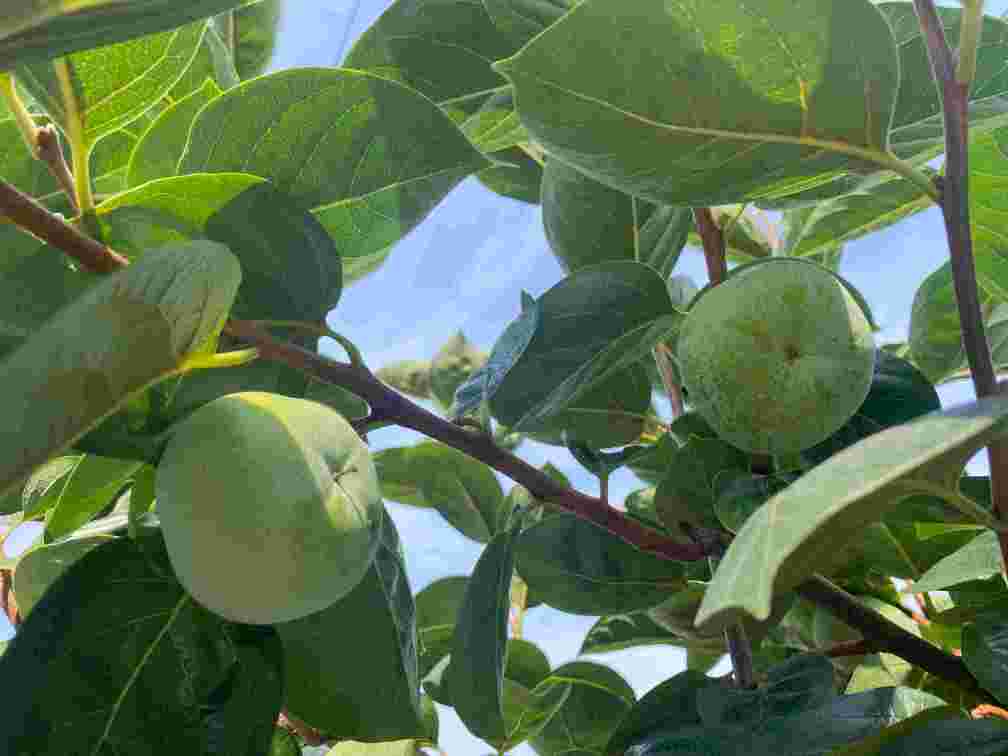

Supplement: Supplementary file 1 [file DataSheet1.zip › 2022-07-22 183454(24)_20220722_183545.jpg]

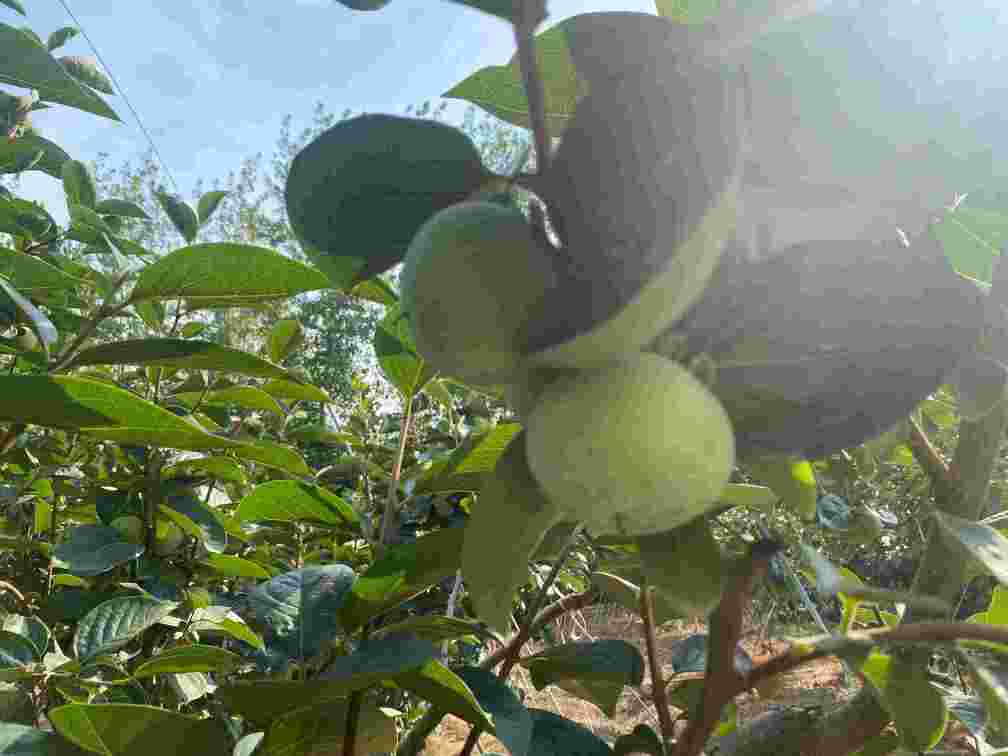

Supplement: Supplementary file 1 [file DataSheet1.zip › 2022-07-22 183454(26).jpg]

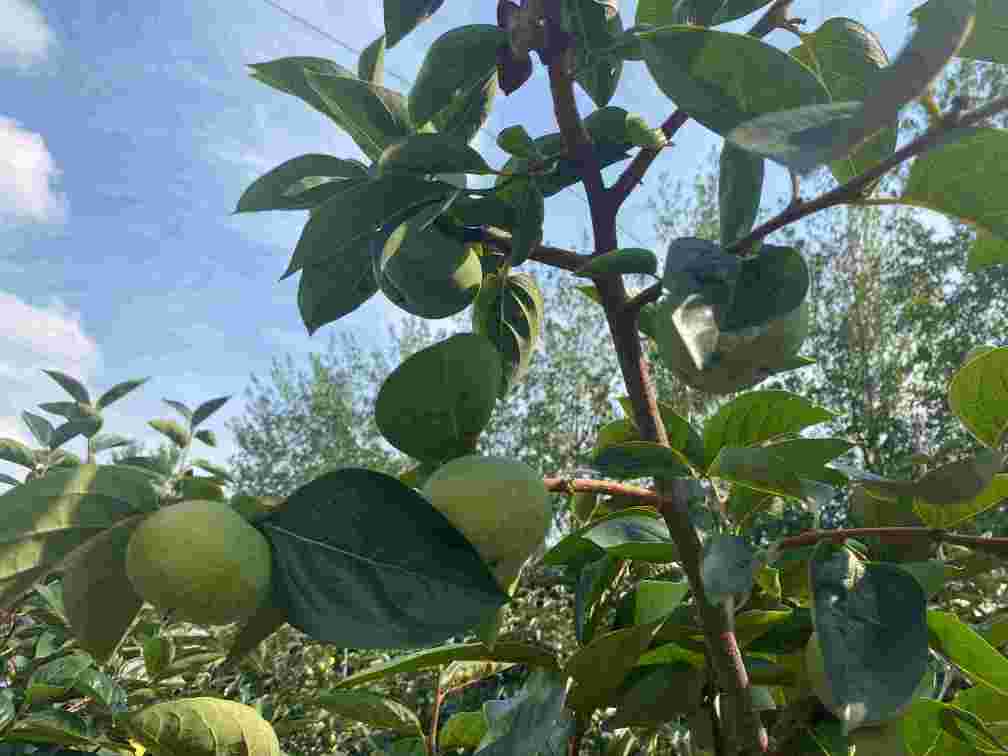

Supplement: Supplementary file 1 [file DataSheet1.zip › 2022-07-22 183454(28).jpg]

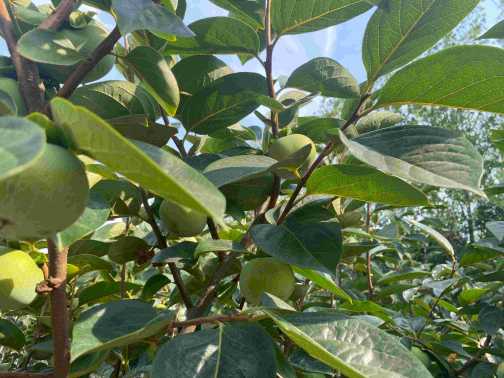

Supplement: Supplementary file 1 [file DataSheet1.zip › 2022-07-22 183454(31).jpg]

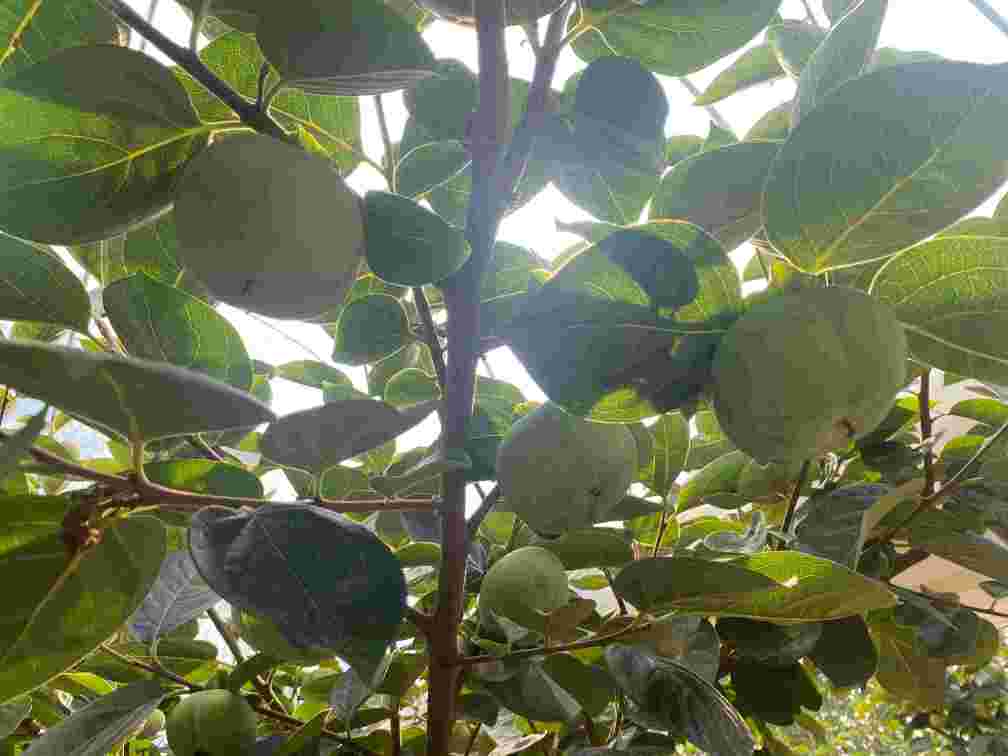

Supplement: Supplementary file 1 [file DataSheet1.zip › 2022-07-22 183454(33).jpg]

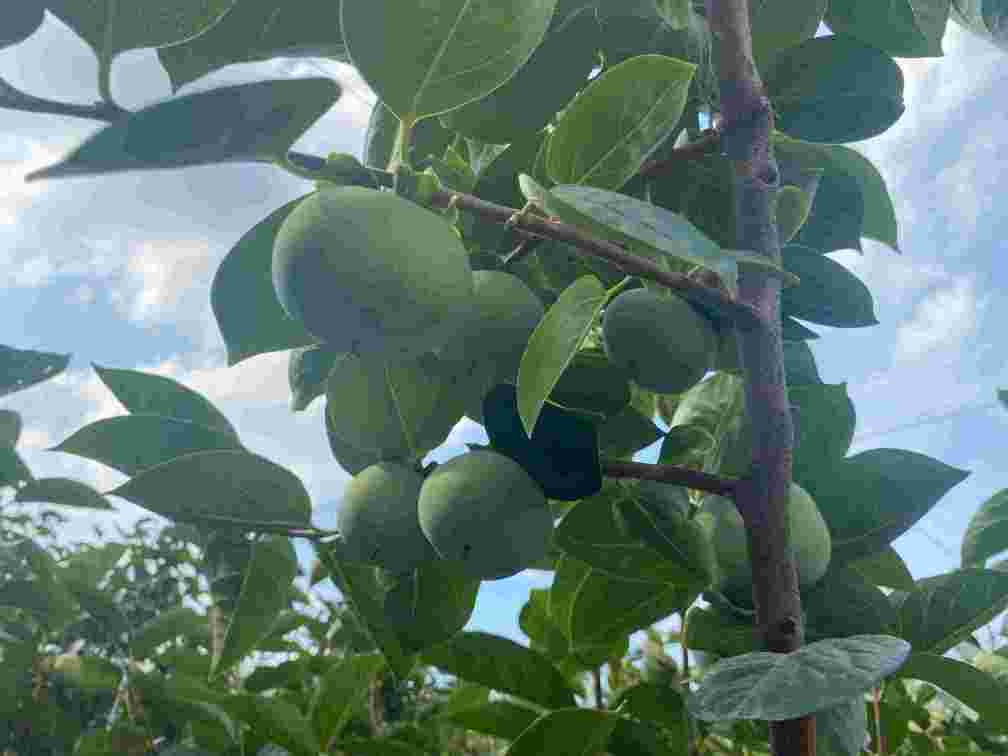

Supplement: Supplementary file 1 [file DataSheet1.zip › 2022-07-22 183454(34)_20220722_183550.jpg]

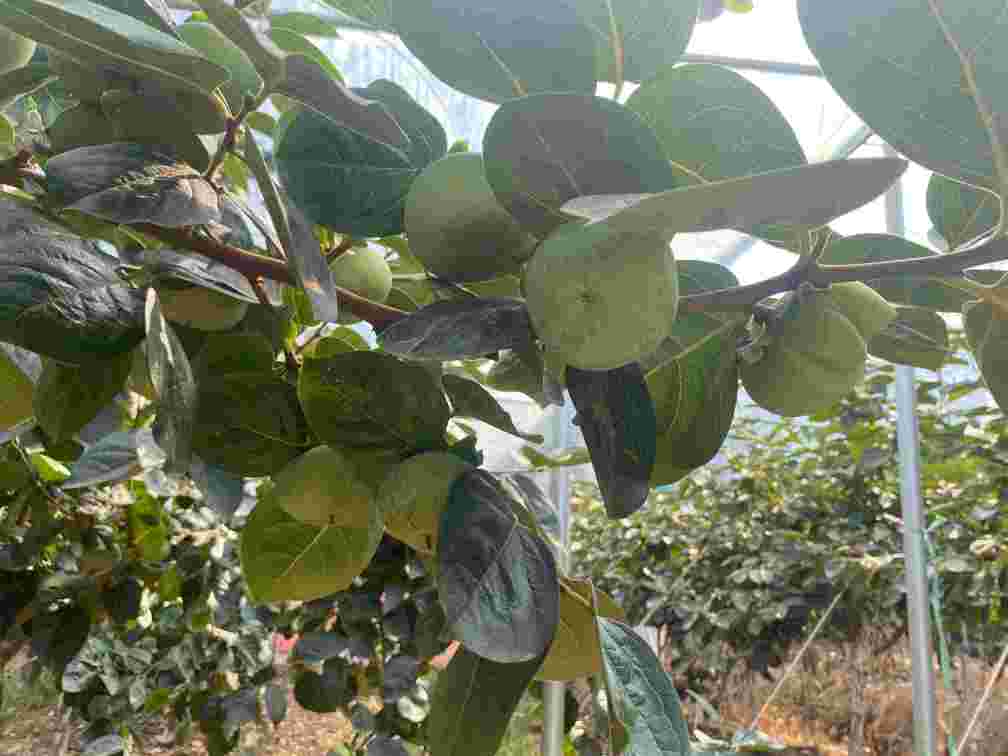

Supplement: Supplementary file 1 [file DataSheet1.zip › 2022-07-22 183454(35).jpg]

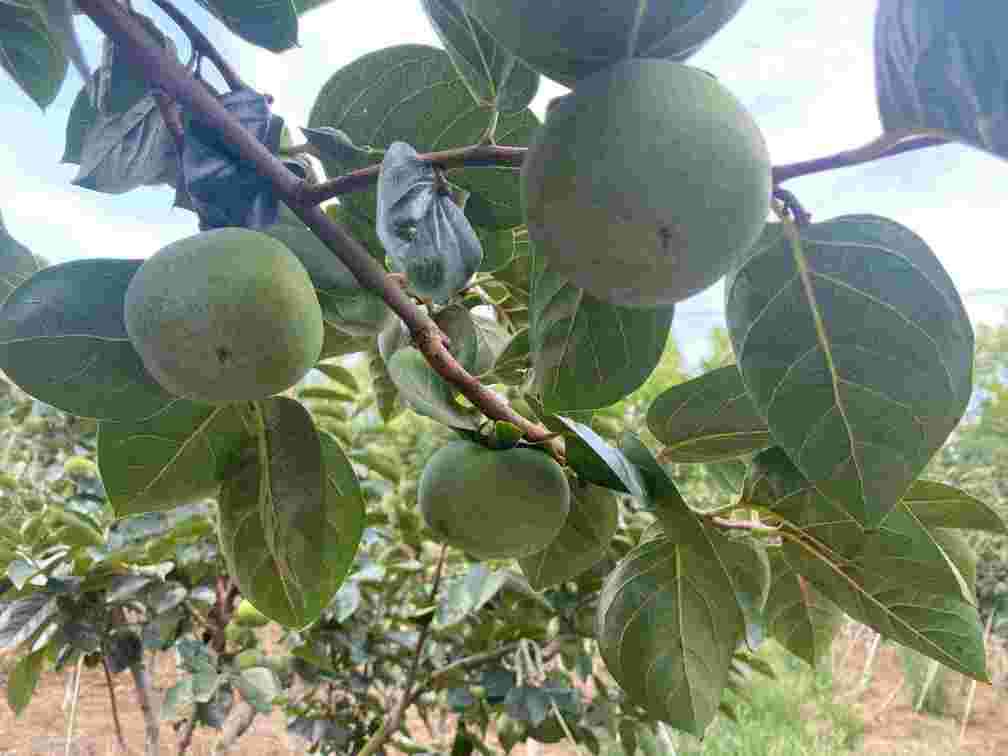

Supplement: Supplementary file 1 [file DataSheet1.zip › 2022-07-22 183454(36).jpg]

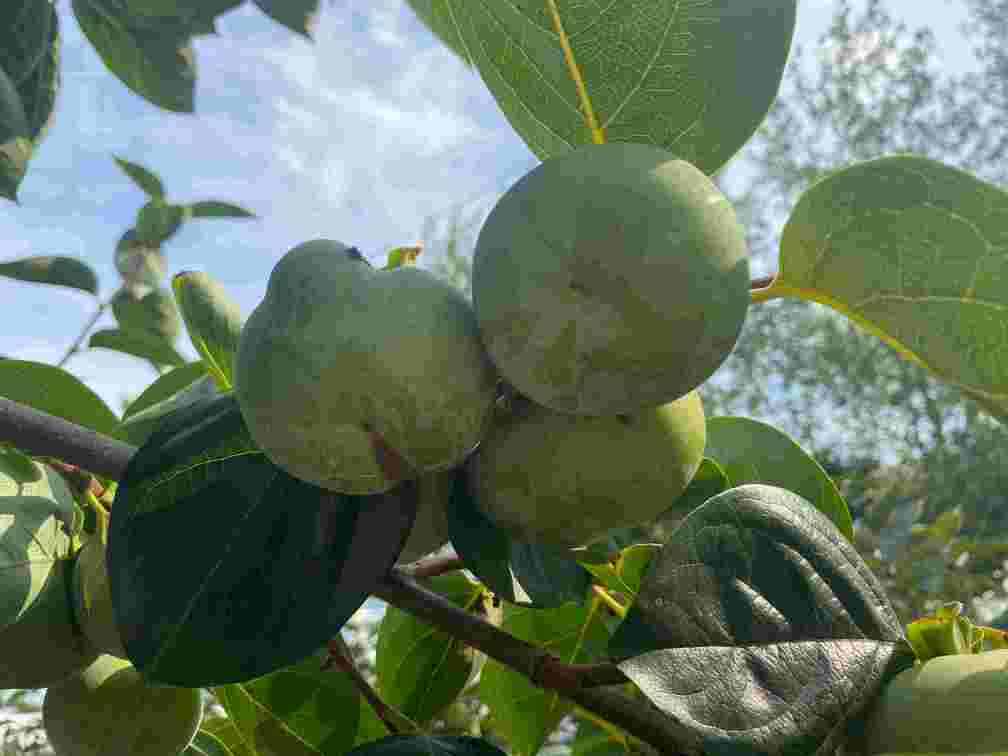

Supplement: Supplementary file 1 [file DataSheet1.zip › 2022-07-22 183454(37).jpg]

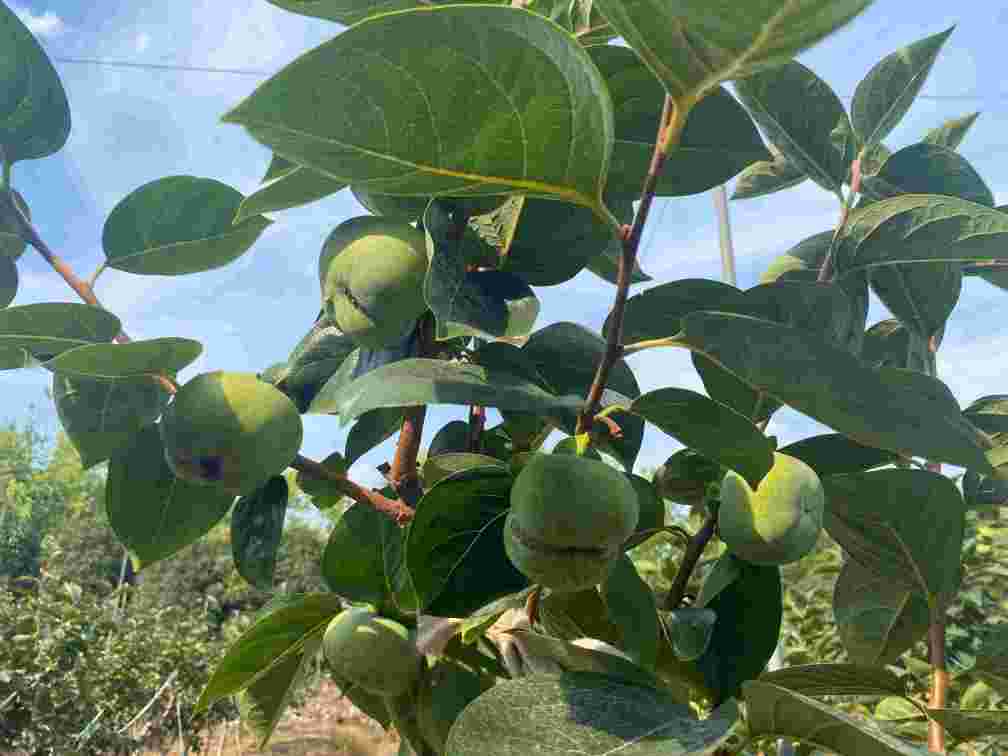

Supplement: Supplementary file 1 [file DataSheet1.zip › 2022-07-22 183454(38).jpg]

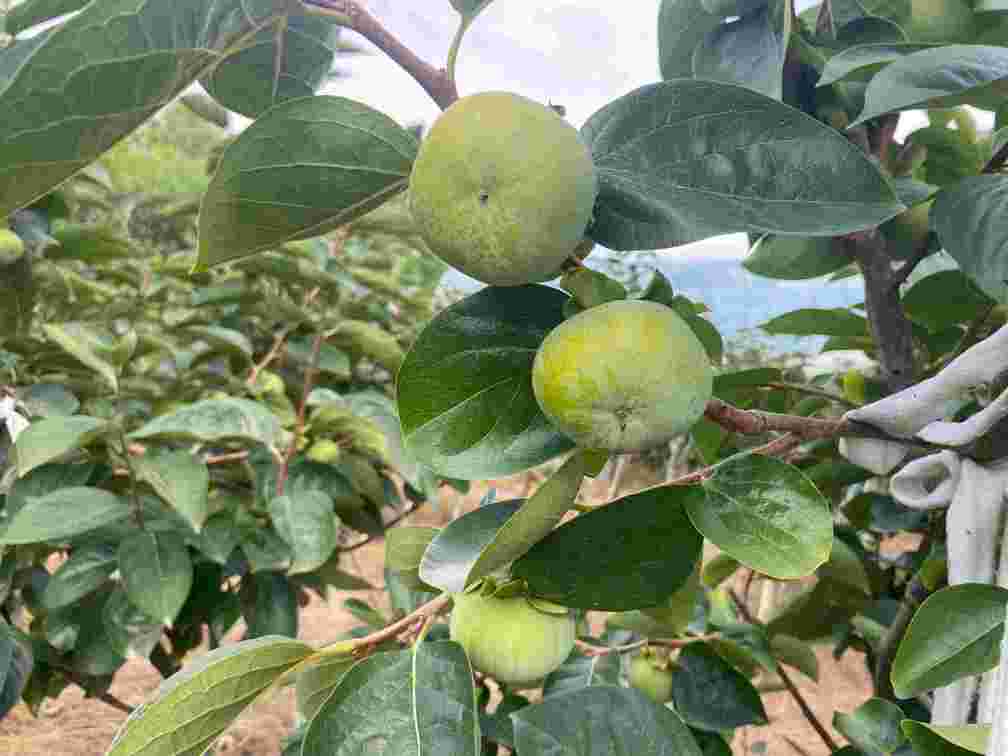

Supplement: Supplementary file 1 [file DataSheet1.zip › 2022-07-22 183454(39).jpg]

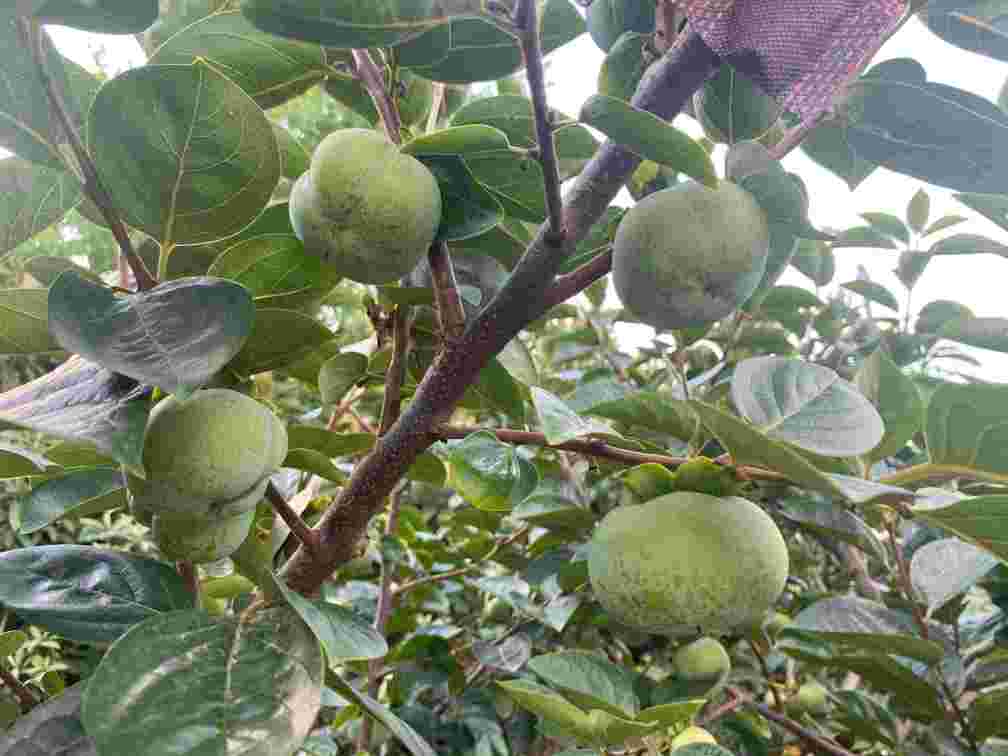

Supplement: Supplementary file 1 [file DataSheet1.zip › 2022-07-22 183454(40).jpg]

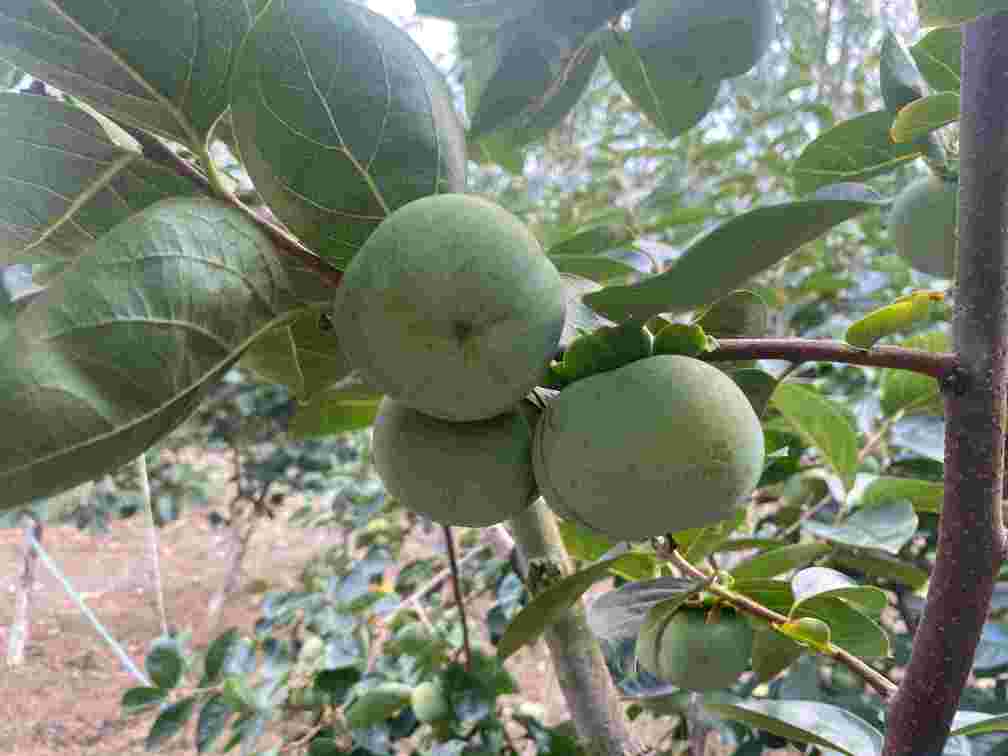

Supplement: Supplementary file 1 [file DataSheet1.zip › 2022-07-22 183454(40)_20220722_183554.jpg]

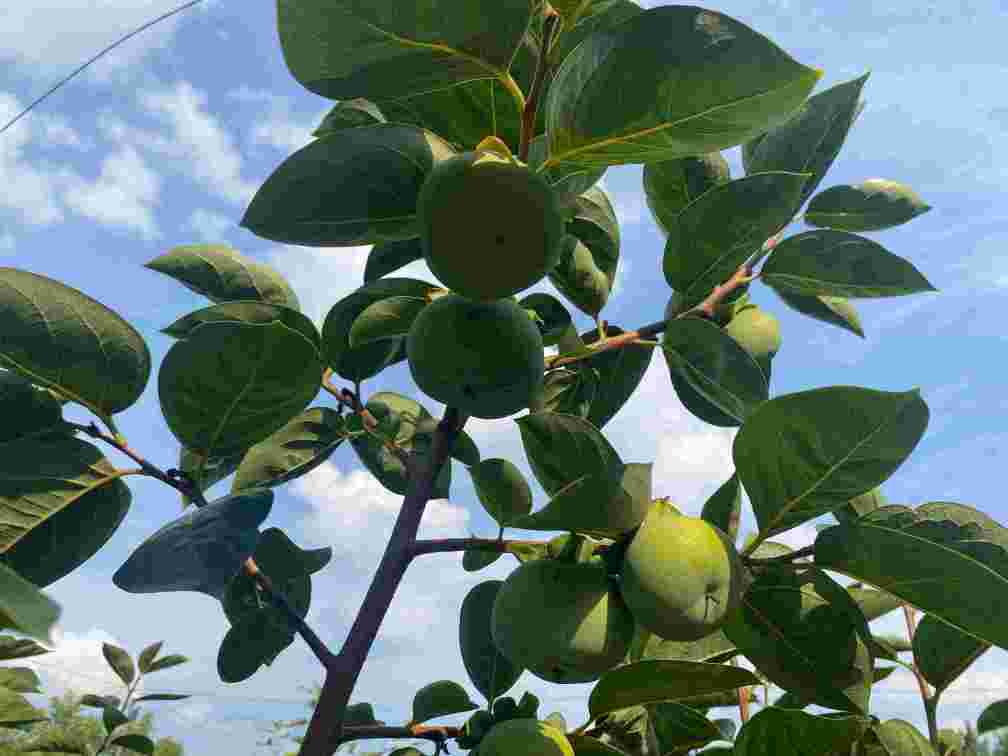

Supplement: Supplementary file 1 [file DataSheet1.zip › 2022-07-22 183454(41).jpg]

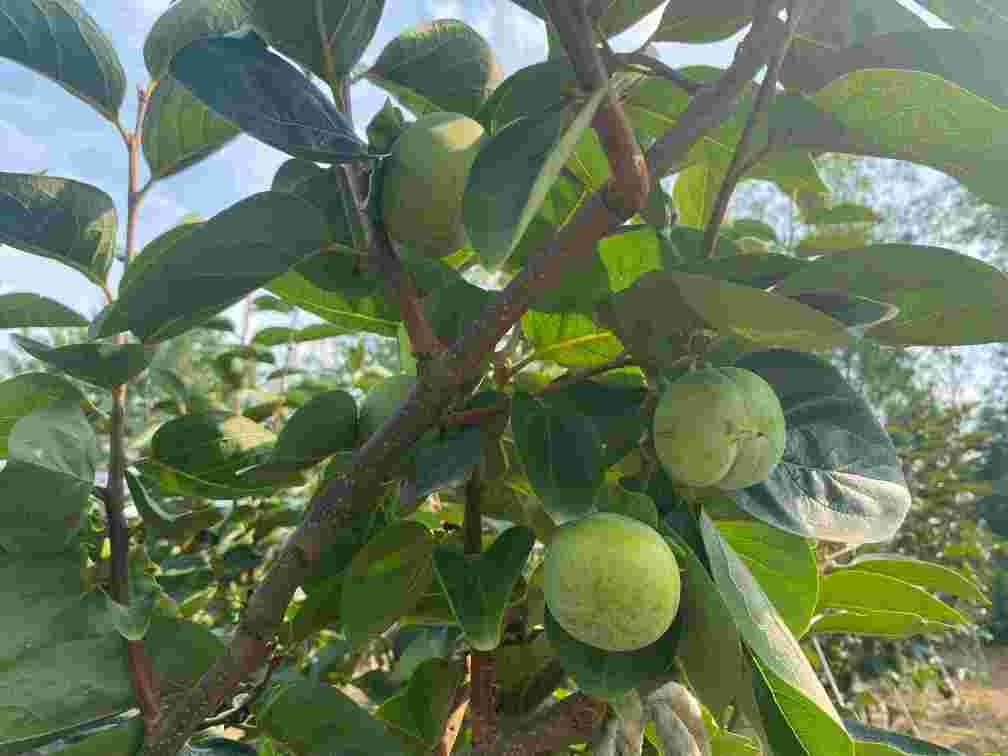

Supplement: Supplementary file 1 [file DataSheet1.zip › 2022-07-22 183454(42).jpg]

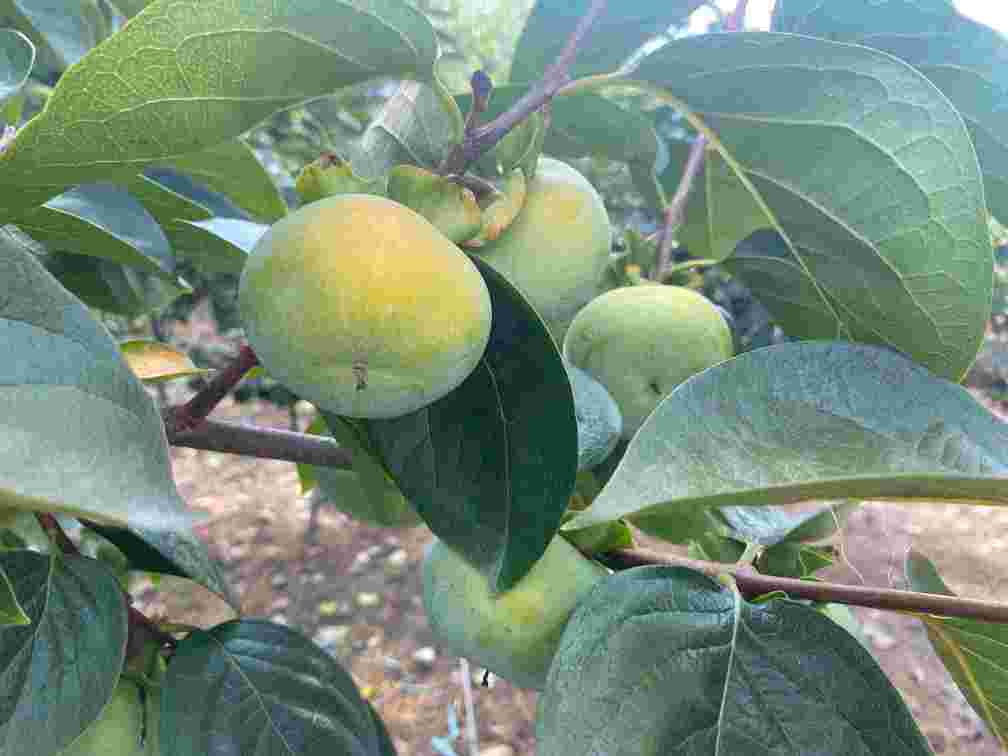

Supplement: Supplementary file 1 [file DataSheet1.zip › 2022-07-22 183454(43).jpg]

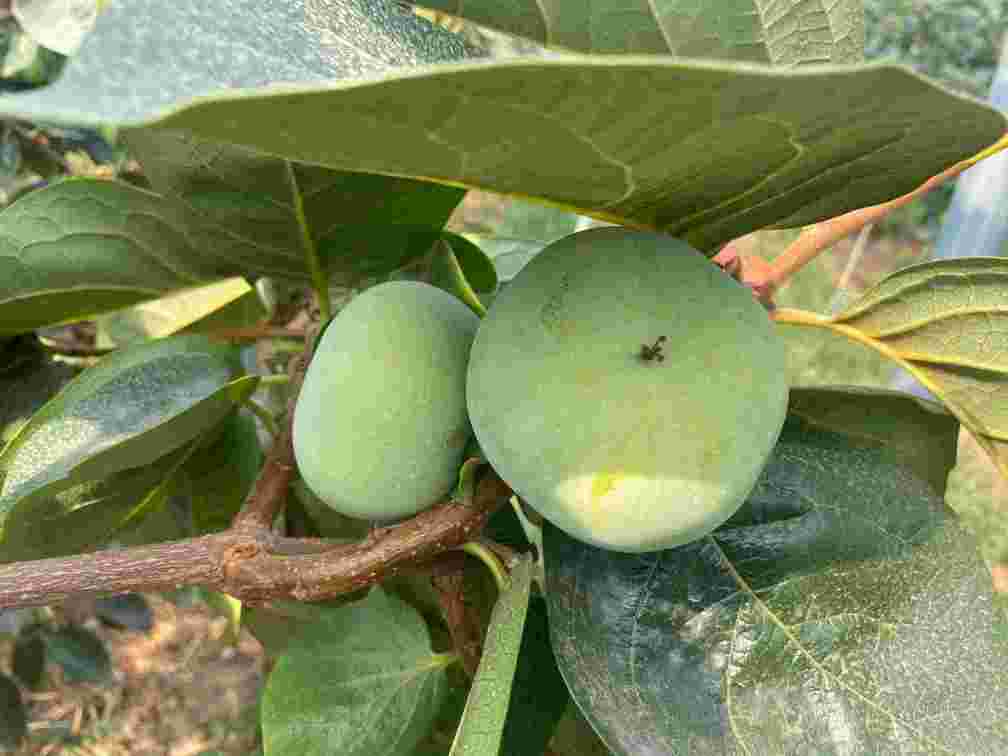

Supplement: Supplementary file 1 [file DataSheet1.zip › 2022-07-22 183454(44).jpg]

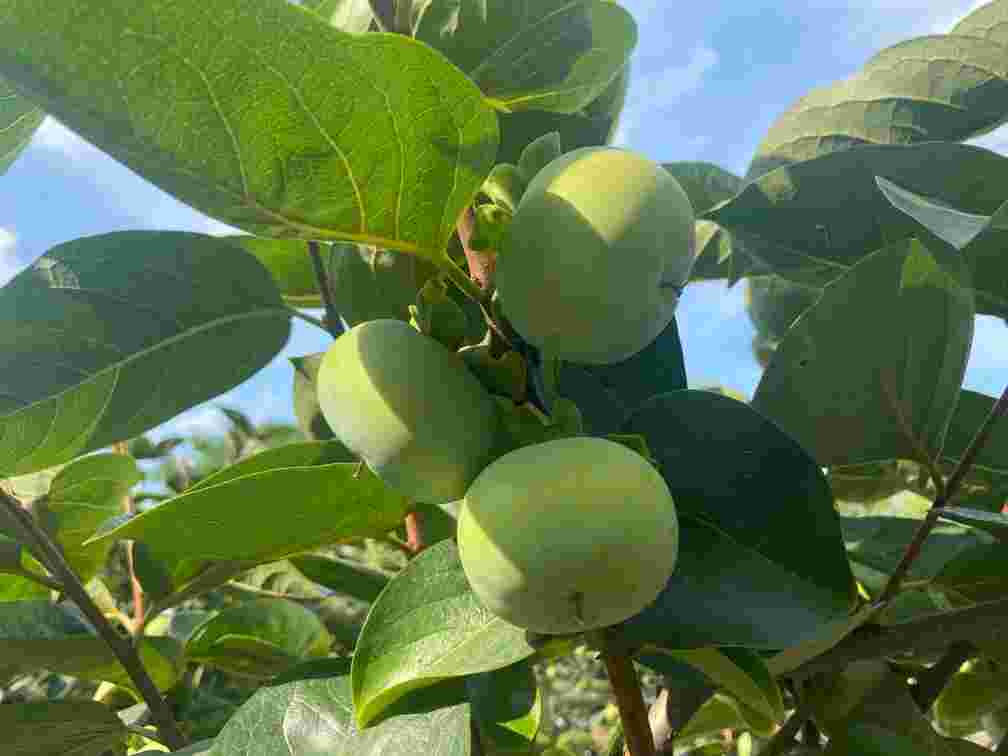

Supplement: Supplementary file 1 [file DataSheet1.zip › 2022-07-22 183454(45).jpg]

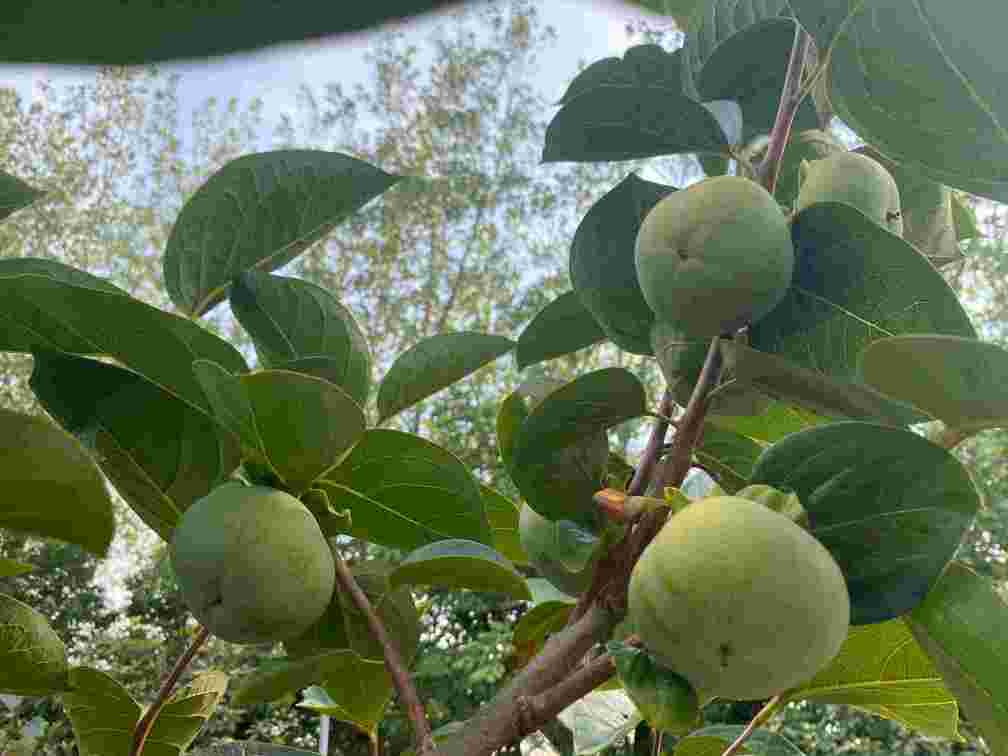

Supplement: Supplementary file 1 [file DataSheet1.zip › 2022-07-22 183454(46).jpg]

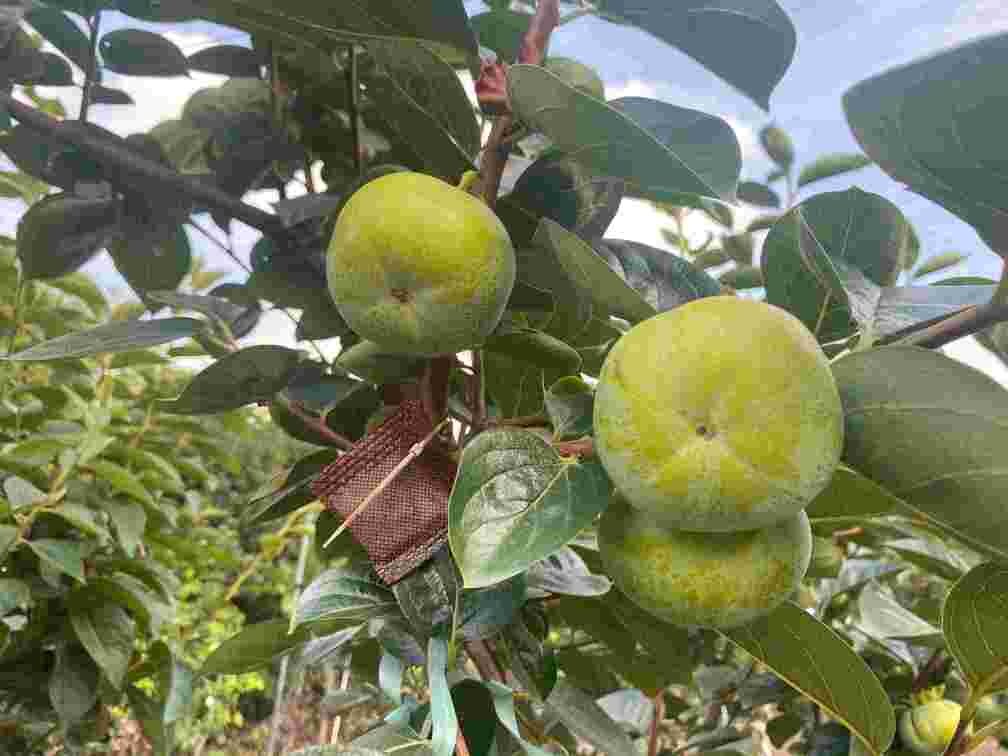

Supplement: Supplementary file 1 [file DataSheet1.zip › 2022-07-22 183454(47).jpg]

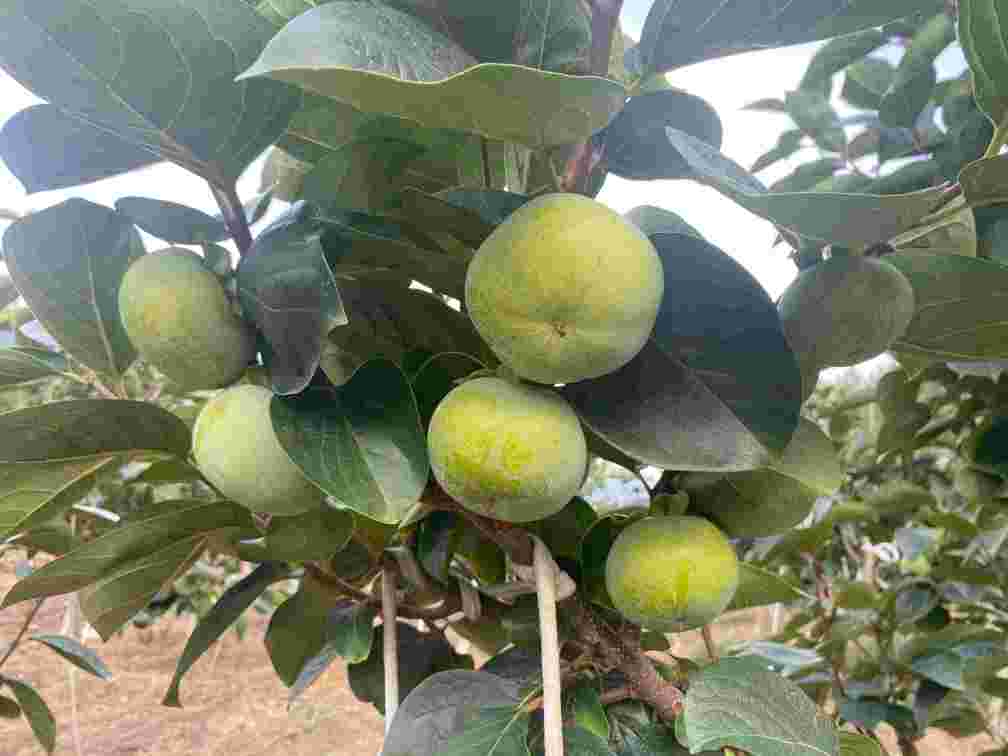

Supplement: Supplementary file 1 [file DataSheet1.zip › 2022-07-22 183454(48).jpg]

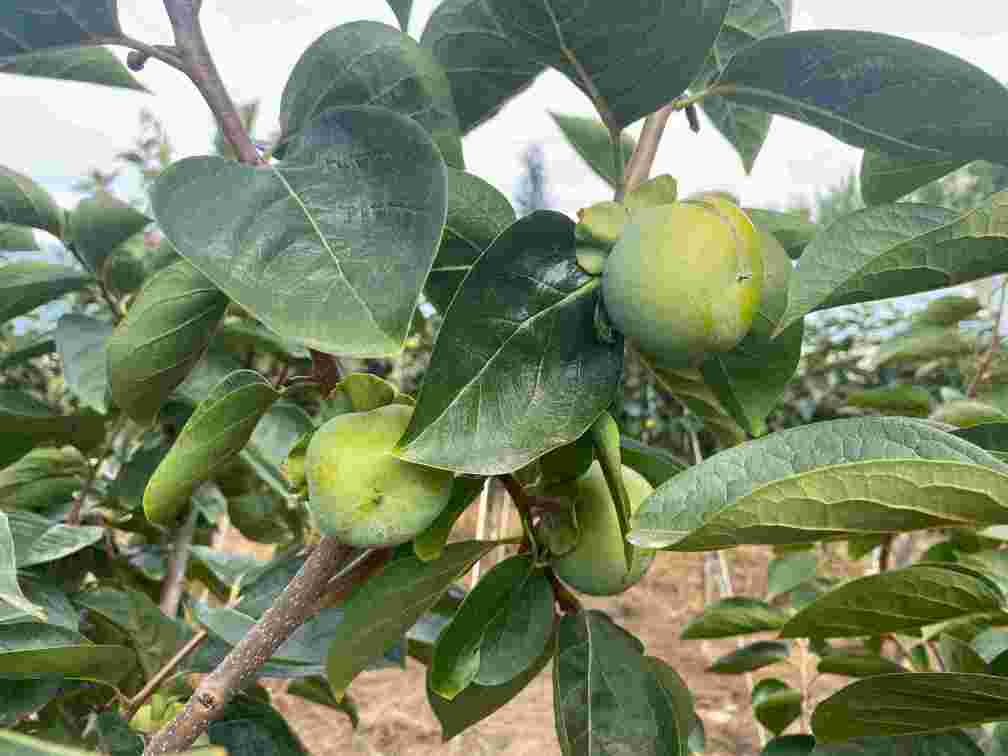

Supplement: Supplementary file 1 [file DataSheet1.zip › 2022-07-22 183454(48)_20220722_183557.jpg]

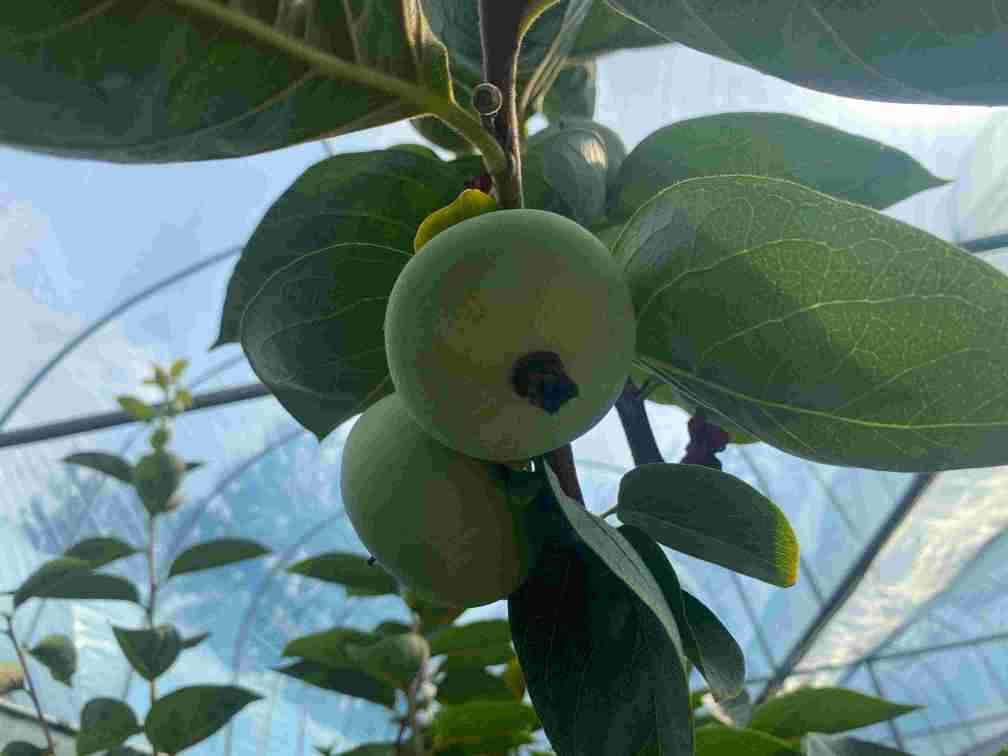

Supplement: Supplementary file 1 [file DataSheet1.zip › 2022-07-22 183454(49).jpg]

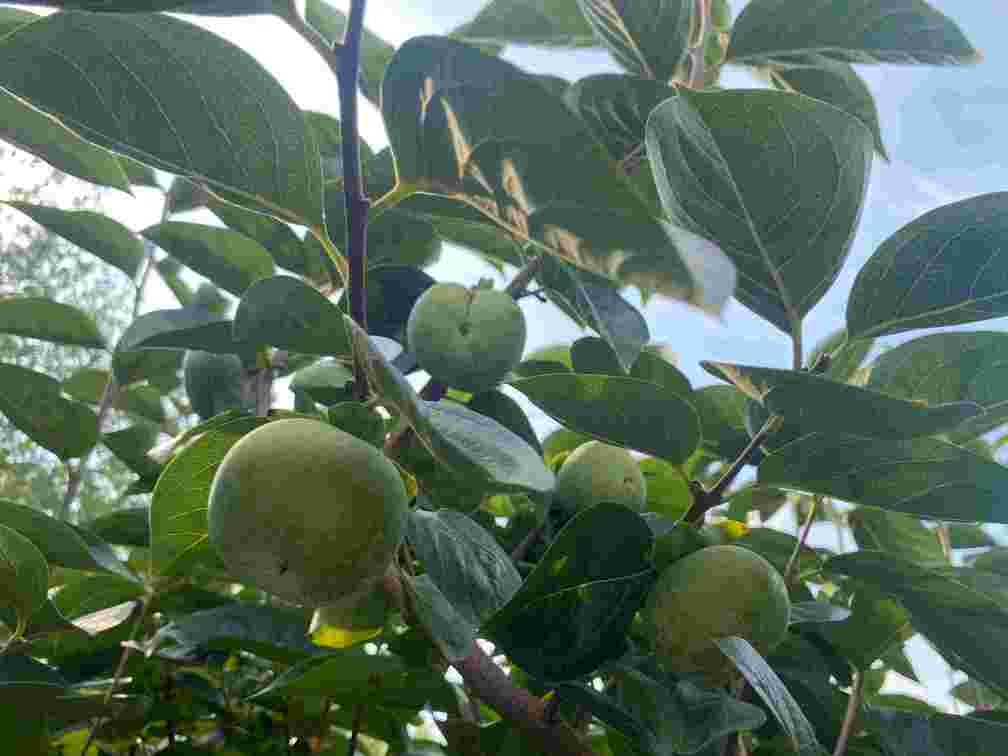

Supplement: Supplementary file 1 [file DataSheet1.zip › 2022-07-22 183454(50).jpg]

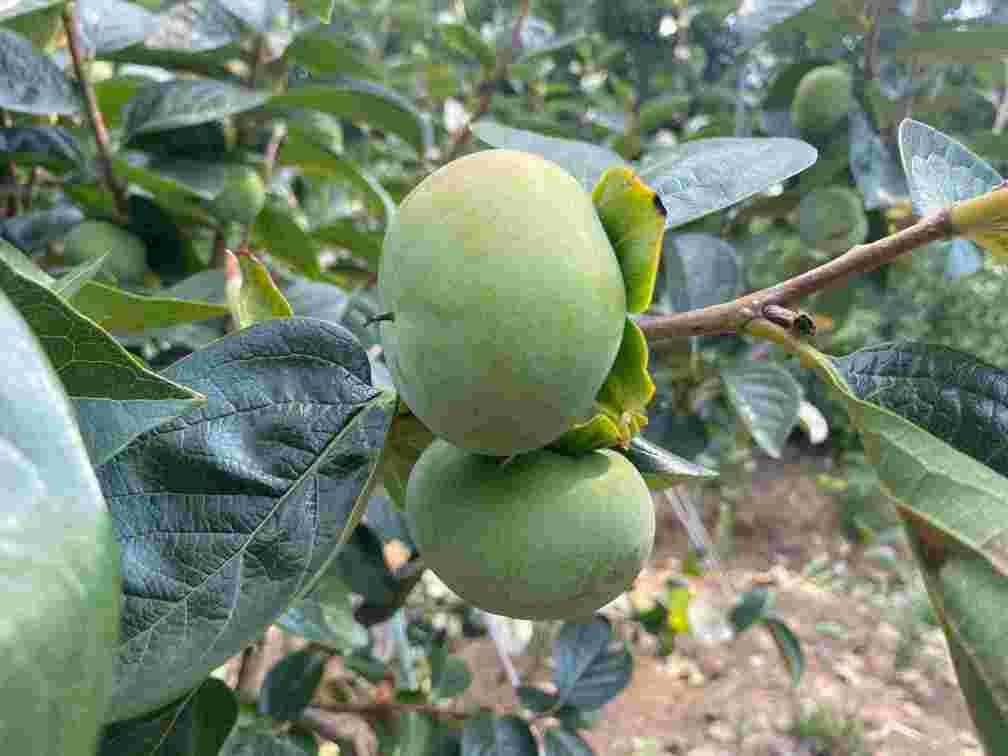

Supplement: Supplementary file 1 [file DataSheet1.zip › 2022-07-22 183454(51).jpg]

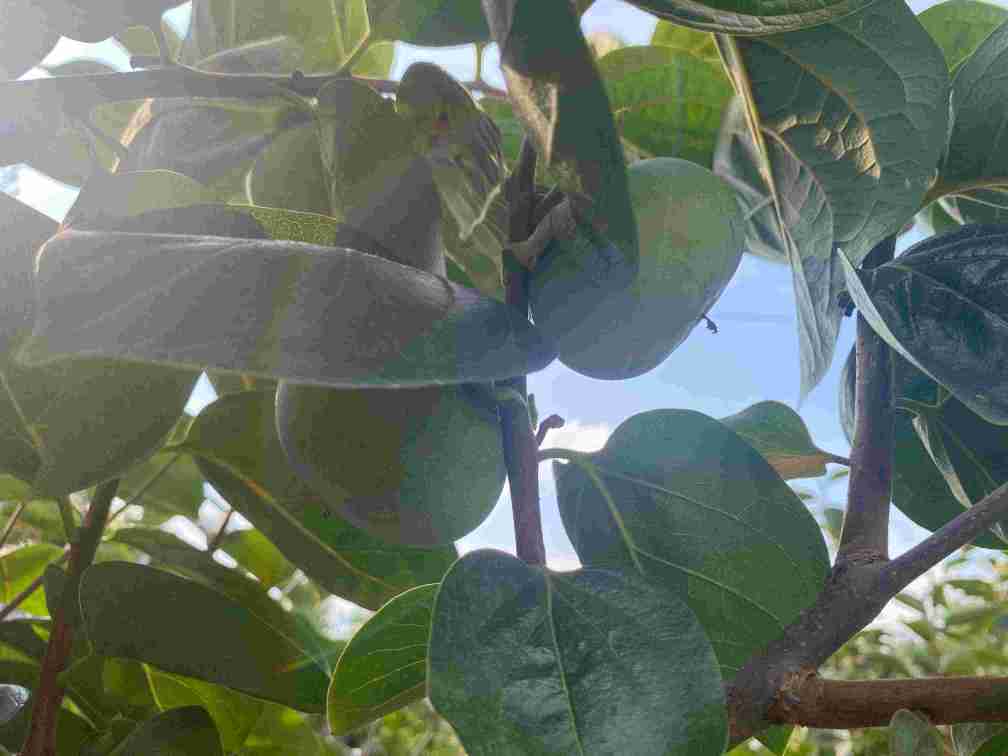

Supplement: Supplementary file 1 [file DataSheet1.zip › 2022-07-22 183454(52).jpg]

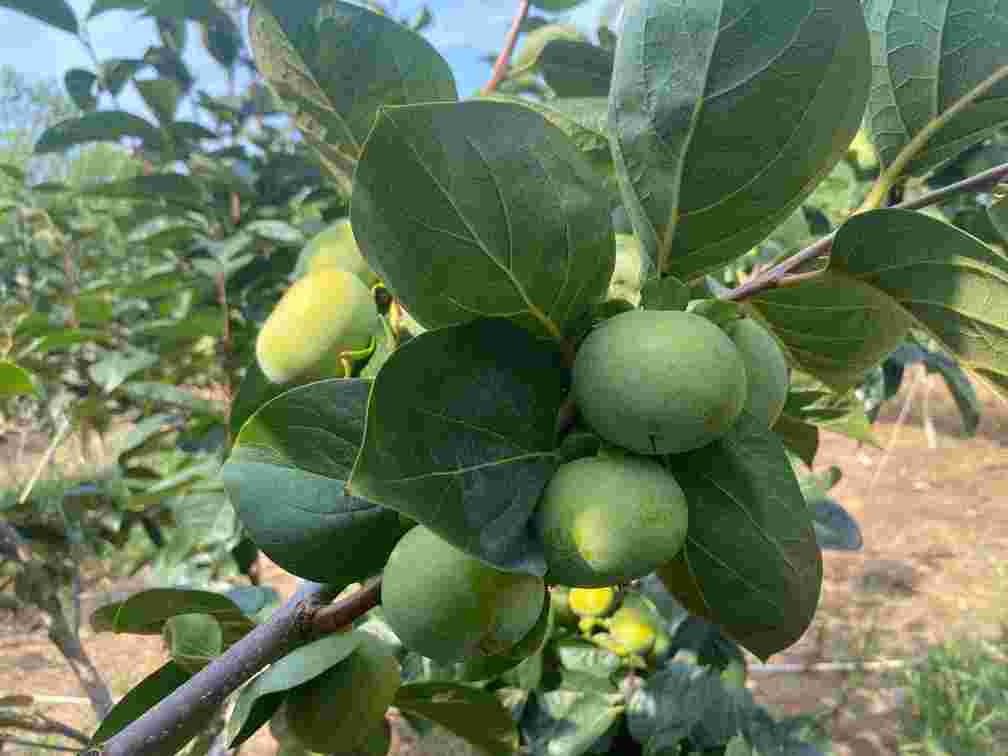

Supplement: Supplementary file 1 [file DataSheet1.zip › 2022-07-22 183454(53).jpg]

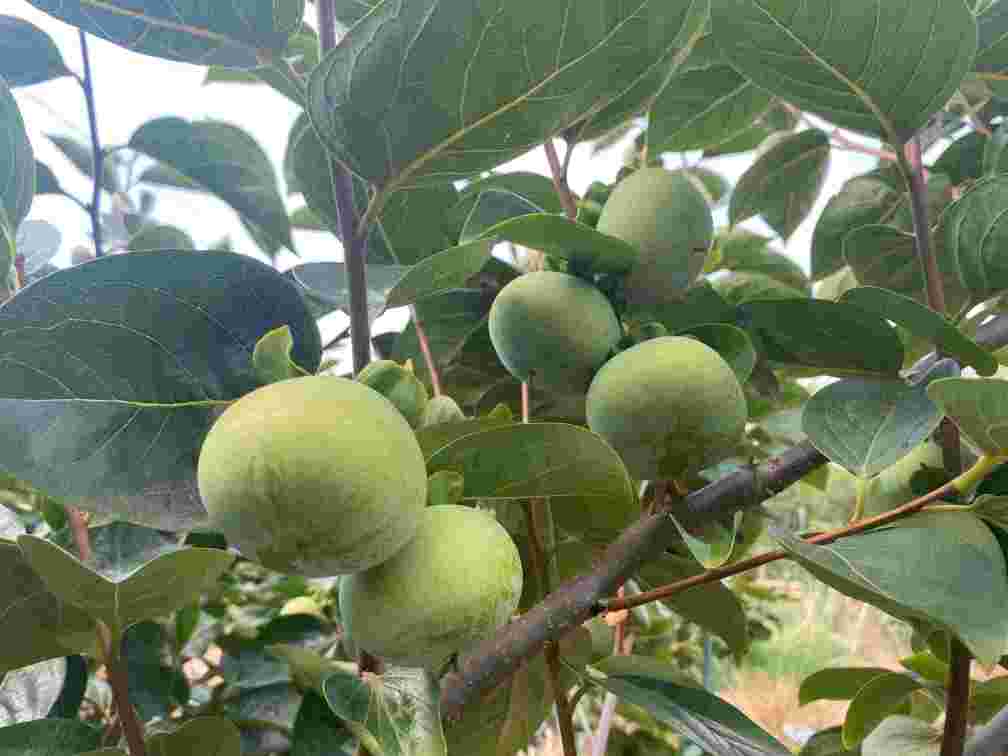

Supplement: Supplementary file 1 [file DataSheet1.zip › 2022-07-22 183454(54).jpg]

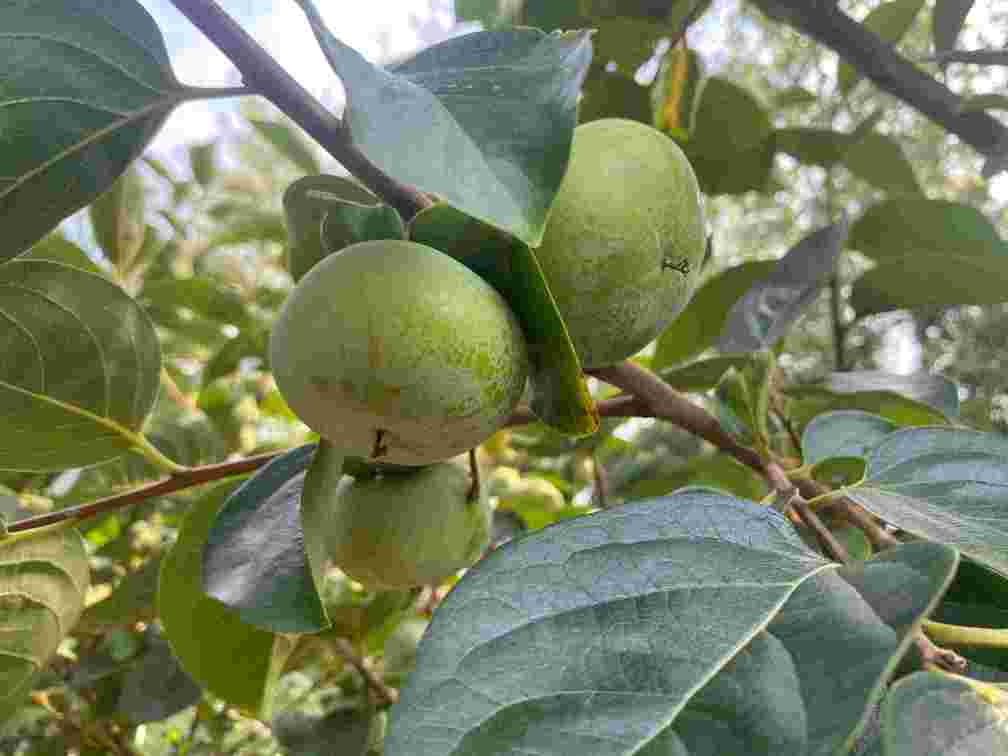

Supplement: Supplementary file 1 [file DataSheet1.zip › 2022-07-22 183454(55).jpg]

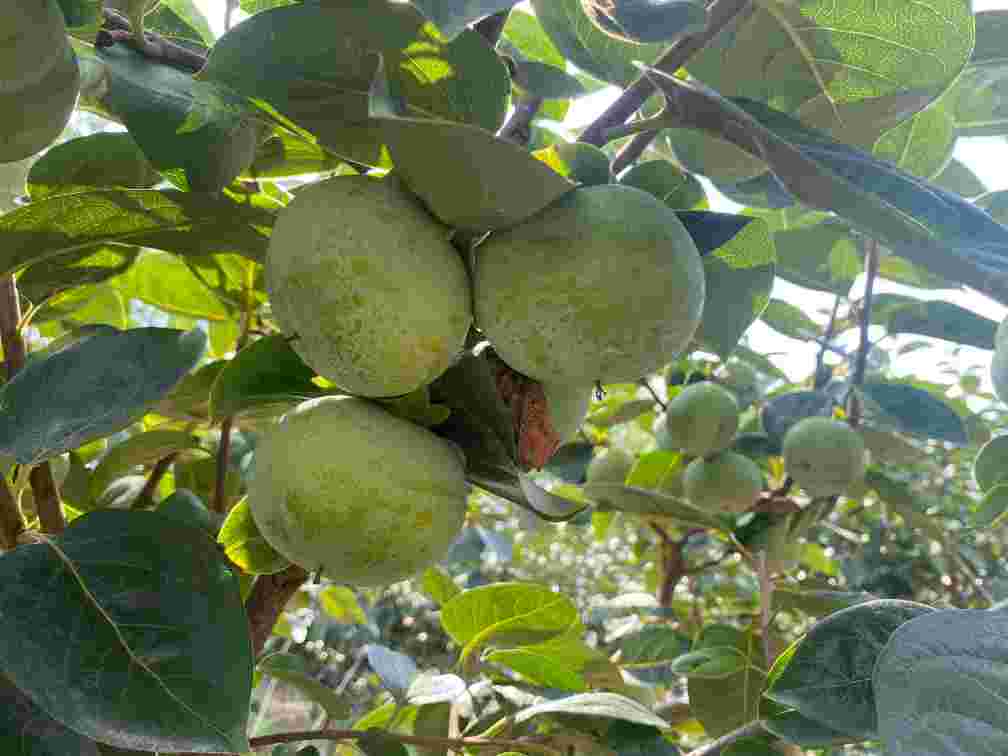

Supplement: Supplementary file 1 [file DataSheet1.zip › 2022-07-22 183454(56).jpg]

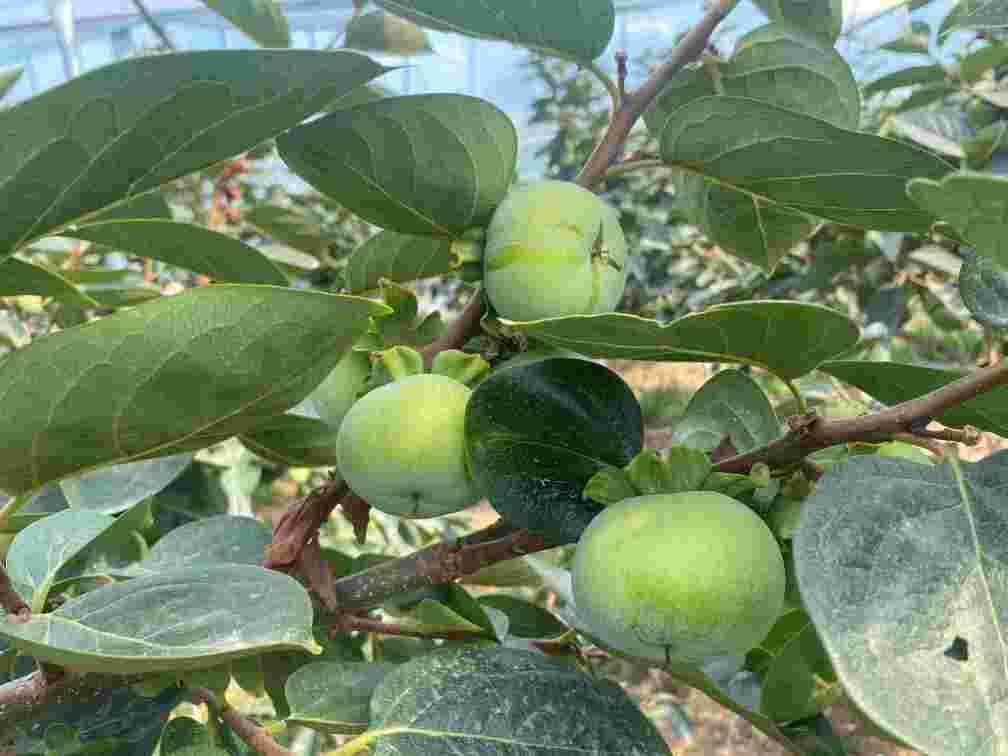

Supplement: Supplementary file 1 [file DataSheet1.zip › 2022-07-22 183454(57).jpg]

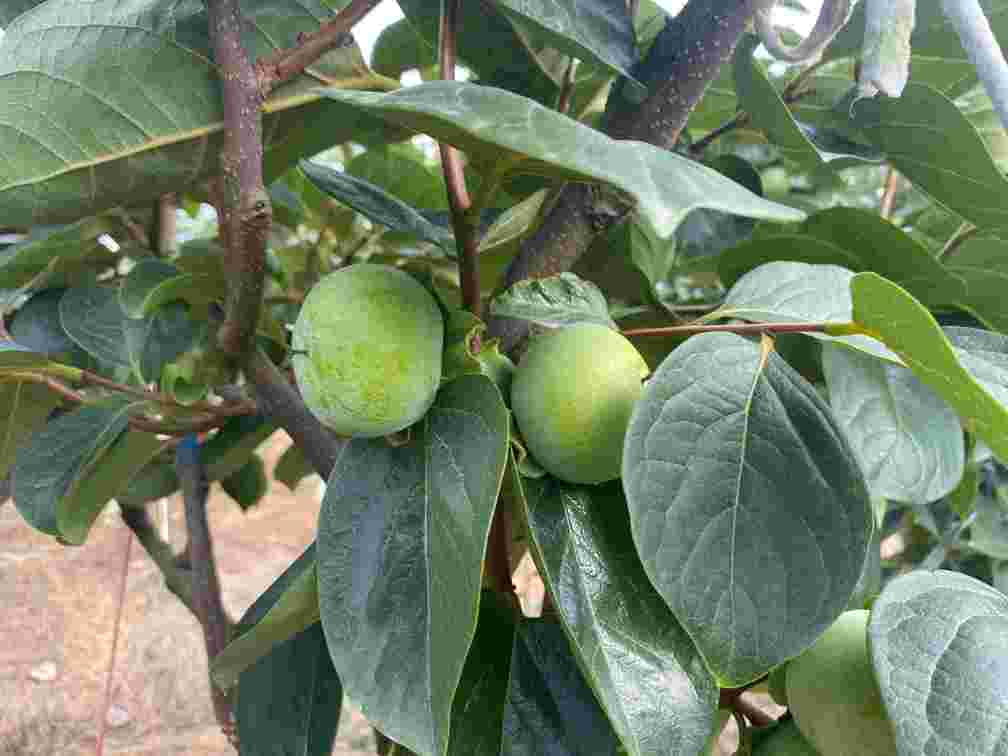

Supplement: Supplementary file 1 [file DataSheet1.zip › 2022-07-22 183454(58).jpg]

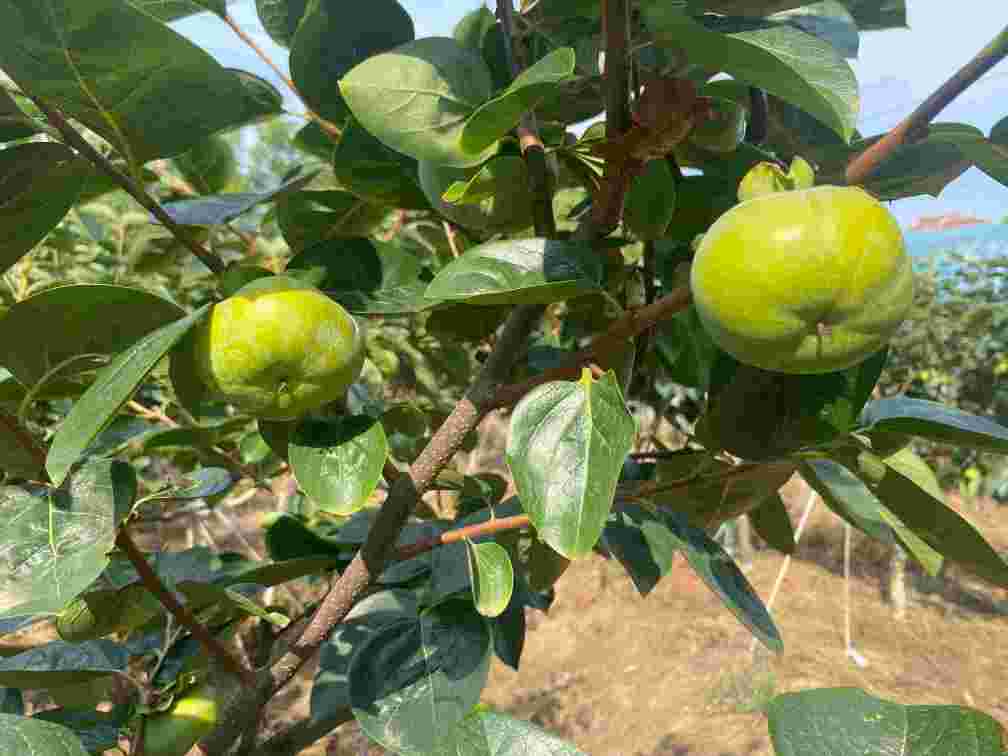

Supplement: Supplementary file 1 [file DataSheet1.zip › 2022-07-22 183454(59).jpg]

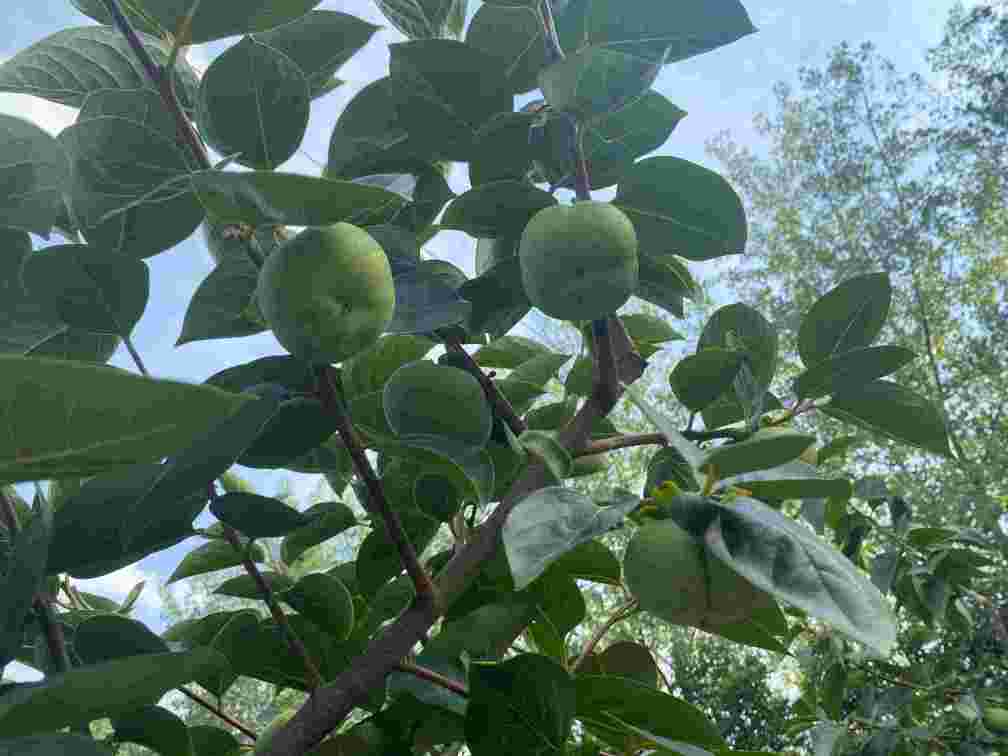

Supplement: Supplementary file 1 [file DataSheet1.zip › 2022-07-22 183454(60).jpg]

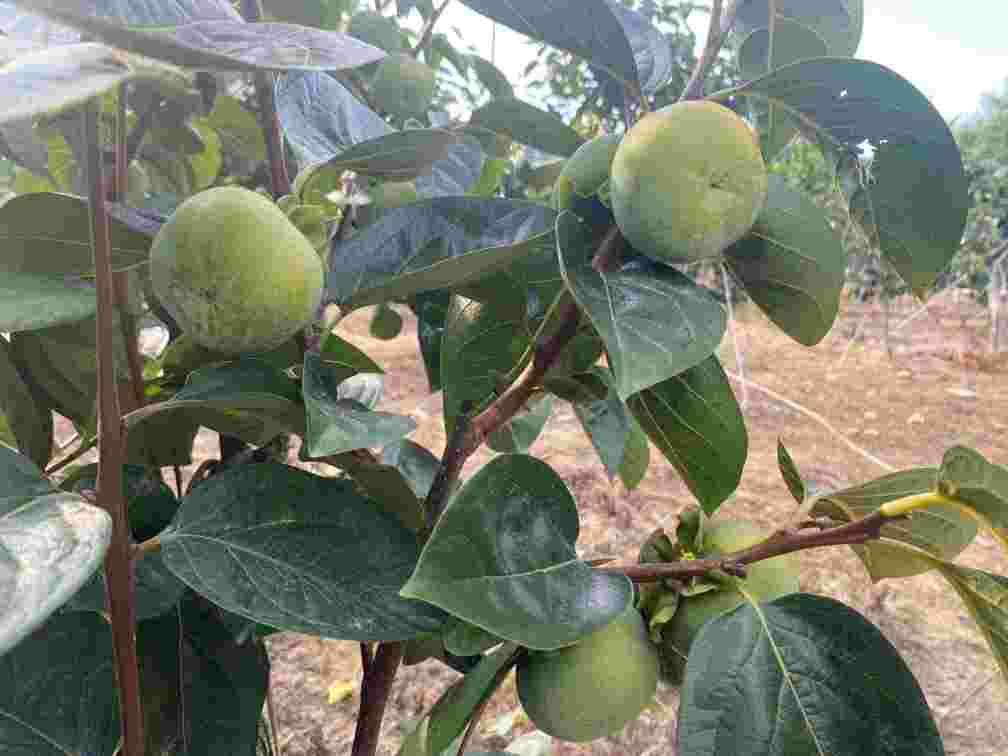

Supplement: Supplementary file 1 [file DataSheet1.zip › 2022-07-22 183454(60)_20220722_183603.jpg]

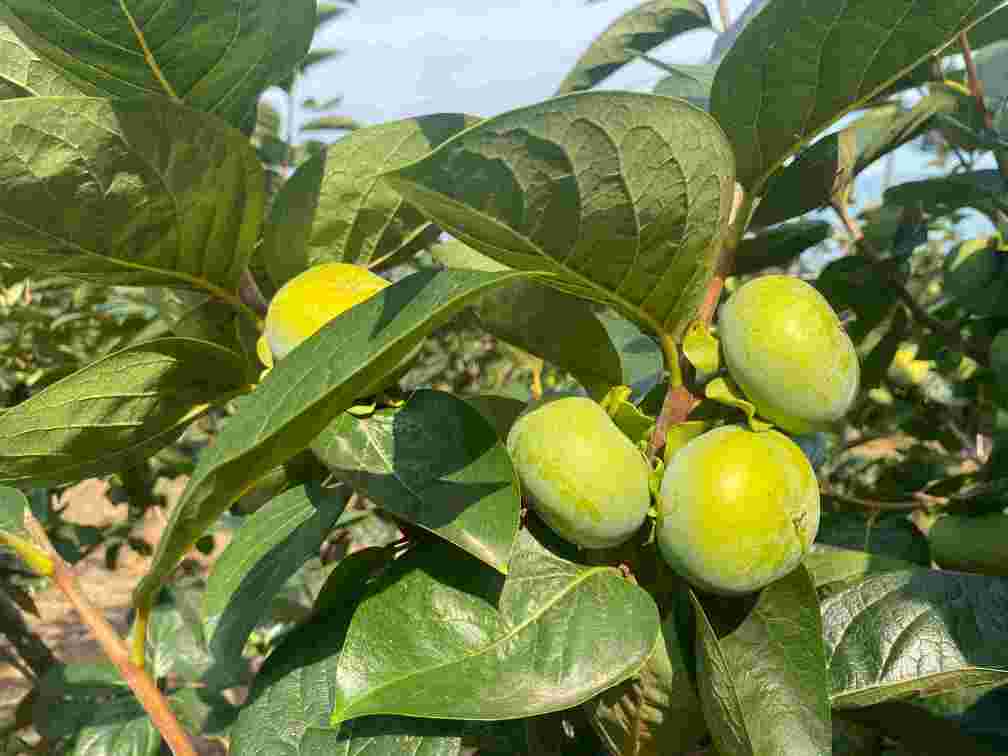

Supplement: Supplementary file 1 [file DataSheet1.zip › 2022-07-22 183454(60)_20220722_183603_20220722_183603.jpg]

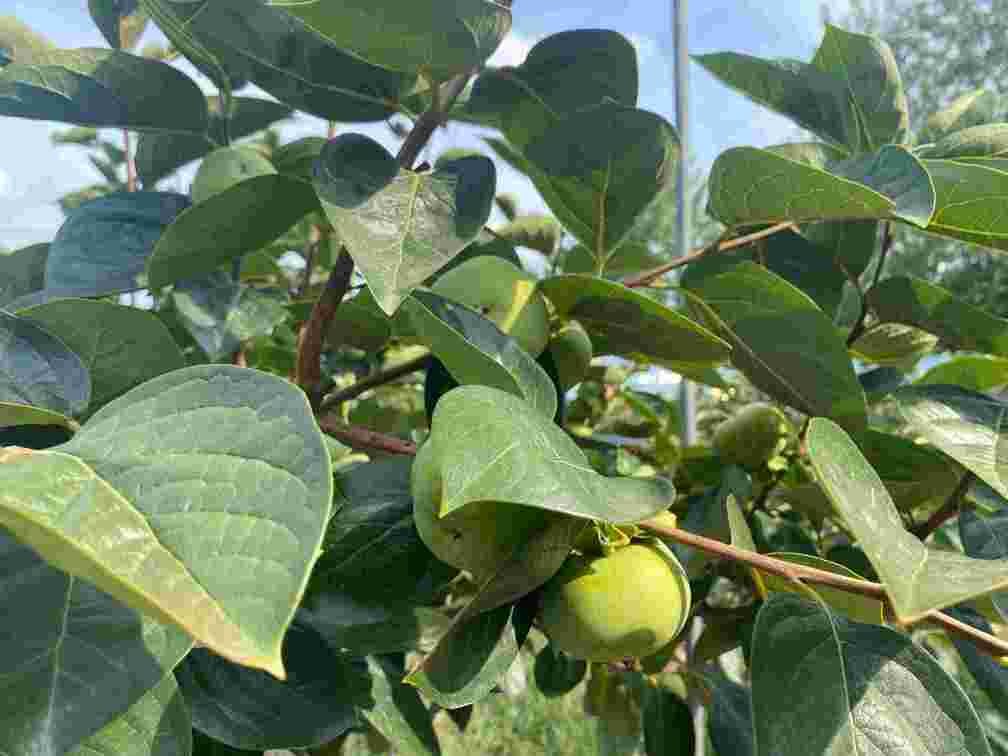

Supplement: Supplementary file 1 [file DataSheet1.zip › 2022-07-22 183454(61).jpg]

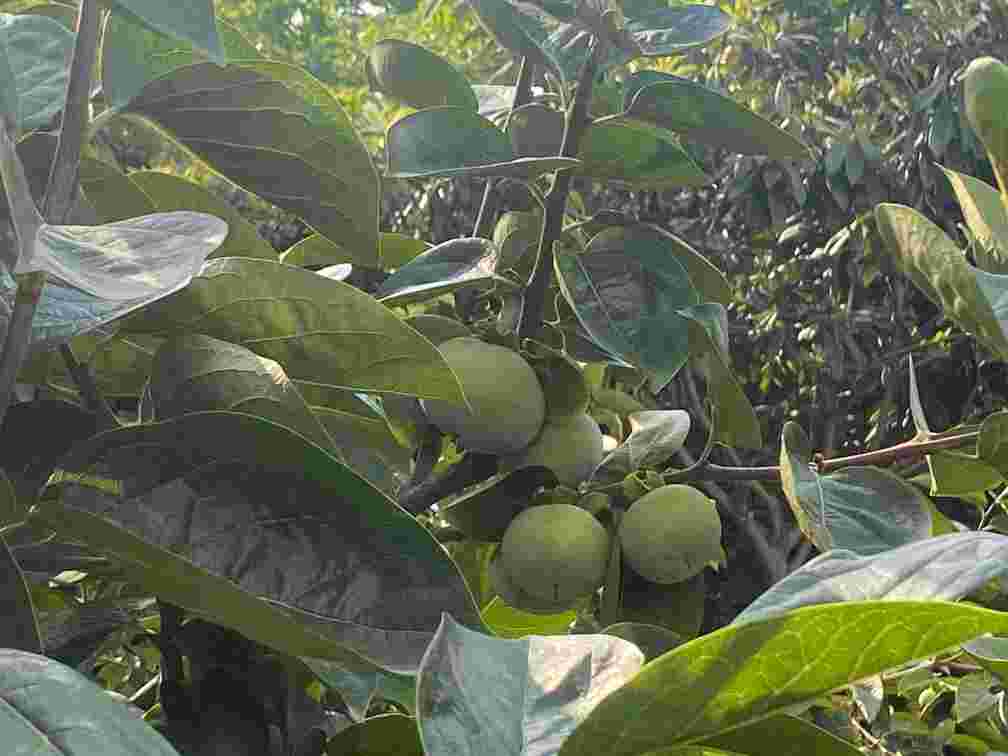

Supplement: Supplementary file 1 [file DataSheet1.zip › 2022-07-22 183454(62).jpg]

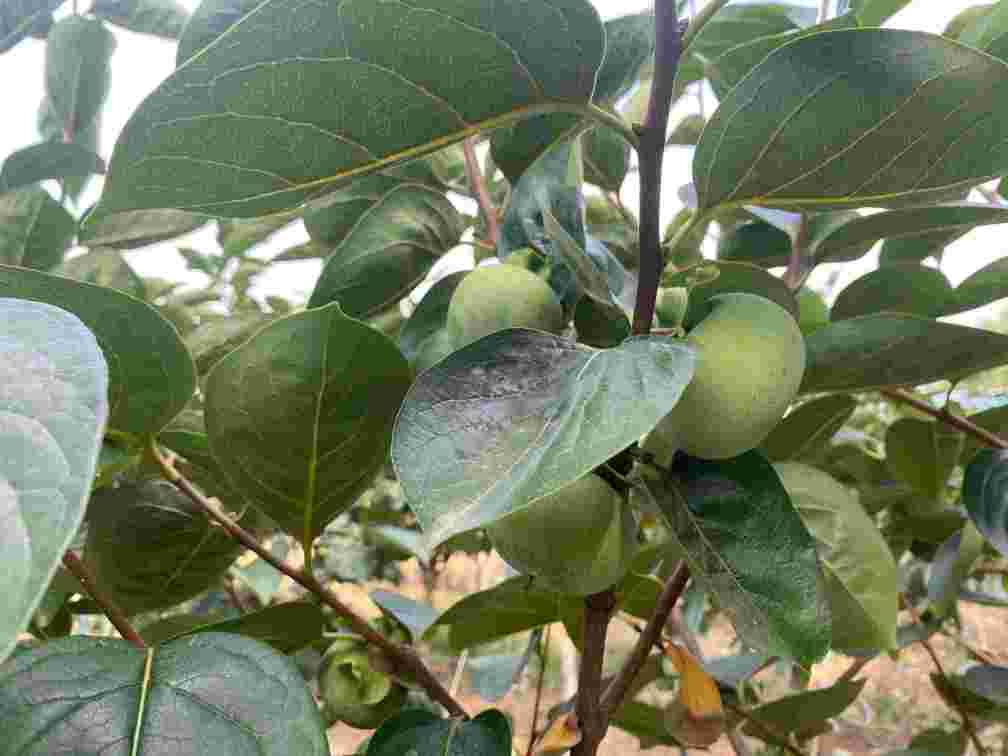

Supplement: Supplementary file 1 [file DataSheet1.zip › 2022-07-22 183454(63).jpg]

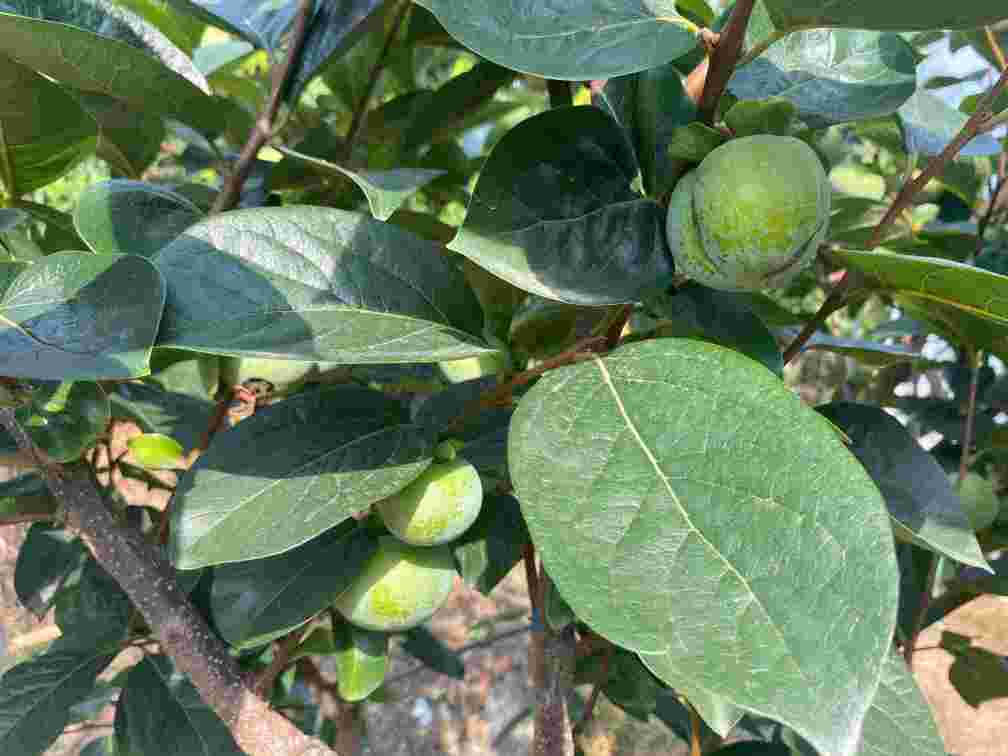

Supplement: Supplementary file 1 [file DataSheet1.zip › 2022-07-22 183454(64).jpg]

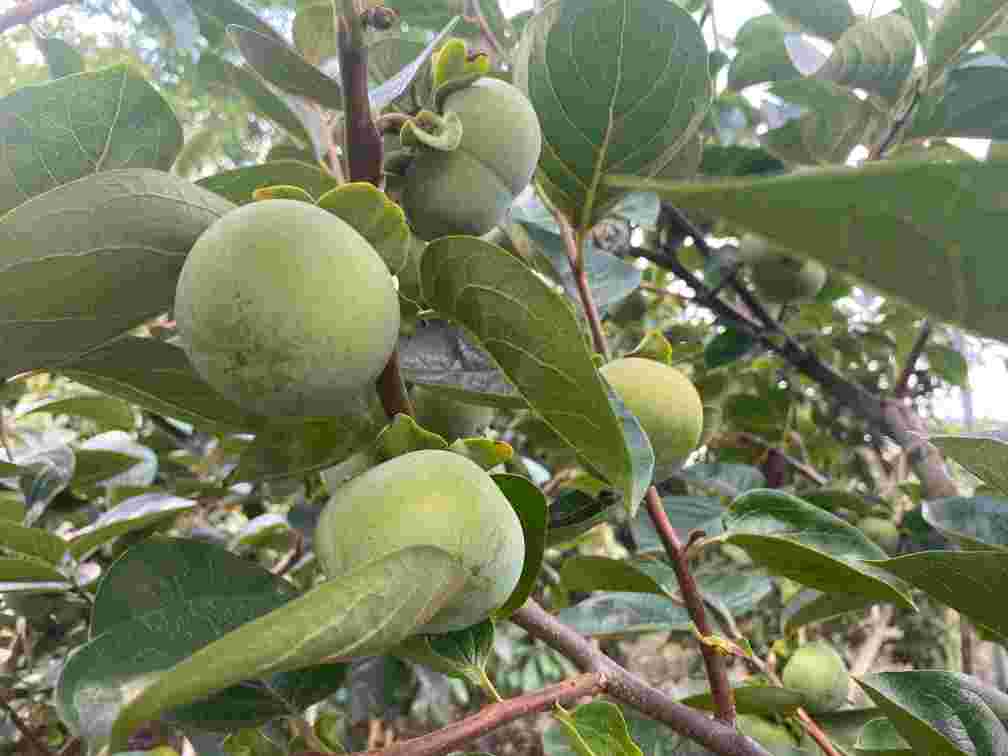

Supplement: Supplementary file 1 [file DataSheet1.zip › 2022-07-22 183454(65).jpg]

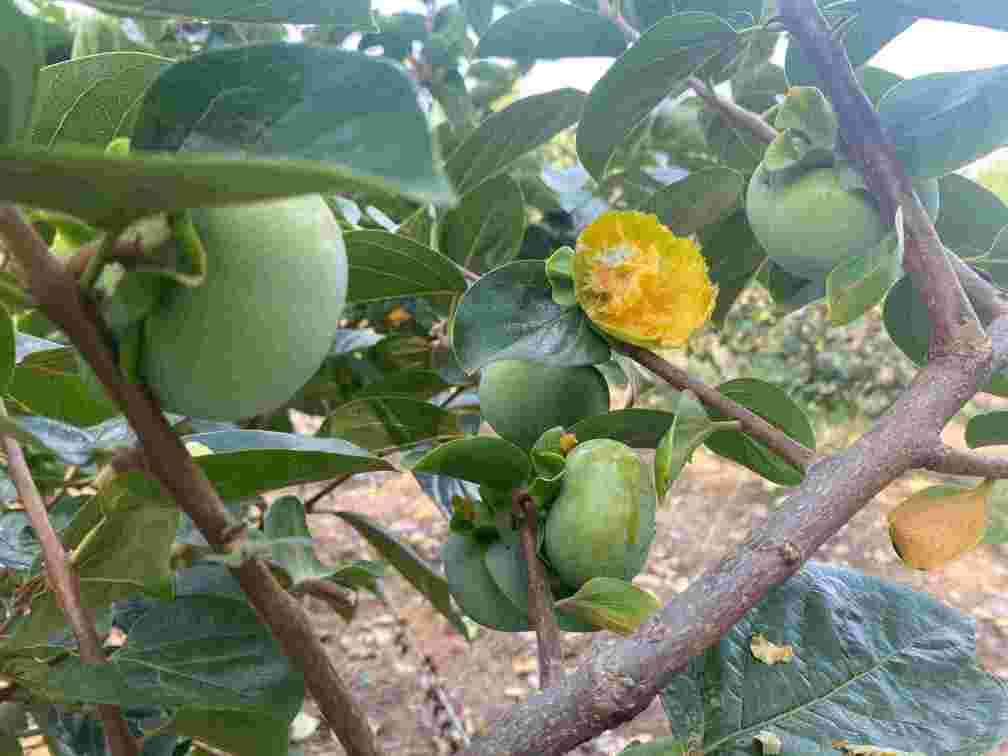

Supplement: Supplementary file 1 [file DataSheet1.zip › 2022-07-22 183454(66).jpg]

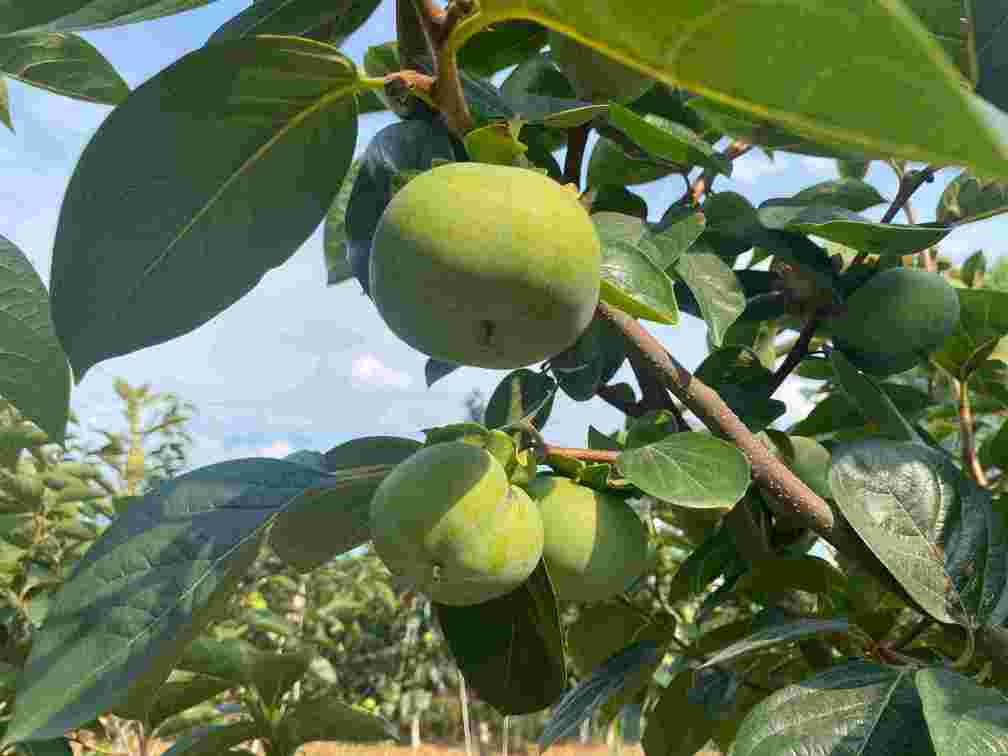

Supplement: Supplementary file 1 [file DataSheet1.zip › 2022-07-22 183454(66)_20220722_183605.jpg]

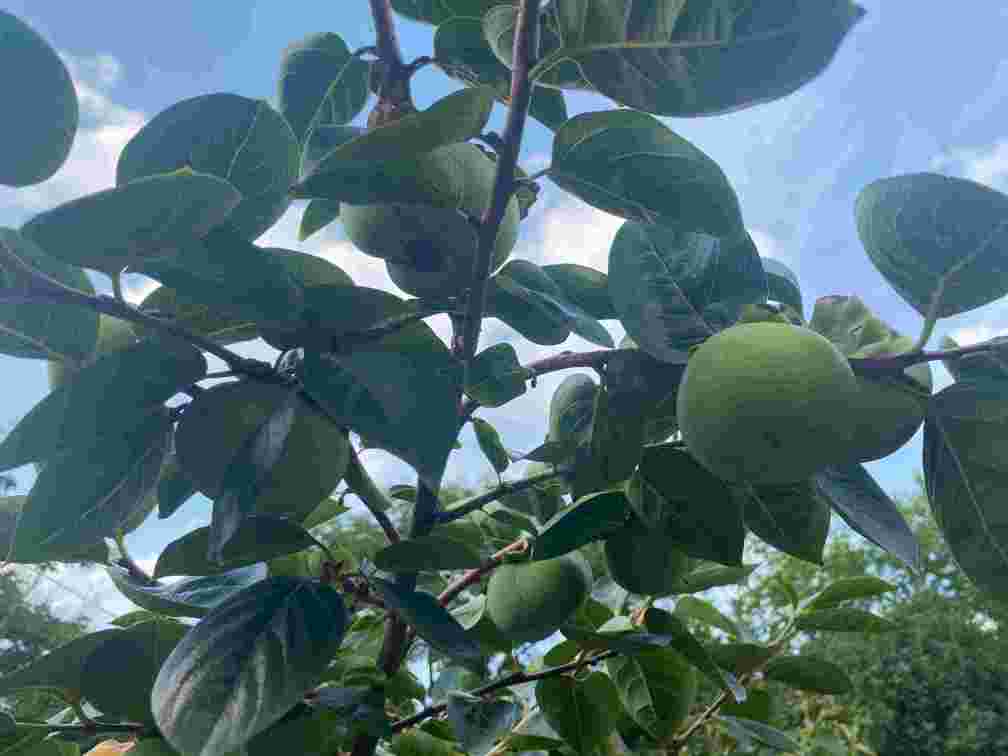

Supplement: Supplementary file 1 [file DataSheet1.zip › 2022-07-22 183454(67).jpg]

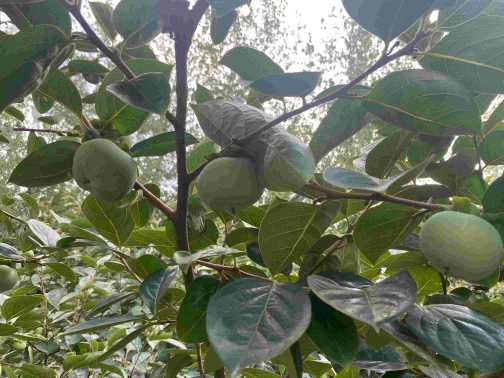

Supplement: Supplementary file 1 [file DataSheet1.zip › 2022-07-22 183454(68).jpg]

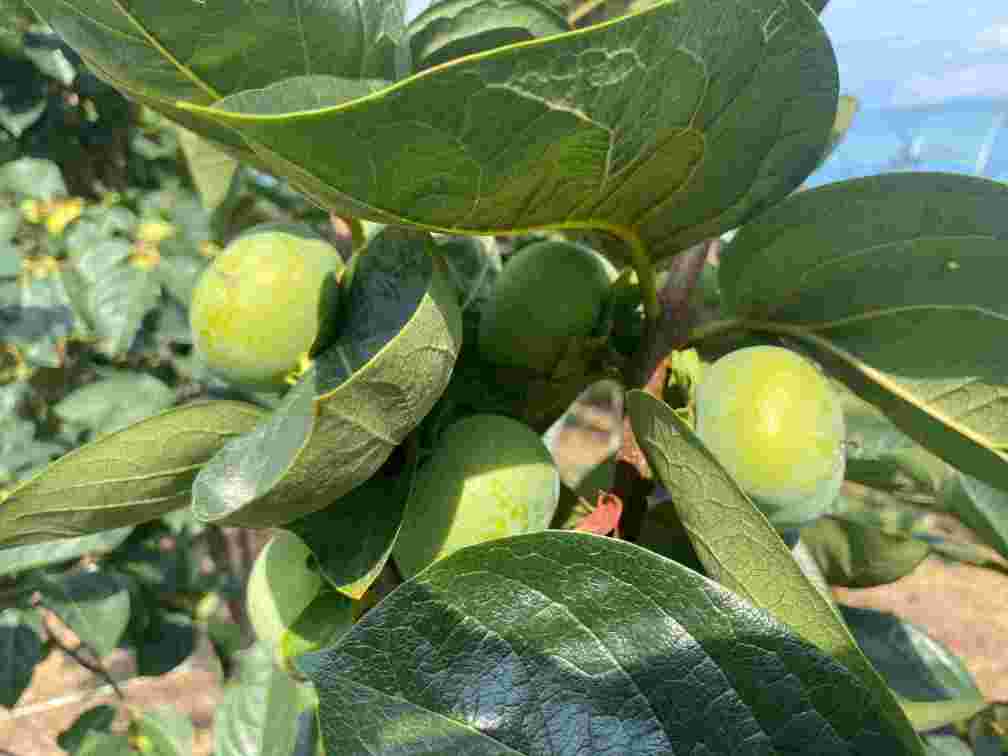

Supplement: Supplementary file 1 [file DataSheet1.zip › 2022-07-22 183454(69).jpg]

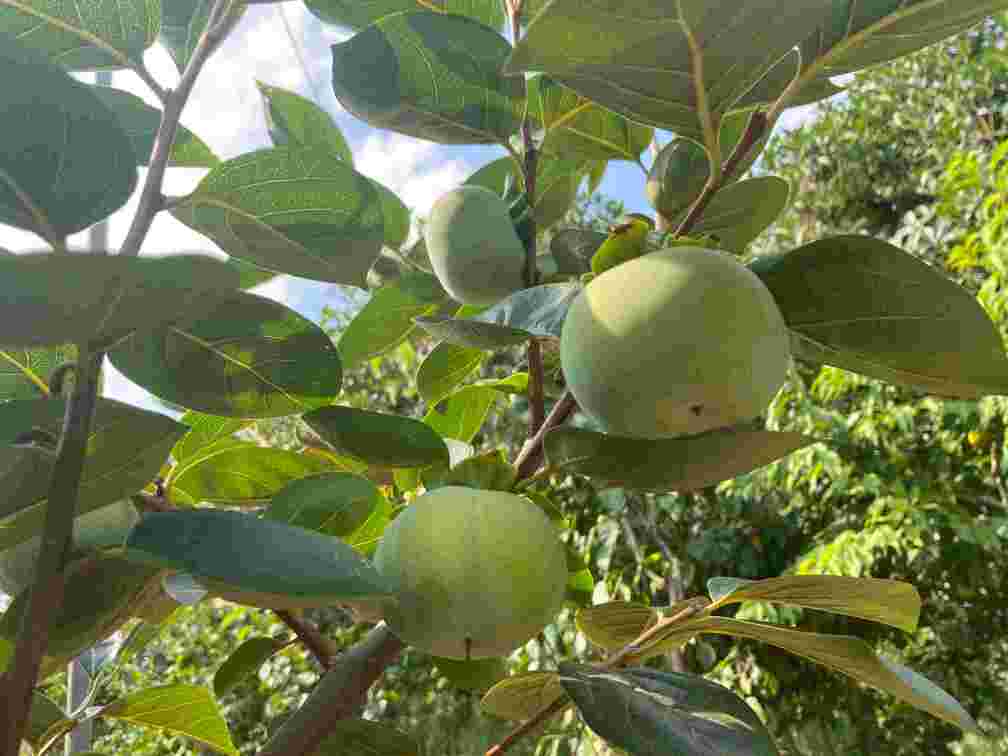

Supplement: Supplementary file 1 [file DataSheet1.zip › 2022-07-22 183454(69)_20220722_183607.jpg]

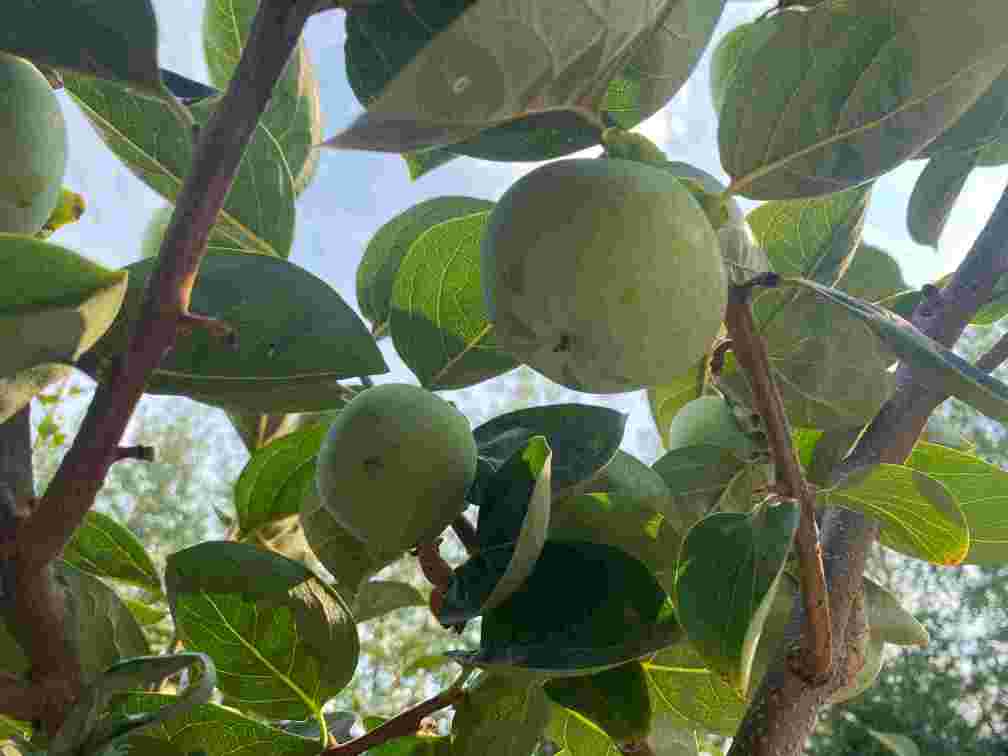

Supplement: Supplementary file 1 [file DataSheet1.zip › 2022-07-22 183454(70).jpg]

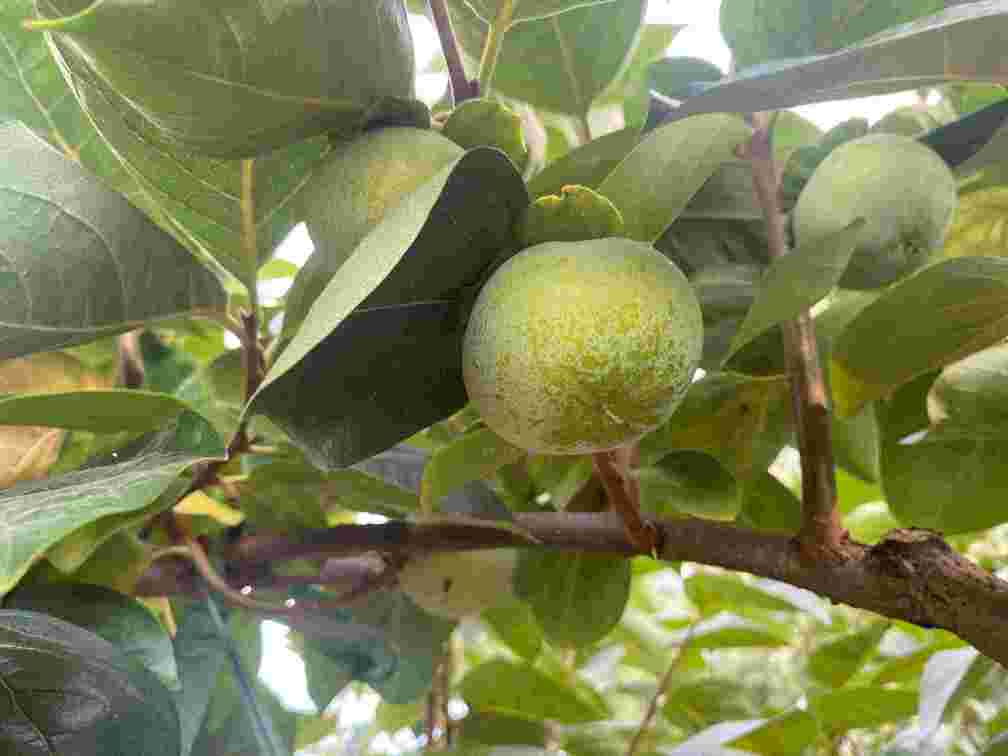

Supplement: Supplementary file 1 [file DataSheet1.zip › 2022-07-22 183454(71).jpg]

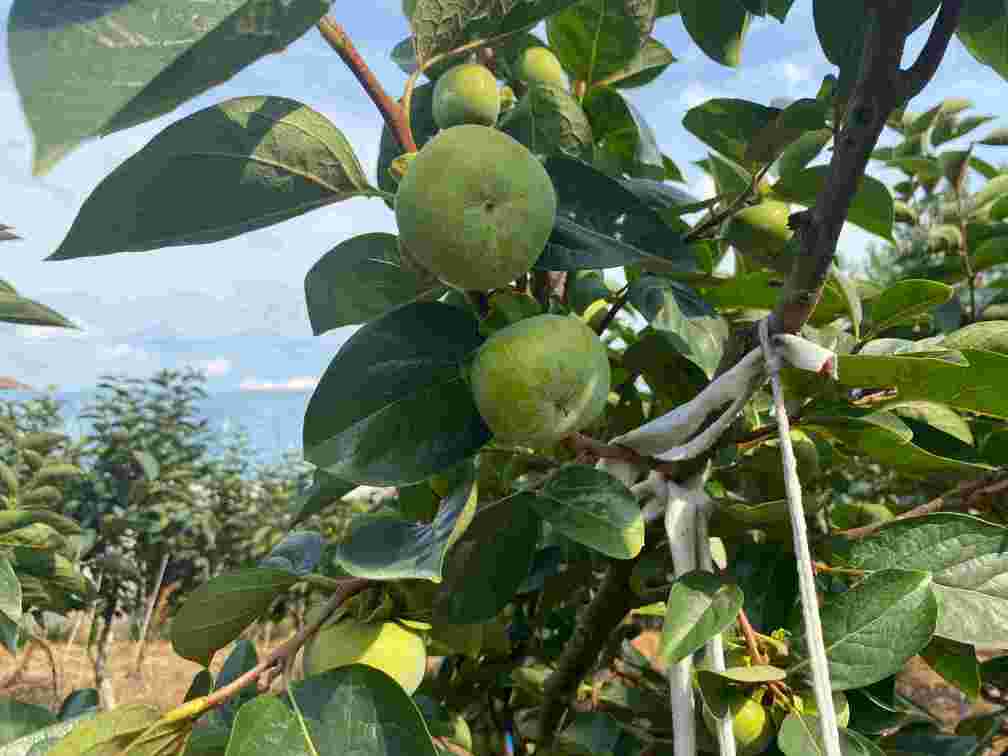

Supplement: Supplementary file 1 [file DataSheet1.zip › 2022-07-22 183454(72).jpg]

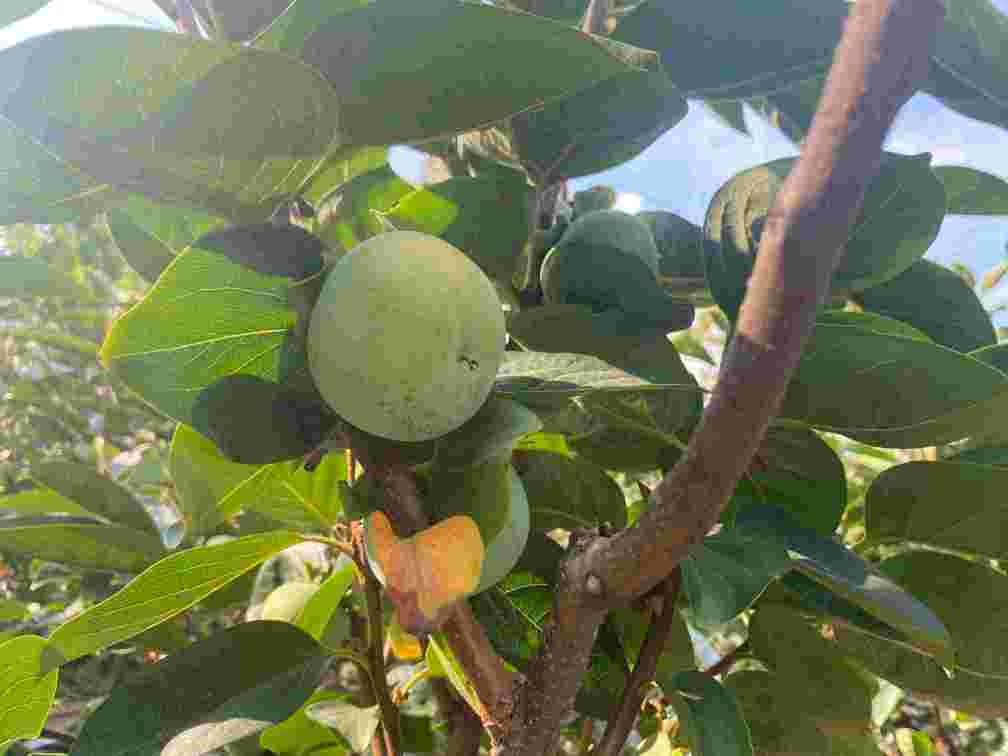

Supplement: Supplementary file 1 [file DataSheet1.zip › 2022-07-22 183454(73).jpg]

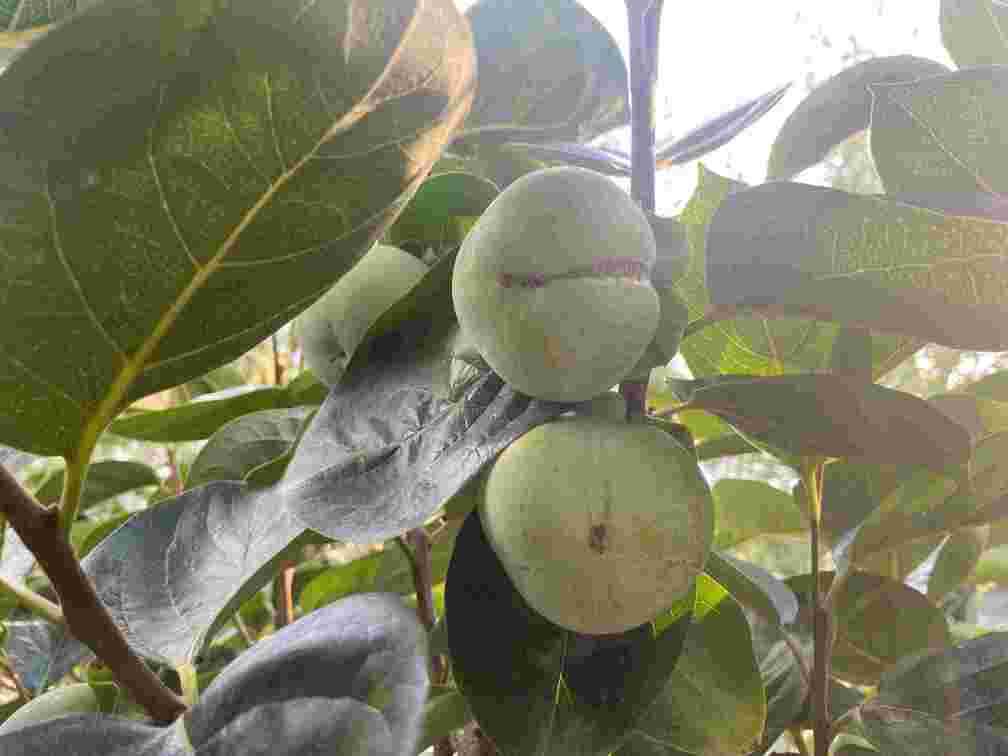

Supplement: Supplementary file 1 [file DataSheet1.zip › 2022-07-22 183454(74).jpg]

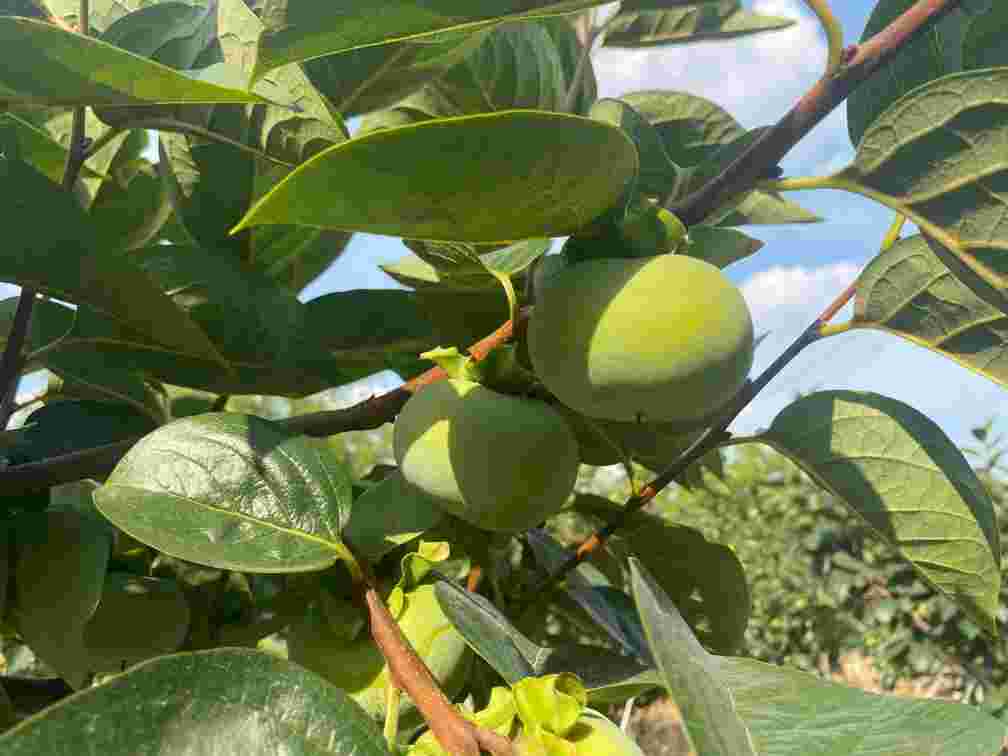

Supplement: Supplementary file 1 [file DataSheet1.zip › 2022-07-22 183454(74)_20220722_183609.jpg]

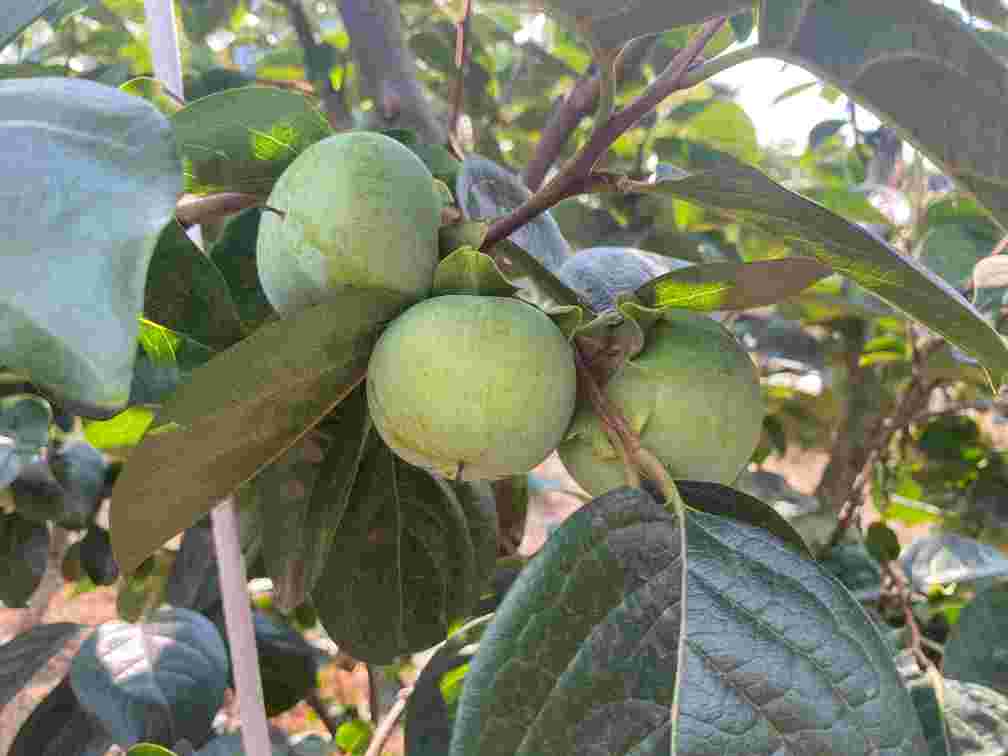

Supplement: Supplementary file 1 [file DataSheet1.zip › 2022-07-22 183454(75).jpg]

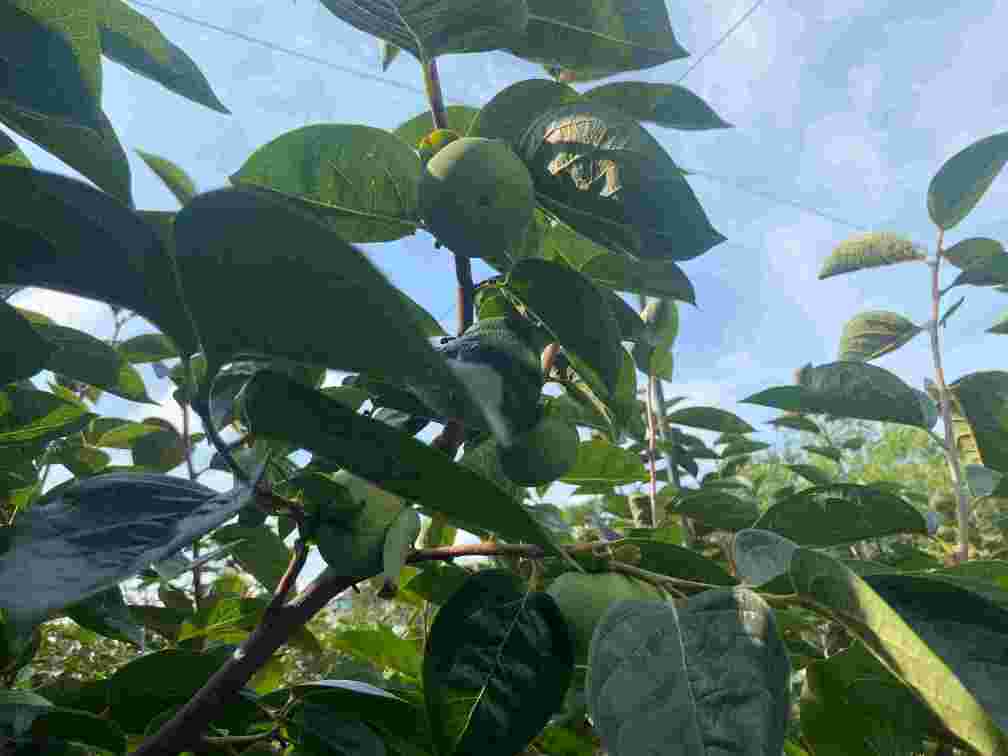

Supplement: Supplementary file 1 [file DataSheet1.zip › 2022-07-22 183454(77).jpg]

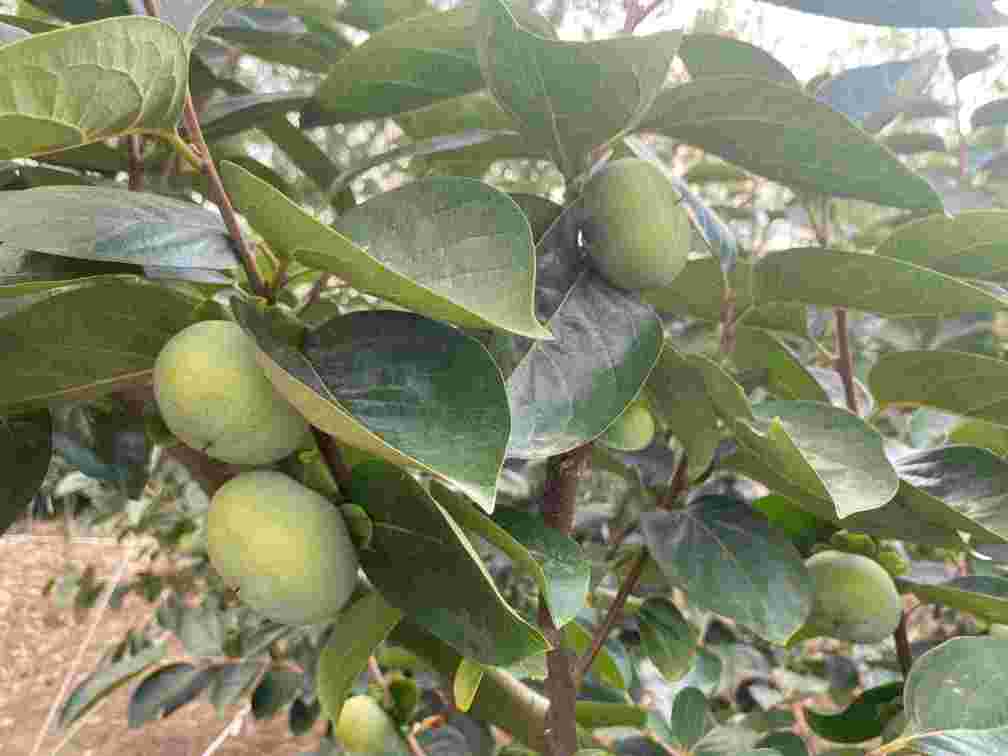

Supplement: Supplementary file 1 [file DataSheet1.zip › 2022-07-22 183454(78).jpg]

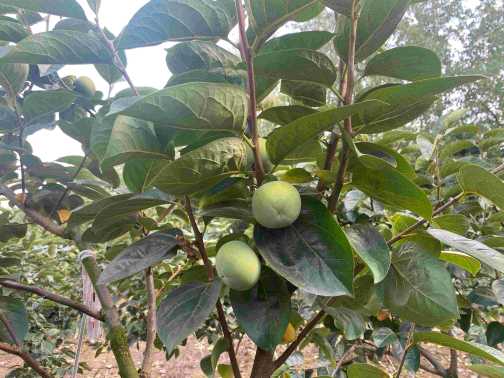

Supplement: Supplementary file 1 [file DataSheet1.zip › 2022-07-22 183454(79).jpg]

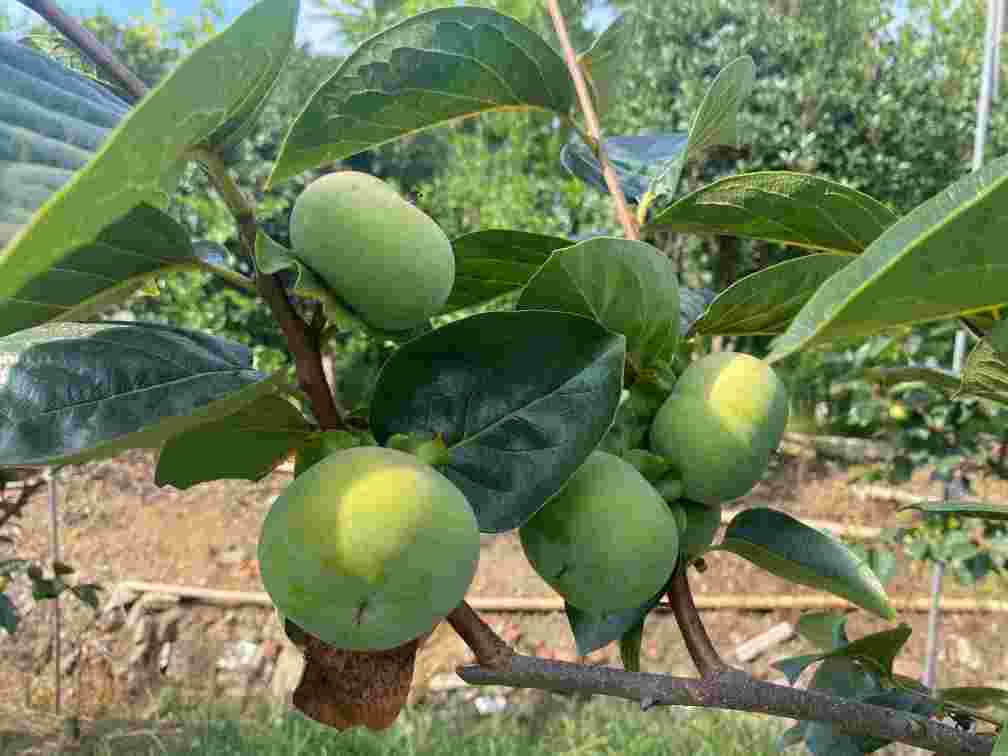

Supplement: Supplementary file 1 [file DataSheet1.zip › 2022-07-22 183454(80).jpg]

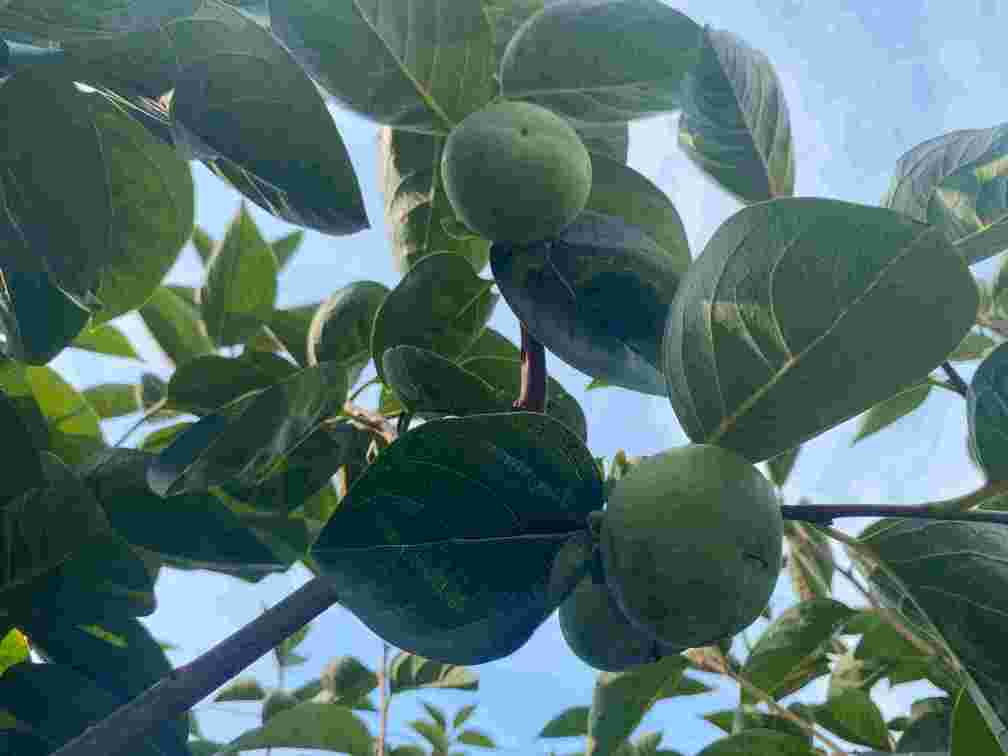

Supplement: Supplementary file 1 [file DataSheet1.zip › 2022-07-22 183454(81).jpg]

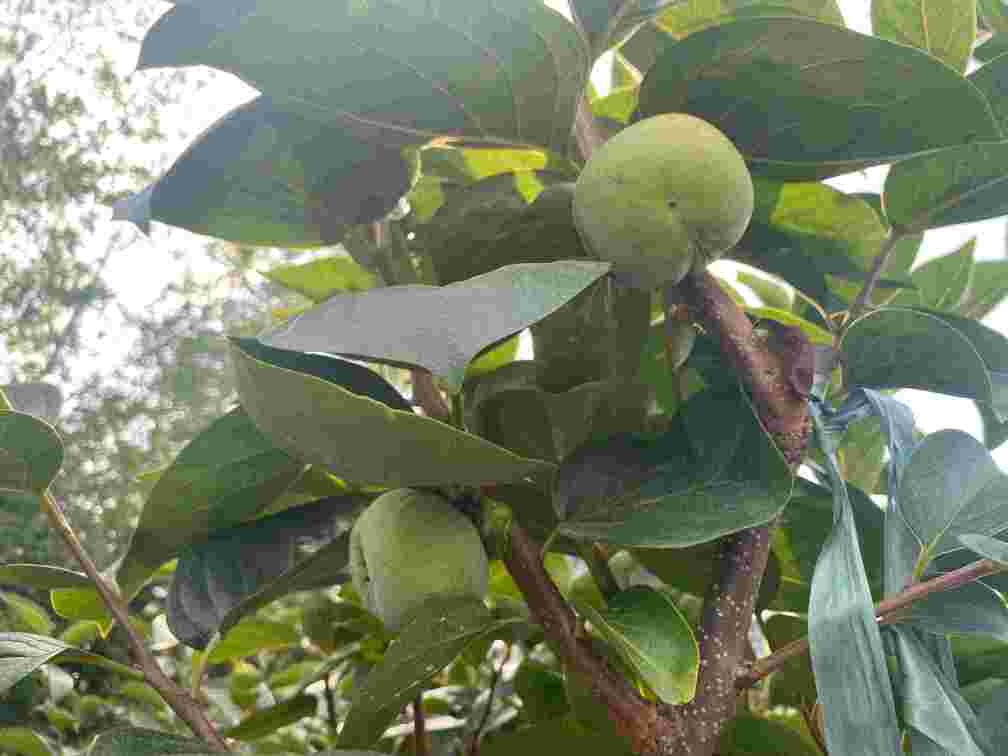

Supplement: Supplementary file 1 [file DataSheet1.zip › 2022-07-22 183454(82).jpg]

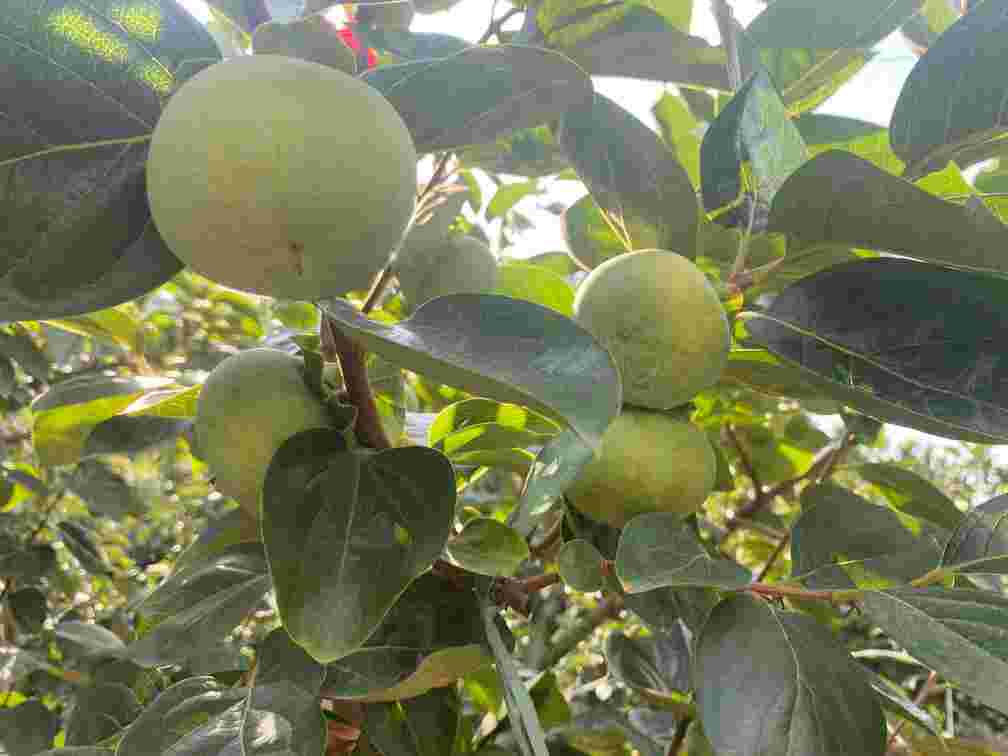

Supplement: Supplementary file 1 [file DataSheet1.zip › 2022-07-22 183454(83).jpg]

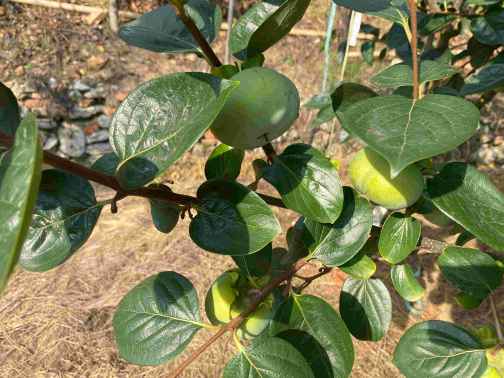

Supplement: Supplementary file 1 [file DataSheet1.zip › 2022-07-22 183454(84).jpg]

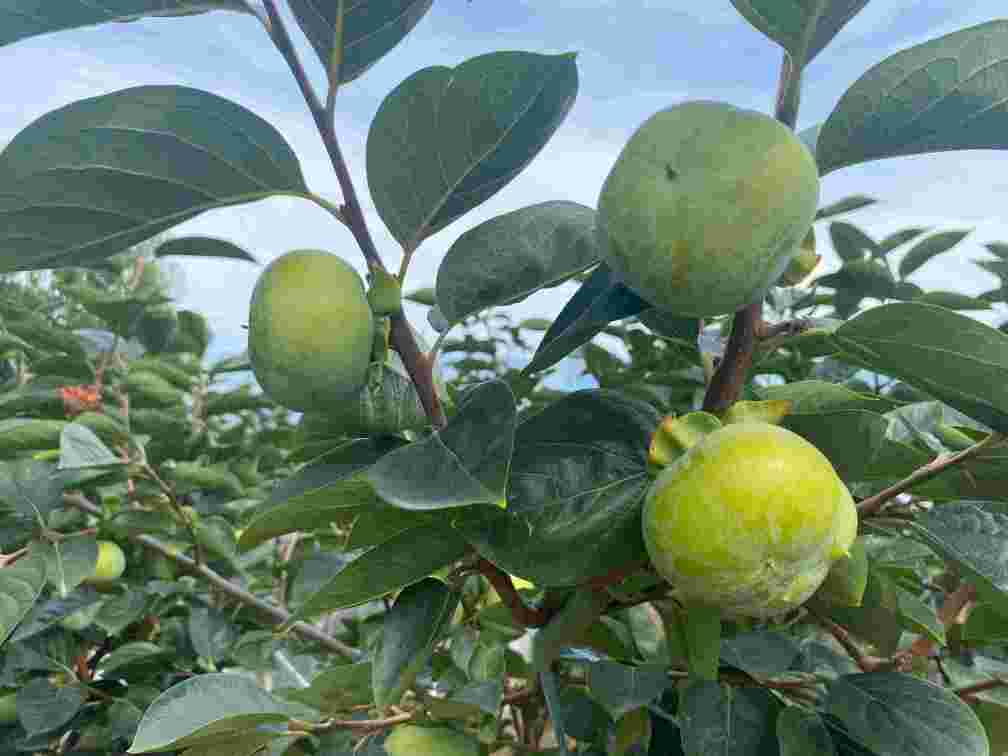

Supplement: Supplementary file 1 [file DataSheet1.zip › 2022-07-22 183454(85).jpg]

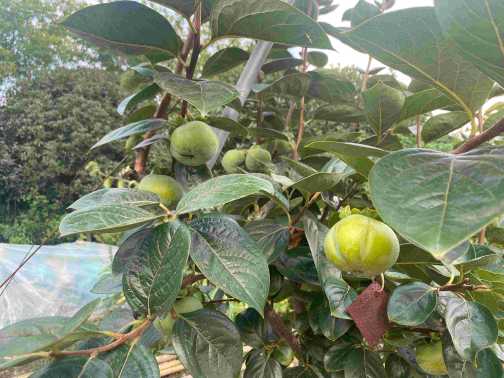

Supplement: Supplementary file 1 [file DataSheet1.zip › 2022-07-22 183454(87).jpg]

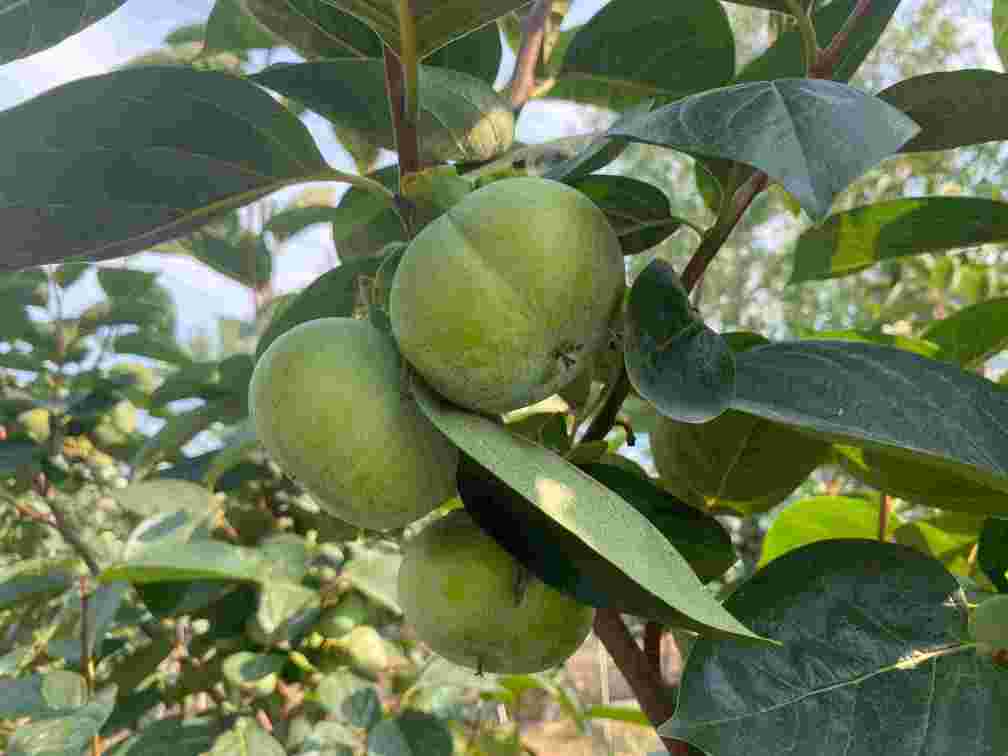

Supplement: Supplementary file 1 [file DataSheet1.zip › 2022-07-22 183454(88).jpg]

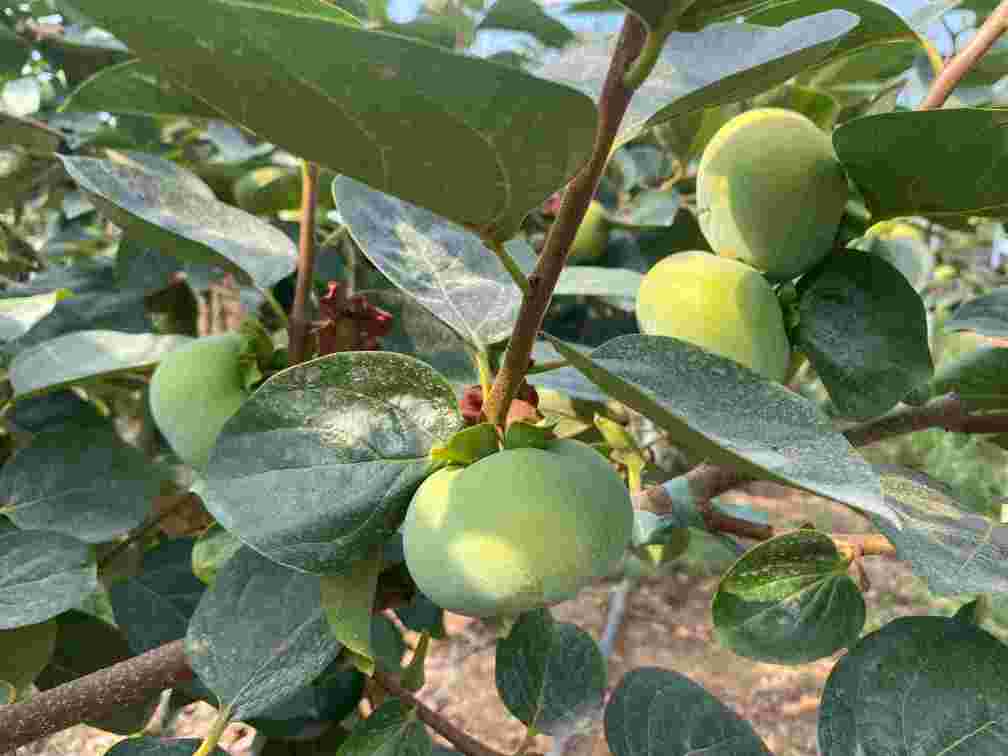

Supplement: Supplementary file 1 [file DataSheet1.zip › 2022-07-22 183454(89).jpg]

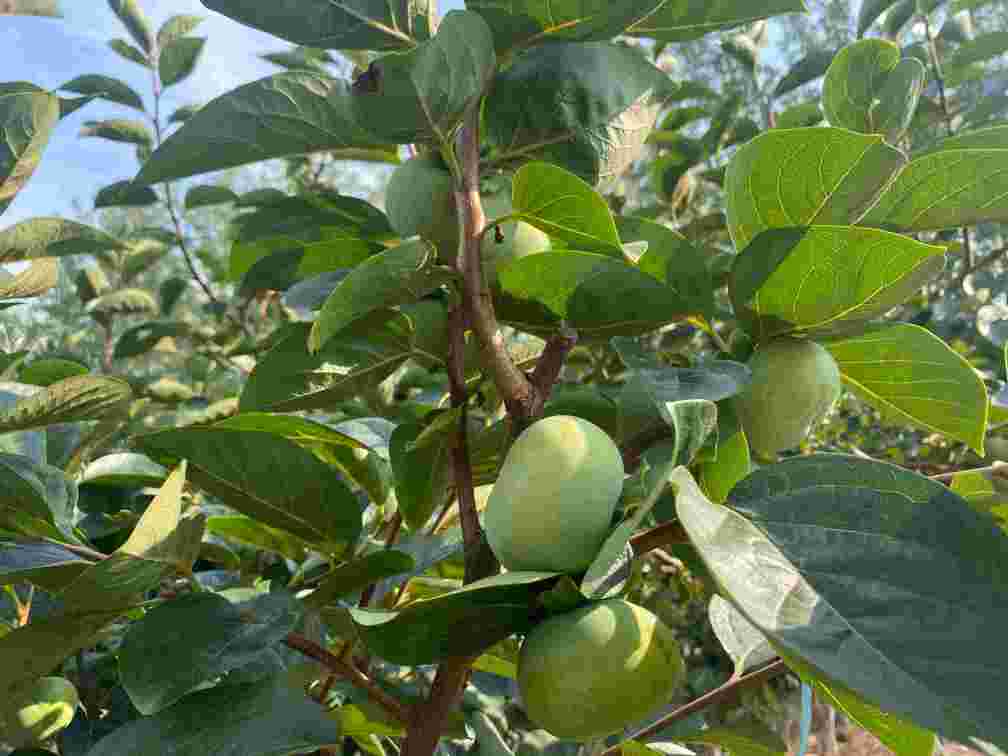

Supplement: Supplementary file 1 [file DataSheet1.zip › 2022-07-22 183454(9).jpg]

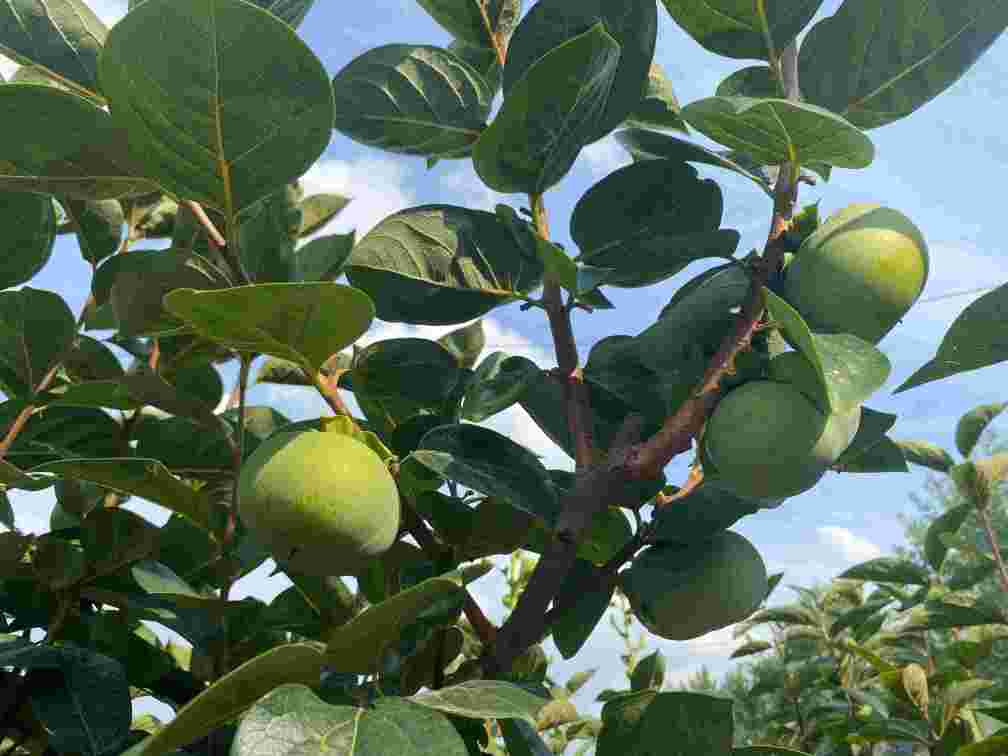

Supplement: Supplementary file 1 [file DataSheet1.zip › 2022-07-22 183454(90).jpg]

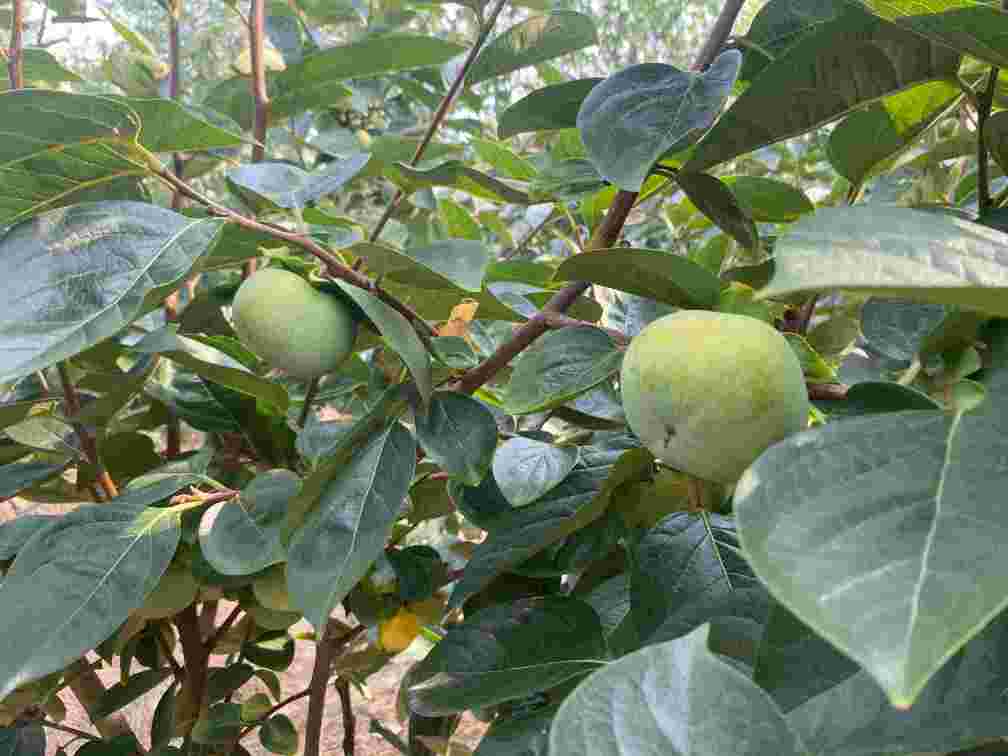

Supplement: Supplementary file 1 [file DataSheet1.zip › 2022-07-22 183454(91).jpg]

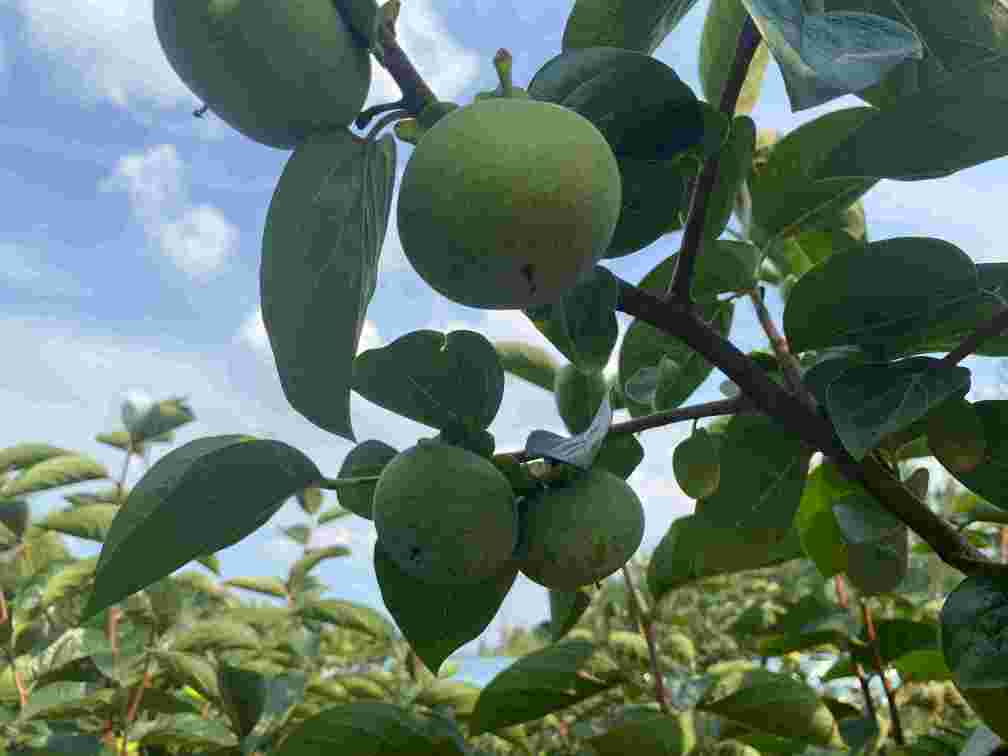

Supplement: Supplementary file 1 [file DataSheet1.zip › 2022-07-22 183454(92).jpg]

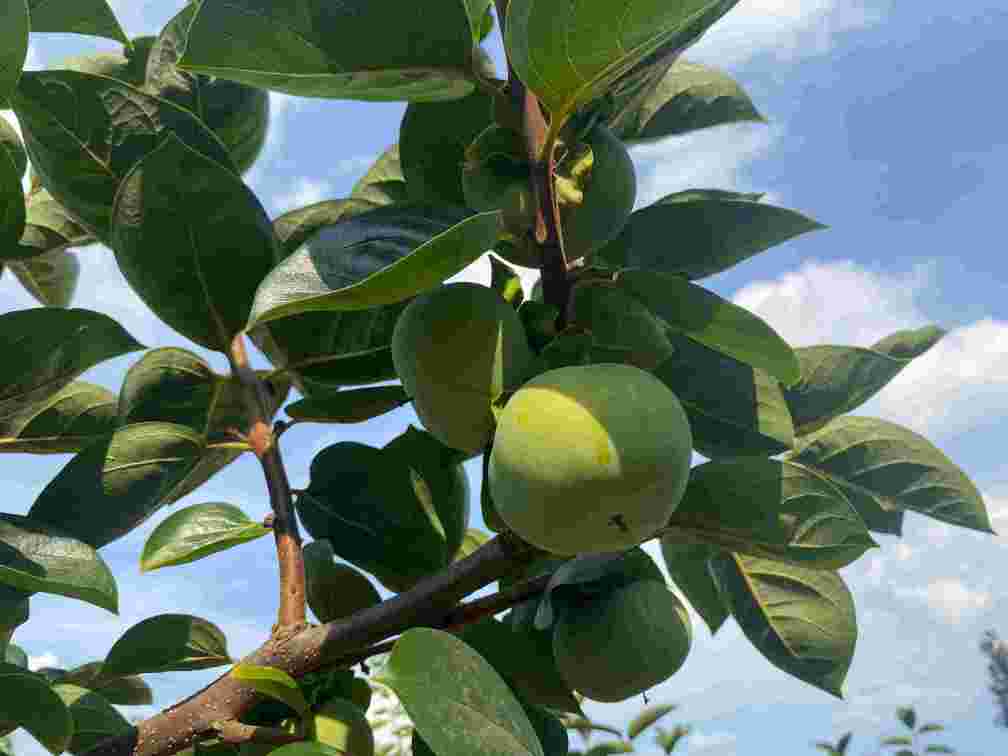

Supplement: Supplementary file 1 [file DataSheet1.zip › 2022-07-22 183454(93).jpg]

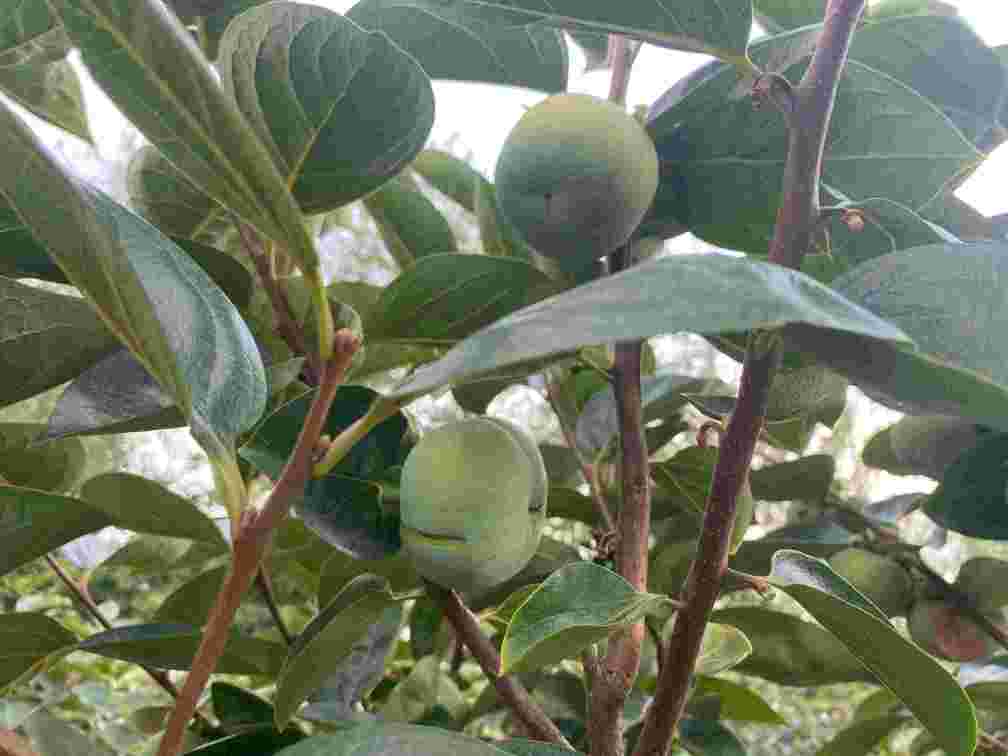

Supplement: Supplementary file 1 [file DataSheet1.zip › 2022-07-22 183454(94).jpg]

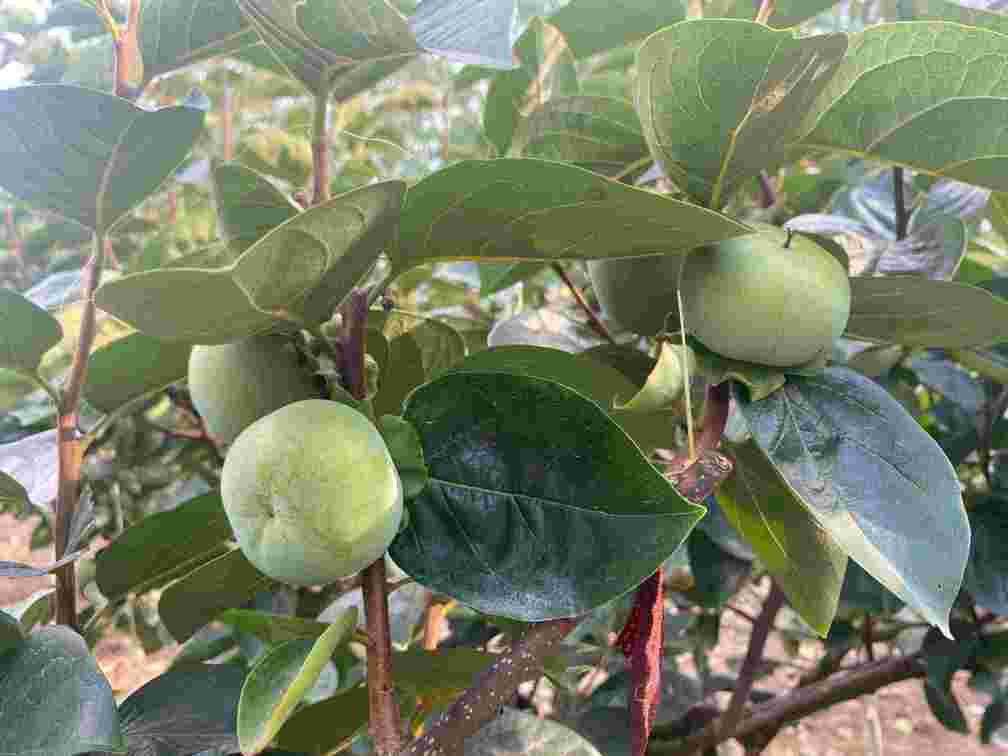

Supplement: Supplementary file 1 [file DataSheet1.zip › 2022-07-22 183454(95).jpg]

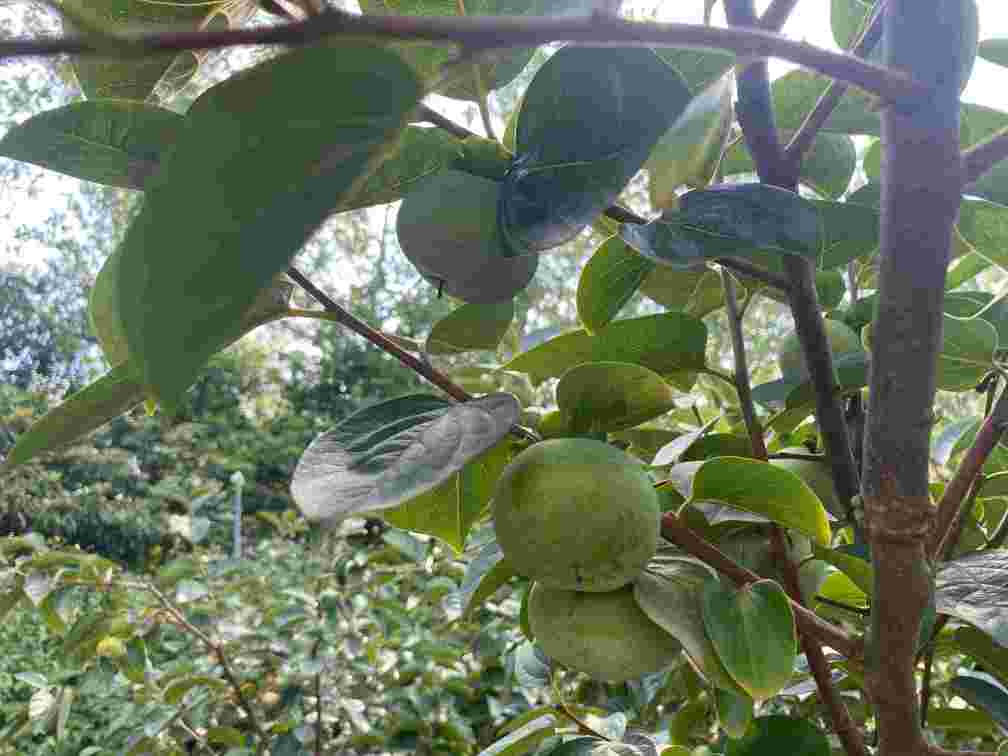

Supplement: Supplementary file 1 [file DataSheet1.zip › 2022-07-22 183454(96).jpg]

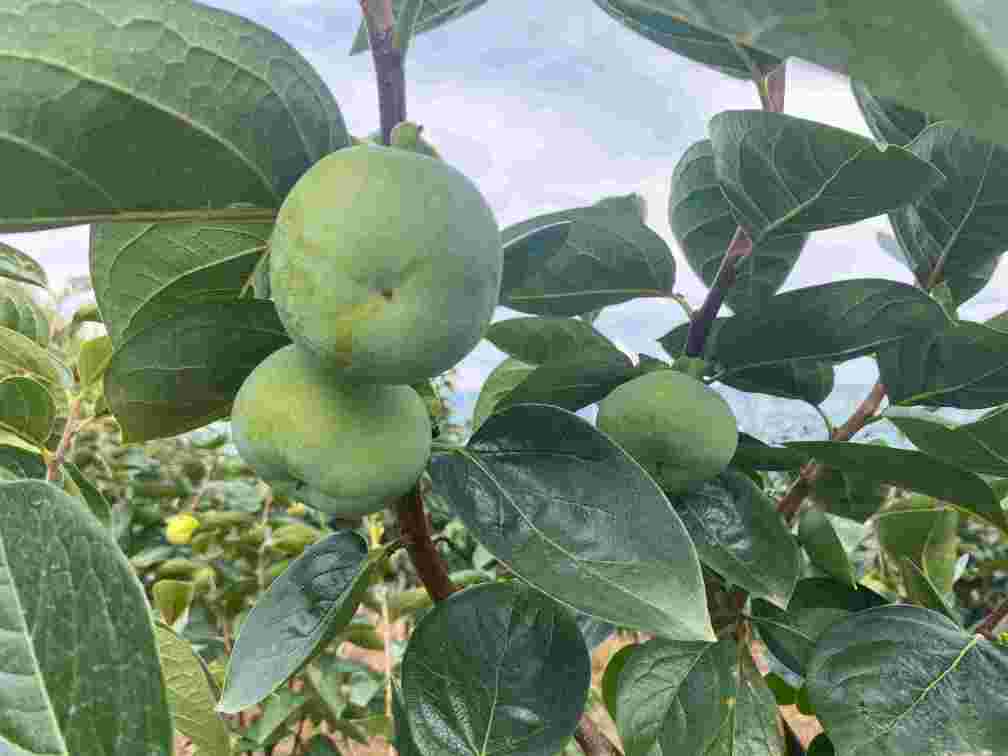

Supplement: Supplementary file 1 [file DataSheet1.zip › 2022-07-22 183454(97).jpg]

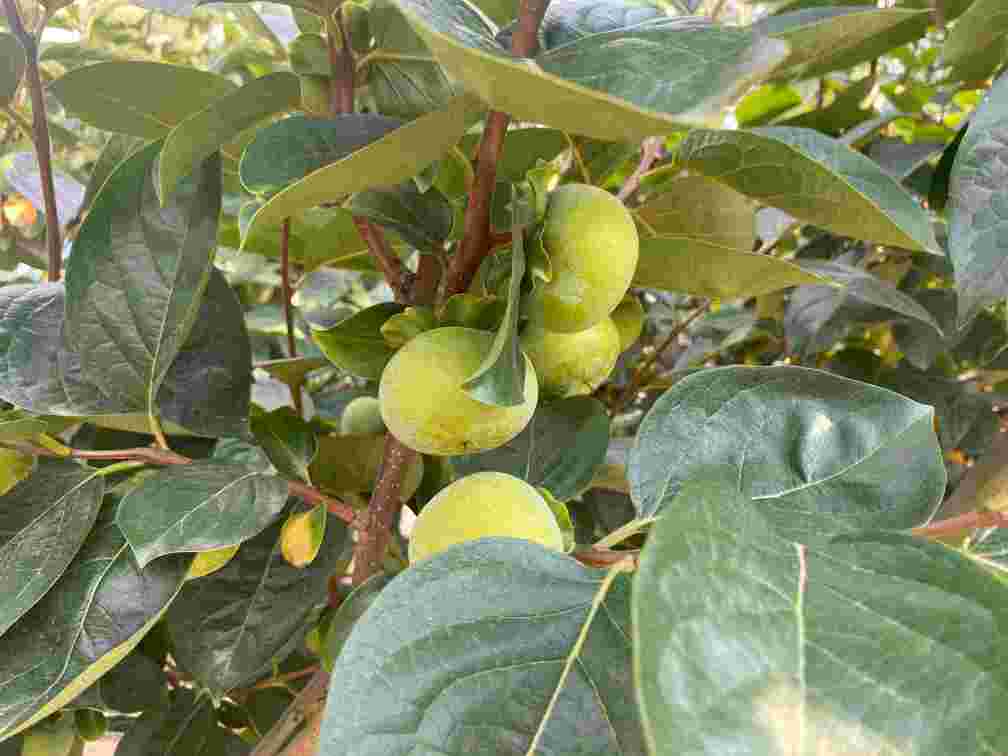

Supplement: Supplementary file 1 [file DataSheet1.zip › 2022-07-22 183454(99).jpg]

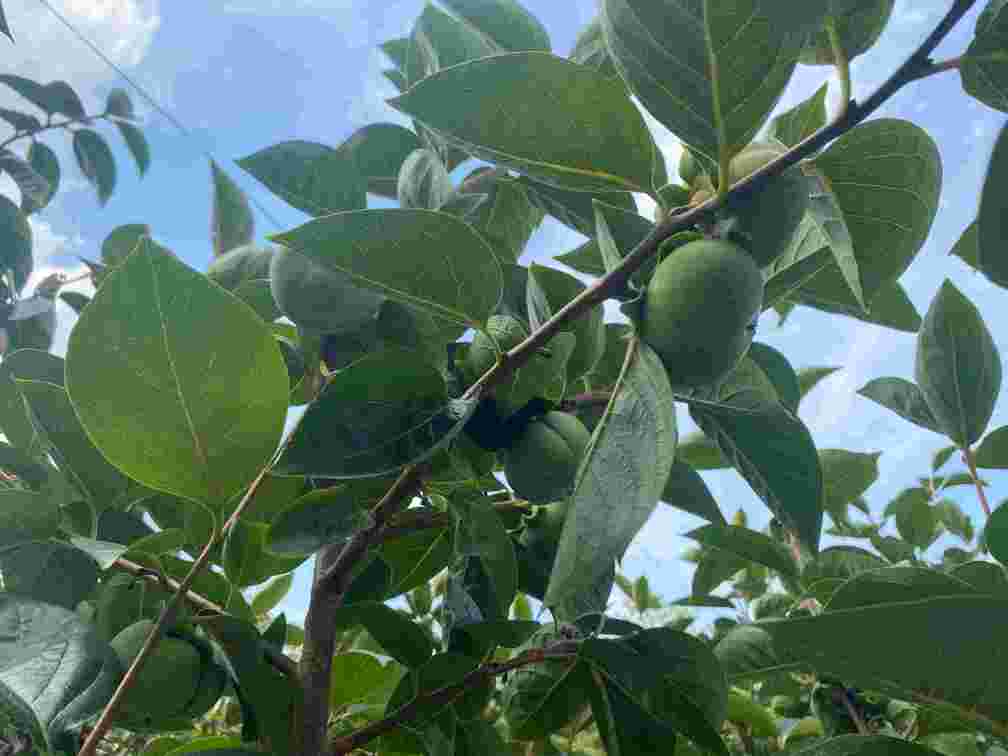

Supplement: Supplementary file 1 [file DataSheet1.zip › 2022-07-22 183454.jpg]

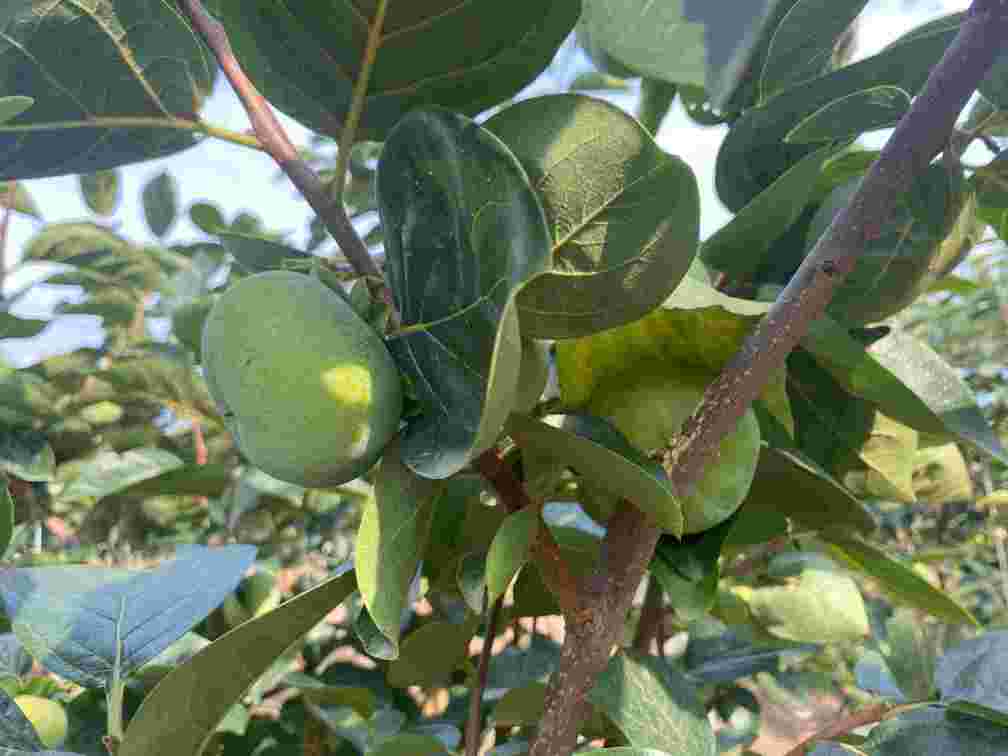

Supplement: Supplementary file 1 [file DataSheet1.zip › 2022-07-22 183454_20220722_183620.jpg]

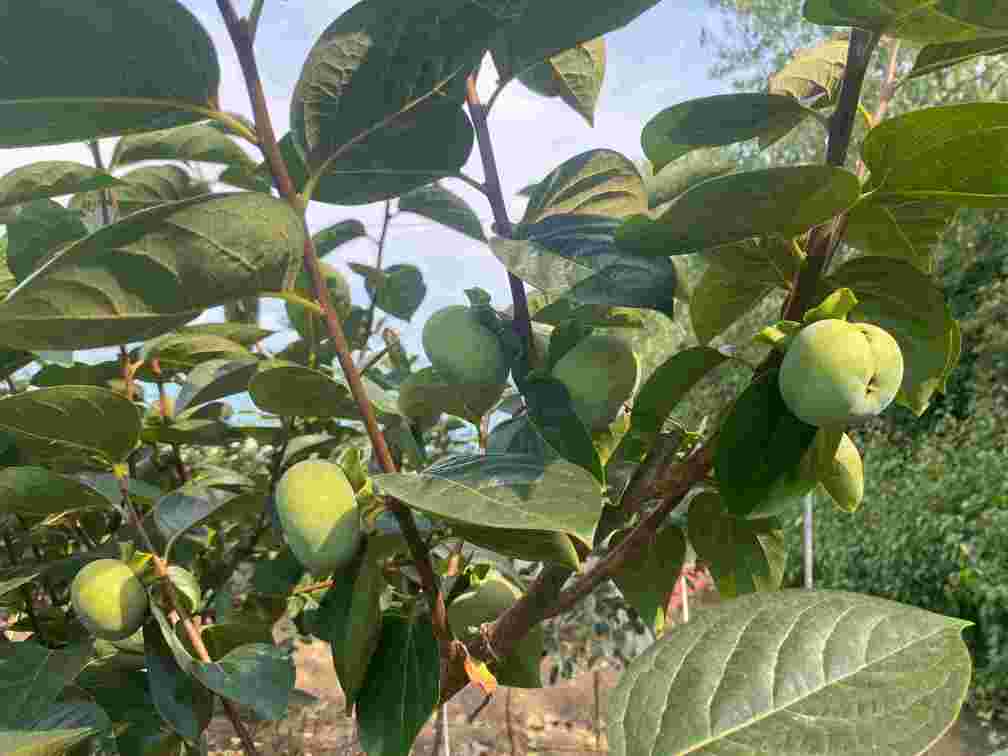

Supplement: Supplementary file 1 [file DataSheet1.zip › 2022-07-22 183454_20220722_183621.jpg]

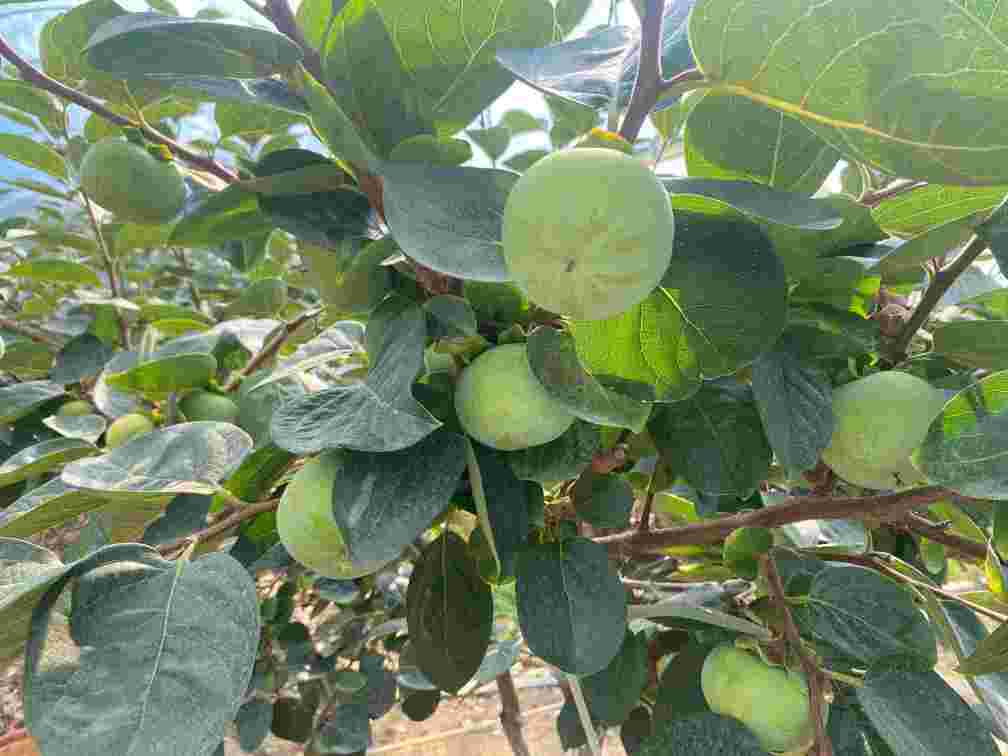

Supplement: Supplementary file 1 [file DataSheet1.zip › 2022-07-22 183454_20220722_183622.jpg]

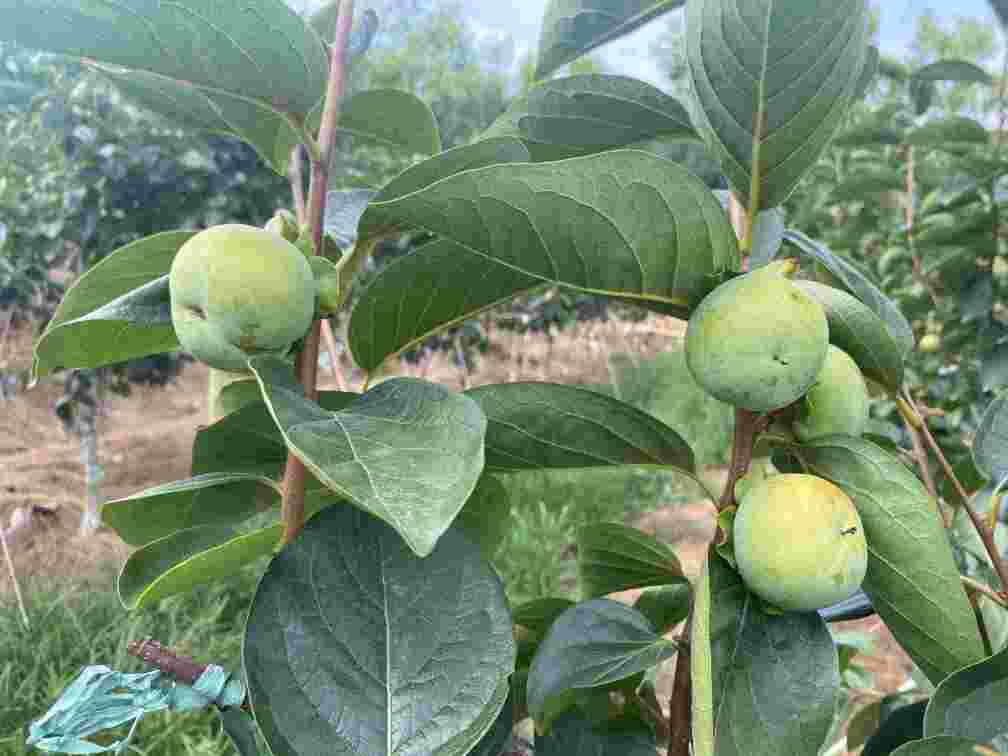

Supplement: Supplementary file 1 [file DataSheet1.zip › 2022-07-22 183454_20220722_183623.jpg]

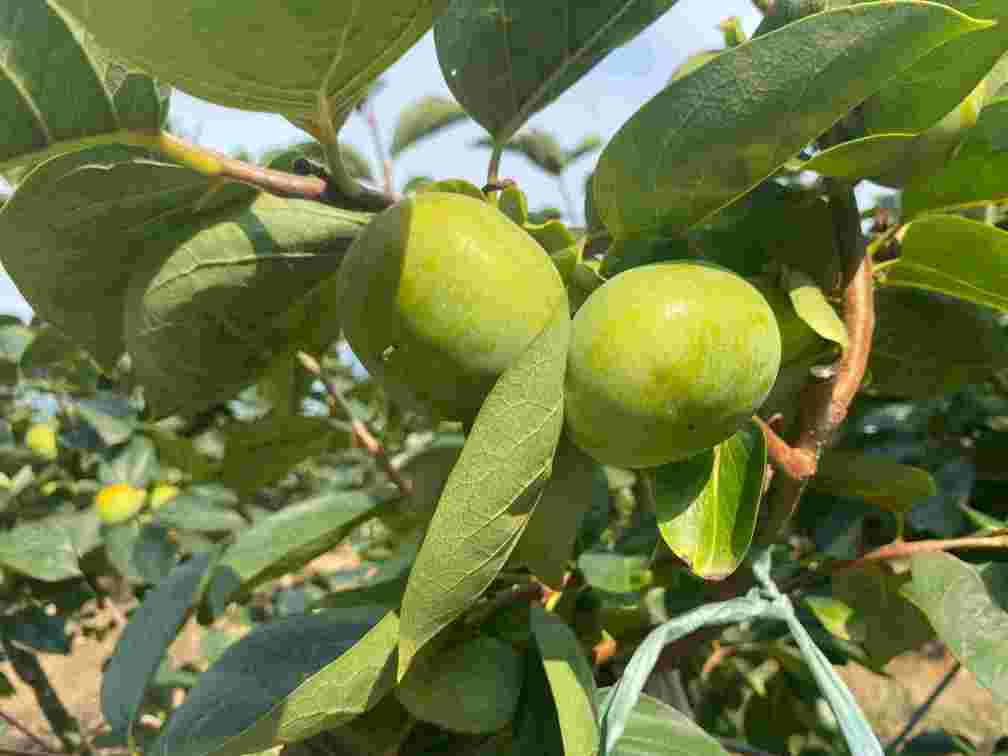

Supplement: Supplementary file 1 [file DataSheet1.zip › 2022-07-22 183454_20220722_183624.jpg]

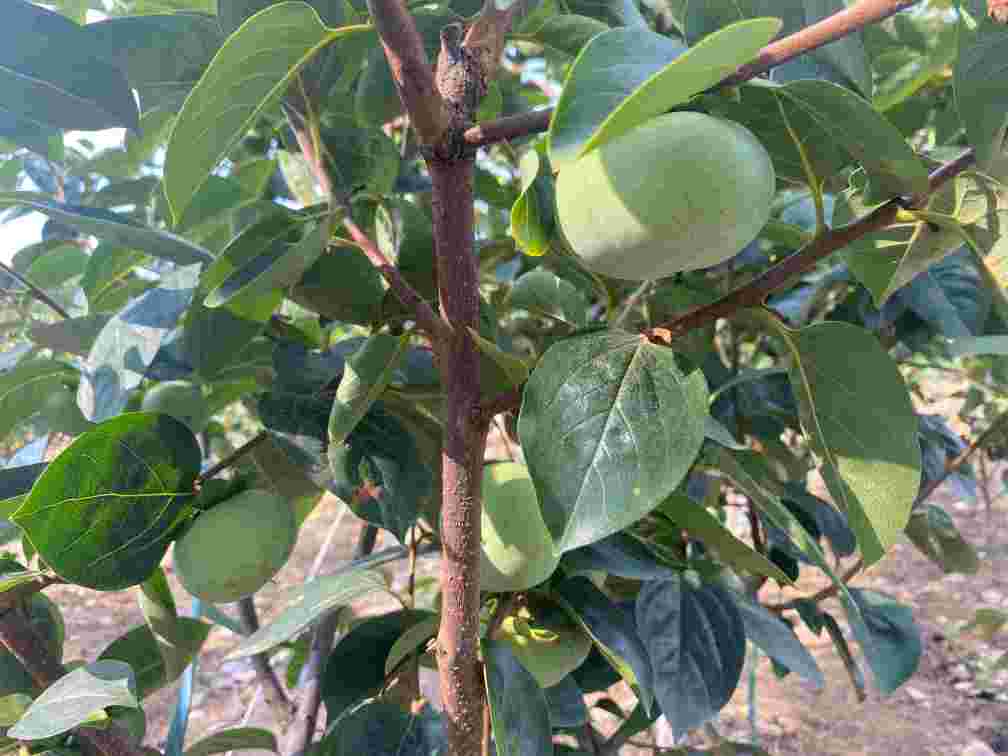

Supplement: Supplementary file 1 [file DataSheet1.zip › 2022-07-22 183454_20220722_183625.jpg]

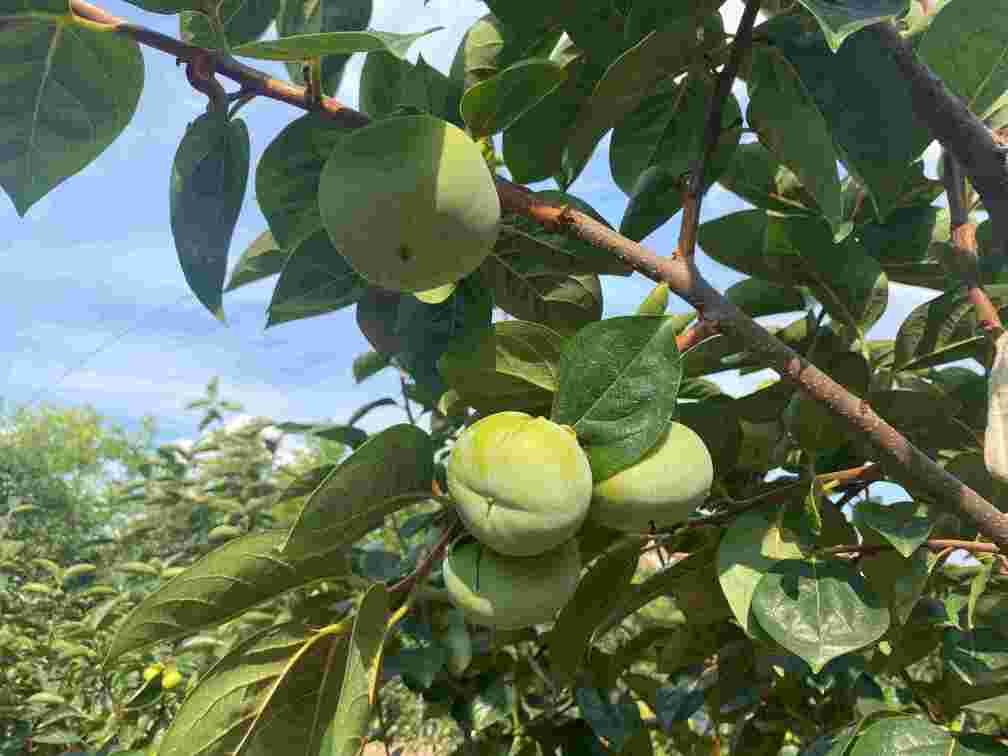

Supplement: Supplementary file 1 [file DataSheet1.zip › 2022-07-22 183454_20220722_183626.jpg]

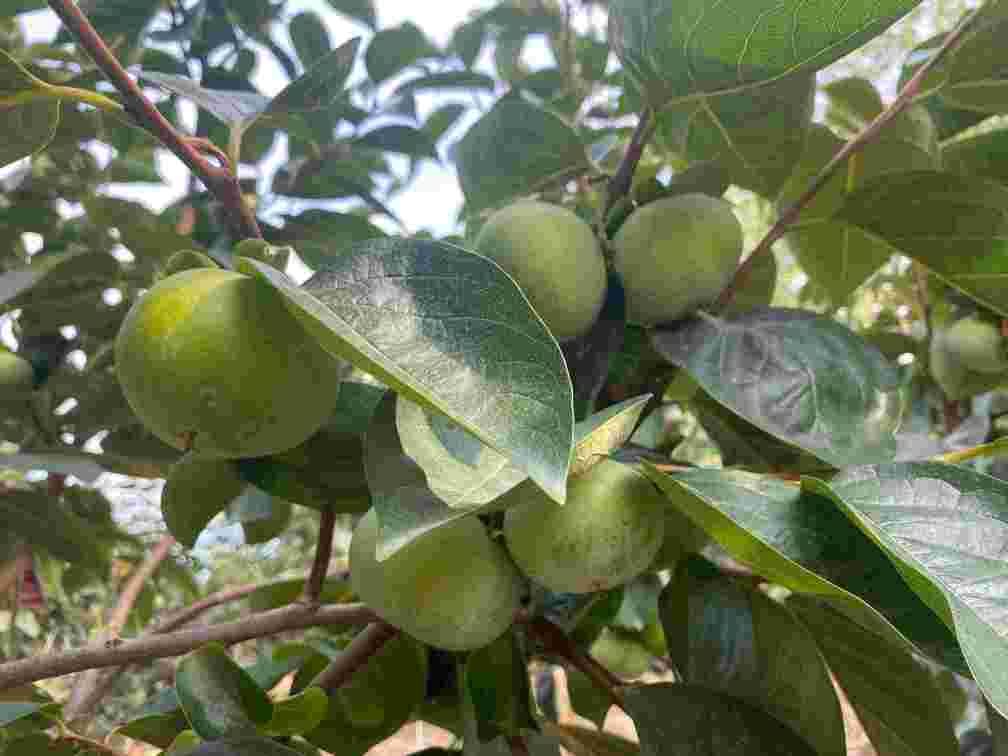

Supplement: Supplementary file 1 [file DataSheet1.zip › 2022-07-22 183454_20220722_183627.jpg]

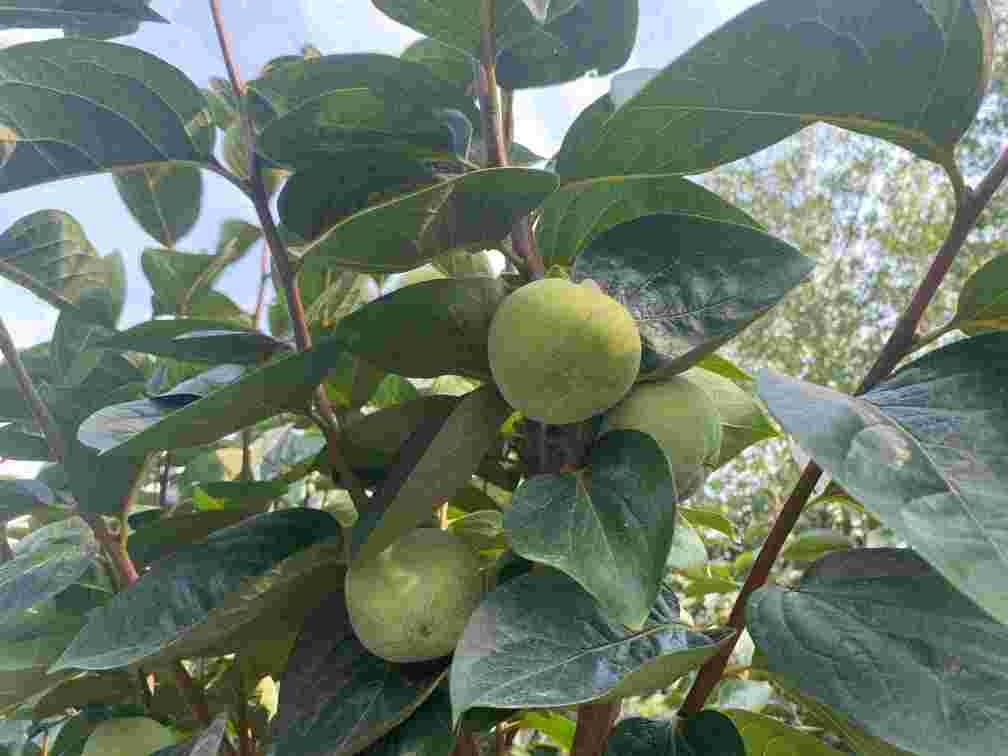

Supplement: Supplementary file 1 [file DataSheet1.zip › 2022-07-22 183454_20220722_183628.jpg]

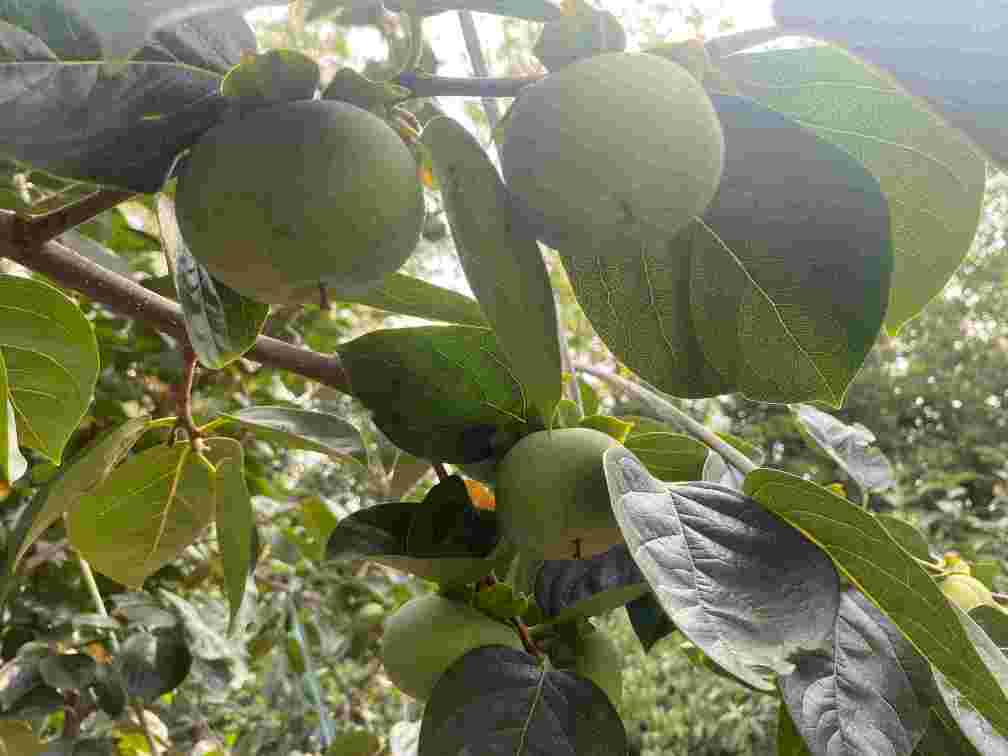

Supplement: Supplementary file 1 [file DataSheet1.zip › 2022-07-22 183454_20220722_183629.jpg]

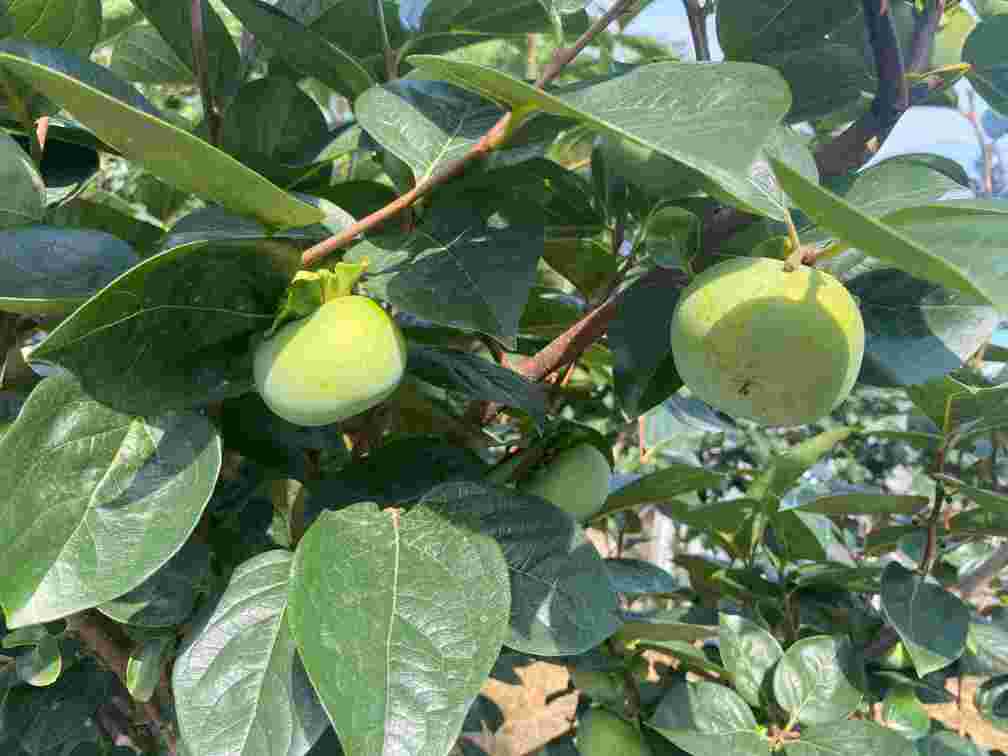

Supplement: Supplementary file 1 [file DataSheet1.zip › 2022-07-22 183454_20220722_183630.jpg]

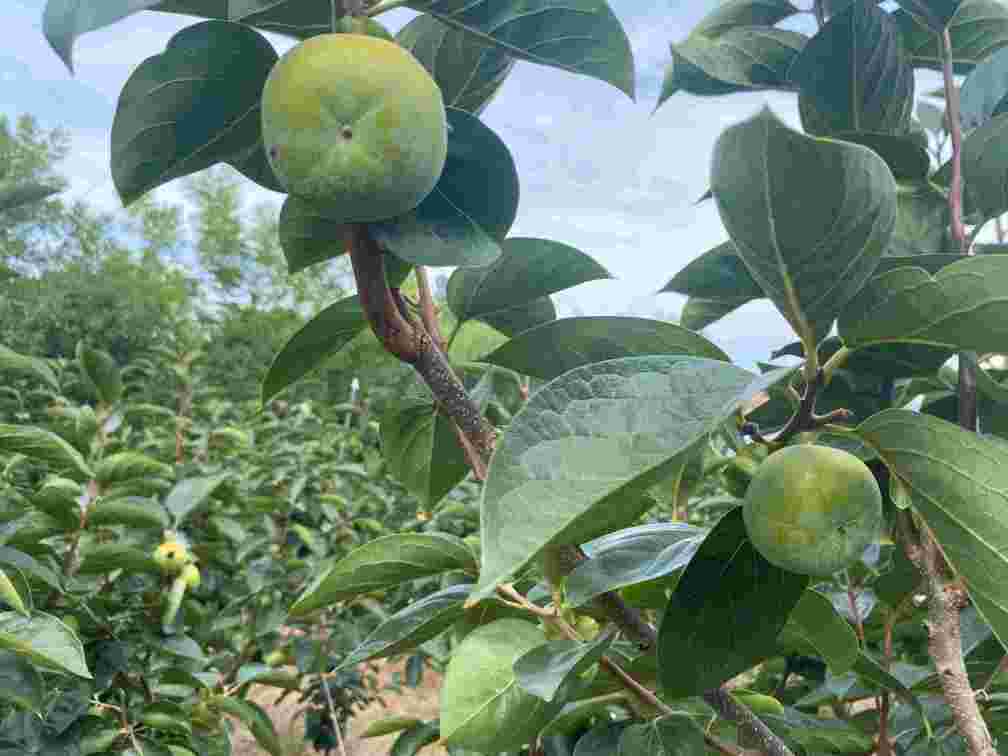

Supplement: Supplementary file 1 [file DataSheet1.zip › 2022-07-22 183454_20220722_183631.jpg]

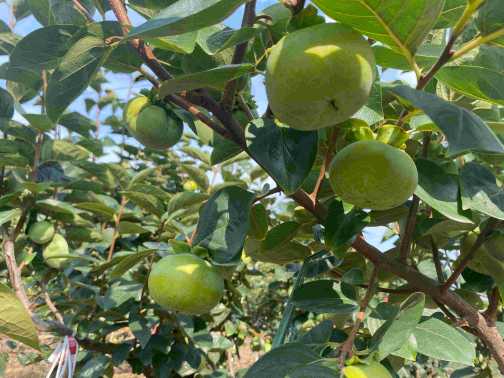

Supplement: Supplementary file 1 [file DataSheet1.zip › 2022-07-22 183454_20220722_183632.jpg]

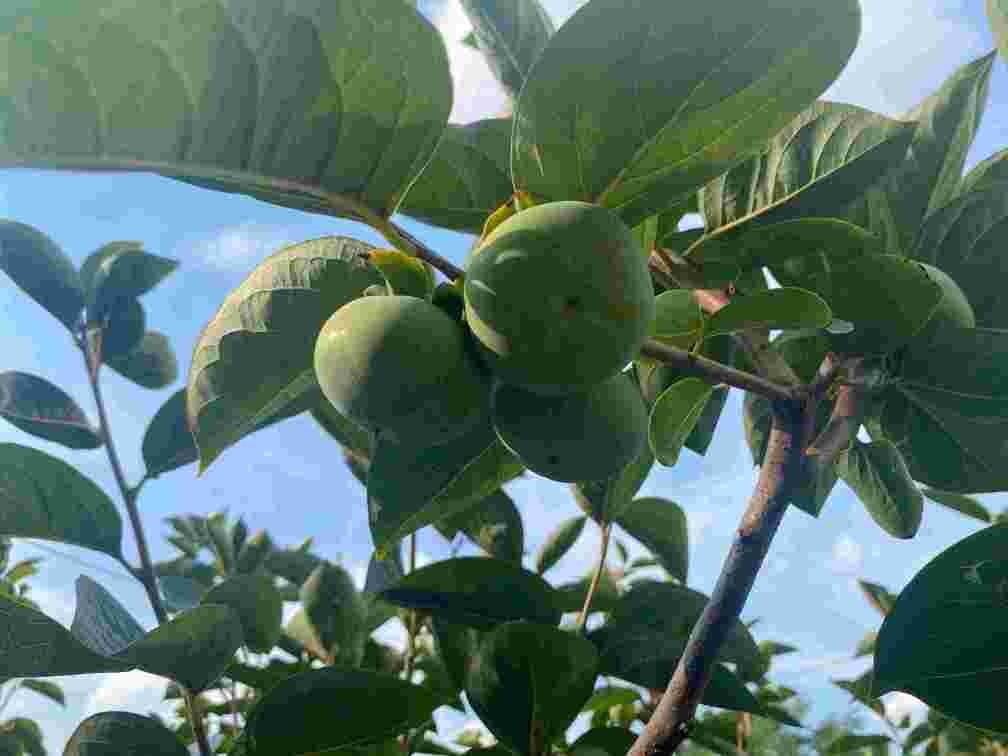

Supplement: Supplementary file 1 [file DataSheet1.zip › 2022-07-22 183454_20220722_183633.jpg]

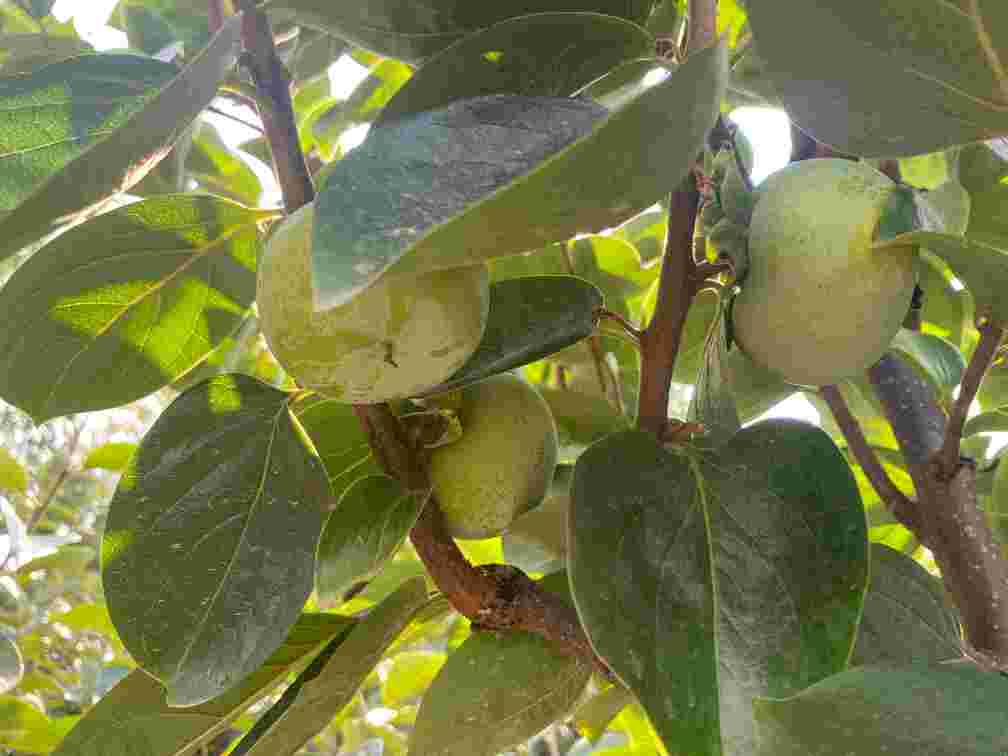

Supplement: Supplementary file 1 [file DataSheet1.zip › 2022-07-22 183454_20220722_183634.jpg]
